# Integrative Transcriptomic Profiling Identifies *TNF* and *IL1B* as Candidate Key Early-Response Genes in Macrophages Infected with Smooth *Brucella* Using a Comprehensive Bioinformatic Approach

**DOI:** 10.3390/biology14050579

**Published:** 2025-05-21

**Authors:** Xiaoyu Yang, Qiang Chen

**Affiliations:** Faculty of Animal Science and Technology, Yunnan Agricultural University, Kunming 650201, China; yangxiaoyu0124@126.com

**Keywords:** macrophage, smooth *Brucella*, transcriptional response, key gene, bioinformatics

## Abstract

Smooth *Brucella* are the main pathogenic bacteria that threaten human health and food safety. The early stage of smooth *Brucella* and macrophage interaction is an important phase, and smooth *Brucella* species elicit a dramatic transcriptional response in infected macrophages. However, the key transcriptional events are still obscure. This study aimed to identify key candidate response pathways and genes in macrophages infected with smooth *Brucella* at the early interaction stage. Three gene expression profiles including GSE21117, GSE5202, and GSE8385 were retrieved from the NCBI GEO database, and were integrated using comprehensive bioinformatics methods including gene set enrichment analysis, differentially expressed gene analysis, protein and protein interaction (PPI) network construction, and transcription factor prediction. The results showed that 16 up-regulated and 22 down-regulated pathways were identified, including six up-regulated immune-related pathways. A total of 41 up-regulated and four down-regulated genes were identified, and a PPI network including 31 nodes and 134 edges was constructed based on the interactive information of 45 dysregulated genes. A highly correlated module comprising 19 nodes and 103 edges was identified based on the topological features of the whole PPI network. Seven centrality analyses revealed that *Tnf* and *Il1b* were essential genes in the highly correlated module, and that the two essential genes were simultaneously enriched in eight significantly up-regulated pathways (including two immune-related pathways). *Bcl3* was predicted as a transcription factor in the highly correlated module, and may play regulatory roles in the transcription of *Tnf* and *Il1b* genes. The present study identified *Tnf* and *IL1b* as candidate key response genes in infected macrophages at the early stage of smooth *Brucella* and macrophage interaction, which contributes to a deeper understanding of the early key transcriptional events in macrophages infected with smooth *Brucella* species.

## Simple Summary

*Brucella* spp. are classic zoonotic intracellular pathogens. Infection in livestock such as cattle, sheep, and pigs often causes abortion and infertility, while in humans, it manifests as arthritis or Mediterranean fever, posing serious threats to both public health and livestock industry development. The smooth phenotype *Brucella* represents a crucial virulent form that establishes chronic infections in hosts. Therefore, elucidating the invasion mechanisms of smooth *Brucella* holds significant value for brucellosis diagnosis and treatment, particularly in deciphering the critical molecular events triggered during early infection. Our study employed a cross-*Brucella* species analysis strategy, integrating gene expression profiles from macrophages infected with smooth phenotype *B. melitensis* and *B. suis* strains through comprehensive bioinformatics approaches. We identified key early transcriptional response events in infected macrophages, including immune-related pathways and genes. These findings provide valuable insights into the transcriptional regulatory mechanisms during initial *Brucella*–macrophage interaction, advancing our understanding of brucellosis pathogenesis.

## 1. Introduction

*Brucella* spp. are Gram-negative facultative intracellular bacteria, and produce brucellosis in a variety of animal species including humans, pigs, sheep, goats, and cattle [1,2]. In humans, brucellosis causes a spectrum of clinical manifestations, ranging from common symptoms such as fever, fatigue, and joint pain to severe complications including endocarditis and neurological disorders [3]. Brucellosis has been globally acknowledged as a substantial public health concern, yet the precise annual incidence rate remains undetermined [4]. Recent studies demonstrate a higher annual incidence, with 1.6–2.1 million new brucellosis cases occurring worldwide [2,5], especially in the Middle East, Mediterranean, Asia, Africa, and America [2]. In domestic animals, brucellosis is closely associated with reproductive losses, often causing abortion and sterility in animals [6,7]. Annually, brucellosis imposes substantial economic burdens on both the healthcare and agricultural sectors in many countries, such as China and India [8,9]. Brucellosis seriously threatens human health and impedes agricultural economic development; elucidating its pathogenesis is crucial for treatment and prevention and the advancement of livestock production.

Currently, more than ten *Brucella* species have been reported [7], including *Brucella suis* (*B. suis*), *Brucella ovis* (*B. ovis*), *Brucella abortus* (*B. abortus*), *Brucella canis* (*B. canis*), *Brucella melitensis* (*B. melitensis*), *Brucella neotomae* (*B. neotomae*), *Brucella ceti* (*B. ceti*), *Brucella pinnipedialis* (*B. pinnipedialis*), *Brucella microti* (*B. microti*), *Brucella inopinata* (*B. inopinata*), *Brucella papionis* (*B. papionis*), and *Brucella vulpis* (*B. vulpis*). Among these *Brucella* species, four are pathogenic to humans, including *B. suis*, *B. melitensis*, *B. abortus*, and *B. canis* [10]. Their primary animal hosts are swine (*B. suis*), sheep and goats (*B. melitensis*), cattle (*B. abortus*), and dogs (*B. canis*), respectively [11,12]. Among the four *Brucella* species, *B. melitensis* and *B. suis* exhibit the highest pathogenicity, responsible for the most severe and moderately severe cases of brucellosis, respectively, whereas *B. abortus* and *B. canis* show moderate virulence and typically cause milder symptoms of the disease [10]. Given the significant health impact of brucellosis, particularly in patients with underlying cardiopulmonary conditions, the cornerstone of brucellosis management lies in rapid disease control to prevent complications and relapse. Consequently, accurate diagnosis, especially during the early (acute) phase, is of paramount importance. However, the non-specific clinical manifestations of brucellosis, which mimic other bacterial and viral infections, frequently lead to misdiagnosis in clinical practice. This often results in delayed treatment during the acute phase, allowing progression to chronic infection with multiple complications, and consequently, poorer prognosis [3]. Presently, the prevention and treatment of brucellosis primarily rely on vaccination and antibiotic therapy [2]. However, available vaccines exhibit inadequate protective efficacy and safety concerns, necessitating the development of safer and more effective vaccines. The establishment of early, rapid, and accurate diagnostic methods would enable combined vaccine–antibiotic therapy to serve as an effective strategy against brucellosis [10], but this strategy first demands the elucidation of *Brucella’s* infection mechanisms.

*B. melitensis* and *B. suis* are the two most pathogenic *Brucella* species, and exhibit strikingly similar genomic characteristics, with nearly identical genetics and gene organization [13]. Previous studies have shown that the open reading frames (ORFs) have 99% or higher sequence identity between *B. melitensis* and *B. suis* [13]. Despite their high genomic similarity, the two *Brucella* species exhibit distinct host tropisms. Genomic variations, including some insertion–deletion (indels) and polymorphic events encoding putative outer membrane proteins, may account for these host specificity differences. On the other hand, while the two *Brucella* species display different host specificities in animals, they share the common capability to cause human infections, which indicates that both *B. melitensis* and *B. suis* possess not only species-specific infection mechanisms, but also shared pathogenic strategies. The pathogenesis of *Brucella* species primarily relies on its virulence determinants, such as lipopolysaccharide (LPS), the type IV secretion system (T4SS), and the BvrR/Bvrs system [7]. Some studies have revealed that *B. melitensis* and *B. suis* share common virulence mechanisms, such as the T4SS and LPS [14,15,16,17], while exhibiting potential distinct pathogenic strategies [1,18]. Despite advances in understanding the pathogenesis of *Brucella* species, the pathogen’s unique absence of classical virulence factors such as exotoxins, cytolysins, and exoenzymes [2] results in exceptionally intricate infection mechanisms, with numerous unresolved questions remaining. It is vital to uncover the consistent infection characteristics for a greater understanding of the infection mechanisms. Therefore, further mechanistic studies are urgently needed.

Macrophages are the main targets of *Brucella* species, and attempt to eliminate the invading bacteria by triggering defense mechanisms. Once the target macrophages are infected, *Brucella* species enter the infected macrophages [19]. When *Brucella* species circumvent macrophage killing, *Brucella* species enter the intracellular life cycle and dramatically replicate in infected macrophages. Smooth *Brucella* species with an intact lipopolysacharide (LPS) O side chain is one major phenotype of *Brucella* species [20]. Unlike rough *Brucella* species, smooth *Brucella* species have been implicated in the virulence of target macrophages, producing enormous effects on animal immune systems and affecting disease processes [21]. Previous studies have demonstrated that smooth *Brucella* species inhibited macrophage apoptosis and impaired cytokine production [18,22,23], which facilitates the survival and replication of smooth *Brucella* species in infected macrophages. Furthermore, in vitro studies have found that the early stage of smooth *Brucella* species and macrophage interaction was the key phase, and that the majority of smooth *Brucella* species were killed and caused a more dramatic transcriptional response in the infected macrophages [20,24]. However, a small subset of *Brucella* species can evade immune surveillance, utilizing macrophages as host cells for intracellular survival and reproduction [25]. The persistent interaction forces the host to enter a chronic infection stage [20,25]. Studies have partially demonstrated that *Brucella* survive in macrophages by inhibiting the fusion of *Brucella*-containing vacuoles (BCVs) with lysosomes and by altering the maturation process of BCVs along the endocytic pathway [26,27]. However, the nature of the replicative compartment of *Brucella* in macrophages remains controversial [28]. Some evidence suggests that *Brucella* replicates within compartments exhibiting phagolysosomal characteristics [27], but this would imply the completion of phagosome maturation—a notion that appears contradictory to the observed inhibition of BCV–lysosome fusion. Thus, it is critical to clarify the interaction mechanism for a better understanding of the establishment of a chronic *Brucella* species infection at the early infection stage. Several independent studies have used gene expression profiles to identify key genes in the infected macrophages at the early stage of macrophage and smooth *Brucella* species interaction [1,18], and hundreds of differentially expressed genes (DEGs) were identified. However, comparative analysis is still lacking, and identifying consistent DEGs via integrative analysis is urgently required. 

Increasing datasets enable the systematic identification of conserved key genes inside the macrophages infected with smooth *Brucella* species at the early infection stage. In this study, we integrated three gene expression profiles obtained from the Gene Expression Omnibus (GEO) database of the National Center for Biotechnology Information (NCBI) to investigate the response mechanism of macrophage and smooth *Brucella* species interaction at the transcriptional level using comprehensive bioinformatic methods including gene set enrichment analysis (GSEA), differentially expressed gene analysis (DEGA), gene ontology (GO) analysis, protein and protein interaction (PPI) network analysis, highly correlated module analysis, and centrality analysis. The present study identified candidate key response genes in macrophages infected with smooth *Brucella* species at the early interaction stage, which contributes to a deeper understanding of the early response mechanisms of macrophages against smooth *Brucella* species infection.

## 2. Materials and Methods

### 2.1. Data Collection

The gene expression profiles were obtained from the NCBI GEO database as follows (http://www.ncbi.nlm.nih.gov/geo/, (accessed on 10 March 2018)): (1) phenotype for smooth *Brucella* species; (2) target cell line for macrophage; and (3) data with the same interaction stage. Finally, two gene expression profiles with accession numbers GSE21117 and GSE5202 were retrieved [1,18]. Two microarray data were separately produced using the GPL1261 platform (Affymetrix Mouse Genome 430 2.0 Array). In this study, two subsets related to the early interaction stage from GSE21117 and GSE5202 were selected to be integrated and reanalyzed. Each subset contained 3 infected macrophages samples (4 h time point post infection) and 3 uninfected macrophages samples (normal). The data subset related to *B. melitensis* in the GSE8385 dataset was retrieved to validate the results [22]. The main information about these subsets, such as the cell line, sample size, and infection time, was listed in Table 1.

### 2.2. Data Preprocessing

To improve the efficiency of data reanalyzing, all data were reprocessed using the same criteria. The data reprocessing was performed using the packages in the Bioconductor project (version 3.6, http://www.bioconductor.org/, (accessed on 16 March 2018)) based on R language [29]. All datasets were background-adjusted and normalized, and log2 probe-set intensities were calculated using the Robust Multichip Averaging (RMA) algorithm in the affy package (version 1.56.0) [30,31]. The interquartile range (IQR) was used to measure the data variability. In order to optimally preserve the functionally relevant low-abundance transcripts while ensuring analytical robustness, an IQR threshold of 0.5 was set based on the resulting distribution of the IQR values for all genes. Genes exhibiting IQR values < 0.5 were filtered out. The preprocessed data were used to perform the DEGA and GSEA.

### 2.3. DEGA of DEGs

The DEGs were identified using the limma package (version 3.32.7) in the Bioconductor project [32]. The limma package employs the *voom* method, liner modeling, and empirical Bayes moderation to assess the differential gene expression and can acquire more robust results, even in fewer microarrays. A false discovery rate (FDR) < 5% and a linear fold change >2 or <0.5(|logFC| > 1) were used as the cutoff criteria. The functional analyses of identified DEGs, including the GO and Reactome pathway (RP), were investigated using the online STRING database (version 11.0, https://string-db.org/, (accessed on 8 June 2018)) [33].

### 2.4. GSEA of KEGG Pathways

The GSEA of KEGG pathways was performed using the category package (version 2.34.2) in the Bioconductor project [34]. The purpose of performing GSEA was to determine whether the members of a gene set *S* were randomly distributed throughout the entire reference gene list *L* or were principally found at the top or bottom. An obvious merit of GSEA was the relative robustness to the noise and outliers in the data. For the GSEA of pathways, when multiple probe sets targeted the same gene, the probe set with the largest variability was kept for the next analysis, and the other probe sets were discarded. The gene sets with less than 10 genes were removed from the GSEA results. The t-statistic mean of the genes was computed in each KEGG pathway. A permutation test with 1000 times was implemented, and the KEGG pathways with *p* < 0.05 were identified to significantly change in the macrophages infected with smooth *Brucella* species [34].

To elucidate the interactive relationships between pathways and genes, a pathway–gene network was graphically represented using the open-source software platform Cytoscape software (version 3.7.0, http://www.cytoscape.org/, (accessed on 20 December 2018)) [35].

### 2.5. PPI Network and Analysis

The interaction relationships among DEGs encoding proteins were analyzed using a PPI network. PPI information was obtained using the online STRING database (version 11.0, https://string-db.org/, (accessed on 8 June 2018)) [33]. To evaluate the reliability of the predicted associations, the interaction score integrating multiple types of interaction evidence was utilized. To maintain prediction confidence and reduce false positive rates, we established an interaction score threshold of 0.4 (false positive rates < 0.15) based on the benchmarking standards specified in the published article [33]. A PPI network was constructed using Cytoscape software [35]. The PPI subnetwork (highly correlated module) was extracted from the whole PPI network using a Molecular COmplex DEtection (MCODE) algorithm based on the topological properties of whole PPI network, and a plugin MCODE (version 1.5.1) in the Cytoscape software was used to perform the MCODE analysis [36]. The threshold parameters were severely set for the Degree Cutoff = 6, Node Score Cutoff = 0.6, K-Core = 2, and Max. Depth = 100. The PPI subnetwork was used to perform a centrality analysis and transcription factor analysis. Seven centrality analyses were used to identify the core genes, including the Subgraph centrality, Degree centrality, Eigenvector centrality, Betweenness centrality, Network centrality, Information centrality, and Closeness centrality. Seven centrality metrics for each gene were computed based on their topological characteristics within the network, with detailed calculation methods for each metric provided in the published article [37]. Higher centrality scores indicate the greater functional importance of genes in the network, and all centrality analyses were performed using a plugin CytoNCA (version 2.1.6) in the Cytoscape software [37]. The genes with higher scores from each centrality method were identified as key genes, and the intersecting genes of key genes obtained via seven centrality methods were identified as essential genes. Transcription factor analysis was performed using an iRegulon plugin in the Cytoscape software and was used to identify the transcription factor in the highly correlated PPI subnetwork [38].

## 3. Results

### 3.1. KEGG Pathways Identification

**Figure 1:**
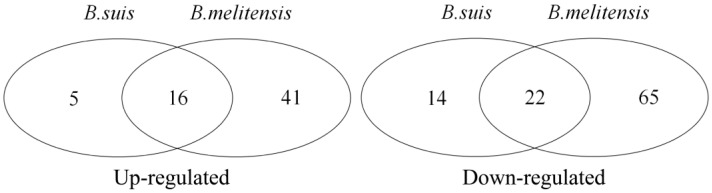
Overlapping pathways identification: 16 up-regulated and 22 down-regulated pathways were identified at early stage of smooth *Brucella* species and macrophage interaction.

According to *p* < 0.05, significantly regulated KEGG pathways were identified inside the infected macrophages at an early stage. In GSE21117, 21 up-regulated and 36 down-regulated pathways were identified (Table S1), and 57 up-regulated and 87 down-regulated pathways were identified in GSE5202 (Table S2). An overlap analysis showed that 16 common up-regulated and 22 common down-regulated pathways were identified as key response KEGG pathways (Figure 1). The common pathways were listed in Table 2.

The 16 up-regulated pathways were primarily associated with the immune system (including six pathways) and infectious diseases (including three pathways). The six up-regulated immune-related pathways were, separately, the toll-like receptor signaling pathway, NOD-like receptor signaling pathway, RIG-I-like receptor signaling pathway, cytosolic DNA-sensing pathway, T cell receptor signaling pathway, and B cell receptor signaling pathway. Importantly, four of these pathways were identified to significantly up-regulate in the GSE8385 dataset, including the toll-like receptor signaling pathway, NOD-like receptor signaling pathway, RIG-I-like receptor signaling pathway, and cytosolic DNA-sensing pathway.

The 22 key down-regulated pathways were mainly related to metabolism (including one global metabolic pathway), cancers (including seven pathways), cell growth and death (including two pathways), signal transduction (including three pathways), and translation (including two pathways). Two down-regulated pathways belonging to cell growth and death were separately cell cycle and oocyte meiosis.

**Figure 2:**
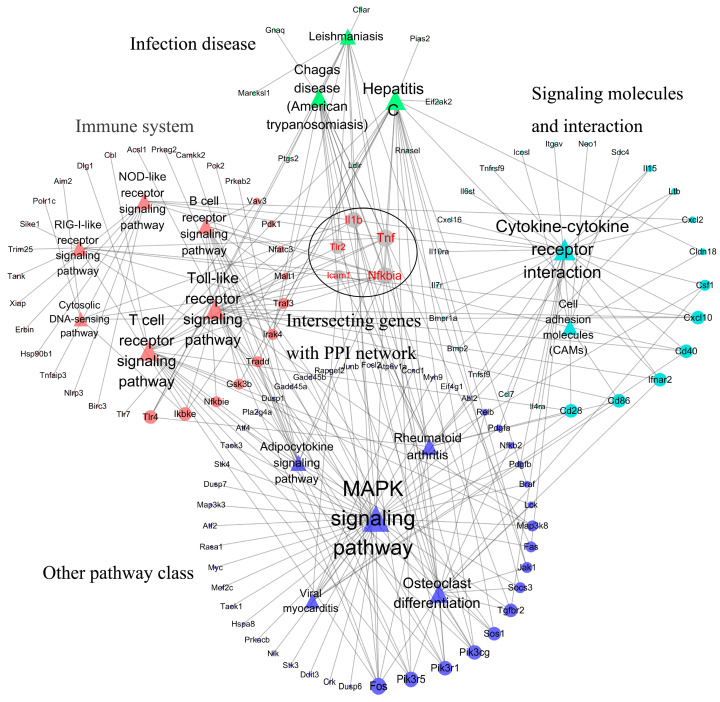
Relational network of pathways–genes in up-regulated pathways. The relational network included 16 up-regulated pathways and 112 up-regulated genes. The relational network mainly contained four pathway classes including immune system, infection disease, signaling molecules and interaction, and other pathway classes. *Tnf*, *Il1b*, *Tlr2*, *Nfkbia*, and *Icam1* genes participated in more KEGG pathways. A bigger node and font represented genes and pathways with more links.

**Figure 3:**
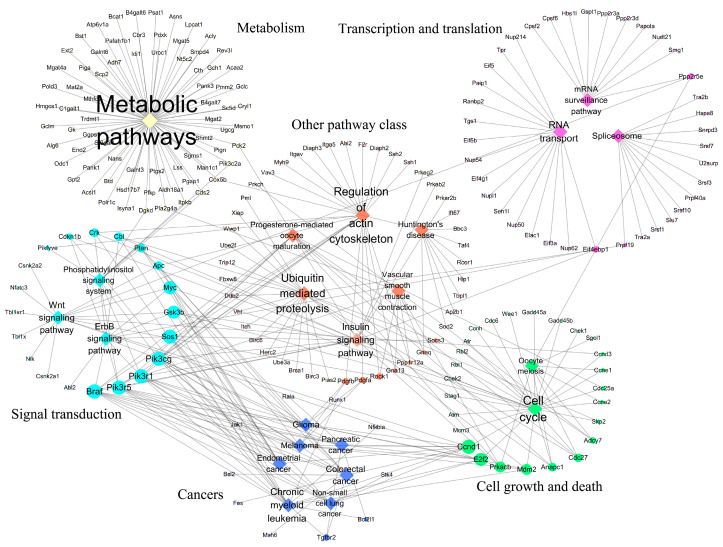
Relational network of pathways–genes in down-regulated pathways. The relational network included 22 down-regulated pathways and 211 up-regulated genes. The relational network mainly contained six pathway classes including metabolism, transcription and translation, signal transduction, cell growth and death, cancers, and other pathway class. Bigger node and font represented gene and pathway with more links.

To elucidate the interactive relationships among pathways and genes, the relational network among pathways and genes was established. Two relational networks including up-regulated and down-regulated pathways are shown in Figure 2 and Figure 3. In the up-regulated pathway–gene network, the immune-related pathways included most of the genes retrieved. In addition, five immune-related genes including *Tnf*, *Nfkbia*, *Il1b*, *Tlr2*, and *Icam1* participated in many of the pathways retrieved. In the down-regulated pathway–gene network, five pathway classes were identified, and metabolic pathways contained most of the genes retrieved.

### 3.2. DEG Identification and Functional Analyses

**Figure 4:**
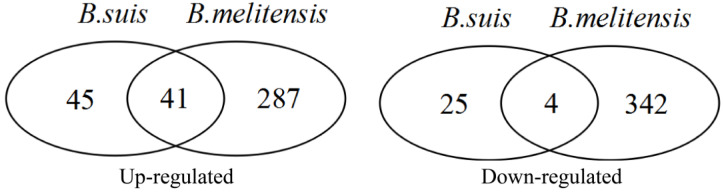
Overlapping differentially expressed genes identification: 41 up-regulated and 4 down-regulated differentially expressed genes were identified inside macrophages infected by smooth *Brucella* species at early infection stage.

DEGAs of two expression profiles including GSE21117 and GSE5202 were separately implemented using the limma package. According to FDR < 0.05 and |logFC| > 1 cutoff criteria, differentially expressed probes (DEPs) and DEGs corresponding to DEPs were identified. In the GSE21117 dataset, 117 up-regulated and 35 down-regulated DEPs were identified, and 86 up-regulated and 29 down-regulated DEGs were identified (Table S3). In the GSE5202 dataset, 444 up-regulated and 400 down-regulated DEPs corresponding to 328 up-regulated and 346 down-regulated DEGs were identified (Table S4). An overlap analysis showed that 54 common up-regulated and four common down-regulated DEPs (Table 3), corresponding to 41 common up-regulated and four common down-regulated DEGs were identified (Table 3, Figure 4). Among 45 common dysregulated genes (41 up- and four down-regulated), a transcription factor *Bcl3* (*B-cell lymphoma 3*) was identified.

GO analysis showed that 45 dysregulated genes were significantly enriched in 797 GO terms related to the biological process (BP) (Table S5), and the majority of significantly enriched BPs were associated with the biological process and regulation. The top five BPs with the most significant *p* value were cellular processes (GO:0009987, *p* = 4.50 × 10^−115^), the regulation of biological processes (GO:0050789, *p* = 3.30 × 10^−108^), the regulation of cellular processes (GO:0050794, *p* = 3.95 × 10^−105^), response to stimulus (GO:0050896, *p* = 8.62 × 10^−102^), and the positive regulation of biological processes (GO:0048518, *p* = 1.27 × 10^−90^).

RP analysis showed that 45 dysregulated genes were significantly enriched in 45 RPs (Table S6), and that the top five RPs with the most significant *p* value were mainly related to the immune system. The top five RPs were, separately, signal transduction (MMU-162582, *p* = 2.32 × 10^−37^), immune system (MMU-168256, *p* = 5.70 × 10^−32^), death receptor signaling (MMU-73887, *p* = 2.22 × 10^−15^), adaptive immune system (MMU-1280218, *p* = 2.22 × 10^−15^), and cytokine signaling in the immune system (MMU-1280215, *p* = 2.22 × 10^−15^).

### 3.3. PPI Network Construction

**Figure 5:**
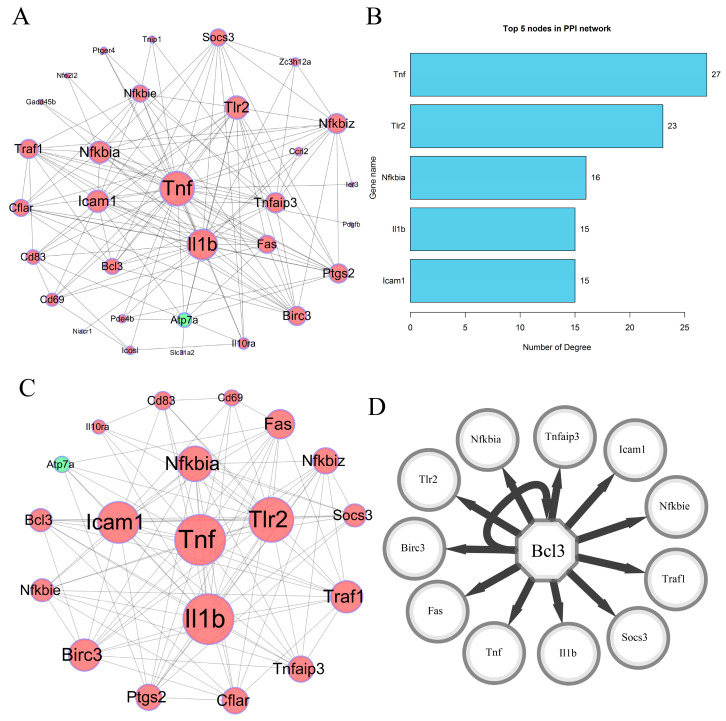
PPI network analysis of differentially expressed genes. (**A**) A PPI network was established with 31 nodes and 134 edges using online STRING database and Cytoscape software at a combined score of 0.4. The 31 nodes (genes) included 30 up-regulated genes and one down-regulated gene. Red and green nodes represented up-regulated and down-regulated genes, separately. Bigger nodes represented genes with more links. (**B**) Top five nodes with higher degrees in PPI network were identified. All five nodes were up-regulated genes. PPI—protein and protein interaction. (**C**) One PPI subnetwork with 19 nodes and 103 edges was extracted in whole PPI network. A total of 18 up-regulated genes and one down-regulated gene were included, and *Tnf*, *Il1b*, *Tlr2*, *Nfkbia*, and *Icam1* had more links. Bigger nodes represented genes with more links. (**D**) Transcription factor was predicted in PPI subnetwork, and transcription regulatory network was established. Bcl3 was identified as a transcription factor in PPI subnetwork, and 12 target genes including Bcl3 were predicted.

To reveal the interactive relationships of 45 dysregulated DEGs, a PPI network including 45 dysregulated DEGs was constructed according to the interactive information from the STRING database. At a combined score > 0.4, 31 DEGs (including 30 up-regulated genes and one down-regulated gene) had 134 interactive relationships, and a PPI network including 31 nodes and 134 edges was established (Figure 5A). Node degree analysis showed that the top five genes with the most links were, separately, *Tnf-α* (logFC = 2.94, *p* = 2.89 × 10^−3^ in GSE21117 and logFC = 1.79, *p* = 2.33 × 10^−8^ in GSE5202), *Il1b* (logFC = 1.96, *p* = 1.26 × 10^−2^ in GSE21117 and logFC = 1.47, *p* = 6.80 × 10^−8^ in GSE5202), *Tlr2* (logFC = 1.81, *p* = 1.02 × 10^−5^ in GSE21117 and logFC = 2.17, *p* = 2.36 × 10^−9^ in GSE5202), *Icam1*(logFC = 1.95, *p* = 1.10 × 10^−4^ in GSE21117 and logFC = 1.57, *p* = 1.43 × 10^−7^ in GSE5202), and *Nfkbiα* (logFC = 1.56, *p* = 1.02 × 10^−3^ in GSE21117 and logFC = 1.57, *p* = 1.66 × 10^−8^ in GSE5202). The degrees of these five genes were 27 (*Tnf-α*), 23 (*Il1b*), 16 (*Tlr2*), 15 (*Icam1*), and 15 (*Nfkbiα*) in the PPI network, respectively (Figure 5B).

### 3.4. Highly Correlated Module Analysis and Essential Gene Identification

To identify the key response genes at the early stage of smooth *Brucella* species and macrophage interaction, highly correlated module and centrality analyses were performed. The results showed that one highly correlated module with 19 nodes and 103 edges (Score = 11.444) was identified (Figure 5C). Centrality analysis showed that up-regulated *Tnf* and *Il-1b* were the top two genes based on comprehensive scores across seven centrality methods (Table 4), and were identified as candidate key response genes. The GSE8385 dataset showed that the two genes were significantly up-regulated. Transcription factor analysis showed that *Bcl3* (NES = 12.212) was predicted as a transcription factor of 12 target genes, including *Tnf* and *Il1b* (Figure 5D).

**Figure 6:**
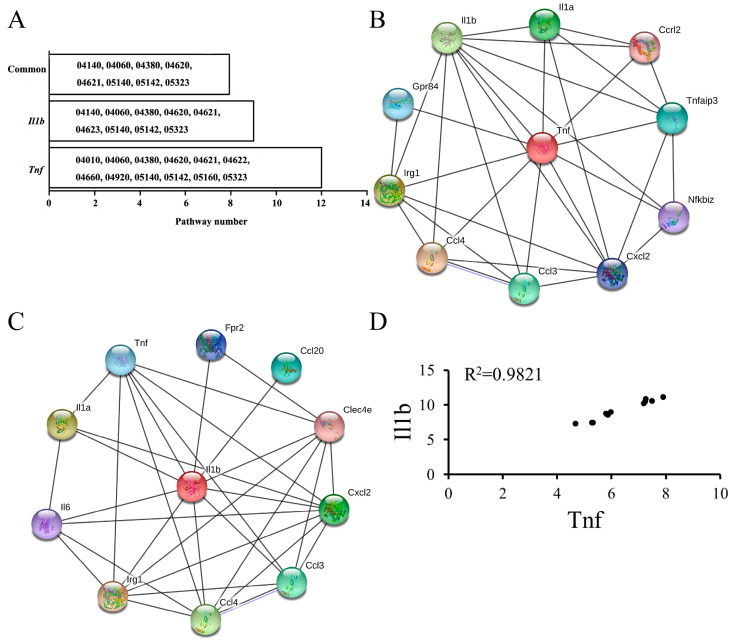
Two essential genes analysis. (**A**) *Tnf* and *Il1b* involved in KEGG pathways. *Tnf* and *Il1b* were separately involved in twelve and nine KEGG pathways, and eight KEGG pathways were common. (**B**) Interactive network of 10 genes most closely related to *Tnf*. (**C**) Interactive network of 10 genes most closely related to *Il1b*. (**D**) Pearson correlation analysis in expression between *Tnf* and *Il1b*. *Tnf* and *Il1b* had extremely strong positive correlation in expression in the normal and infected J774.A1 murine cell lines (R^2^ = 0.9821). KEGG, Kyoto Encyclopedia of Genes and Genomes. PPI—protein and protein interaction.

Furthermore, two essential genes, *Tnf* and *Il-1b*, were found to be enriched in twelve and nine pathways among sixteen common up-regulated pathways, respectively (Figure 6A). An overlap analysis showed that eight pathways were common (Figure 6A) and two pathways were related to the immune system. The two immune-related pathways were the toll-like receptor signaling pathway and NOD-like receptor signaling pathway. In addition, the two essential response genes were observed to be significantly enriched in the top five GO terms (Table S5), including cellular processes (GO:0009987), the regulation of biological processes (GO: 0050789), the regulation of cellular processes (GO: 0050794), response to stimulus (GO:0050896), and the positive regulation of biological processes (GO:0048518).

Network analysis on the basis of single genes showed that the two essential response genes had a stronger interactive relationship (Figure 6B,C). Pearson correlation analysis showed that the two essential response genes had an extremely strong positive correlation in expression in the normal and infected J774.A1murine cell lines (R^2^ = 0.9821, Figure 6D).

## 4. Discussion

The interaction of smooth *Brucella* species and macrophages is a dynamic process [1]. Studying molecular events is critical for understanding the pathogenesis of brucellosis in the process of smooth *Brucella* species and macrophage interaction. Especially, it is key to elucidate the early macrophage transcriptional response elicited by smooth *Brucella* species for understanding how a chronic infection is established [1]. *B. melitensis* and *B. suis*, as the two most detrimental *Brucella* species to both human health and the livestock industry, necessitate the urgent elucidation of their infection mechanisms. Previous single-species studies have identified some DEGs at the early stage of smooth *Brucella* species and macrophage interaction, advancing our understanding of brucellosis pathogenesis [1,18]. Nevertheless, the remarkable diversity and unique infectious strategies of *Brucella* species necessitate the elucidation of conserved infection mechanisms to fully decipher their distinct pathogenicity. Integrated cross-species analysis represents a powerful yet underutilized approach to address this knowledge gap. Currently, such comparative studies remain scarce. To bridge this gap, we performed a comprehensive bioinformatics analysis of two gene expression profiles, combining DEGA, GSEA, and GO analysis, PPI network construction, and highly correlated module and centrality analyses. Through a multi-dimensional bioinformatics analytical framework (differentially expressed gene identification→Gene functional enrichment analysis→Gene interaction network construction→Network topological feature characterization→Transcription factor prediction), we systematically investigated the translational events during the early-stage interactions between *Brucella* species and macrophages. Finally, 16 key up-regulated and 22 key down-regulated pathways were identified, including six key immune-related pathways. A total of 41 up-regulated and four down-regulated DEGs were identified. A PPI network with 31 nodes and 134 edges was established, and a highly correlated module with 19 nodes and 103 edges was extracted from the whole PPI network. *Tnf* and *Il1b* were identified as the candidate essential early-response genes of smooth *Brucella* and macrophage interaction. The findings indicate that six key immune-related pathways and two essential genes may play roles at the early stage of smooth *Brucella* species and macrophage interaction.

The six significantly up-regulated immune-related pathways were, separately, the toll-like receptor signaling pathway, NOD-like receptor signaling pathway, RIG-I-like receptor signaling pathway, cytosolic DNA-sensing pathway, T cell receptor signaling pathway, and B cell receptor signaling pathway. The toll-like receptor signaling pathway is a vital biological pathway generating innate immune responses and developing adaptive immunity [39,40]. In the pathway, the key genes *toll-like receptors* (*TLRs*) play critical roles in host resistance to infection, such as *Brucella* species infection [41,42]. For example, TLR6 was able to independently trigger an innate immune response against *B. abortus*, and further cooperated with TRL2 to activate the NF-kB signaling pathway [43]. Our study demonstrated that some *TLRs*, including *Tlr2*, were actively regulated in response to *Brucella* species, which indicates the important role of the toll-like receptor signaling pathway. The RIG-I-like receptor signaling pathway is an important pathway triggering an innate immune response by detecting viral pathogens [44,45,46]. Most published results showed that the RIG-I-like receptor signaling pathway plays a vital role in RNA virus recognition [47,48]. Lately, some studies have shown that the RIG-I-like receptor signaling pathway is a universal mechanism in defending against bacterial infection [49,50]. For example, *Yersinia pestis* infection induced the up-regulation of numerous genes in the pathway, including critical RIG-I-like receptors such as *RIG-I* and *MDA5* [50]. Despite these findings, no published studies reported the role of the pathway in macrophages defending against *Brucella* species. Our study showed that the RIG-I-like receptor signaling pathway was significantly up-regulated by smooth *Brucella* species induction, which indicates that the pathway may play an important role in triggering immune responses against smooth *Brucella* species. For many years, the cytosolic DNA-sensing pathway has been appreciated because exogenous cytosolic DNA could evoke a type I interferon response [51,52]. During host infection, most DNA-containing microbes released DNA into the cytoplasm and induced DNA immunity [53,54]. In this study, the cytosolic DNA-sensing pathway was up-regulated, which indicates that killed smooth *Brucella* species were disintegrated and released DNA into the infected macrophages to induce DNA immunity at the early interaction stage of smooth *Brucella* species and macrophages [55].

Many studies have focused on the key response genes in the processes of smooth *Brucella* species and macrophage interaction, and some DEGs have been identified as being involved in the interaction responses [1,18,22]. Some studies reported that two critical immune genes, *Tnf-α* and *Il1b*, were up-regulated at the early stage of smooth *Brucella* species and macrophage interaction via a DEGA method [1,56]. Our results further confirmed via a comprehensive bioinformatic method that the two genes played key roles as essential response genes between smooth *Brucella* species and macrophage interaction. As we know, Tnf-α is a cytokine mainly secreted by activated macrophages, and involved in systemic inflammation in an acute phase reaction, inducing cell death [57]. So, *Tnf-α* is usually used for anti-cancer therapy [57]. Recently, more and more studies have shown that *Tnf-α* functions in activating defense mechanisms [58,59]. Some studies have shown that *Tnf- α* was able to control the number of *Brucella* species in BALB/c mice [60], and that the production of *Tnf-α* was triggered through a Tlr2-dependent pathway in response to *B. abortus* [61]. Mentioned above, Tlr2 is a membrane protein playing a key role in innate immunity by recognizing bacterial lipoproteins and other microbial components. In our study, *Tlr2* was identified to significantly up-regulate, which further demonstrates that *Tnf-α* may play a defense role through *Tlr2*. *Il1b*, a member of the interleukin 1 family, is an important mediator of the inflammatory response produced by activated macrophages [18]. A large number of studies have proven the critical roles of *Il1b* in regulating immune responses [62,63]. In this study, *Il1b* was identified as an essential response gene via systematic analysis, which indicates that *Il1b* may play a key immune role in macrophages against *Brucella* species at the early infection stage. Two up-regulated genes related to immunity and inflammation may contribute to clearing smooth *Brucella* species at the early infection stage [18]. However, it should be noted that these two cytokine genes may exert dual roles during host defense against *Brucella* infection. While their pro-inflammatory effects contribute to bacterial clearance, sustained overexpression may lead to excessive inflammation and subsequent tissue damage. Therefore, during early infection, vaccines or adjuvants could be utilized to up-regulate their expression to control *Brucella* replication. Conversely, in later stages, the moderated down-regulation of these cytokines may help to mitigate tissue damage (e.g., arthritis) while maintaining adequate bacterial containment.

Transcription factor Bcl3, belonging to the I kappa B protein family, is the major regulator of the NFκB signaling pathway [64]. Bcl3 protein contains ankyrin repeat domains and transactivation domains, and has the ability to bind and regulate specific NF-κB dimers [65]. *Bcl3* has been identified as a candidate proto-oncogene [66], while accumulating evidence suggests its potential tumor-suppressive functions [67]. The dual role of *Bcl3* in tumorigenesis depends on the cellular and environmental context [67]. Recent studies have shown that *Bcl3* plays roles in regulating immunity and inflammation [65], and has an anti-apoptotic effect [68,69,70]. For example, *Bcl3* can reduce the sterile inflammatory response in pancreatic and biliary tissues [71]. *Bcl3* mediates cell proliferation by inducing PD-L1 expression in ovarian cancer [72]. Currently, no studies have demonstrated direct associations between *Bcl3* and *Brucella* infection. Several studies have shown regulatory relationships between *Bcl3* and *Tnf/Il1b* [67,73,74]. For example, *Bcl3* promotes *Tnf*-induced hepatocyte apoptosis, and plays a role in regulating *Tnf*-induced hepatic cell death [73]. The expression of *Tnf* is oscillatory along the estrous cycle in ICR mice, and its expression level is negatively associated with the presence of *Bcl3* [74]. In this study, *Bcl3* was predicted as a transcription factor to play roles in regulating *Tnf* and *Il1b* expression, but its precise regulatory mechanism remains unclear. Based on existing research evidence [69,70], we hypothesize that up-regulated *Bcl3* may inhibit macrophage apoptosis during the early stage of *Brucella* infection. Future studies should focus on elucidating the precise regulatory role of *Bcl3* in *Brucella* infection mechanisms.

Collectively, on the basis of a comprehensive bioinformatic analysis, we systematically investigated the transcriptional response events in macrophages infected with smooth *Brucella* species at an early interaction stage. The findings contribute to a better understanding of the transcriptional response mechanisms of macrophages against smooth *Brucella* species. Nevertheless, some limitations should be mentioned. First, the *B. abortus*-related gene expression data should have been included in this study. However, the lack of suitable datasets represents a significant limitation. Second, the functional roles of the two key candidate response genes *Tnf* and *Il1b* should be further investigated. Next, we will focus on the functional studies of two key candidate genes.

## 5. Conclusions

The study identified key candidate immune pathways and response genes in macrophages infected with smooth *Brucella* species during the early infection stage. The findings contribute to a deeper understanding of the early transcriptional response in macrophages infected with smooth *Brucella* species.

## Figures and Tables

**Table 1:** Experimental design of microarray experiments.

Dataset	Chip Platform	Probes	Murine Cell Line	*Brucella* Strain	Infection Time Point
GSE21117	GPL1261	45k	J774.A1	*B. suis* strain 1330	0 h
					4 h
GSE5202	GPL1261	45k	J774.A1	*B. melitensis* strain 16M	0 h
					4 h
GSE8385	GPL81	12,488	RAW 264.7	*B. melitensis*	0 h
					4 h

**Table 2:** Common dysregulated pathways identified via GSEA in GSE21117 and GSE5202.

Pathway ID	Pathway Name	Pathway Class	Enrichment Gene Number		Count
			GSE21117	GSE5202	
Up-regulated					
mmu04010	MAPK signaling pathway	Signal transduction	46	104	34
mmu04060	Cytokine–cytokine receptor interaction	Signaling molecules and interaction	27	51	23
mmu04380	Osteoclast differentiation	Development	22	52	17
mmu04514	Cell adhesion molecules (CAMs)	Signaling molecules and interaction	18	26	9
mmu04620	Toll-like receptor signaling pathway	Immune system	27	42	18
mmu04621	NOD-like receptor signaling pathway	Immune system	17	26	10
mmu04622	RIG-I-like receptor signaling pathway	Immune system	12	24	9
mmu04623	Cytosolic DNA-sensing pathway	Immune system	11	23	6
mmu04660	T cell receptor signaling pathway	Immune system	22	55	18
mmu04662	B cell receptor signaling pathway	Immune system	16	38	11
mmu04920	Adipocytokine signaling pathway	Endocrine system	14	30	10
mmu05140	Leishmaniasis	Infectious diseases	18	24	10
mmu05142	Chagas disease (American trypanosomiasis)	Infectious diseases	20	40	14
mmu05160	Hepatitis C	Infectious diseases	27	46	20
mmu05323	Rheumatoid arthritis	Immune diseases	17	25	12
mmu05416	Viral myocarditis	Cardiovascular diseases	13	25	8
Down-regulated					
mmu01100	Metabolic pathways	/	97	378	73
mmu03013	RNA transport	Translation	24	66	16
mmu03015	mRNA surveillance pathway	Translation	15	33	10
mmu03040	Spliceosome	Transcription	15	65	12
mmu04012	ErbB signaling pathway	Signal transduction	15	42	12
mmu04070	Phosphatidylinositol signaling system	Signal transduction	12	32	9
mmu04110	Cell cycle	Cell growth and death	33	68	26
mmu04114	Oocyte meiosis	Cell growth and death	13	42	8
mmu04120	Ubiquitin-mediated proteolysis	Folding, sorting, and degradation	27	66	22
mmu04270	Vascular smooth muscle contraction	Circulatory system	11	29	9
mmu04310	Wnt signaling pathway	Signal transduction	21	51	14
mmu04810	Regulation of actin cytoskeleton	Cell motility	31	71	23
mmu04910	Insulin signaling pathway	Endocrine system	19	63	15
mmu04914	Progesterone-mediated oocyte maturation	Endocrine system	11	40	9
mmu05016	Huntington’s disease	Neurodegenerative diseases	14	63	10
mmu05210	Colorectal cancer	Cancers	15	34	12
mmu05212	Pancreatic cancer	Cancers	14	40	10
mmu05213	Endometrial cancer	Cancers	11	28	10
mmu05214	Glioma	Cancers	14	32	11
mmu05218	Melanoma	Cancers	14	29	10
mmu05220	Chronic myeloid leukemia	Cancers	21	45	16
mmu05223	Non-small cell lung cancer	Cancers	11	28	8

GSE21117 and GSE5202 were accession numbers of two expression profiles. Abbreviations: GSEA, gene set enrichment analysis.

**Table 3:** Common dysregulated probes and corresponding genes identified in GSE21117 and GSE5202.

Number	Probe ID	Gene Symbol	LogFC		Adjusted *p*-Value	
			GSE21117	GSE5202	GSE21117	GSE5202
Up-regulated						
1	1418133_at	*Bcl3*	1.6376	1.2896	2.10 × 10^−5^	6.27 × 10^−7^
2	1421392_a_at	*Birc3*	1.3919	1.2825	1.52 × 10^−5^	1.84 × 10^−7^
3	1427736_a_at	*Ccrl2*	1.6309	1.2135	6.79 × 10^−5^	7.38 × 10^−7^
4	1428735_at	*Cd69*	2.9727	1.6583	2.97 × 10^−3^	1.55 × 10^−8^
5	1416111_at	*Cd83*	1.5845	2.4933	3.59 × 10^−5^	1.48 × 10^−9^
6	1428750_at	*Cdc42ep2*	1.0463	1.6527	8.58 × 10^−3^	1.07 × 10^−6^
7	1424996_at	*Cflar*	1.0649	1.5431	5.07 × 10^−5^	8.81 × 10^−8^
8	1449317_at	*Cflar*	1.2286	1.1349	3.86 × 10^−5^	1.81 × 10^−7^
9	1416010_a_at	*Ehd1*	1.2293	1.5092	2.59 × 10^−5^	3.57 × 10^−8^
10	1416011_x_at	*Ehd1*	1.3414	1.5224	259 × 10^−5^	3.93 × 10^−8^
11	1416012_at	*Ehd1*	1.2436	1.6730	6.46 × 10^−3^	1.46 × 10^−8^
12	1448175_at	*Ehd1*	1.3386	1.6506	2.29 × 10^−5^	1.55 × 10^−8^
13	1450744_at	*Ell2*	1.0320	1.3000	2.11 × 10^−3^	2.27 × 10^−7^
14	1448021_at	*Fam46c*	1.9514	1.7736	1.10 × 10^−5^	9.36 × 10^−8^
15	1460251_at	*Fas*	1.0830	2.2110	6.62 × 10^−5^	7.27 × 10^−9^
16	1449773_s_at	*Gadd45b*	1.6456	1.4205	2.04 × 10^−2^	1.81 × 10^−7^
17	1419721_at	*Hcar2*	1.1207	1.8983	1.77 × 10^−2^	2.77 × 10^−8^
18	1435626_a_at	*Herpud1*	1.9177	1.4910	1.22 × 10^−6^	3.85 × 10^−7^
19	1448185_at	*Herpud1*	2.1214	1.6464	1.22 × 10^−6^	1.66 × 10^−8^
20	1424067_at	*Icam1*	1.9478	1.5665	1.10 × 10^−5^	1.43 × 10^−7^
21	1419212_at	*Icosl*	1.8808	1.4179	3.24 × 10^−5^	2.77 × 10^−8^
22	1419647_a_at	*Ier3*	1.4769	1.5453	5.77 × 10^−6^	1.42 × 10^−8^
23	1448731_at	*Il10ra*	1.0320	1.3867	8.47 × 10^−5^	2.80 × 10^−7^
24	1449399_a_at	*Il1b*	1.9577	1.4708	1.26 × 10^−2^	6.80 × 10^−8^
25	1448306_at	*Nfkbia*	1.2173	1.9230	1.88 × 10^−3^	1.35 × 10^−8^
26	1449731_s_at	*Nfkbia*	1.5581	1.7485	1.02 × 10^−3^	1.54 × 10^−8^
27	1431843_a_at	*Nfkbie*	1.8611	1.3758	7.18 × 10^−6^	3.84 × 10^−7^
28	1458299_s_at	*Nfkbie*	1.9203	1.9025	1.22 × 10^−6^	8.02 × 10^−9^
29	1417483_at	*Nfkbiz*	3.8560	2.6059	1.71 × 10^−5^	8.00 × 10^−10^
30	1448728_a_at	*Nfkbiz*	2.7324	2.4021	1.50 × 10^−5^	2.06 × 10^−9^
31	1422474_at	*Pde4b*	1.3013	1.3126	4.47 × 10^−5^	2.04 × 10^−7^
32	1450413_at	*Pdgfb*	1.6909	1.6032	1.54 × 10^−3^	1.92 × 10^−8^
33	1450414_at	*Pdgfb*	1.5424	1.0075	7.70 × 10^−5^	1.01 × 10^−6^
34	1417801_a_at	*Ppfibp2*	1.2448	1.1562	5.07 × 10^−5^	9.74 × 10^−7^
35	1424208_at	*Ptger4*	1.0394	2.0903	1.37 × 10^−2^	4.41 × 10^−9^
36	1417263_at	*Ptgs2*	1.9088	1.7250	4.86 × 10^−2^	7.70 × 10^−8^
37	1423134_at	*Rilpl2*	1.0142	1.4033	9.48 × 10^−5^	4.12 × 10^−8^
38	1432478_a_at	*Rnf19b*	1.4471	1.4949	9.28 × 10^−5^	4.42 × 10^−8^
39	1435226_at	*Rnf19b*	1.3508	1.2286	8.32 × 10^−5^	1.70 × 10^−7^
40	1422054_a_at	*Skil*	1.1581	1.2304	8.32 × 10^−5^	7.05 × 10^−7^
41	1452214_at	*Skil*	1.1702	1.7727	1.50 × 10^−5^	7.27 × 10^−9^
42	1416654_at	*Slc31a2*	1.6967	2.0730	3.93 × 10^−5^	1.02 × 10^−8^
43	1453721_a_at	*Slc31a2*	1.1741	2.2449	3.68 × 10^−3^	1.06 × 10^−9^
44	1416576_at	*Socs3*	1.6717	1.1579	1.48 × 10^−3^	5.77 × 10^−7^
45	1455899_x_at	*Socs3*	4.0894	2.3810	8.01 × 10^−5^	8.00 × 10^−10^
46	1456212_x_at	*Socs3*	3.6576	1.9634	1.88 × 10^−5^	2.74 × 10^−8^
47	1419132_at	*Tlr2*	1.8123	2.1650	1.02 × 10^−5^	2.36 × 10^−9^
48	1419607_at	*Tnf*	2.9434	1.7900	2.89 × 10^−9^	2.33 × 10^−8^
49	1433699_at	*Tnfaip3*	1.9794	2.3763	2.71 × 10^−3^	1.32 × 10^−9^
50	1450829_at	*Tnfaip3*	1.1995	1.1096	5.81 × 10^−3^	7.50 × 10^−7^
51	1427689_a_at	*Tnip1*	1.5081	1.5509	2.38 × 10^−5^	4.83 × 10^−8^
52	1423602_at	*Traf1*	1.6793	2.2973	2.21 × 10^−5^	1.95 × 10^−7^
53	1427348_at	*Zc3h12a*	1.2715	1.7723	7.72 × 10^−5^	1.04 × 10^−6^
54	1444402_at	*Zc3h12c*	2.9487	1.8413	1.52 × 10^−5^	4.68 × 10^−9^
Down-regulated						
1	1418774_a_at	*Atp7a*	−1.3005	−1.0209	1.48 × 10^−5^	1.95 × 10^−6^
2	1415834_at	*Dusp6*	−1.1775	−1.6091	3.70 × 10^−3^	8.99 × 10^−8^
3	1448890_at	*Klf2*	−1.0636	−1.9034	8.58 × 10^−3^	3.57 × 10^−8^
4	1427285_s_at	*Malat1*	−1.1471	−1.1179	7.27 × 10^−5^	5.10 × 10^−5^

**Table 4:** Centrality scores of genes in the subnetwork via seven centrality methods.

Rank	Gene	Subgraph	Degree	Eigenvector	Information	Betweenness	Closeness	Network
1	*Tnf*	28,456.67	18	0.3470	4.8312	310.15	0.9091	25.49
2	*Il1b*	26,182.86	18	0.3470	4.7046	158.31	0.8108	20.84
3	*Tlr2*	17,841.82	16	0.3470	4.3743	27.25	0.6818	12.61
4	*Icam1*	16,906.33	15	0.3470	4.3095	19.51	0.6667	12.06
5	*Nfkbia*	16,117.95	13	0.3470	4.3095	34.27	0.6522	12.40
6	*Birc3*	13,404.47	12	0.3470	4.0727	3.14	0.6122	10.26
7	*Traf1*	12,295.37	12	0.3470	4.0727	6.24	0.6122	9.26
8	*Tnfaip3*	11,955.47	10	0.3470	4.1597	22.63	0.6250	10.35
9	*Nfkbiz*	11,372.57	10	0.3470	4.1597	26.64	0.6250	9.89
10	*Fas*	10,675.32	11	0.3470	3.9756	7.70	0.6122	8.55
11	*Ptgs2*	10,368.22	10	0.3470	4.0727	15.88	0.6250	9.42
12	*Nfkbie*	9154.65	9	0.3470	3.8666	6.54	0.5882	8.33
13	*Cflar*	9116.53	10	0.3470	3.8666	2.96	0.5882	7.61
14	*Bcl3*	8139.34	9	0.3470	3.7435	2.08	0.5769	7.00
15	*Socs3*	8047.73	9	0.3470	3.8666	9.93	0.5882	7.21
16	*Cd83*	4734.80	7	0.3470	3.6033	5.47	0.5660	6.35
17	*Atp7a*	4670.12	6	0.3470	3.6033	59.50	0.5769	5.71
18	*Cd69*	4128.84	6	0.3470	3.4422	3.75	0.5556	5.67
19	*Il10ra*	2774.70	5	0.3470	3.2550	3.40	0.5455	4.67

## Data Availability

The gene expression profiles used to support the findings of this study were deposited in the NCBI GEO database, and were freely downloaded according to accession numbers GSE21117, GSE5202, and GSE8385.

## Supplementary Materials

The following supporting information can be downloaded at: https://www.mdpi.com/article/10.3390/biology14050579/s1, Table S1: Dysregulated pathways inside infected macrophages with smooth *B. suis*. Table S2: Dysregulated pathways inside infected macrophages with smooth *B. melitensis*. Table S3: Differentially expressed probes and genes inside infected macrophages with smooth *B. suis*. Table S4: Differentially expressed probes and genes inside infected macrophages with smooth *B. melitensis*. Table S5: GO terms enriched by 45 common differentially expressed genes. Table S6: Reactome pathways enriched by 45 common differentially expressed genes.
