# Supplementary material for: Integrative Transcriptomic Profiling Identifies TNF and IL1B as Candidate Key Early-Response Genes in Macrophages Infected with Smooth Brucella Using a Comprehensive Bioinformatic Approach
Source: Biology (Basel). 2025 May 21;14(5):579. doi: 10.3390/biology14050579 (PMC12109160; doi:10.3390/biology14050579)
Supplement: Supplementary file 1 [file biology-14-00579-s001.zip › Table S1.pdf]

Table S1 Dysregulated pathways inside infected macrophages with smooth B.suis

At 4h interaction time-point					
**Up-regulated pathways**					
**Entry**	**Pathway_names**	**p value**	**Class**	**Gene number**	**Gene**
04010	MAPK signaling pathway	0	Signal transduction	46	Dusp6, Crk, Jun, Ddit3, Relb, Stk3, Pdgfa, Nlk, Map3k8, Rela, Mapk1, Tnf, Prkacb, Hspa8, Fos, Max, Taok1, Mef2c, Myc, Rasgrp4, Nfkb2, Tgfbr2, Rasa1, Atf2, Map3k3, Dusp7, Stk4, Sos1, Taok3, Rasgrp3, Cacng7, Dusp16, Fgf1, Atf4, Pla2g4a, Dusp1, Il1b, Gadd45a, Gadd45b, Pdgfb, Dusp2, Tab2, Rapgef2, Gadd45g, Braf, Fas
04060	Cytokine-cytokine receptor interaction	0	Signaling molecules and interaction	27	Ccl5, Il15, Pdgfa, Cxcl10, Ltb, Ccl3, Tnf, Il4ra, Ccl7, Ccl4, Tnfsf9, Bmp2, Bmpr1a, Tgfbr2, Cd40, Ifnar2, Il7r, Il10ra, Cxcl16, Il1b, Cxcl2, Pdgfb, Il6ra, Il6st, Csf1, Fas, Tnfrsf9
04115	p53 signaling pathway	0	Cell growth and death	23	Ccnd3, Cd82, Ccng2, Ccne1, Ccnd1, Pmaip1, Atm, Cdkn1a, Ccne2, Chek2, Bbc3, Sesn2, Ddb2, Atr, Gadd45a, Gadd45b, Ccng1, Chek1, Gadd45g, Pten, Mdm2, Fas, Cdk6
04141	Protein processing in endoplasmic reticulum	0	Folding, sorting and degradation	25	Dnajb1, Ddit3, Xbp1, Hspa8, Dnajb12, Eif2ak2, Hsph1, Mbtps1, Uggt1, Atxn3, Derl2, Man1c1, Bcl2, Derl1, Ubqlnl, Hsp90b1, Sec62, Atf4, Herpud1, Ppp1r15a, Ubqln4, Dnajc5, Dnajc3, Ero1lb, Nfe2l2
04210	Apoptosis	0	Cell growth and death	21	Birc2, Rela, Tnf, Prkacb, Bcl2l1, Atm, Birc3, Pik3cg, Pik3r1, Endod1, Tradd, Pik3r5, Irak2, Bcl2, Prkar2b, Cflar, Il1b, Nfkbia, Irak4, Xiap, Fas
04380	Osteoclast differentiation	0	Development	22	Junb, Jun, Relb, Rela, Mapk1, Tnf, Pik3cg, Fos, Lck, Pik3r1, Nfkb2, Tgfbr2, Jak1, Pik3r5, Fosl2, Ifnar2, Il1b, Nfkbia, Sqstm1, Tab2, Socs3, Csf1
04514	Cell adhesion molecules (CAMs)	0	Signaling molecules and interaction	18	Icosl, H2-Oa, Cd274, Cd86, Itgav, Cdh5, H2-T24, Itga6, Icam1, Cldn18, Cd28, Cd40, Alcam, Neo1, Sdc4, Cldn1, Itga4, H2-DMa

04620	Toll-like receptor signaling pathway	0	Immune system	27	Irf7, Jun, Ikbke, Ccl5, Traf3, Cxcl10, Tlr2, Map3k8, Rela, Ccl3, Mapk1, Tnf, Cd86, Ccl4, Tlr7, Pik3cg, Fos, Pik3r1, Pik3r5, Cd40, Ifnar2, Tlr4, Il1b, Nfkbia, Tlr8, Tab2, Irak4
04621	NOD-like receptor signaling pathway	0	Immune system	17	Ccl5, Birc2, Rela, Mapk1, Tnf, Nfkbib, Birc3, Nlrp3, Tnfaip3, Hsp90b1, Erbin, Il1b, Nfkbia, Cxcl2, Ripk2, Tab2, Xiap
04622	RIG-I-like receptor signaling pathway	0	Immune system	12	Irf7, Ikbke, Traf3, Cxcl10, Rela, Tnf, Nfkbib, Tank, Trim25, Tradd, Sike1, Nfkbia
04623	Cytosolic DNA-sensing pathway	0	Immune system	11	Polr1c, Irf7, Ikbke, Ccl5, Cxcl10, Rela, Nfkbib, Ccl4, Aim2, Il1b, Nfkbia
04660	T cell receptor signaling pathway	0	Immune system	22	Vav3, Jun, Map3k8, Rela, Mapk1, Tnf, Nfkbib, Pik3cg, Fos, Lck, Pik3r1, Sos1, Pik3r5, Pdk1, Gsk3b, Cd28, Nfkbia, Dlg1, Nfatc3, Cbl, Malt1, Nfkbie
04662	B cell receptor signaling pathway	0	Immune system	16	Vav3, Jun, Rela, Mapk1, Nfkbib, Pik3cg, Fos, Pik3r1, Sos1, Pik3r5, Gsk3b, Rasgrp3, Nfkbia, Nfatc3, Malt1, Nfkbie
04722	Neurotrophin signaling pathway	0	Nervous system	20	Crk, Jun, Rela, Mapk1, Nfkbib, Pik3cg, Pik3r1, Map3k3, Sos1, Pik3r5, Pdk1, Irak2, Gsk3b, Bcl2, Atf4, Nfkbia, Ripk2, Irak4, Braf, Nfkbie
04912	GnRH signaling pathway	0	Endocrine system	11	Itpr1, Jun, Mapk1, Prkacb, Map3k3, Gnaq, Sos1, Ptk2b, Atf4, Pla2g4a, Adcy7
04920	Adipocytokine signaling pathway	0	Endocrine system	14	Rela, Tnf, Nfkbib, Acsl1, Prkag2, Camkk2, Pck2, Acsl3, Tradd, Prkab2, Adipor2, Nfkbia, Socs3, Nfkbie
05140	Leishmaniasis	0	Infectious diseases	18	Ptgs2, Jun, Tlr2, H2-Oa, Rela, Mapk1, Tnf, Nfkbib, Fos, Jak1, Marcksl1, Tlr4, Il1b, Nfkbia, Tab2, Irak4, Itga4, H2-DMa
05142	Chagas disease (American trypanosomiasis)	0	Infectious diseases	20	Jun, Ccl5, Tlr2, Rela, Ccl3, Mapk1, Tnf, Pik3cg, Fos, Pik3r1, Tgfbr2, Gnaq, Pik3r5, Tlr4, Cflar, Il1b, Nfkbia, Irak4, Ppp2ca, Fas

05160	Hepatitis C	0	Infectious diseases	27	Irf7, Ikbke, Traf3, Pias2, Rela, Mapk1, Tnf, Cdkn1a, Eif2ak2, Pik3cg, Cldn18, Pik3r1, Rnasel, Tradd, Ldlr, Jak1, Sos1, Pik3r5, Pdk1, Gsk3b, Ifnar2, Irf1, Nfkbia, Cldn1, Socs3, Ppp2ca, Braf,
05323	Rheumatoid arthritis	0	Immune diseases	17	Jun, Ccl5, Il15, Tlr2, Ltb, H2-Oa, Ccl3, Tnf, Cd86, Fos, Icam1, Cd28, Tlr4, Il1b, Atp6v1a, H2-DMa, Csf1
05416	Viral myocarditis	0	Cardiovascular diseases	13	Ccnd1, H2-Oa, Myh9, Cd86, H2-T24, Icam1, Eif4g1, Myh3, Sgcg, Cd28, Cd40, Abl2, H2-DMa

Down-regulated pathways

Entry	Pathway_names	p value	Class	Gene number	Gene
01100	Metabolic pathways	0		97	Isyna1, Polr1c, Ptgs2, Galnt3, Nans, Hsd17b7, Btd, Hal, Pank1, Eno2, St3gal1, Mthfd2, Ggps1, Trdmt1, Idh1, Scp2, Hk1, Adh7, Acsl1, Gk, C1galt1, Dnmt3a, Lipt2, Mat2a, Idi1, Fasn, Galnt11, Uroc1, Pck2, Nmnat1, Pik3c2a, Nt5c2, Cth, Pank3, B4galt7, Mgat2, Shmt2, Sgms1, Galnt2, Lss, Piga, Galnt6, Pafah1b1, Cbr3, Pdxk, Acsl3, Ppat, Mgat5, Smpd4, Gch1, Pmm2, Mthfr, Sc5d, Ugcg, Pign, Gda, Cox5b, Man1c1, Pgap1, Inpp4a, Aldh18a1, Pfkp, Gpt2, Odc1, Cds2, Alg6, Gclm, Pigl, Itpkb, Hmgcs1, Chka, Prdx6, Pold3, Mgat4a, Ext2, Smpd1, Ggt1, Pla2g4a, B4galt3, Bst1, Atp6v1a, Bcat1, B4galt6, Psat1, Asns, Lpcat1, Umps, Acly, Rev3l, Dgkd, Dctd, Alg10b, Acaa2, Gclc, Cryl1, Msmo1, Rdh10
03013	RNA transport	0	Translation	24	Nup62, Eif3a, Xpo1, Elac1, Srrm1, Nup50, Eif1, Seh1l, Nupl1, Eif4g1, Nup54, Eif5b, Eif4ebp1, Nmd3, Thoc2, Nup98, Nup153, Tgs1, Ranbp2, Paip1, Ube2i, Eif5, Tpr, Nup214
03015	mRNA surveillance pathway	0	Translation	15	Srrm1, Cpsf2, Cpsf6, Ppp2r5e, Ppp2r5c, Rnmt, Hbs1l, Gspt1, Ppp2r3a, Ppp2r3d, Papola, Nudt21, Ppp2ca, Pcf11, Smg1,
03040	Spliceosome	0	Transcription	15	Tra2b, Hspa8, Snrpd3, Srsf7, Ccdc12, Ppih, U2surp, Srsf3, Thoc2, Prpf40a, Srsf10, Slu7, Srsf1, Tra2a, Prpf19

04012	ErbB signaling pathway	0.049	Signal transduction	15	Crk, Jun, Cdkn1b, Mapk1, Cdkn1a, Pik3cg, Myc, Pik3r1, Sos1, Eif4ebp1, Pik3r5, Gsk3b, Abl2, Cbl, Braf
04020	Calcium signaling pathway	0	Signal transduction	14	Atp2a2, Itpr1, Cysltr1, Prkacb, Htr5b, Ptafr, Atp2b1, Gnaq, F2r, Itpkb, Ptk2b, Bst1, Adcy7, P2rx4
04070	Phosphatidylinositol signaling system	0	Signal transduction	12	Itpr1, Pip4k2a, Pik3cg, Pik3r1, Pik3c2a, Pik3r5, Inpp4a, Cds2, Itpkb, Pikfyve, Dgkd, Pten
04110	Cell cycle	0	Cell growth and death	33	Ccnd3, Ccne1, Wee1, Cdc6, Cdc25a, Ccnd1, Ccnh, Cdkn1b, Mcm3, Atm, Cdkn1a, Stag1, Ccne2, Chek2, Myc, Skp2, Rbl1, Rbl2, Cdc27, Anapc1, Crebbp, Gsk3b, Atr, Plk1, Ttk, Gadd45a, Gadd45b, Stag2, Chek1, Gadd45g, E2f2, Mdm2, Cdk6
04114	Oocyte meiosis	0	Cell growth and death	13	Ccne1, Itpr1, Mapk1, Prkacb, Ccne2, Cdc27, Ppp2r5e, Ppp2r5c, Anapc1, Sgol1, Plk1, Adcy7, Ppp2ca,
04120	Ubiquitin mediated proteolysis	0	Folding, sorting and degradation	27	Birc2, Pias2, Birc3, Brca1, Skp2, Ube3a, Cdc27, Herc2, Herc4, Birc6, Uba6, Itch, Anapc1, Vhl, Ddb2, Fbxw8, Trip12, Ube2f, Wwp1, Cul5, Ube2i, Cbl, Socs3, Xiap, Pml, Mdm2, Prpf19
04144	Endocytosis	0	Transport and catabolism	29	Ehd1, Stam2, Chmp1b, Eps15, Hspa8, Grk1, H2-T24, Tfrc, Agap3, Rab5c, Tgfbr2, Dab2, Itch, Ldlr, Acap2, Arf6, Agap1, Arap2, F2r, Ehd4, Arap3, Ap2b1, Wwp1, Iqsec1, Dnm1l, Rab11fip2, Cbl, Pml, Mdm2
04145	Phagosome	0	Transport and catabolism	15	Tlr2, H2-Oa, Itgav, H2-T24, Tfrc, Itga5, Rab5c, Tubb2a, Stx18, Coro1a, Tlr4, Marco, Atp6v1a, Pikfyve, H2-DMa
04270	Vascular smooth muscle contraction	0	Circulatory system	11	Itpr1, Mapk1, Prkacb, Prkch, Gnaq, Ppp1r12a, Gna13, Pla2g4a, Adcy7, Braf, Rock1
04310	Wnt signaling pathway	0	Signal transduction	21	Ccnd3, Jun, Ccnd1, Csnk2a1, Nlk, Prkacb, Myc, Ppp2r5e, Ppp2r5c, Tbl1x, Ctbp2, Crebbp, Gsk3b, Ppard, Tbl1xr1, Apc, Lrp6, Nfatc3, Csnk2a2, Ppp2ca, Rock1
04360	Axon guidance	0	Development	11	Mapk1, Rgs3, Rasa1, Sema4c, Gsk3b, Nrp1, Sema4g, Plxnc1, Nfatc3, Rnd1, Rock1

04510	Focal adhesion	0	Cellular community - eukaryotes	28	Ccnd3, Crk, Vav3, Jun, Ccnd1, Pdgfa, Birc2, Mapk1, Itgav, Birc3, Parvb, Itga6, Pik3cg, Arhgap5, Itga5, Pik3r1, Ppp1r12a, Sos1, Pik3r5, Gsk3b, Bcl2, Diaph1, Pdgfb, Xiap, Itga4, Braf, Pten, Rock1
04630	Jak-STAT signaling pathway	0	Signal transduction	26	Ccnd3, Stam2, Ccnd1, Il15, Pias2, Bcl2l1, Il4ra, Pik3cg, Socs5, Myc, Pik3r1, Jak1, Sos1, Pik3r5, Pim1, Spry2, Spred2, Crebbp, Ifnar2, Socs2, Il7r, Il10ra, Il6ra, Il6st, Cbl, Socs3
04650	Natural killer cell mediated cytotoxicity	0	Immune system	15	Vav3, Mapk1, Tnf, Pik3cg, Icam1, Lck, Pik3r1, Sos1, Pik3r5, H60a, Ifnar2, Ptk2b, Nfatc3, Braf, Fas
04666	Fc gamma R-mediated phagocytosis	0	Immune system	12	Marcks, Crk, Vav3, Mapk1, Pik3cg, Pik3r1, Arf6, Pik3r5, Marcksl1, Pla2g4a, Hck, Dnm1
04670	Leukocyte transendothelial migration	0	Immune system	14	Sipa1, Vav3, Cdh5, Rassf5, Pik3cg, Arhgap5, Icam1, Cldn18, Pik3r1, Pik3r5, Ptk2b, Cldn1, Itga4, Rock1
04810	Regulation of actin cytoskeleton	0	Cell motility	31	Rdx, Crk, Vav3, Pdgfa, Pip4k2a, Mapk1, Myh9, Itgav, Itga6, Pik3cg, Diaph3, Itga5, Pik3r1, Ppp1r12a, Gna13, Sos1, Pik3r5, Arhgef4, Abi2, F2r, Fgf1, Diaph2, Diaph1, Apc, Pdgfb, Pikfyve, Ssh2, Ssh1, Itga4, Braf, Rock1
04910	Insulin signaling pathway	0	Endocrine system	19	Crk, Mapk1, Prkacb, Hk1, Pik3cg, Fasn, Prkag2, Pik3r1, Pck2, Sos1, Eif4ebp1, Pik3r5, Prkab2, Gsk3b, Prkar2b, Socs2, Cbl, Socs3, Braf, ,
04914	Progesterone-mediated oocyte maturation	0	Endocrine system	11	Cdc25a, Mapk1, Prkacb, Pik3cg, Pik3r1, Cdc27, Anapc1, Pik3r5, Plk1, Adcy7, Braf
05016	Huntington's disease	0	Neurodegenerative diseases	14	Itpr1, Sp1, Ift57, Bbc3, Taf4, Gnaq, Rcor1, Hip1, Tbpl1, Cox5b, Crebbp, Sod1, Ap2b1, Sod2
05146	Amoebiasis	0	Infectious diseases	11	Tlr2, Rela, Tnf, Prkacb, Pik3cg, Rab5c, Pik3r1, Gnaq, Pik3r5, Tlr4, Il1b

05200	Pathways in cancer	0	Cancers	60	Hif1a, Crk, Ccne1, Msh6, Ptgs2, Jun, Ccnd1, Traf3, Pdgfa, Birc2, Pias2, Cdkn1b, Rela, Mapk1, Bcl2l1, Itgav, Birc3, Cdkn1a, Itga6, Ccne2, Rassf5, Pik3cg, Fos, Max, Traf1, Bmp2, Myc, Skp2, Pik3r1, Nfkb2, Tgfbr2, Smo, Runx1, Jak1, Stk4, Sos1, Ctbp2, Vhl, Pik3r5, Crebbp, Gsk3b, Bcl2, Hsp90b1, Ppard, Fgf1, Nfkbia, Apc, Pdgfb, Rala, E2f2, Cbl, Xiap, Pml, Tpr, Braf, Pten, Mdm2, Fas, Cdk6, Ralgds
05210	Colorectal cancer	0	Cancers	15	Msh6, Jun, Ccnd1, Mapk1, Pik3cg, Fos, Myc, Pik3r1, Tgfbr2, Pik3r5, Gsk3b, Bcl2, Apc, Braf, Ralgds
05212	Pancreatic cancer	0	Cancers	14	Ccnd1, Rela, Mapk1, Bcl2l1, Pik3cg, Pik3r1, Tgfbr2, Jak1, Pik3r5, Rala, E2f2, Braf, Cdk6, Ralgds
05213	Endometrial cancer	0	Cancers	11	Ccnd1, Mapk1, Pik3cg, Myc, Pik3r1, Sos1, Pik3r5, Gsk3b, Apc, Braf, Pten
05214	Glioma	0	Cancers	14	Ccnd1, Pdgfa, Mapk1, Cdkn1a, Pik3cg, Pik3r1, Sos1, Pik3r5, Pdgfb, E2f2, Braf, Pten, Mdm2, Cdk6
05218	Melanoma	0	Cancers	14	Ccnd1, Pdgfa, Mapk1, Cdkn1a, Pik3cg, Pik3r1, Pik3r5, Fgf1, Pdgfb, E2f2, Braf, Pten, Mdm2, Cdk6,
05220	Chronic myeloid leukemia	0	Cancers	21	Crk, Ccnd1, Cdkn1b, Rela, Mapk1, Bcl2l1, Cdkn1a, Pik3cg, Myc, Pik3r1, Tgfbr2, Runx1, Sos1, Ctbp2, Pik3r5, Nfkbia, E2f2, Cbl, Braf, Mdm2, Cdk6
05222	Small cell lung cancer	0	Cancers	26	Ccne1, Ptgs2, Ccnd1, Traf3, Birc2, Pias2, Cdkn1b, Rela, Bcl2l1, Itgav, Birc3, Itga6, Ccne2, Pik3cg, Max, Traf1, Myc, Skp2, Pik3r1, Pik3r5, Bcl2, Nfkbia, E2f2, Xiap, Pten, Cdk6
05223	Non-small cell lung cancer	0	Cancers	11	Ccnd1, Mapk1, Rassf5, Pik3cg, Pik3r1, Stk4, Sos1, Pik3r5, E2f2, Braf, Cdk6
05410	Hypertrophic cardiomyopathy (HCM)	0	Cardiovascular diseases	11	Atp2a2, Tnf, Itgav, Itga6, Itga5, Prkag2, Sgcg, Prkab2, Cacng7, Itga4, Lmna

05414	Dilated cardiomyopathy	0	Cardiovascular diseases	11	Atp2a2, Tnf, Prkacb, Itgav, Itga6, Itga5, Sgcg, Cacng7, Adcy7, Itga4, Lmna
