# Supplementary material for: Integrative Transcriptomic Profiling Identifies TNF and IL1B as Candidate Key Early-Response Genes in Macrophages Infected with Smooth Brucella Using a Comprehensive Bioinformatic Approach
Source: Biology (Basel). 2025 May 21;14(5):579. doi: 10.3390/biology14050579 (PMC12109160; doi:10.3390/biology14050579)
Supplement: Supplementary file 1 [file biology-14-00579-s001.zip › Table S2.pdf]

Table S2 Dysregulated pathways inside infected macrophages with smooth B.melitensis

At 4h interaction time-point

Up-regulated pathways

Entry	Pathway_names	p value	Class	Gene number	Gene
00100	Steroid biosynthesis	0	Lipid metabolism	10	Sqle，Nsdhl，Soat1，Hsd17b7，Cyp51，Msmo1，Lss，Sc5d，Fdft1，Soat2
00510	N-Glycan biosynthesis	0	Glycan biosynthesis and metabolism	19	Man1a，B4galt2，Dad1，Alg9，Rpn2，Alg14，Man1a2，Mogs，Stt3a，Alg5，Mgat4b，Mgat2，Mgat5，Alg11，Man1c1，Ganab，Alg6，Mgat4a，Man1b1
00590	Arachidonic acid metabolism	0	Lipid metabolism	11	Ptgs2，Pla2g5，Cbr2，Cbr3，Pla2g6，Lta4h，Ptgs1，Pla2g4a，Cyp2c54，Cbr1，Gpx1
00600	Sphingolipid metabolism	0	Lipid metabolism	16	Sgpl1，Sphk2，Sgpp1，Ugcg，Sptlc1，Degs1，Sgms1，Sgms2，Plpp3，Smpd4，Cerk，Acer3，Neu1，Gla，B4galt6，Galc
04010	MAPK signaling pathway	0.041	Signal transduction	104	Dusp6，Map2k2，Crk，Akt1，Mapkapk5，Ecsit，Rras2，Ddit3，Rps6ka2，Mknk1，Pla2g5，Relb，Srf，Mknk2，Stk3，Map3k8，Il1r2，Tnf，Map3k7，Prkacb，Ppp3cc，Tgfbr1，Pak1，Gna12，Il1a，Mapk9，Sos1，Elk1，Crkl，Rasa2，Fos，Prkx，Taok1，Map3k1，Myc，Ppm1b，Tgfbr2，Map2k3，Ppm1a，Zak，Nfkb2，Nfatc2，Casp3，Rasa1，Atf2，Mapkapk2，Map4k3，Dusp7，Nfkb1，Ppp3cb，Dusp4，Hspa8，Pla2g6，Ntf3，Mapkapk3，Rap1b，Ikbkg，Akt3，Egfr，Stk4，Map3k3，Mapk8ip3，Dusp5，Map3k2，Map4k1，Map3k5，Fgf12，Map4k4，Stmn1，Atf4，Rps6ka4，Pla2g4a，Dusp1，Grb2，Pdgfa，Il1b，Gadd45a，Fgf18，Cdc42，Gadd45b，Chp1，Pdgfb，Prkaca，Ppp5c，Chuk，Mef2c，Mapk14，Kras，Ppp3ca，Sos2，Rps6ka3，Rapgef2，Map2k5，Ikbkb，Dusp9，Mapt，Gng12，Taok3，Nlk，Braf，Cacng8，Map3k4，Fas，Arrb1

04020	Calcium signaling pathway	0	Signal transduction	35	Itpr3，Sphk2，Adcy9，P2rx7，Prkacb，Ppp3cc，Adcy7，Htr5b，Htr5a，Camk2d，Camk2g，Prkx，Atp2a2，Itpr2，Ryr1，Ptafr，Ppp3cb，Phkg2，Phkb，Itpkb，Egfr，F2r，Vdac1，Slc25a5，Grm1，Pde1c，Plcb3，Bst1，Chp1，Adora2b，Prkaca，Ppp3ca，P2rx4，Gnaq，P2rx1
04060	Cytokine-cytokine receptor interaction	0	Signaling molecules and interaction	51	Il2rg，Il18，Ghr，Tnfrsf1b，Il15，Ppbp，Cxcl16，Cxcl10，Ltb，Il1r2，Tnf，Tgfbr1，Il17ra，Ccr2，Ccl7，Il18rap，Csf2rb，Il1a，Tnfsf9，Tnfrsf13b，Ifngr2，Csf1r，Bmp2，Il4ra，Ccr5，Tgfbr2，Bmpr1a，Lepr，Il13ra1，Tnfrsf9，Acvr1b，Egfr，Osm，Ifnar2，Il7r，Il10ra，Ccl9，Pf4，Pdgfa，Il1b，Cxcl2，Il6，Pdgfb，Ccl24，Vegfb，Lifr，Acvr2a，Csf1，Fas，Il6st，Cd40
04062	Chemokine signaling pathway	0	Immune system	68	Crk，Akt1，Vav3，Tiam1，Gng2，Pik3r2，Ppbp，Cxcl16，Cxcl10，Adcy9，Prkacb，Adcy7，Pak1，Jak2，Ccr2，Ccl7，Sos1，Crkl，Stat5b，Gnb5，Pik3cg，Rock1，Csk，Pxn，Prkx，Ccr5，Pik3r1，Ncf1，Nfkb1，Gngt2，Pik3ca，Ptk2，Pik3r5，Rap1b，Ikbkg，Gnai2，Akt3，Gnai3，Grk6，Gnb4，Vav1，Fgr，Gng4，Nfkbia，Plcb3，Ccl9，Gng11，Pf4，Grb2，Grk5，Cdc42，Cxcl2，Stat1，Ccl24，Prkaca，Gnb2，Rhoa，Gsk3b，Chuk，Kras，Grk2，Sos2，Pik3cb，Pik3cd，Ikbkb，Gng12，Braf，Arrb1
04080	Neuroactive ligand-receptor interaction	0	Signaling molecules and interaction	29	Ghr，C3ar1，P2rx7，Ptger4，C5ar1，Htr5b，Htr5a，Sstr4，S1pr1，P2ry6，Calcrl，Lepr，Npy1r，Ptafr，S1pr2，Grid2，Gria4，F2r，Aplnr，Grm1，Gabrg3，Ptger2，Gabrd，Adora2b，P2rx4，Grm4，Nr3c1，S1pr3，P2rx1

04144	Endocytosis	0	Transport and catabolism	81	Itch，Ehd1，Il2rg，Stam，Stam2，Vps4b，Prkci，Sh3glb1，Rab4a，Psd3，Arf6，Eps15，Arap3，Smad2，Tgfbr1，H2-M3，Tfrc，Csf1r，Agap3，Ldlrap1，Vps37b，Vps36，Ccr5，Tgfbr2，Asap1，Chmp2a，Pip5k1a，Rabep1，Pdcd6ip，Sh3glb2，Igf1r，Rab11fip5，Mdm2，H2-T3，H2-K1，Arap1，Mvb12b，Chmp2b，Dab2，Hspa8，Sh3kbp1，Rab31，Smap1，Stambp，Dnm2，Ap2a2，Sh3gl3，Snf8，Clta，Agap1，Psd，Egfr，Mvb12a，Pld1，F2r，Grk6，Vps37c，Rab11fip3，Eea1，Cltc，Rab11b，Zfyve16，Pard6a，Grk5，Cdc42，Ehd4，Ldlr，Cbl，Smad3，Rhoa，Grk2，Arap2，Ap2b1，Wwp1，Iqsec1，H2-D1，Dnm1l，Cblb，Rab11fip2，Pml，Arrb1
04145	Phagosome	0	Transport and catabolism	45	Actg1，Atp6v1h，Sec61a1，Lamp2，M6pr，Thbs3，Dync1h1，Tuba4a，Stx7，Tlr2，Atp6v1e1，Clec7a，Vamp3，Itgav，H2-M3，Atp6v1a，Canx，Tfrc，Cybb，Pikfyve，Atp6v1g1，Itga5，C3，Atp6v0a2，Msr1，Ncf1，Tubb2a，H2-T3，H2-K1，Stx18，Coro1a，Fcgr2b，Eea1，Atp6v1d，H2-Ob，Tlr4，Fcgr3，Stx12，Cd36，H2-D1，Itgb1，Tubb2b，Tap2，Itgb5，Atp6v0a1
04350	TGF-beta signaling pathway	0	Signal transduction	25	Smad1，Thbs3，Id3，Tnf，Smad2，Tgfbr1，Rps6kb2，Smad4，Id2，Id4，Rock1，Bmp2，Rbl1，Myc，Tgfbr2，Bmpr1a，Id1，Rbl2，Lefty2，Zfyve16，Smad3，Rhoa，Acvr2a，Rps6kb1，E2f5
04380	Osteoclast differentiation	0	Development	52	Junb，Akt1，Sirpa，Fyn，Relb，Pik3r2，Spi1，Tnf，Gab2，Map3k7，Ppp3cc，Tgfbr1，Il1a，Trem2，Mapk9，Pik3cg，Cybb，Fos，Ifngr2，Csf1r，Pirb，Lck，Tgfbr2，Pik3r1，Ncf1，Nfkb2，Nfkb1，Ppp3cb，Nfatc1，Pik3ca，Cyld，Jak1，Pik3r5，Fcgr2b，Ikbkg，Akt3，Fosl2，Ifnar2，Nfkbia，Fcgr3，Grb2，Il1b，Stat1，Chuk，Mapk14，Ppp3ca，Pik3cb，Pik3cd，Ikbkb，Mitf，Socs3，Csf1

04510	Focal adhesion	0	Cellular community - eukaryotes	67	Actg1，Ccnd3，Vcl，Crk，Capn2，Pdpk1，Thbs3，Akt1，Parvg，Vav3，Ccnd1，Fyn，Pik3r2，Pak1，Itgav，Lamc2，Birc3，Mapk9，Sos1，Elk1，Crkl，Pik3cg，Col1a2，Itga5，Rock1，Pxn，Col4a5，Pik3r1，Ppp1cb，Igf1r，Rapgef1，Tln2，Pik3ca，Ctnnb1，Ptk2，Pik3r5，Rap1b，Akt3，Egfr，Pak3，Parvb，Vav1，Dock1，Pak6，Tln1，Grb2，Pdgfa，Cdc42，Pdgfb，Col2a1，Rhoa，Pten，Arhgap5，Gsk3b，Vasp，Vegfb，Igf1，Sos2，Itgb1，Pik3cb，Ppp1r12a，Pik3cd，Ccnd2，Xiap，Itgb5，Braf，Bcl2
04512	ECM-receptor interaction	0	Signaling molecules and interaction	16	Thbs3，Itgav，Lamc2，Col1a2，Itga5，Sv2a，Col4a5，Cd47，Sdc3，Sdc4，Hmmr，Col2a1，Cd36，Cd44，Itgb1，Itgb5
04514	Cell adhesion molecules (CAMs)	0	Signaling molecules and interaction	26	Glg1，Cd28，Icosl，Cd86，Itgav，H2-M3，Ncam2，Cldn13，Icam1，F11r，Itgal，Cldn18，H2-T3，H2-K1，Mpzl1，Nrcam，Sdc3，Cntnap2，Ptprc，H2-Ob，Neo1，Sdc4，Cd80，H2-D1，Itgb1，Cd40
04610	Complement and coagulation cascades	0	Immune system	14	C1qa，Serpinc1，Serpine1，C3ar1，Plau，C5ar1，C3，Cr1l，F2r，C1qb，Serpina1c，C1qc，Cfh，Plaur
04612	Antigen processing and presentation	0	Immune system	19	Hspa4，Hsp90ab1，Psme3，Rfxank，Tnf，H2-M3，Canx，Pdia3，H2-T3，H2-K1，Hspa8，H2-Ob，Ctsb，Lgmn，Tapbp，H2-D1，Nfya，Tap2，Rfxap
04620	Toll-like receptor signaling pathway	0	Immune system	42	Map2k2，Akt1，Ikbke，Pik3r2，Traf3，Cxcl10，Tlr2，Map3k8，Tnf，Map3k7，Cd86，Irak4，Mapk9，Tlr7，Pik3cg，Tlr3，Tollip，Fos，Map2k3，Pik3r1，Nfkb1，Pik3ca，Pik3r5，Ikbkg，Akt3，Ripk1，Ifnar2，Tlr4，Nfkbia，Irak1，Il1b，Ly96，Stat1，Il6，Chuk，Mapk14，Cd80，Pik3cb，Pik3cd，Ikbkb，Irf5，Cd40

04621	NOD-like receptor signaling pathway	0	Immune system	26	Hsp90ab1，Pycard，Il18，Tnf，Map3k7，Birc3，Naip5，Mapk9，Pstpip1，Nlrp3，Nfkb1，Tab3，Tnfaip3，Ikbkg，Hsp90b1，Erbin，Nfkbia，Casp1，Il1b，Cxcl2，Il6，Chuk，Mapk14，Ikbkb，Xiap，Naip2
04622	RIG-I-like receptor signaling pathway	0	Immune system	24	Ikbke，Traf3，Cxcl10，Tnf，Map3k7，Dhx58，Tank，Mapk9，Ddx3x，Map3k1，Ifih1，Trim25，Nfkb1，Cyld，Ikbkg，Sike1，Mavs，Ripk1，Nfkbia，Chuk，Atg12，Mapk14，Tradd，Ikbkb
04623	Cytosolic DNA-sensing pathway	0	Immune system	23	Pycard，Ikbke，Il18，Cxcl10，Polr3k，Adar，Aim2，Nfkb1，Ikbkg，Polr3f，Mavs，Ripk1，Polr3b，Nfkbia，Polr3g，Casp1，Il1b，Polr1c，Il6，Polr3gl，Chuk，Polr3c，Ikbkb
04630	Jak-STAT signaling pathway	0	Signal transduction	50	Ccnd3，Il2rg，Akt1，Stam，Stam2，Ccnd1，Ghr，Il15，Pik3r2，Pias2，Bcl2l1，Jak2，Csf2rb，Sos1，Stat5b，Pik3cg，Socs5，Ifngr2，Il4ra，Myc，Pik3r1，Lepr，Il13ra1，Spred1，Pik3ca，Jak1，Socs7，Pik3r5，Spred3，Akt3，Spred2，Osm，Ifnar2，Il7r，Il10ra，Grb2，Stat1，Il6，Cbl，Sos2，Pik3cb，Pik3cd，Lifr，Cblb，Socs4，Pias4，Pias1，Socs3，Ccnd2，Il6st
04640	Hematopoietic cell lineage	0	Immune system	16	Cd9，Cd5，Il1r2，Tnf，Anpep，Il1a，Tfrc，Itga5，Csf1r，Il4ra，Il7r，Il1b，Il6，Cd36，Cd44，Csf1
04650	Natural killer cell mediated cytotoxicity	0	Immune system	41	Map2k2，Vav3，Fyn，Pik3r2，Tnf，Nfatc3，Ppp3cc，Pak1，Sos1，Pik3cg，Ifngr2，Icam1，Itgal，Lck，Pik3r1，Nfatc2，Casp3，Klra7，H2-K1，Ppp3cb，Nfatc1，Araf，Pik3ca，Pik3r5，Nfat5，Ifnar2，Vav1，Bid，Fcgr3，Grb2，Cd244，Chp1，Kras，Ppp3ca，Sos2，H2-D1，Pik3cb，Pik3cd，Braf，H60a，Fas

04660	T cell receptor signaling pathway	0	Immune system	55	Dlg1，Map2k2，Akt1，Vav3，Fyn，Cd28，Pik3r2，Bcl10，Map3k8，Tnf，Nfatc3，Map3k7，Ppp3cc，Pak1，Nck1，Mapk9，Sos1，Cdk4，Pik3cg，Fos，Pdk1，Lck，Pik3r1，Nfatc2，Nfkb1，Ppp3cb，Nfatc1，Pik3ca，Nck2，Pik3r5，Ikbkg，Akt3，Pak3，Nfat5，Ptprc，Vav1，Pak6，Nfkbia，Grb2，Cdc42，Chp1，Cbl，Rhoa，Gsk3b，Chuk，Mapk14，Kras，Ppp3ca，Sos2，Pik3cb，Pik3cd，Ikbkb，Cblb，Malt1，Nfkbie
04662	B cell receptor signaling pathway	0	Immune system	38	Map2k2，Akt1，Vav3，Pik3r2，Bcl10，Nfatc3，Ppp3cc，Pik3ap1，Sos1，Pik3cg，Fos，Inpp5d，Pirb，Pik3r1，Nfatc2，Nfkb1，Ppp3cb，Nfatc1，Pik3ca，Pik3r5，Fcgr2b，Ikbkg，Akt3，Nfat5，Vav1，Nfkbia，Grb2，Chp1，Gsk3b，Chuk，Kras，Ppp3ca，Sos2，Pik3cb，Pik3cd，Ikbkb，Malt1，Nfkbie
04666	Fc gamma R-mediated phagocytosis	0	Immune system	40	Marcks，Crk，Akt1，Vav3，Sphk2，Cfl2，Pik3r2，Limk2，Arf6，Gab2，Pak1，Crkl，Rps6kb2，Pik3cg，Inpp5d，Pik3r1，Asap1，Ncf1，Pip5k1a，Pik3ca，Plpp3，Pla2g6，Dnm2，Pik3r5，Marcksl1，Fcgr2b，Akt3，Arpc5l，Pld1，Gsn，Ptprc，Vav1，Pla2g4a，Cdc42，Myo10，Vasp，Dnm1l，Pik3cb，Pik3cd，Rps6kb1
04670	Leukocyte transendothelial migration	0	Immune system	36	Actg1，Vcl，Sipa1，Vav3，Pik3r2，Pik3cg，Cldn13，Cybb，Rock1，Pxn，Icam1，F11r，Itgal，Cldn18，Pik3r1，Ncf1，Pik3ca，Ctnnb1，Ptk2，Pik3r5，Rap1b，Gnai2，Afdn，Gnai3，Vav1，Mmp9，Cdc42，Msn，Rhoa，Ezr，Arhgap5，Vasp，Mapk14，Itgb1，Pik3cb，Pik3cd
04740	Olfactory transduction	0	Sensory system	11	Prkacb，Olfr690，Olfr66，Camk2d，Camk2g，Prkx，Olfr78，Grk3，Pde1c，Prkaca，Olfr749
04916	Melanogenesis	0.038	Endocrine system	24	Map2k2，Fzd2，Wnt6，Adcy9，Prkacb，Adcy7，Camk2d，Camk2g，Prkx，Tcf7l2，Ctnnb1，Gnai2，Gnai3，Fzd3，Wnt8b，Dvl2，Plcb3，Prkaca，Gsk3b，Kras，Mitf，Fzd5，Gnaq，Fzd6

04920	Adipocytokine signaling pathway	0	Endocrine system	30	Akt1，Rxrb，Mtor，Tnfrsf1b，Stk11，Tnf，Jak2，Mapk9，Acsl1，Irs1，Prkag2，Prkab1，Camkk2，Pck2，Lepr，Slc2a1，Nfkb1，Cpt1c，Ikbkg，Prkab2，Akt3，Nfkbia，Cd36，Adipor1，Chuk，Acsl4，Tradd，Ikbkb，Socs3，Nfkbie
04930	Type II diabetes mellitus	0	Endocrine and metabolic diseases	18	Mtor，Pik3r2，Tnf，Mapk9，Hk2，Pik3cg，Irs1，Pik3r1，Pik3ca，Insr，Pik3r5，Hk3，Pik3cb，Pik3cd，Ikbkb，Socs4，Abcc8，Socs3
04940	Type I diabetes mellitus	0	Endocrine and metabolic diseases	12	Cd28，Tnf，Cd86，H2-M3，Il1a，H2-T3，H2-K1，H2-Ob，Il1b，Cd80，H2-D1，Fas
04974	Protein digestion and absorption	0	Digestive system	10	Slc1a5，Fxyd2，Kcnn4，Col1a2，Col4a5，Slc36a1，Slc16a10，Slc7a7，Kcnq1，Col2a1
04976	Bile secretion	0	Digestive system	14	Slc4a2，Abcb1b，Fxyd2，Adcy9，Prkacb，Adcy7，Aqp9，Prkx，Slc2a1，Hmgcr，Nceh1，Car2，Ldlr，Prkaca
05014	Amyotrophic lateral sclerosis (ALS)	0	Neurodegenerative diseases	22	Bax，Tnfrsf1b，Tnf，Ppp3cc，Bcl2l1，Derl1，Map2k3，Tomm40，Casp3，Ppp3cb，Tomm40l，Casp9，Map3k5，Bid，Ccs，Casp1，Chp1，Apaf1，Mapk14，Ppp3ca，Bcl2，Gpx1
05100	Bacterial invasion of epithelial cells	0	Infectious diseases	28	Actg1，Vcl，Crk，Pik3r2，Crkl，Pik3cg，Itga5，Pxn，Pik3r1，Arhgap10，Pik3ca，Ctnnb1，Ptk2，Dnm2，Clta，Pik3r5，Arpc5l，Cltc，Dock1，Cdc42，Cbl，Rhoa，Itgb1，Dnm1l，Pik3cb，Pik3cd，Cblb，Mad2l2
05140	Leishmaniasis	0	Infectious diseases	24	Ptgs2，Tlr2，Tnf，Map3k7，Jak2，Il1a，Irak4，Elk1，Fos，Ifngr2，C3，Ncf1，Nfkb1，Jak1，Marcksl1，H2-Ob，Tlr4，Nfkbia，Fcgr3，Irak1，Il1b，Stat1，Mapk14，Itgb1

05142	Chagas disease (American trypanosomiasis)	0	Infectious diseases	40	Akt1，C1qa，Pik3r2，Tlr2，Serpine1，Tnf，Smad2，Tgfbr1，Irak4，Mapk9，Pik3cg，Fos，Ifngr2，C3，Tgfbr2，Pik3r1，Cflar，Nfkb1，Pik3ca，Pik3r5，Ikbkg，Gnai2，Akt3，Gnai3，C1qb，Tlr4，Nfkbia，Plcb3，Irak1，Il1b，C1qc，Il6，Smad3，Chuk，Mapk14，Pik3cb，Pik3cd，Ikbkb，Gnaq，Fas
05144	Malaria	0	Infectious diseases	15	Thbs3，Il18，Tlr2，Tnf，Gypc，Icam1，Itgal，Sdc3，Tlr4，Lrp1，Sdc4，Il1b，Il6，Cd36，Cd40
05145	Toxoplasmosis	0	Infectious diseases	48	Akt1，Pla2g5，Pik3r2，Tlr2，Tnf，Map3k7，Bcl2l1，Jak2，Lamc2，Birc3，Irak4，Mapk9，Pik3cg，Ifngr2，Pdk1，Ccr5，Map2k3，Pik3r1，Casp3，Nfkb1，Pik3ca，Hspa8，Pla2g6，Jak1，Pik3r5，Ikbkg，Gnai2，Akt3，Gnai3，Casp9，H2-Ob，Tlr4，Nfkbia，Pla2g4a，Irak1，Il10ra，Ly96，Stat1，Ldlr，Chuk，Mapk14，Itgb1，Pik3cb，Pik3cd，Ikbkb，Xiap，Bcl2，Cd40
05146	Amoebiasis	0	Infectious diseases	29	Vcl，Pik3r2，Tlr2，Il1r2，Tnf，Prkacb，Lamc2，Serpinb9，Pik3cg，Serpinb6b，Col1a2，Prkx，Col4a5，Pik3r1，Casp3，Nfkb1，Pik3ca，Ptk2，Pik3r5，Tlr4，Plcb3，Il1b，Serpinb6a，Il6，Prkaca，Col2a1，Pik3cb，Pik3cd，Gnaq
05150	Staphylococcus aureus infection	0	Infectious diseases	14	C1qa，C3ar1，C5ar1，C3，Icam1，Itgal，Ptafr，Fcgr2b，C1qb，H2-Ob，Fcgr3，C1qc，Cfh，Krt10
05160	Hepatitis C	0	Infectious diseases	46	Akt1，Ikbke，Psme3，Pik3r2，Traf3，Pias2，Tnf，Eif2ak4，Mapk9，Sos1，Pik3cg，Tlr3，Cldn13，Pdk1，Cldn18，Pik3r1，Rnasel，Nfkb1，Araf，Pik3ca，Jak1，Pik3r5，Ikbkg，Akt3，Egfr，Mavs，Ripk1，Ifnar2，Eif2ak2，Nfkbia，Grb2，Stat1，Ldlr，Gsk3b，Chuk，Mapk14，Kras，Sos2，Tradd，Pik3cb，Pik3cd，Ikbkb，Pias4，Pias1，Socs3，Braf

05211	Renal cell carcinoma	0.041	Cancers	35	Map2k2，Crk，Akt1，Pik3r2，Pak1，Vhl，Sos1，Crkl，Pik3cg，Egln1，Fh1，Pik3r1，Slc2a1，Rapgef1，Araf，Pik3ca，Pik3r5，Rap1b，Akt3，Tceb2，Arnt，Pak3，Pak6，Hif1a，Grb2，Pdgfa，Cdc42，Pdgfb，Vegfb，Kras，Sos2，Tceb1，Pik3cb，Pik3cd，Braf
05216	Thyroid cancer	0	Cancers	11	Map2k2，Rxrb，Ccnd1，Myc，Tpr，Tpm3，Tcf7l2，Ctnnb1，Ncoa4，Kras，Braf
05222	Small cell lung cancer	0	Cancers	45	Ccne1，Akt1，Rxrb，Ptgs2，Ccnd1，Rb1，E2f1，Pik3r2，Traf3，Pias2，Bcl2l1，Itgav，Lamc2，Birc3，Cdk4，Ccne2，Pik3cg，Traf1，Myc，Col4a5，Pik3r1，Nfkb1，Pik3ca，Ptk2，Cdkn1b，E2f3，Pik3r5，Ikbkg，Akt3，Skp2，Casp9，Nfkbia，Apaf1，Pten，Chuk，Itgb1，Pik3cb，Pik3cd，Ikbkb，Rarb，Pias4，Pias1，E2f2，Xiap，Bcl2
05320	Autoimmune thyroid disease	0	Immune diseases	10	Cd28，Cd86，H2-M3，H2-T3，H2-K1，H2-Ob，Cd80，H2-D1，Fas，Cd40
05322	Systemic lupus erythematosus	0	Immune diseases	23	C1qa，Cd28，Snrpb，Tnf，Cd86，Snrpd3，C3，H2afy，H2afj，Hist2h2bb，H3f3b，Fcgr2b，Hist3h2a，Trove2，C1qb，H2afz，H2-Ob，Hist2h2be，Fcgr3，C1qc，Cd80，Ssb，Cd40
05323	Rheumatoid arthritis	0	Immune diseases	25	Atp6v1h，Cd28，Il18，Il15，Tlr2，Ltb，Tnf，Atp6v1e1，Cd86，Il1a，Atp6v1a，Fos，Atp6v1g1，Icam1，Atp6v0a2，Itgal，Atp6v1d，H2-Ob，Tlr4，Il1b，Il6，Vegfb，Cd80，Csf1，Atp6v0a1
05330	Allograft rejection	0	Immune diseases	11	Cd28，Tnf，Cd86，H2-M3，H2-T3，H2-K1，H2-Ob，Cd80，H2-D1，Fas，Cd40
05332	Graft-versus-host disease	0	Immune diseases	14	Cd28，Tnf，Cd86，H2-M3，Il1a，Klra7，H2-T3，H2-K1，H2-Ob，Il1b，Il6，Cd80，H2-D1，Fas

05410	Hypertrophic cardiomyopathy (HCM)	0	Cardiovascular diseases	19	Actg1，Tnf，Tpm2，Itgav，Lmna，Tpm1，Itga5，Prkag2，Prkab1，Tnnt2，Atp2a2，Tpm3，Prkab2，Sgcb，Il6，Igf1，Itgb1，Itgb5，Cacng8
05412	Arrhythmogenic right ventricular cardiomyopathy (ARVC)	0	Cardiovascular diseases	13	Actg1，Itgav，Lmna，Itga5，Atp2a2，Tcf7l2，Ctnnb1，Sgcb，Gja1，Pkp2，Itgb1，Itgb5，Cacng8
05414	Dilated cardiomyopathy	0	Cardiovascular diseases	20	Actg1，Tnf，Tpm2，Adcy9，Prkacb，Adcy7，Itgav，Lmna，Tpm1，Itga5，Prkx，Tnnt2，Atp2a2，Tpm3，Sgcb，Prkaca，Igf1，Itgb1，Itgb5，Cacng8
05416	Viral myocarditis	0	Cardiovascular diseases	25	Actg1，Ccnd1，Fyn，Cd28，Myh9，Cd86，H2-M3，Icam1，Itgal，Casp3，Eif4g3，Eif4g1，H2-T3，H2-K1，Sgcb，Casp9，H2-Ob，Abl1，Bid，Myh11，Cd80，H2-D1，Myh10，Abl2，Cd40

Down-regulated pathways

**Entry**	**Pathway_names**	**p value**	**Class**	**Gene number**	**Gene**
00010	Glycolysis / Gluconeogenesis	0	Carbohydrate metabolism	14	Eno2，Adh7，Hk2，Pck2，Dlat，Pfkp，Aldh2，Aldh9a1，Aldoa，Hk3，Pdhb，Adpgk，Aldh1b1，Pgm1
00020	Citrate cycle (TCA cycle)	0	Carbohydrate metabolism	18	Suclg1，Mdh2，Sdhb，Idh3b，Idh3a，Cs，Dlst，Fh1，Acly，Pck2，Dlat，Suclg2，Aco2，Sdhd，Pdhb，Sdhc，Idh2，Ogdh
00030	Pentose phosphate pathway	0	Carbohydrate metabolism	10	Prps1，Tktl1，Dera，Rbks，Pfkp，Aldoa，Tkt，H6pd，Prps1l1，Pgm1
00071	Fatty acid degradation	0	Lipid metabolism	19	Acadm，Cpt2，Eci1，Adh7，Acsl1，Eci2，Aldh2，Cpt1c，Aldh9a1，Hadhb，Gcdh，Acadl，Aldh1b1，Acat1，Acsl4，Echs1，Acaa2，Acadsb，Hadh

00190	Oxidative phosphorylation	0	Energy metabolism	48	Atp6v1h，Ndufv1，Atp5g1，Atp5c1，Ndufc1，Uqcrb，Ndufs5，Cyc1，Ppa1，Ndufb5，Ndufa5，Ndufa2，Sdhb，Ndufa12，Atp6v1e1，Atp6v1a，Atp6v1g1，Ndufa4，Ndufs7，Ppa2，Atp6v0a2，Ndufv3，Ndufab1，Ndufb10，Uqcrc1，Cox11，Cox10，Ndufs6，Atp5k，Ndufb6，Cox17，Atp5j2，Uqcrc2，Uqcrq，Atp5o，Sdhd，Ndufs4，Atp6v1d，Ndufb8，Uqcr11，Sdhc，Ndufa10，Ndufs2，Cox15，Ndufv2，Cox5b，Ndufa7，Atp6v0a1
00230	Purine metabolism	0	Nucleotide metabolism	58	Prps1，Gart，Polr2c，Guk1，Nme4，Pde6d，Nt5c，Entpd5，Ntpcr，Ada，Prim2，Nme7，Pde8a，Nudt2，Pola1，Adcy9，Adcy7，Pde4b，Ampd3，Polr3k，Pole4，Nme1，Nt5c2，Nudt9，Polr2i，Dck，Pde3b，Rrm2b，Ampd1，Paics，Pde8b，Adk，Polr3f，Pde10a，Polr3b，Pold3，Pde1c，Rrm1，Rrm2，Pold2，Gmpr，Pole，Nme2，Prim1，Polr3g，Polr1c，Pde7a，Entpd1，Adprm，Polr3gl，Pnpt1，Atic，Prps1l1，Polr3c，Pgm1，Pfas，Pde5a，Pold1
00240	Pyrimidine metabolism	0	Nucleotide metabolism	44	Polr2c，Nme4，Nt5c，Entpd5，Dhodh，Prim2，Nme7，Nudt2，Dut，Pola1，Polr3k，Cmpk1，Pole4，Ak3，Nme1，Txnrd1，Uckl1，Nt5c2，Tk2，Dpyd，Polr2i，Dck，Rrm2b，Tyms，Polr3f，Uck2，Polr3b，Pold3，Rrm1，Rrm2，Pold2，Pole，Nme2，Prim1，Txnrd2，Dtymk，Polr3g，Polr1c，Entpd1，Polr3gl，Pnpt1，Cad，Polr3c，Pold1
00250	Alanine, aspartate and glutamate metabolism	0	Amino acid metabolism	10	Got2，Nit2，Gfpt1，Asns，Gls2，Asl，Acy3，Got1，Cad，Gpt2
00270	Cysteine and methionine metabolism	0	Amino acid metabolism	17	Amd1，Ahcy，Mri1，Got2，Apip，Trdmt1，Dnmt3l，Ahcyl1，Cth，Dnmt1，Enoph1，Mat2a，Mtr，Mat2b，Adi1，Got1，Mtap

00280	Valine, leucine and isoleucine degradation	0	Amino acid metabolism	25	Acadm，Mccc1，Acad8，Auh，Bckdhb，Hmgcs1，Aldh2，Aldh9a1，Oxct1，Hadhb，Hsd17b10，Mcee，Pcca，Aldh6a1，Mut，Dbt，Bcat1，Aldh1b1，Acat1，Hibch，Echs1，Mccc2，Acaa2，Acadsb，Hadh
00310	Lysine degradation	0	Amino acid metabolism	19	Plod1，Setdb1，Dlst，Suv39h1，Whsc1，Setmar，Aldh2，Kmt5b，Setd7，Aldh9a1，Whsc1l1，Gcdh，Aldh1b1，Acat1，Ogdh，Echs1，Hadh，Kmt5a，Ehmt2
00330	Arginine and proline metabolism	0	Amino acid metabolism	17	Pycrl，Amd1，Got2，Acy1，Sat1，Pycr1，P4ha1，Lcmt2，Aldh2，Gls2，Aldh9a1，Aldh18a1，Odc1，Asl，Got1，Aldh1b1，Ckb
00380	Tryptophan metabolism	0	Amino acid metabolism	11	Wars2，Wars，Aldh2，Aldh9a1，Gcdh，Aldh1b1，Acat1，Ogdh，Echs1，Hadh，Kyat3
00480	Glutathione metabolism	0	Metabolism of other amino acids	15	Gstm1，Gstt2，Anpep，Gclc，Rrm2b，Ggct，Odc1，Gclm，Rrm1，Rrm2，Gss，Gstp1，Idh2，Gsto1，Gpx1
00520	Amino sugar and nucleotide sugar metabolism	0	Carbohydrate metabolism	19	Ugdh，Galk1，Nans，Nagk，Hk2，Gnpnat1，Cyb5r1，Pmm1，Cyb5r3，Pgm3，Gfpt1，Pmm2，Amdhd2，Uap1l1，Uap1，Fuk，Hk3，Uxs1，Pgm1
00561	Glycerolipid metabolism	0	Lipid metabolism	17	Dgat1，Dgkg，Gk，Gpat4，Dgkz，Plpp3，Lpl，Agk，Dgkd，Lclat1，Aldh2，Agpat4，Aldh9a1，Akr1b8，Gla，Aldh1b1，Agpat9
00562	Inositol phosphate metabolism	0	Carbohydrate metabolism	25	Isyna1，Pip4k2c，Pi4k2b，Pik3cg，Pikfyve，Minpp1，Pik3c2a，Pip5k1a，Synj2，Pik3ca，Pi4k2a，Inpp5a，Pi4ka，Pip4k2b，Itpkb，Ipmk，Pi4kb，Inpp1，Aldh6a1，Plcb3，Pten，Inpp5b，Pik3cb，Pik3cd，Impa2

00564	Glycerophospholipid metabolism	0	Lipid metabolism	29	Taz，Gpd2，Pla2g5，Cds2，Dgkg，Lpcat1，Pcyt2，Gpat4，Pla2g15，Lpgat1，Pcyt1a，Dgkz，Cds1，Plpp3，Crls1，Pla2g6，Ept1，Dgkd，Ptdss2，Lclat1，Agpat4，Pld1，Gpd1l，Pla2g4a，Adprm，Lypla1，Etnk1，Agpat9，Chpt1
00565	Ether lipid metabolism	0	Lipid metabolism	12	Pafah1b3，Pafah1b1，Pla2g5，Lpcat1，Plpp3，Pla2g6，Ept1，Pld1，Pafah2，Pla2g4a，Pafah1b2，Chpt1
00620	Pyruvate metabolism	0	Carbohydrate metabolism	14	Mdh2，Hagh，Pck2，Dlat，Me2，Acaca，Haghl，Aldh2，Aldh9a1，Pdhb，Akr1b8，Acyp1，Aldh1b1，Acat1
00640	Propanoate metabolism	0	Carbohydrate metabolism	14	Suclg1，Acadm，Suclg2，Acaca，Aldh2，Aldh9a1，Mcee，Pcca，Aldh6a1，Mut，Aldh1b1，Acat1，Hibch，Echs1
00670	One carbon pool by folate	0	Metabolism of cofactors and vitamins	12	Mthfd1，Gart，Dhfr，Mthfd2，Aldh1l1，Shmt1，Mthfd2l，Tyms，Mtr，Atic，Shmt2，Mthfd1l
00860	Porphyrin and chlorophyll metabolism	0	Metabolism of cofactors and vitamins	10	Fech，Hccs，Cpox，Alad，Cox10，Gusb，Hmox1，Blvrb，Cox15，Eprs
00970	Aminoacyl-tRNA biosynthesis	0	Translation	23	Kars，Hars，Gars，Dars，Iars，Iars2，Wars2，Tars2，Dars2，Nars2，Wars，Farsb，Lars，Vars，Aars，Sars，Eprs，Cars，Nars，Mars，Qars，Tars，Yars

01100	Metabolic pathways	0		378	Atp6v1h，Suclg1，Sgpl1，Mthfd1，Pigq，Ndufv1，Isyna1，Acadm，Sqle，Atp5g1，Prps1，Atp5c1，Nsdhl，Rfk，Nadk，Gart，Ndufc1，Ugdh，Uqcrb，Polr2c，Guk1，Pycrl，Pafah1b3，Mdh2，Ndufs5，Cyc1，C1galt1c1，Coq7，Cryl1，Nme4，Cyp3a11，Amd1，Pafah1b1，Mecr，Ndufb5，Man1a，Ahcy，Mri1，Galk1，Mccc1，Nt5c，Ptgs2，Ndufa5，Mvd，Ndufa2，Sphk2，Dhodh，Galnt3，Cyp27a1，Scly，Naglu，Got2，Nans，Ntpcr，Pla2g5，Hsd17b7，Ada，Btd，Sdhb，Prim2，B4galt2，Nme7，Apip，Dgat1，Hsd17b2，Cbr2，Dad1，B4galnt1，Fech，Comt，Cds2，Eno2，Qprt，Alg9，Idh3b，Rpn2，St3gal1，Alg14，Dhfr，Acy1，Mthfd2，Acad8，Dut，Pon3，Dhrs4，Pola1，Gaa，Ggps1，Trdmt1，Dgkg，Scp2，Atp6v1e1，Tktl1，Lpcat1，Pi4k2b，Pcyt2，Gch1，Sat1，Gbe1，Auh，B3gnt2，Man1a2，Adh7，Ugcg，Anpep，Ppt1，Mogs，Cpox，Idh3a，Atp6v1a，Acsl1，Cyp51，Extl2，Ampd3，Cs，Hk2，Sptlc1，Gk，Polr3k，C1galt1，Gpat4，Sec1，Stt3a，Cmpk1，Msmo1，Atp6v1g1，Degs1，Pole4，Fdps，Coasy，Dlst，Ndufa4，Nme1，Alg5，Pmm1，Gclc，Ndufs7，Ppcdc，Csgalnact2，Pcyt1a，Trit1，Atp6v0a2，Pycr1，Ndufv3，Rev3l，Uckl1，Mgat4b，Fh1，Alad，Uroc1，Dnmt3l，Shmt1，Acly，Ahcyl1，Pck2，Pik3c2a，Nt5c2，Pip5k1a，Tk2，Cth，Pank3，Dlat，B4galt7，Mgat2，P4ha1，Sgms1，Xylb，Dgkz，Coq6，Galnt7，Lss，Bckdhb，Hmgcr，Galnt6，Suclg2，Acaca，Synj2，Cbr3，Pdxk，Dpyd，Ndufab1，Ndufb10，Glce，B3galt5，Polr2i，Mgat5，Sgms2，Cds1，Gfpt1，Tha1，Uqcrc1，Dck，Cox11，Cox10，Plpp3，Smpd4，Mthfd2l，Crls1，Gusb，Pfkp，Pank1，Agl，Pla2g6，Pmm2，Agk，Ept1，Ces5a，Hmgcs1，Pi4k2a，Lcmt2，Dgkd，Ndufs6，Inpp5a，Rrm2b，Hpse，Asns，Atp5k，Ndufb6，Alg11，Chsy1，Cox17
02010	ABC transporters	0	Membrane transport	13	Abcb1b，Abcd4，Abcc1，Abcb6，Abcb7，Abcb9，Abcc9，Abcd2，Abca1，Abca6，Tap2，Abcg1，Abcc8

03008	Ribosome biogenesis in eukaryotes	0	Translation	31	Tbl3，Emg1，Nhp2，Fbl，Csnk2b，Nxf1，Pop5，Csnk2a1，Xrn2，Gtpbp4，Tcof1，Cirh1a，Rpp40，Drosha，Eif6，Bms1，Utp14a，Riok2，Gnl3l，Dkc1，Pop4，Riok1，Nop58，Rexo2，Rbm28，Heatr1，Fcf1，Nvl，Csnk2a2，Nop56，Nxt2
03013	RNA transport	0	Translation	66	Eif3c，Nup62，Magoh，Eif3i，Eif3a，Nxf1，Tacc3，Nup210，Pop5，Rbm8a，Nup160，Elac1，Upf1，Eif1ax，Strap，Acin1，Eif2s3x，Tgs1，Nup50，Pnn，Seh1l，Nup93，Rpp40，Trnt1，Eif4b，Eif4g3，Tpr，Eif4g1，Nup88，Elac2，Xpot，Nupl1，Eif4a1，Nup43，Ncbp1，Ndc1，Eif2b5，Nup214，Kpnb1，Eif5b，Upf3b，Eif4ebp1，Eif4e2，Gemin5，Nup85，Sumo1，Nup54，Nup188，Ranbp2，Ddx39b，Pop4，Eif2s2，Eif2b4，Eif4e，Eif4a2，Magohb，Gemin2，Pabpc4，Aaas，Gemin8，Senp2，Paip1，Thoc5，Eif5，Nup205，Nxt2
03015	mRNA surveillance pathway	0	Translation	33	Magoh，Nxf1，Cpsf1，Rbm8a，Dazap1，Upf1，Etf1，Cpsf2，Acin1，Cpsf3，Pnn，Papola，Gspt1，Ppp2r5b，Cpsf6，Ppp2r5e，Smg1，Sympk，Ncbp1，Pelo，Upf3b，Msi2，Nudt21，Cstf3，Ppp2r3a，Ddx39b，Magohb，Pabpc4，Fip1l1，Hbs1l，Ppp2r3d，Smg7，Nxt2
03018	RNA degradation	0	Folding, sorting and degradation	32	C1d，Exosc5，Exosc9，Eno2，Cnot4，Skiv2l，Xrn2，Tob1，Lsm1，Dcps，Dhx36，Exosc4，Cnot6l，Cnot6，Edc4，Exosc8，Cnot7，Hspa9，Exosc3，Dcp1a，Cnot3，Lsm6，Parn，Btg1，Skiv2l2，Ddx6，Exosc7，Lsm8，Cnot9，Pabpc4，Pnpt1，Cnot2
03022	Basal transcription factors	0	Transcription	16	Taf6，Taf12，Gtf2e1，Taf4b，Gtf2a1，Tbpl1，Taf5，Gtf2h4，Taf1，Taf10，Gtf2e2，Gtf2ird1，Taf4，Taf6l，Gtf2h1，Taf2
03030	DNA replication	0	Replication and repair	25	Mcm5，Mcm4，Lig1，Prim2，Rfc1，Pola1，Pole4，Rfc3，Mcm3，Rnaseh2c，Rfc4，Mcm6，Mcm7，Pold3，Pold2，Pole，Mcm2，Rpa3，Prim1，Dna2，Ssbp1，Rnaseh2a，Rpa2，Pold1，Rfc2

03040	Spliceosome	0	Transcription	65	Wbp11，Srsf2，Dhx15，Srsf3，Snrpc，Magoh，Srsf6，Snrpa1，Srsf9，Rbm8a，Hnrnpc，Snrpb，Tra2b，Prpf19，Crnkl1，Acin1，Prpf8，Snrpd3，Ddx5，Sf3a3，Dhx16，Srsf10，Srsf7，Ddx46，Slu7，Hnrnpm，Rbmx，Cdc5l，Snrpf，Prpf38b，Ppil1，Sf3b2，Prpf40a，Sf3b3，Aqr，Hspa8，Prpf38a，Ncbp1，Xab2，Lsm6，Bcas2，Rbm25，Sf3b5，Eftud2，Tra2a，Sf3a1，Rbm17，Ddx39b，Plrg1，Snrpg，Prpf31，Sart1，Lsm8，Tcerg1，Magohb，Snrpb2，Srsf1，Snrpd2，Snrnp40，Dhx38，Prpf6，U2surp，Pqbp1，Snrnp200，Snrnp27
03050	Proteasome	0	Folding, sorting and degradation	22	Psmd2，Psmb7，Psmc4，Psmc6，Psme3，Psmd4，Psma7，Psmd6，Psme4，Psmd11，Psmc2，Psma1，Psmb8，Psmd13，Psmb1，Psmd3，Psmb10，Psmb6，Psmb9，Psmd7，Psmb3，Psma4
03320	PPAR signaling pathway	0	Endocrine system	17	Scd2，Acadm，Pdpk1，Cpt2，Rxrb，Cyp27a1，Scp2，Acsl1，Gk，Pck2，Lpl，Cpt1c，Sorbs1，Acadl，Fads2，Cd36，Acsl4
03410	Base excision repair	0	Replication and repair	20	Xrcc1，Lig1，Mpg，Parp2，Nthl1，Parp1，Pole4，Ung，Apex2，Parp3，Polb，Lig3，Pold3，Pold2，Pole，Ogg1，Mbd4，Parp4，Neil1，Pold1
03420	Nucleotide excision repair	0	Replication and repair	27	Lig1，Cul4b，Rfc1，Ccnh，Xpc，Pole4，Rfc3，Rad23b，Mnat1，Ercc8，Ercc4，Ddb2，Gtf2h4，Rfc4，Pold3，Pold2，Ercc3，Pole，Rpa3，Ercc5，Cul4a，Gtf2h1，Rpa2，Rad23a，Pold1，Rfc2，Xpa
03430	Mismatch repair	0	Replication and repair	16	Lig1，Msh6，Msh2，Exo1，Rfc1，Rfc3，Msh3，Mlh3，Rfc4，Pold3，Pold2，Rpa3，Ssbp1，Rpa2，Pold1，Rfc2
03440	Homologous recombination	0	Replication and repair	14	Mre11a，Rad51，Brca2，Rad54l，Rad51d，Rad54b，Rad51c，Pold3，Pold2，Rpa3，Blm，Ssbp1，Rpa2，Pold1

04012	ErbB signaling pathway	0	Signal transduction	42	Map2k2，Crk，Akt1，Mtor，Pik3r2，Pak1，Nck1，Nrg4，Mapk9，Sos1，Elk1，Crkl，Stat5b，Rps6kb2，Camk2d，Pik3cg，Camk2g，Myc，Pik3r1，Araf，Pik3ca，Nck2，Ptk2，Cdkn1b，Eif4ebp1，Pik3r5，Akt3，Egfr，Pak3，Abl1，Pak6，Grb2，Cbl，Gsk3b，Kras，Sos2，Pik3cb，Pik3cd，Cblb，Abl2，Braf，Rps6kb1
04070	Phosphatidylinositol signaling system	0	Signal transduction	32	Pip4k2c，Itpr3，Pik3r2，Cds2，Dgkg，Pi4k2b，Pik3cg，Pikfyve，Inpp5d，Pik3r1，Pik3c2a，Pip5k1a，Dgkz，Itpr2，Synj2，Cds1，Pik3ca，Pi4k2a，Dgkd，Inpp5a，Pik3r5，Pi4ka，Pip4k2b，Itpkb，Pi4kb，Inpp1，Plcb3，Pten，Inpp5b，Pik3cb，Pik3cd，Impa2
04110	Cell cycle	0	Cell growth and death	68	Anapc2，Ccnd3，Mcm5，Orc3，Rad21，Mcm4，Ccne1，Wee1，Cdkn2c，Cdc6，Orc6，Cdc25a，Ccnd1，Anapc1，Smc1a，Rb1，E2f1，Orc2，Dbf4，Anapc7，Ccnh，Smad2，Ywhag，Ywhaq，Atm，Stag1，Cdc25c，Cdk4，Smad4，Ccne2，Chek2，Anapc4，Rbl1，Myc，Mad1l1，Rbl2，Cdc7，Mcm3，Mdm2，Ccnb3，Cdc14b，Cdc23，Cdkn1b，E2f3，Skp2，Cdc14a，Ywhaz，Tfdp2，Ywhae，Mcm6，Atr，Mcm7，Abl1，Bub3，Mcm2，Hdac2，Gadd45a，Gadd45b，Smad3，Chek1，Gsk3b，Prkdc，Anapc5，Cdc27，E2f2，Ccnd2，E2f5，Mad2l2
04114	Oocyte meiosis	0	Cell growth and death	42	Anapc2，Ccne1，Itpr3，Rps6ka2，Anapc1，Smc1a，Cpeb1，Anapc7，Adcy9，Prkacb，Ppp3cc，Ywhag，Ywhaq，Adcy7，Cdc25c，Ccne2，Camk2d，Anapc4，Camk2g，Prkx，Aurka，Fbxw11，Slk，Ppp1cb，Igf1r，Ppp2r5b，Itpr2，Ppp2r5e，Ppp3cb，Fbxo5，Cdc23，Ywhaz，Ywhae，Sgol1，Chp1，Prkaca，Igf1，Ppp3ca，Rps6ka3，Anapc5，Cdc27，Mad2l2

04115	p53 signaling pathway	0	Cell growth and death	35	Ccnd3，Cd82，Ccne1，Bax，Ccnd1，Pmaip1，Serpine1，Ccng1，Atm，Cdk4，Ccne2，Chek2，Bbc3，Casp3，Mdm2，Ccnb3，Rrm2b，Ddb2，Casp9，Atr，Rrm2，Ccng2，Bid，Zmat3，Gadd45a，Gadd45b，Apaf1，Pten，Chek1，Sesn2，Igf1，Sesn3，Sesn1，Ccnd2，Fas
04120	Ubiquitin mediated proteolysis	0	Folding, sorting and degradation	66	Huwe1，Anapc2，Ube2r2，Itch，Ube2d2a，Stub1，Ube3a，Ube2e1，Ube2g2，Cul7，Ube2l6，Cul4b，Anapc1，Anapc7，Pias2，Prpf19，Vhl，Birc3，Anapc4，Ube2d1，Ube2m，Brca1，Map3k1，Fbxw7，Fbxw11，Herc2，Fbxw8，Uba7，Trim32，Mdm2，Ube2h，Sae1，Uba3，Ercc8，Ube2f，Cdc23，Herc3，Ube2q2，Ube2o，Herc1，Trim37，Cul3，Cdc34，Skp2，Ddb2，Tceb2，Birc6，Uba1，Wwp2，Ube4b，Cbl，Trip12，Cul4a，Ube2w，Wwp1，Tceb1，Ppil2，Anapc5，Cdc27，Cblb，Pias4，Ube2d3，Pias1，Socs3，Xiap，Pml
04130	SNARE interactions in vesicular transport	0	Folding, sorting and degradation	19	Gosr1，Stx8，Stx7，Vamp2，Snap23，Vamp3，Vamp4，Snap47，Stx3，Bnip1，Stx17，Vamp5，Stx18，Use1，Stx4a，Vamp7，Stx11，Stx6，Ykt6
04141	Protein processing in endoplasmic reticulum	0	Folding, sorting and degradation	79	Sec61a1，Capn2，Hsp90ab1，Bag1，Ube2d2a，Nfe2l2，Stub1，Bax，Hspbp1，Ube2e1，Ube2g2，Man1a，Atf6b，Fbxo6，Ddit3，Dad1，Rpn2，Bak1，Ero1l，Sec63，Xbp1，Man1a2，Dnajb12，Eif2ak4，Mapk9，Mogs，Canx，Stt3a，Lman2，Derl1，Sec23a，Pdia3，Hsph1，Pdia6，Edem2，Sar1a，Txndc5，Ube2d1，Edem1，Rad23b，Ero1lb，Rrbp1，Ckap4，Sec24b，Sec24d，Nsfl1c，Sel1l，Hspa8，Mbtps1，Atxn3，Plaa，Herpud1，Man1c1，Mbtps2，Yod1，Ern1，Ganab，Hsp90b1，Map3k5，Eif2ak2，Atf4，Ppp1r15a，Ube4b，Wfs1，Ubqln4，Hspa4l，Dnajc3，Amfr，Hyou1，Vimp，Man1b1，Lman1，Rad23a，Ube2d3，Preb，Atf6，Bcl2，Dnajc1，Dnaja1

04142	Lysosome	0	Transport and catabolism	46	Atp6v1h，Gm2a，Ctns，Lamp2，M6pr，Npc2，Naglu，Ap3d1，Ap1g2，Gaa，Psap，Ppt1，Ap3s1，Sort1，Ap1g1，Pla2g15，Atp6v0a2，Acp2，Ctse，Gga2，Gusb，Abcb9，Mcoln1，Clta，Gnptab，Laptm5，Laptm4b，Fuca1，Ctsc，Cltc，Ctsh，Ap3m1，Neu1，Ctsb，Lgmn，Gla，Ctsf，Manba，Hgsnat，Slc11a2，Galc，Nagpa，Ap1s3，Ap4e1，Arsb，Atp6v0a1
04146	Peroxisome	0	Transport and catabolism	22	Pex12，Pex11a，Dhrs4，Abcd4，Scp2，Pex11b，Pex13，Acsl1，Slc25a17，Paox，Eci2，Abcd2，Pex19，Ech1，Pecr，Idh2，Crot，Acsl4，Far1，Pex6，Sod2，Hsd17b4
04150	mTOR signaling pathway	0	Signal transduction	30	Mlst8，Pdpk1，Akt1，Rps6ka2，Mtor，Ulk2，Cab39，Pik3r2，Stk11，Rps6kb2，Pik3cg，Pik3r1，Eif4b，Strada，Ddit4，Pik3ca，Rptor，Eif4ebp1，Pik3r5，Eif4e2，Akt3，Hif1a，Eif4e，Vegfb，Igf1，Rps6ka3，Pik3cb，Pik3cd，Braf，Rps6kb1
04260	Cardiac muscle contraction	0	Circulatory system	14	Uqcrb，Cyc1，Fxyd2，Tpm2，Tpm1，Tnnt2，Atp2a2，Tpm3，Uqcrc1，Uqcrc2，Uqcrq，Uqcr11，Cox5b，Cacng8
04270	Vascular smooth muscle contraction	0	Circulatory system	29	Map2k2，Itpr3，Pla2g5，Adcy9，Prkacb，Adcy7，Gna12，Arhgef1，Actg2，Kcnu1，Rock1，Prkx，Calcrl，Ppp1cb，Itpr2，Araf，Kcnma1，Pla2g6，Prkch，Pla2g4a，Plcb3，Myh11，Adora2b，Prkaca，Rhoa，Ppp1r12a，Gnaq，Braf，Gna13
04310	Wnt signaling pathway	0	Signal transduction	51	Ctbp1，Ccnd3，Ruvbl1，Csnk2b，Csnk1e，Ccnd1，Fzd2，Csnk2a1，Wnt6，Nfatc3，Map3k7，Prkacb，Smad2，Ppp3cc，Apc，Mapk9，Smad4，Camk2d，Rock1，Camk2g，Prkx，Myc，Fbxw11，Nfatc2，Ppp2r5b，Axin1，Ppp2r5e，Ppp3cb，Nfatc1，Tcf7l2，Ctnnb1，Tbl1x，Fzd3，Nfat5，Wnt8b，Tbl1xr1，Chd8，Dvl2，Plcb3，Chp1，Smad3，Prkaca，Rhoa，Gsk3b，Senp2，Ppp3ca，Csnk2a2，Fzd5，Ccnd2，Nlk，Fzd6

04330	Notch signaling pathway	0	Signal transduction	13	Ctbp1，Rbpj，Notch1，Adam17，Cir1，Aph1a，Numb，Maml1，Kat2b，Jag1，Dvl2，Ncor2，Hdac2
04340	Hedgehog signaling pathway	0	Signal transduction	16	Csnk1e，Wnt6，Prkacb，Rab23，Bmp2，Prkx，Fbxw11，Smo，Csnk1g1，Ptch1，Csnk1g3，Shh，Wnt8b，Sufu，Prkaca，Gsk3b
04370	VEGF signaling pathway	0	Signal transduction	31	Map2k2，Akt1，Ptgs2，Sphk2，Pla2g5，Pik3r2，Nfatc3，Ppp3cc，Pik3cg，Pxn，Pik3r1，Nfatc2，Mapkapk2，Ppp3cb，Nfatc1，Pik3ca，Ptk2，Pla2g6，Mapkapk3，Pik3r5，Akt3，Casp9，Nfat5，Pla2g4a，Cdc42，Chp1，Mapk14，Kras，Ppp3ca，Pik3cb，Pik3cd
04520	Adherens junction	0	Cellular community - eukaryotes	28	Actg1，Vcl，Csnk2b，Ptpn1，Fyn，Csnk2a1，Map3k7，Smad2，Tgfbr1，Fer，Smad4，Acp1，Tgfbr2，Ptprj，Igf1r，Tcf7l2，Ctnnb1，Iqgap1，Insr，Egfr，Afdn，Sorbs1，Ssx2ip，Cdc42，Smad3，Rhoa，Csnk2a2，Nlk
04530	Tight junction	0	Cellular community - eukaryotes	40	Actg1，Vapa，Akt1，Csnk2b，Rras2，Prkci，Tjp3，Patj，Csnk2a1，Sptbn1，Myh9，Cdk4，Cask，Cldn13，Epb41，F11r，Cldn18，Zak，Sympk，Ctnnb1，Exoc4，Epb41l2，Prkch，Gnai2，Akt3，Afdn，Gnai3，Myh11，Pard6a，Cdc42，Mpp5，Rhoa，Pten，Tjp2，Ybx3，Kras，Myh10，Csnk2a2，Exoc3，Epb41l1
04540	Gap junction	0	Cellular community - eukaryotes	26	Map2k2，Itpr3，Tuba4a，Adcy9，Prkacb，Adcy7，Sos1，Prkx，Itpr2，Tubb2a，Gnai2，Egfr，Gnai3，Gja1，Map3k2，Grm1，Plcb3，Grb2，Pdgfa，Pdgfb，Prkaca，Kras，Sos2，Tubb2b，Map2k5，Gnaq
04664	Fc epsilon RI signaling pathway	0	Immune system	28	Map2k2，Akt1，Vav3，Fyn，Pla2g5，Pik3r2，Tnf，Gab2，Mapk9，Sos1，Pik3cg，Pdk1，Inpp5d，Map2k3，Pik3r1，Pik3ca，Pla2g6，Pik3r5，Akt3，Vav1，Ms4a2，Pla2g4a，Grb2，Mapk14，Kras，Sos2，Pik3cb，Pik3cd

04720	Long-term potentiation	0	Nervous system	24	Map2k2，Itpr3，Rps6ka2，Prkacb，Ppp3cc，Camk2d，Camk2g，Prkx，Ppp1cb，Itpr2，Ppp3cb，Araf，Rap1b，Grm1，Atf4，Plcb3，Chp1，Prkaca，Kras，Ppp3ca，Rps6ka3，Ppp1r12a，Gnaq，Braf
04722	Neurotrophin signaling pathway	0	Nervous system	60	Map2k2，Crk，Akt1，Bax，Rps6ka2，Pik3r2，Ywhag，Ywhaq，Irak4，Mapk9，Sos1，Crkl，Camk2d，Pik3cg，Irs1，Sort1，Csk，Pdk1，Camk2g，Map3k1，Pik3r1，Arhgdib，Mapkapk2，Rapgef1，Nfkb1，Bex3，Pik3ca，Irak3，Kidins220，Ntf3，Pik3r5，Rap1b，Akt3，Irak2，Map3k3，Ywhaz，Ywhae，Map3k5，Abl1，Atf4，Nfkbia，Rps6ka4，Irak1，Grb2，Cdc42，Rhoa，Zfp110，Gsk3b，Mapk14，Kras，Prdm4，Sos2，Rps6ka3，Pik3cb，Pik3cd，Map2k5，Ikbkb，Braf，Bcl2，Nfkbie
04730	Long-term depression	0	Nervous system	20	Map2k2，Itpr3，Pla2g5，Gna12，Igf1r，Itpr2，Ryr1，Araf，Pla2g6，Grid2，Gnai2，Gnai3，Grm1，Pla2g4a，Plcb3，Kras，Igf1，Gnaq，Braf，Gna13
04810	Regulation of actin cytoskeleton	0	Cell motility	71	Actg1，Map2k2，Vcl，Crk，Pip4k2c，Vav3，Rras2，Tiam1，Cfl2，Pfn2，Pik3r2，Limk2，Myh9，Apc，Pak1，Gna12，Arhgef1，Itgav，Sos1，Crkl，Pik3cg，Diaph3，Pikfyve，Brk1，Itga5，Rock1，Csk，Pxn，Itgal，Pik3r1，Pip5k1a，Ppp1cb，Diaph2，Cyfip2，Araf，Pik3ca，Ptk2，Iqgap1，Abi2，Pik3r5，Pip4k2b，Egfr，Arpc5l，Gsn，F2r，Pak3，Fgf12，Vav1，Dock1，Pak6，Arhgef7，Pdgfa，Fgf18，Cdc42，Msn，Pdgfb，Rhoa，Ezr，Kras，Sos2，Itgb1，Myh10，Pik3cb，Ppp1r12a，Pik3cd，Gng12，Ssh1，Ssh2，Itgb5，Braf，Gna13

04910	Insulin signaling pathway	0	Endocrine system	63	Map2k2，Crk，Pdpk1，Akt1，Ptpn1，Prkci，Mtor，Mknk1，Mknk2，Pik3r2，Prkacb，Mapk9，Sos1，Elk1，Crkl，Rps6kb2，Hk2，Pik3cg，Irs1，Prkag2，Prkab1，Inpp5d，Prkx，Pik3r1，Pck2，Ppp1cb，Rapgef1，Prkar2a，Acaca，Phkg2，Araf，Pik3ca，Rptor，Pde3b，Prkar1b，Insr，Phkb，Eif4ebp1，Pik3r5，Eif4e2，Prkab2，Akt3，Sorbs1，Flot2，Prkar2b，Hk3，Flot1，Grb2，Cbl，Prkaca，Eif4e，Gsk3b，Kras，Prkar1a，Sos2，Pik3cb，Pik3cd，Ikbkb，Cblb，Socs4，Socs3，Braf，Rps6kb1
04912	GnRH signaling pathway	0	Endocrine system	31	Map2k2，Itpr3，Pla2g5，Adcy9，Prkacb，Adcy7，Mapk9，Sos1，Elk1，Camk2d，Camk2g，Prkx，Map3k1，Map2k3，Itpr2，Pla2g6，Egfr，Map3k3，Pld1，Map3k2，Atf4，Pla2g4a，Plcb3，Grb2，Cdc42，Prkaca，Mapk14，Kras，Sos2，Gnaq，Map3k4
04914	Progesterone-mediated oocyte maturation	0	Endocrine system	40	Anapc2，Hsp90ab1，Akt1，Cdc25a，Rps6ka2，Anapc1，Cpeb1，Anapc7，Pik3r2，Adcy9，Prkacb，Adcy7，Mapk9，Cdc25c，Pik3cg，Anapc4，Prkx，Mad1l1，Pik3r1，Igf1r，Ccnb3，Araf，Pik3ca，Cdc23，Pde3b，Pik3r5，Gnai2，Akt3，Gnai3，Prkaca，Mapk14，Kras，Igf1，Rps6ka3，Pik3cb，Pik3cd，Anapc5，Cdc27，Braf，Mad2l2
04960	Aldosterone-regulated sodium reabsorption	0	Excretory system	13	Pdpk1，Pik3r2，Fxyd2，Pik3cg，Irs1，Pik3r1，Pik3ca，Insr，Pik3r5，Kras，Igf1，Pik3cb，Pik3cd
04962	Vasopressin-regulated water reabsorption	0	Excretory system	14	Dync1h1，Dynll2，Adcy9，Prkacb，Vamp2，Dctn4，Nsf，Dctn1，Prkx，Dctn2，Arhgdib，Rab11b，Prkaca，Stx4a
04970	Salivary secretion	0	Digestive system	17	Slc4a2，Itpr3，Slc12a2，Fxyd2，Adcy9，Prkacb，Vamp2，Adcy7，Kcnn4，Prkx，Cst3，Itpr2，Kcnma1，Plcb3，Bst1，Prkaca，Gnaq

04971	Gastric acid secretion	0	Digestive system	18	Slc4a2，Itpr3，Kcnj10，Adcy9，Prkacb，Adcy7，Camk2d，Camk2g，Prkx，Itpr2，Gnai2，Gnai3，Plcb3，Car2，Kcnq1，Prkaca，Ezr，Gnaq
04972	Pancreatic secretion	0	Digestive system	19	Slc4a2，Itpr3，Slc12a2，Pla2g5，Fxyd2，Adcy9，Adcy7，Atp2a2，Itpr2，Kcnma1，Pla2g6，Rap1b，Pla2g4a，Plcb3，Car2，Bst1，Kcnq1，Rhoa，Gnaq
04973	Carbohydrate digestion and absorption	0	Digestive system	12	Akt1，Pik3r2，Fxyd2，Hk2，Pik3cg，Pik3r1，Pik3ca，Pik3r5，Akt3，Hk3，Pik3cb，Pik3cd
05010	Alzheimer's disease	0	Neurodegenerative diseases	65	Ndufv1，Atp5g1，Atp5c1，Capn2，Ndufc1，Uqcrb，Ndufs5，Cyc1，Ndufb5，Ndufa5，Itpr3，Ndufa2，Sdhb，Ndufa12，Tnf，Ppp3cc，Adam17，Nae1，Ndufa4，Ndufs7，Ndufv3，Aph1a，Casp3，Atp2a2，Itpr2，Ndufab1，Ndufb10，Ppp3cb，Uqcrc1，Lpl，Ndufs6，Ndufb6，Uqcrc2，Ern1，Uqcrq，Atp5o，Sdhd，Casp9，Ndufs4，App，Hsd17b10，Ndufb8，Uqcr11，Bid，Sdhc，Lrp1，Casp7，Plcb3，Ndufa10，Il1b，Chp1，Adam10，Apaf1，Cdk5，Gsk3b，Ndufs2，Ppp3ca，Ndufv2，Ide，Cox5b，Mapt，Gnaq，Ndufa7，Atf6，Fas
05012	Parkinson's disease	0	Neurodegenerative diseases	46	Ndufv1，Atp5g1，Atp5c1，Ndufc1，Uqcrb，Ndufs5，Cyc1，Ube2g2，Ndufb5，Ube2l6，Ndufa5，Ndufa2，Sdhb，Ndufa12，Ndufa4，Ndufs7，Ndufv3，Casp3，Uba7，Ndufab1，Ndufb10，Uqcrc1，Lrrk2，Ndufs6，Ndufb6，Uqcrc2，Uqcrq，Atp5o，Sdhd，Casp9，Vdac1，Ndufs4，Slc25a5，Pink1，Uba1，Ndufb8，Uqcr11，Sdhc，Ndufa10，Apaf1，Ndufs2，Ndufv2，Cox5b，Ndufa7，Park7，Htra2

05016	Huntington's disease	0	Neurodegenerative diseases	63	Ndufv1，Atp5g1，Atp5c1，Ndufc1，Uqcrb，Polr2c，Ndufs5，Cyc1，Bax，Ndufb5，Ndufa5，Ndufa2，Sdhb，Ndufa12，Ift57，Dctn4，Dctn1，Bbc3，Ndufa4，Ndufs7，Dctn2，Ndufv3，Nrf1，Htt，Casp3，Ndufab1，Ndufb10，Polr2i，Uqcrc1，Taf4b，Ap2a2，Ndufs6，Ndufb6，Rcor1，Clta，Hip1，Tbpl1，Uqcrc2，Uqcrq，Atp5o，Sdhd，Casp9，Vdac1，Ndufs4，Slc25a5，Cltc，Grm1，Ndufb8，Uqcr11，Sdhc，Plcb3，Ndufa10，Hdac2，Apaf1，Ndufs2，Ap2b1，Taf4，Ndufv2，Cox5b，Sod2，Gnaq，Ndufa7，Gpx1
05210	Colorectal cancer	0	Cancers	34	Akt1，Bax，Msh6，Msh2，Ccnd1，Pik3r2，Smad2，Tgfbr1，Apc，Mapk9，Smad4，Pik3cg，Fos，Msh3，Myc，Tgfbr2，Pik3r1，Casp3，Axin1，Araf，Tcf7l2，Pik3ca，Ctnnb1，Pik3r5，Akt3，Casp9，Smad3，Rhoa，Gsk3b，Kras，Pik3cb，Pik3cd，Braf，Bcl2
05212	Pancreatic cancer	0	Cancers	40	Akt1，Ralbp1，Ccnd1，Rb1，E2f1，Rad51，Pik3r2，Brca2，Smad2，Bcl2l1，Tgfbr1，Mapk9，Cdk4，Smad4，Pik3cg，Rala，Tgfbr2，Pik3r1，Nfkb1，Araf，Pik3ca，Jak1，E2f3，Pik3r5，Ikbkg，Akt3，Egfr，Pld1，Casp9，Cdc42，Stat1，Smad3，Chuk，Vegfb，Kras，Pik3cb，Pik3cd，Ikbkb，E2f2，Braf
05213	Endometrial cancer	0	Cancers	28	Map2k2，Pdpk1，Akt1，Ccnd1，Pik3r2，Apc，Sos1，Elk1，Pik3cg，Myc，Pik3r1，Axin1，Araf，Tcf7l2，Pik3ca，Ctnnb1，Pik3r5，Akt3，Egfr，Casp9，Grb2，Pten，Gsk3b，Kras，Sos2，Pik3cb，Pik3cd，Braf
05214	Glioma	0	Cancers	32	Map2k2，Akt1，Ccnd1，Mtor，Rb1，E2f1，Pik3r2，Sos1，Cdk4，Camk2d，Pik3cg，Camk2g，Pik3r1，Igf1r，Mdm2，Araf，Pik3ca，E2f3，Pik3r5，Akt3，Egfr，Grb2，Pdgfa，Pdgfb，Pten，Kras，Igf1，Sos2，Pik3cb，Pik3cd，E2f2，Braf

05215	Prostate cancer	0	Cancers	47	Map2k2，Hsp90ab1，Ccne1，Pdpk1，Akt1，Ccnd1，Mtor，Rb1，E2f1，Pik3r2，Sos1，Ccne2，Pik3cg，Pik3r1，Igf1r，Nfkb1，Mdm2，Araf，Tcf7l2，Pik3ca，Ctnnb1，Cdkn1b，E2f3，Pik3r5，Ikbkg，Akt3，Egfr，Casp9，Hsp90b1，Atf4，Nfkbia，Grb2，Pdgfa，Gstp1，Pdgfb，Pten，Gsk3b，Chuk，Kras，Igf1，Sos2，Pik3cb，Pik3cd，Ikbkb，E2f2，Braf，Bcl2
05218	Melanoma	0	Cancers	29	Map2k2，Akt1，Ccnd1，Rb1，E2f1，Pik3r2，Cdk4，Pik3cg，Pik3r1，Igf1r，Mdm2，Araf，Pik3ca，E2f3，Pik3r5，Akt3，Egfr，Fgf12，Pdgfa，Fgf18，Pdgfb，Pten，Kras，Igf1，Pik3cb，Pik3cd，Mitf，E2f2，Braf
05219	Bladder cancer	0	Cancers	16	Map2k2，Ccnd1，Rb1，E2f1，Cdk4，Dapk3，Myc，Mdm2，Araf，E2f3，Egfr，Mmp9，Vegfb，Kras，E2f2，Braf
05220	Chronic myeloid leukemia	0	Cancers	45	Ctbp1，Map2k2，Crk，Akt1，Ccnd1，Rb1，E2f1，Pik3r2，Gab2，Bcl2l1，Tgfbr1，Sos1，Crkl，Stat5b，Cdk4，Smad4，Pik3cg，Runx1，Myc，Tgfbr2，Pik3r1，Nfkb1，Mdm2，Araf，Pik3ca，Cdkn1b，E2f3，Pik3r5，Ikbkg，Akt3，Abl1，Nfkbia，Hdac2，Grb2，Cbl，Smad3，Chuk，Kras，Sos2，Pik3cb，Pik3cd，Ikbkb，Cblb，E2f2，Braf
05221	Acute myeloid leukemia	0	Cancers	31	Map2k2，Akt1，Ccnd1，Mtor，Pik3r2，Spi1，Sos1，Stat5b，Rps6kb2，Pik3cg，Runx1，Myc，Pik3r1，Nfkb1，Araf，Tcf7l2，Pik3ca，Eif4ebp1，Pik3r5，Ikbkg，Akt3，Grb2，Chuk，Kras，Sos2，Pik3cb，Pik3cd，Ikbkb，Pml，Braf，Rps6kb1
05223	Non-small cell lung cancer	0	Cancers	28	Map2k2，Pdpk1，Akt1，Rxrb，Ccnd1，Rb1，E2f1，Pik3r2，Sos1，Cdk4，Pik3cg，Pik3r1，Araf，Pik3ca，E2f3，Pik3r5，Akt3，Egfr，Stk4，Casp9，Grb2，Kras，Sos2，Pik3cb，Pik3cd，Rarb，E2f2，Braf

05340	Primary immunodeficiency	0	Immune diseases	12	Il2rg，Ada，Rfxank，Tnfrsf13b，Lck，Ung，Ikbkg，Ptprc，Il7r，Tap2，Rfxap，Cd40
