# Supplementary material for: Integrative Transcriptomic Profiling Identifies TNF and IL1B as Candidate Key Early-Response Genes in Macrophages Infected with Smooth Brucella Using a Comprehensive Bioinformatic Approach
Source: Biology (Basel). 2025 May 21;14(5):579. doi: 10.3390/biology14050579 (PMC12109160; doi:10.3390/biology14050579)
Supplement: Supplementary file 1 [file biology-14-00579-s001.zip › Table S3.pdf]

Table S3 Differentially expressed probes and genes inside infected macrophages with smooth B.suis

4h postinfection

Up-regulated Probes or Genes

PROBEID	logFC	AveExpr	t	P.Value	adj.P.Val	SYMBOL	GENENAME	ENTREZID
1456225_x_at	3.297759989	7.290337149	39.43443915	6.93E-13	3.13E-08	Trib3	tribbles pseudokinase 3	228775
1426065_a_at	3.084265212	6.845987618	35.1129528	2.36E-12	5.33E-08	Trib3	tribbles pseudokinase 3	228775
1419666_x_at	2.65454547	7.091933068	27.46283007	3.15E-11	4.74E-07	Nupr1	nuclear protein transcription regulator 1	56312
1419665_a_at	3.121087598	7.089500512	24.56046141	1.02E-10	1.15E-06	Nupr1	nuclear protein transcription regulator 1	56312
1435626_a_at	1.91765383	7.230232154	22.88483872	2.13E-10	1.22E-06	Herpud1	homocysteine-inducible, endoplasmic reticulum stress-inducible, ubiquitin-like domain member 1	64209
1458299_s_at	1.920266968	8.21414668	22.61419451	2.41E-10	1.22E-06	Nfkbie	nuclear factor of kappa light polypeptide gene enhancer in B cells inhibitor, epsilon	18037
1448185_at	2.121376775	8.250230986	22.60347704	2.43E-10	1.22E-06	Herpud1	homocysteine-inducible, endoplasmic reticulum stress-inducible, ubiquitin-like domain member 1	64209
1451382_at	3.053993464	7.126286591	23.74071752	1.45E-10	1.22E-06	Chac1	ChaC, cation transport regulator 1	69065
1417516_at	1.756713443	7.111122401	21.9087691	3.36E-10	1.38E-06	Ddit3	DNA-damage inducible transcript 3	13198
1449519_at	2.606058195	6.921154788	21.90055979	3.37E-10	1.38E-06	Gadd45a	growth arrest and DNA-damage-inducible 45 alpha	13197
1433453_a_at	1.788518478	4.48627316	21.60636384	3.88E-10	1.46E-06	Abtb2	ankyrin repeat and BTB (POZ) domain containing 2	99382
1448135_at	1.473761703	10.9905883	21.08904	5.00E-10	1.73E-06	Atf4	activating transcription factor 4	11911

1455197_at	1.426911604	5.521283785	20.63853855	6.26E-10	2.02E-06	Rnd1	Rho family GTPase 1	223881
1419647_a_at	1.476879741	9.392487583	18.52637813	1.92E-09	5.77E-06	Ier3	immediate early response 3	15937
1431843_a_at	1.861067114	6.827063241	18.02636778	2.55E-09	7.18E-06	Nfkbie	nuclear factor of kappa light polypeptide gene enhancer in B cells inhibitor, epsilon	18037
1451340_at	1.238735337	6.680060859	17.78976136	2.92E-09	7.75E-06	Arid5a	AT rich interactive domain 5A (MRF1-like)	214855
1434976_x_at	1.574852081	9.001875679	17.190623	4.16E-09	1.02E-05	Eif4ebp1	eukaryotic translation initiation factor 4E binding protein 1	13685
1419132_at	1.812314153	10.52883272	17.12964314	4.32E-09	1.02E-05	Tlr2	toll-like receptor 2	24088
1433966_x_at	1.257506094	8.239061051	16.60947301	5.93E-09	1.34E-05	Asns	asparagine synthetase	27053
1438992_x_at	1.165763911	9.922461818	16.11281054	8.10E-09	1.52E-05	Atf4	activating transcription factor 4	11911
1421392_a_at	1.391890184	8.892632388	16.29723792	7.20E-09	1.52E-05	Birc3	baculoviral IAP repeat-containing 3	11796
1444402_at	2.948698771	6.068383919	16.14120184	7.95E-09	1.52E-05	Zc3h12c	zinc finger CCCH type containing 12C	244871
1417483_at	3.856044593	8.155749056	15.86641012	9.48E-09	1.71E-05	Nfkbiz	nuclear factor of kappa light polypeptide gene enhancer in B cells inhibitor, zeta	80859
1418133_at	1.637647197	6.616022289	15.43204345	1.26E-08	2.10E-05	Bcl3	B cell leukemia/lymphoma 3	12051
1427844_a_at	1.175422322	9.195105063	15.35318987	1.33E-08	2.14E-05	Cebpb	CCAAT/enhancer binding protein (C/EBP), beta	12608
1438855_x_at	1.847130052	9.677379024	15.23998252	1.43E-08	2.16E-05	Tnfaip2	tumor necrosis factor, alpha-induced protein 2	21928
1428306_at	2.092479025	5.492042271	15.23491012	1.44E-08	2.16E-05	Ddit4	DNA-damage-inducible transcript 4	74747
1417562_at	1.52920102	8.628858869	15.06467937	1.61E-08	2.30E-05	Eif4ebp1	eukaryotic translation initiation factor 4E binding protein 1	13685
1456413_at	1.842387622	4.958371743	15.04194245	1.64E-08	2.30E-05	Pde4dip	phosphodiesterase 4D interacting protein (myomegalin)	83679
1427689_a_at	1.508069555	8.849805561	14.90413678	1.80E-08	2.38E-05	Tnip1	TNFAIP3 interacting protein 1	57783

1415899_at	1.731229782	7.91163786	14.53916319	2.31E-08	2.98E-05	Junb	jun B proto-oncogene	16477
1442700_at	1.929040381	4.92871293	14.43973238	2.48E-08	3.07E-05	Pde4b	phosphodiesterase 4B, cAMP specific	18578
1457117_at	1.650455074	5.137144008	14.31850425	2.70E-08	3.21E-05	Nfe2l2	nuclear factor, erythroid derived 2, like 2	18024
1419212_at	1.880792153	7.298457773	14.26974907	2.80E-08	3.24E-05	Icosl	icos ligand	50723
1425615_a_at	1.251470099	7.278929994	14.04542205	3.29E-08	3.71E-05	Pck2	phosphoenolpyruvate carboxykinase 2 (mitochondrial)	74551
1416654_at	1.696709502	7.304863103	13.89724292	3.66E-08	3.93E-05	Slc31a2	solute carrier family 31, member 2	20530
1422474_at	1.301266538	5.970406908	13.65919416	4.36E-08	4.47E-05	Pde4b	phosphodiesterase 4B, cAMP specific	18578
1449984_at	3.69642979	9.598739135	13.60439219	4.54E-08	4.55E-05	Cxcl2	chemokine (C-X-C motif) ligand 2	20310
1416332_at	1.346609997	6.884111508	13.52170169	4.83E-08	4.74E-05	Cirbp	cold inducible RNA binding protein	12696
1416273_at	1.751304757	8.216081778	13.41769461	5.22E-08	4.91E-05	Tnfaip2	tumor necrosis factor, alpha-induced protein 2	21928
1439221_s_at	1.946017789	5.636342854	13.27118907	5.83E-08	5.26E-05	Cd40	CD40 antigen	21939
1428691_at	1.103116281	7.34602782	13.13102046	6.49E-08	5.53E-05	Chd2	chromodomain helicase DNA binding protein 2	244059
1425139_at	1.339009995	6.606304148	13.13807259	6.46E-08	5.53E-05	Sesn2	sestrin 2	230784
1427736_a_at	1.630936149	6.593027011	12.79535636	8.43E-08	6.79E-05	Ccrl2	chemokine (C-C motif) receptor-like 2	54199
1418854_at	1.028124686	6.132167735	12.61445111	9.72E-08	7.69E-05	Birc2	baculoviral IAP repeat-containing 2	11797
1455899_x_at	4.08939267	6.355926248	12.54186618	1.03E-07	8.01E-05	Socs3	suppressor of cytokine signaling 3	12702
1422054_a_at	1.158146745	5.469579433	12.46800421	1.09E-07	8.32E-05	Skil	SKI-like	20482
1435226_at	1.350791439	8.611900516	12.45278985	1.11E-07	8.32E-05	Rnf19b	ring finger protein 19B	75234
1448731_at	1.03203369	6.309640228	12.41003508	1.15E-07	8.47E-05	Il10ra	interleukin 10 receptor, alpha	16154
1423233_at	1.285633364	8.037545378	12.2948724	1.26E-07	9.00E-05	Cebpd	CCAAT/enhancer binding protein (C/EBP), delta	12609
1432478_a_at	1.447124404	8.187998979	12.23829983	1.32E-07	9.28E-05	Rnf19b	ring finger protein 19B	75234

1423134_at	1.01418555	8.464029839	12.19294032	1.37E-07	9.48E-05	Rilpl2	Rab interacting lysosomal protein-like 2	80291
1451095_at	1.101688819	8.549467455	12.0438932	1.54E-07	9.84E-05	Asns	asparagine synthetase	27053
1418326_at	1.142710654	7.896413507	12.041067	1.55E-07	9.84E-05	Slc7a5	solute carrier family 7 (cationic amino acid transporter, y+ system), member 5	20539
1421818_at	1.294708561	6.953911141	11.8934856	1.75E-07	0.000108168	Bcl6	B cell leukemia/lymphoma 6	12053
1436507_at	1.184514494	7.428051879	11.76520343	1.95E-07	0.000110116	Irak2	interleukin-1 receptor-associated kinase 2	108960
1424067_at	1.947825272	8.164310131	11.80332138	1.89E-07	0.000110116	Icam1	intercellular adhesion molecule 1	15894
1448021_at	1.951368677	6.813794655	11.7861369	1.92E-07	0.000110116	Fam46c	family with sequence similarity 46, member C	74645
1416488_at	1.395717878	6.066682891	11.47044116	2.51E-07	0.000134838	Ccng2	cyclin G2	12452
1457404_at	1.429358511	5.7259295	11.43872014	2.58E-07	0.000134838	Nfkbiz	nuclear factor of kappa light polypeptide gene enhancer in B cells inhibitor, zeta	80859
1452214_at	1.170208687	9.496088705	11.19611596	3.19E-07	0.000149465	Skil	SKI-like	20482
1448728_a_at	2.732437844	6.857990957	11.21930863	3.12E-07	0.000149465	Nfkbiz	nuclear factor of kappa light polypeptide gene enhancer in B cells inhibitor, zeta	80859
1427381_at	2.790456416	6.753699009	11.18509863	3.22E-07	0.000149465	Acod1	aconitate decarboxylase 1	16365
1456341_a_at	1.126154919	8.624726062	10.95790649	3.94E-07	0.000176011	Klf9	Kruppel-like factor 9	16601
1428468_at	1.045167258	7.092146213	10.91351812	4.10E-07	0.000176665	3110043O21Rik	RIKEN cDNA 3110043O21 gene	73205
1456212_x_at	3.657553329	5.672560597	10.81345414	4.49E-07	0.000187557	Socs3	suppressor of cytokine signaling 3	12702
1429682_at	1.14796105	5.078137373	10.71063701	4.93E-07	0.000196539	Fam46c	family with sequence similarity 46, member C	74645
1417856_at	1.219344471	6.44655841	10.66316705	5.15E-07	0.000199181	Relb	avian reticuloendotheliosis viral (v-rel) oncogene related B	19698

1423306_at	1.024366688	7.025845228	10.56279177	5.65E-07	0.000214255	Smim3	small integral membrane protein 3	106878
1423602_at	1.679316608	5.758187852	10.52160544	5.87E-07	0.000220753	Traf1	TNF receptor-associated factor 1	22029
1448175_at	1.338628044	8.509307157	10.45810847	6.23E-07	0.000228505	Ehd1	EH-domain containing 1	13660
1416010_a_at	1.22931143	9.297844974	10.28407982	7.34E-07	0.000258986	Ehd1	EH-domain containing 1	13660
1416011_x_at	1.341426549	8.881583502	10.29450124	7.27E-07	0.000258986	Ehd1	EH-domain containing 1	13660
1420331_at	1.253631625	9.310752879	10.12394722	8.55E-07	0.00028157	Clec4e	C-type lectin domain family 4, member e	56619
1416111_at	1.584490739	4.892407778	9.703217683	1.29E-06	0.000359219	Cd83	CD83 antigen	12522
1428637_at	1.245172984	6.967715319	9.57073761	1.47E-06	0.000381054	Dyrk2	dual-specificity tyrosine-(Y)-phosphorylation regulated kinase 2	69181
1449317_at	1.228618814	6.631654755	9.543105646	1.51E-06	0.000385941	Cflar	CASP8 and FADD-like apoptosis regulator	12633
1445452_at	1.596885537	5.957172428	9.286956989	1.97E-06	0.000452483	Traf1	TNF receptor-associated factor 1	22029
1449363_at	1.64529038	9.425526825	9.287935592	1.96E-06	0.000452483	Atf3	activating transcription factor 3	11910
1424996_at	1.064939559	8.087393606	9.111427299	2.36E-06	0.000507308	Cflar	CASP8 and FADD-like apoptosis regulator	12633
1417801_a_at	1.244821758	6.723842651	9.110338645	2.36E-06	0.000507308	Ppfibp2	PTPRF interacting protein, binding protein 2 (liprin beta 2)	19024
1433761_at	1.072946013	7.045158863	8.895262078	2.96E-06	0.00059884	Pde4dip	phosphodiesterase 4D interacting protein (myomegalin)	83679
1428288_at	1.100460414	8.085169419	8.82167875	3.21E-06	0.000631642	Klf9	Kruppel-like factor 9	16601
1460251_at	1.083043684	6.159487202	8.770382431	3.39E-06	0.000661686	Fas	Fas (TNF receptor superfamily member 6)	14102
1450414_at	1.542387161	8.694765612	8.552445491	4.30E-06	0.000769724	Pdgfb	platelet derived growth factor, B polypeptide	18591
1427348_at	1.271489544	5.728414836	8.538264081	4.36E-06	0.000771807	Zc3h12a	zinc finger CCCH type containing 12A	230738
1451599_at	1.245265303	6.124696307	8.496521181	4.57E-06	0.000798759	Sesn2	sestrin 2	230784
1416101_a_at	1.168425882	8.870782374	8.443016567	4.85E-06	0.000828246	Hist1h1c	histone cluster 1, H1c	50708

1449731_s_at	1.558057478	9.367992586	8.149925042	6.74E-06	0.001023715	Nfkbia	nuclear factor of kappa light polypeptide gene enhancer in B cells inhibitor, alpha	18035
1418932_at	1.1866696	6.245886436	8.014061891	7.88E-06	0.001168808	Nfil3	nuclear factor, interleukin 3, regulated	18030
1460415_a_at	1.369393778	6.239791361	7.823101703	9.84E-06	0.001354875	Cd40	CD40 antigen	21939
1418392_a_at	1.082905998	5.674270559	7.730503966	1.10E-05	0.001469809	Gbp3	guanylate binding protein 3	55932
1416576_at	1.671692852	5.814496151	7.714286123	1.12E-05	0.001479947	Socs3	suppressor of cytokine signaling 3	12702
1436994_a_at	1.094463263	8.312199734	7.670074253	1.18E-05	0.001528476	Hist1h1c	histone cluster 1, H1c	50708
1450413_at	1.690872519	6.988947156	7.65428848	1.20E-05	0.001543745	Pdgfb	platelet derived growth factor, B polypeptide	18591
1448306_at	1.217303866	10.17831879	7.402465439	1.63E-05	0.001879064	Nfkbia	nuclear factor of kappa light polypeptide gene enhancer in B cells inhibitor, alpha	18035
1434070_at	1.232061373	6.328390354	7.39023874	1.65E-05	0.001890041	Jag1	jagged 1	16449
1450744_at	1.031981629	8.302704587	7.263563969	1.93E-05	0.002105456	Ell2	elongation factor RNA polymerase II 2	192657
1452519_a_at	1.549699388	8.275828633	7.173030019	2.16E-05	0.002227398	Zfp36	zinc finger protein 36	22695
1433699_at	1.979403846	6.533249767	6.936052629	2.92E-05	0.002706199	Tnfaip3	tumor necrosis factor, alpha-induced protein 3	21929
1450957_a_at	1.21697311	9.804544222	6.914130164	3.00E-05	0.002745759	Sqstm1	sequestosome 1	18412
1419607_at	2.943397596	8.831363007	6.843471261	3.29E-05	0.002890923	Tnf	tumor necrosis factor	21926
1417168_a_at	1.012973746	5.478109401	6.838956842	3.31E-05	0.002897575	Usp2	ubiquitin specific peptidase 2	53376
1428735_at	2.972688423	4.840066932	6.809665454	3.43E-05	0.00296813	Cd69	CD69 antigen	12515
1418930_at	1.438599039	6.860143806	6.590062738	4.58E-05	0.003614287	Cxcl10	chemokine (C-X-C motif) ligand 10	15945
1453721_a_at	1.174058014	7.227536015	6.566616032	4.72E-05	0.003676685	Slc31a2	solute carrier family 31, member 2	20530
1460220_a_at	1.275341499	5.941872764	6.480135256	5.30E-05	0.003947781	Csf1	colony stimulating factor 1 (macrophage)	12977

1417262_at	2.674088974	6.825841163	6.333180632	6.45E-05	0.00449324	Ptgs2	prostaglandin-endoperoxide synthase 2	19225
1450829_at	1.199490519	5.680042713	6.047446573	9.56E-05	0.005810009	Tnfaip3	tumor necrosis factor, alpha-induced protein 3	21929
1416012_at	1.243645909	6.018147624	5.921596967	0.000114038	0.006461351	Ehd1	EH-domain containing 1	13660
1428750_at	1.046288363	6.316241963	5.613764494	0.000177241	0.008576963	Cdc42ep2	CDC42 effector protein (Rho GTPase binding) 2	104252
1424250_a_at	1.410270473	5.556364113	5.446141878	0.000226607	0.010039507	Arhgef3	Rho guanine nucleotide exchange factor (GEF) 3	71704
1449399_a_at	1.957658507	6.07902066	5.219606707	0.000317863	0.012564357	Il1b	interleukin 1 beta	16176
1424208_at	1.039416385	8.317735691	5.138595682	0.000359391	0.013655333	Ptger4	prostaglandin E receptor 4 (subtype EP4)	19219
1419721_at	1.120677022	6.460687954	4.867219487	0.000545965	0.017676661	Hcar2	hydroxycarboxylic acid receptor 2	80885
1449773_s_at	1.645561731	6.169354061	4.741561731	0.000664951	0.020387464	Gadd45b	growth arrest and DNA-damage-inducible 45 beta	17873
1417263_at	1.908802235	5.388031631	3.919104367	0.002548356	0.048556567	Ptgs2	prostaglandin-endoperoxide synthase 2	19225

Down-regulated Probes or Genes

PROBEID	logFC	AveExpr	t	P.Value	adj.P.Val	SYMBOL	GENENAME	ENTREZID
1456060_at	-1.836182222	8.446436382	-23.40802456	1.68E-10	1.22E-06	Maf	avian musculoaponeurotic fibrosarcoma oncogene homolog	17132
1420946_at	-1.556309075	5.747955681	-9.884309106	1.08E-06	0.00032231	Atrx	alpha thalassemia/mental retardation syndrome X-linked	22589
1442827_at	-1.51820092	4.334089663	-16.22086721	7.56E-09	1.52E-05	Tlr4	toll-like receptor 4	21898
1433751_at	-1.513919808	4.975323702	-11.75592191	1.97E-07	0.000110116	Slc39a10	solute carrier family 39 (zinc transporter), member 10	227059
1450267_at	-1.481565385	5.749555096	-11.29377964	2.93E-07	0.000147508	Tlr8	toll-like receptor 8	170744

1435465_at	-1.394148897	6.434343854	-9.226757277	2.09E-06	0.000471883	Kbtbd11	kelch repeat and BTB (POZ) domain containing 11	74901
1418774_a_at	-1.300545783	6.411221041	-11.27433774	2.98E-07	0.000147508	Atp7a	ATPase, Cu++ transporting, alpha polypeptide	11977
1439122_at	-1.26768766	6.067378468	-13.15272731	6.39E-08	5.53E-05	Ddx6	DEAD (Asp-Glu-Ala-Asp) box polypeptide 6	13209
1416619_at	-1.218812819	6.613647086	-9.567044182	1.48E-06	0.000381054	Vsir	V-set immunoregulatory receptor	74048
1434846_at	-1.20475363	6.280596933	-11.20361605	3.17E-07	0.000149465	Dennd4c	DENN/MADD domain containing 4C	329877
1420622_a_at	-1.187776217	11.19525893	-11.17518905	3.25E-07	0.000149465	Hspa8	heat shock protein 8	15481
1435828_at	-1.178940045	5.892650907	-10.47132177	6.16E-07	0.000228505	Maf	avian musculoaponeurotic fibrosarcoma oncogene homolog	17132
1415834_at	-1.177538066	8.235682768	-6.559527722	4.76E-05	0.003698615	Dusp6	dual specificity phosphatase 6	67603
1426370_at	-1.164789125	7.99321279	-13.67265638	4.32E-08	4.47E-05	Far1	fatty acyl CoA reductase 1	67420
1436746_at	-1.15917519	9.035727004	-9.153136453	2.26E-06	0.000494043	Wnk1	WNK lysine deficient protein kinase 1	232341
1460006_at	-1.152695035	4.876639117	-8.323030274	5.54E-06	0.000902473	Zfhx3	zinc finger homeobox 3	11906
1427285_s_at	-1.147113186	7.256864312	-8.637481085	3.91E-06	0.000726503	Malat1	metastasis associated lung adenocarcinoma transcript 1 (non-coding RNA)	72289
1426259_at	-1.138993007	6.985609664	-7.880041913	9.21E-06	0.001301448	Pank3	pantothenate kinase 3	211347
1428046_a_at	-1.105640861	5.145527147	-7.282362414	1.89E-05	0.002075788	Zfx	zinc finger protein X-linked	22764
1453550_a_at	-1.098857359	6.076654022	-14.99782486	1.69E-08	2.30E-05	Far1	fatty acyl CoA reductase 1	67420
1426371_at	-1.08643437	8.203134536	-15.75861757	1.02E-08	1.76E-05	Far1	fatty acyl CoA reductase 1	67420
1440315_at	-1.070177954	5.249399615	-8.301265664	5.68E-06	0.000919314	Mbnl1	muscleblind-like 1 (Drosophila)	56758
1427488_a_at	-1.066814276	5.669970428	-7.195207283	2.10E-05	0.002219124	Birc6	baculoviral IAP repeat-containing 6	12211
1448890_at	-1.063563447	6.397923699	-5.614405463	0.000177076	0.008576963	Klf2	Kruppel-like factor 2 (lung)	16598
1429432_at	-1.056802571	4.270018885	-11.71740978	2.03E-07	0.000111702	Prrc2c	proline-rich coiled-coil 2C	226562

1439450_x_at	-1.044243396	6.625338142	-11.15660383	3.30E-07	0.000150407	A230046K03Rik	RIKEN cDNA A230046K03 gene	319277
1450051_at	-1.040639272	6.590632547	-7.767355397	1.05E-05	0.001423209	Atrx	alpha thalassemia/mental retardation syndrome X-linked	22589
1439247_at	-1.038167925	5.128483221	-8.397155251	5.10E-06	0.000858579	Dock10	dedicator of cytokinesis 10	210293
1420623_x_at	-1.036545317	12.29132934	-11.23061496	3.09E-07	0.000149465	Hspa8	heat shock protein 8	15481
1447849_s_at	-1.030345722	7.65422376	-8.418398466	4.98E-06	0.000844815	Maf	avian musculoaponeurotic fibrosarcoma oncogene homolog	17132
1425597_a_at	-1.022642092	4.376056412	-9.796095417	1.18E-06	0.000344664	Qk	quaking	19317
1438031_at	-1.013535801	6.356737213	-8.538812069	4.36E-06	0.000771807	Rasgrp3	RAS, guanyl releasing protein 3	240168
1438666_at	-1.008322032	7.43906187	-14.4202412	2.52E-08	3.07E-05	Ldlrad3	low density lipoprotein receptor class A domain containing 3	241576
1427490_at	-1.007586144	5.358175688	-11.7492292	1.98E-07	0.000110116	Abcb7	ATP-binding cassette, sub-family B (MDR/TAP), member 7	11306
1429505_at	-1.004339697	5.377845226	-7.225140076	2.03E-05	0.002176733	Nbeal1	neurobeachin like 1	269198
