# Supplementary material for: Integrative Transcriptomic Profiling Identifies TNF and IL1B as Candidate Key Early-Response Genes in Macrophages Infected with Smooth Brucella Using a Comprehensive Bioinformatic Approach
Source: Biology (Basel). 2025 May 21;14(5):579. doi: 10.3390/biology14050579 (PMC12109160; doi:10.3390/biology14050579)
Supplement: Supplementary file 1 [file biology-14-00579-s001.zip › Table S4.pdf]

Table S4 Differentially expressed probes and genes inside infected macrophages with smooth B.melitensis

4h postinfection

Up-regulated Probes or Genes

PROBEID	logFC	AveExpr	t	P.Value	adj.P.Val	SYMBOL	GENENAME	ENTREZID
1438362_x_at	1.000664725	3.417693866	9.789360626	1.58E-07	6.19E-06	2310035C23Rik	RIKEN cDNA 2310035C23 gene	227446
1450939_at	1.000815203	4.776206807	9.584903825	2.03E-07	7.38E-06	Entpd1	ectonucleoside triphosphate diphosphohydrolase 1	12495
1455285_at	1.003007325	6.974699915	9.812341465	1.53E-07	6.07E-06	Slc31a1	solute carrier family 31, member 1	20529
1420951_a_at	1.003181057	6.544798305	8.901967641	4.87E-07	1.39E-05	Son	Son DNA binding protein	20658
1433515_s_at	1.004303175	4.897960785	9.738771135	1.68E-07	6.46E-06	Etnk1	ethanolamine kinase 1	75320
1450414_at	1.007450178	9.318255964	12.04912553	1.24E-08	1.01E-06	Pdgfb	platelet derived growth factor, B polypeptide	18591
1424595_at	1.008750426	6.997046462	10.07641827	1.11E-07	4.88E-06	F11r	F11 receptor	16456
1421013_at	1.00883827	7.831693005	7.982219375	1.72E-06	3.60E-05	Pitpnb	phosphatidylinositol transfer protein, beta	56305
1430784_a_at	1.009095569	5.469157186	12.82692546	5.63E-09	5.75E-07	Rptor	regulatory associated protein of MTOR, complex 1	74370
1451072_a_at	1.009732426	8.359478647	9.796176263	1.57E-07	6.15E-06	Rnf4	ring finger protein 4	19822
1439622_at	1.010224893	9.293957173	12.9209132	5.14E-09	5.39E-07	Rassf4	Ras association (RalGDS/AF-6) domain family member 4	213391

1420976_at	1.010678963	6.157893746	8.497427539	8.38E-07	2.09E-05	Man1a2	mannosidase, alpha, class 1A, member 2	17156
1426541_a_at	1.011635626	6.563546345	7.488494493	3.51E-06	6.22E-05	Endod1	endonuclease domain containing 1	71946
1460599_at	1.013599608	5.934955048	11.80233776	1.60E-08	1.22E-06	Ermp1	endoplasmic reticulum metallopeptidase 1	226090
1430289_a_at	1.014351514	7.161256374	9.271980537	3.02E-07	9.84E-06	Wdr77	WD repeat domain 77	70465
1435176_a_at	1.015301047	11.35734835	11.75444464	1.68E-08	1.25E-06	Id2	inhibitor of DNA binding 2	15902
1453763_at	1.016230375	4.905605604	11.40402466	2.45E-08	1.64E-06	Txndc11	thioredoxin domain containing 11	106200
1439397_at	1.016616485	6.520848773	11.35680669	2.58E-08	1.70E-06	Fmn1	formin 1	14260
1416543_at	1.016655535	10.25695047	10.37096476	7.86E-08	3.80E-06	Nfe2l2	nuclear factor, erythroid derived 2, like 2	18024
1452772_at	1.020539415	4.35615217	8.75565745	5.92E-07	1.62E-05	Tnks2	tankyrase, TRF1-interacting ankyrin-related ADP-ribose polymerase 2	74493
1449195_s_at	1.021814538	8.459772665	13.48188047	3.00E-09	3.69E-07	Cxcl16	chemokine (C-X-C motif) ligand 16	66102
1434352_at	1.022220659	8.060331445	9.166861766	3.45E-07	1.08E-05	Bmt2	base methyltransferase of 25S rRNA 2	101148
1451134_a_at	1.023713568	8.918105074	9.956257191	1.29E-07	5.36E-06	Tm2d2	TM2 domain containing 2	69742
1435349_at	1.02403151	9.717006572	12.22525663	1.03E-08	8.87E-07	Nrp2	neuropilin 2	18187
1429240_at	1.027347375	7.639361554	12.02962256	1.26E-08	1.02E-06	Stard4	StAR-related lipid transfer (START) domain containing 4	170459
1427763_a_at	1.027694083	7.47824333	13.52237063	2.89E-09	3.62E-07	Camk2d	calcium/calmodulin-dependent protein kinase II, delta	108058

1448482_at	1.030392269	5.320868702	7.345816569	4.35E-06	7.36E-05	Slc39a8	solute carrier family 39 (metal ion transporter), member 8	67547
1426836_s_at	1.031613339	9.643399101	12.7198749	6.26E-09	6.17E-07	Metap1	methionyl aminopeptidase 1	75624
1435727_s_at	1.032575734	8.555513852	13.28322783	3.62E-09	4.22E-07	Lima1	LIM domain and actin binding 1	65970
1439974_at	1.032706318	5.718776488	10.05842293	1.14E-07	4.96E-06	Fkbp15	FK506 binding protein 15	338355
1419482_at	1.033680687	10.83010177	13.78304794	2.27E-09	3.01E-07	C3ar1	complement component 3a receptor 1	12267
1425294_at	1.034738839	5.805393512	10.90786744	4.24E-08	2.39E-06	Slamf8	SLAM family member 8	74748
1426334_a_at	1.035400786	5.684368137	9.622231098	1.94E-07	7.13E-06	Bcl2l11	BCL2-like 11 (apoptosis facilitator)	12125
1439965_at	1.036323392	8.299096161	12.09230012	1.18E-08	9.78E-07	Slc43a2	solute carrier family 43, member 2	215113
1422526_at	1.03684016	6.216793947	9.101535582	3.76E-07	1.15E-05	Acsl1	acyl-CoA synthetase long-chain family member 1	14081
1416635_at	1.038323311	8.043212714	11.79086492	1.62E-08	1.23E-06	Smpdl3a	sphingomyelin phosphodiesterase, acid-like 3A	57319
1449222_at	1.039290895	8.79327411	13.27694982	3.64E-09	4.22E-07	Ebi3	Epstein-Barr virus induced gene 3	50498
1450295_s_at	1.039738808	7.577183737	9.755671828	1.65E-07	6.39E-06	Pvr	poliovirus receptor	52118
1420472_at	1.04055464	8.132593076	8.579123743	7.50E-07	1.94E-05	Mtpn	myotrophin	14489
1452137_at	1.040806612	6.209495206	8.828102698	5.37E-07	1.50E-05	Acbd3	acyl-Coenzyme A binding domain containing 3	170760
1428380_at	1.040905959	8.258724933	9.809087432	1.54E-07	6.08E-06	Atraid	all-trans retinoic acid induced differentiation factor	381629

1457528_at	1.042286677	6.240564745	8.707374349	6.31E-07	1.70E-05	Slc4a7	solute carrier family 4, sodium bicarbonate cotransporter, member 7	218756
1435058_x_at	1.043225371	6.323008781	6.861076048	9.14E-06	0.000131643	Stxbp3	syntaxin binding protein 3	20912
1450898_at	1.045491317	8.49877379	12.89519454	5.27E-09	5.48E-07	Mfsd14a	major facilitator superfamily domain containing 14A	15247
1450165_at	1.047864286	9.332772074	10.01783433	1.20E-07	5.10E-06	Slfn2	schlafen 2	20556
1452439_s_at	1.048241347	9.62746433	9.717799978	1.72E-07	6.56E-06	Srsf2	serine/arginine-rich splicing factor 2	20382
1450006_at	1.049005988	8.180128588	11.30516418	2.73E-08	1.75E-06	Ncoa4	nuclear receptor coactivator 4	27057
1456328_at	1.050611815	7.365089239	11.43344288	2.37E-08	1.60E-06	Bank1	B cell scaffold protein with ankyrin repeats 1	242248
1450157_a_at	1.050679667	7.037283376	9.149947876	3.53E-07	1.10E-05	Hmmr	hyaluronan mediated motility receptor (RHAMM)	15366
1420591_at	1.05104776	10.3283038	10.95749549	4.01E-08	2.31E-06	Gpr84	G protein-coupled receptor 84	80910
1447926_at	1.05281272	6.24592702	8.418810877	9.33E-07	2.27E-05	Arl5a	ADP-ribosylation factor-like 5A	75423
1419835_s_at	1.053156594	10.03754719	11.31258376	2.71E-08	1.75E-06	Plec	plectin	18810
1425862_a_at	1.054133285	6.492494793	7.560566087	3.16E-06	5.75E-05	Pik3c2a	phosphatidylinositol 3-kinase, C2 domain containing, alpha polypeptide	18704
1418099_at	1.055177876	7.467134658	14.12925322	1.65E-09	2.44E-07	Tnfrsf1b	tumor necrosis factor receptor superfamily, member 1b	21938

1416190_a_at	1.05712823	7.299492257	9.067026082	3.93E-07	1.19E-05	Sec61a1	Sec61 alpha 1 subunit (S. cerevisiae)	53421
1422473_at	1.060594594	6.416404485	8.775463347	5.76E-07	1.58E-05	Pde4b	phosphodiesterase 4B, cAMP specific	18578
1434773_a_at	1.060795169	10.14643592	12.69176206	6.44E-09	6.27E-07	Slc2a1	solute carrier family 2 (facilitated glucose transporter), member 1	20525
1426708_at	1.064631663	9.797382572	12.82660527	5.64E-09	5.75E-07	Antxr2	anthrax toxin receptor 2	71914
1453683_a_at	1.065920522	5.973129372	7.687943138	2.62E-06	4.98E-05	Cep55	centrosomal protein 55	74107
1421285_at	1.066768732	7.118690991	10.01880064	1.19E-07	5.10E-06	Pik3ap1	phosphoinositide-3-kinase adaptor protein 1	83490
1448161_a_at	1.067259407	7.659619588	11.26122586	2.86E-08	1.81E-06	Clcn4	chloride channel, voltage-sensitive 4	12727
1423668_at	1.07139201	7.196199302	11.86331471	1.50E-08	1.17E-06	Zdhhc14	zinc finger, DHHC domain containing 14	224454
1419099_x_at	1.074858551	8.528502149	8.059570612	1.54E-06	3.32E-05	Stom	stomatin	13830
1453960_a_at	1.07555122	9.487853045	10.58947223	6.10E-08	3.17E-06	Capzb	capping protein (actin filament) muscle Z-line, beta	12345
1455480_s_at	1.07699149	11.16292647	9.644810998	1.89E-07	7.01E-06	Ube2d3	ubiquitin-conjugating enzyme E2D 3	66105
1422101_at	1.080838215	5.602000351	7.838206084	2.11E-06	4.22E-05	Tnfrsf23	tumor necrosis factor receptor superfamily, member 23	79201
1425932_a_at	1.081420263	6.716531102	7.578150061	3.08E-06	5.64E-05	Celf1	CUGBP, Elav-like family member 1	13046
1451158_at	1.083041355	6.486003695	10.55439695	6.35E-08	3.25E-06	Trip12	thyroid hormone receptor interactor 12	14897
1451913_a_at	1.083376249	5.916922932	8.551340904	7.79E-07	1.99E-05	Hyou1	hypoxia up-regulated 1	12282

1416700_at	1.085085209	5.222494166	11.83809266	1.54E-08	1.18E-06	Rnd3	Rho family GTPase 3	74194
1423797_at	1.085471909	7.206215725	14.38742961	1.31E-09	2.11E-07	Aacs	acetoacetyl-CoA synthetase	78894
1417933_at	1.086512389	5.735964986	13.3447948	3.42E-09	4.01E-07	Igfbp6	insulin-like growth factor binding protein 6	16012
1435066_at	1.086823996	7.144530415	12.10718836	1.16E-08	9.70E-07	Pitpnc1	phosphatidylinositol transfer protein, cytoplasmic 1	71795
1416529_at	1.088764705	9.794480368	9.40442938	2.55E-07	8.72E-06	Emp1	epithelial membrane protein 1	13730
1450977_s_at	1.088781995	7.886838247	14.08166553	1.72E-09	2.51E-07	Ndrg1	N-myc downstream regulated gene 1	17988
1421038_a_at	1.089230507	7.201493877	11.90278113	1.44E-08	1.13E-06	Kcnn4	potassium intermediate/small conductance calcium-activated channel, subfamily N, member 4	16534
1429502_at	1.089998265	6.555714388	10.09907819	1.08E-07	4.80E-06	Hspa13	heat shock protein 70 family, member 13	110920
1433668_at	1.093415203	9.699323279	11.06169631	3.57E-08	2.13E-06	Pnrc1	proline-rich nuclear receptor coactivator 1	108767
1423743_at	1.095177698	8.338866481	10.75933498	5.02E-08	2.76E-06	Arcn1	archain 1	213827
1453988_a_at	1.097718313	6.073427062	6.000250702	3.69E-05	0.000402651	Ide	insulin degrading enzyme	15925
1416835_s_at	1.097738071	8.784269583	11.55065192	2.09E-08	1.47E-06	Amd1	S-adenosylmethionine decarboxylase 1	11702
1417661_at	1.098089198	7.253167358	12.95360279	4.98E-09	5.25E-07	Rdm1	RAD52 motif 1	66599
1416794_at	1.09889017	7.398605451	9.295487767	2.93E-07	9.63E-06	Atl2	atlastin GTPase 2	56298

1435945_a_at	1.099839439	8.757233426	11.83740055	1.54E-08	1.18E-06	Kcnn4	potassium intermediate/small conductance calcium-activated channel, subfamily N, member 4	16534
1460191_at	1.100740884	7.698481827	9.754294372	1.65E-07	6.39E-06	Ykt6	YKT6 v-SNARE homolog (S. cerevisiae)	56418
1425326_at	1.102058819	8.6820924	10.11873679	1.06E-07	4.73E-06	Acly	ATP citrate lyase	104112
1422499_at	1.103068537	7.957789884	12.36526632	8.94E-09	8.04E-07	Lima1	LIM domain and actin binding 1	65970
1452784_at	1.104885671	9.180839584	13.81795384	2.19E-09	2.96E-07	Itgav	integrin alpha V	16410
1433933_s_at	1.105550243	5.908144119	9.68614599	1.79E-07	6.77E-06	Slco2b1	solute carrier organic anion transporter family, member 2b1	101488
1449670_x_at	1.108625988	8.029113699	6.399056084	1.91E-05	0.000236052	Gpr137b	G protein-coupled receptor 137B	83924
1450829_at	1.109643453	5.161894204	12.4501242	8.20E-09	7.50E-07	Tnfaip3	tumor necrosis factor, alpha-induced protein 3	21929
1417936_at	1.111876313	12.29109483	10.19484734	9.68E-08	4.43E-06	Ccl9	chemokine (C-C motif) ligand 9	20308
1453228_at	1.112180758	6.900063706	12.35623123	9.02E-09	8.05E-07	Stx11	syntaxin 11	74732
1416191_at	1.116167213	8.453557874	9.233250266	3.17E-07	1.02E-05	Sec61a1	Sec61 alpha 1 subunit (S. cerevisiae)	53421
1415779_s_at	1.116937007	12.06887692	12.53098459	7.56E-09	7.11E-07	Actg1	actin, gamma, cytoplasmic 1	11465
1450409_a_at	1.118843211	7.493952949	11.41140062	2.43E-08	1.62E-06	Slc48a1	solute carrier family 48 (heme transporter), member 1	67739

1449373_at	1.119339281	5.964494226	6.41876275	1.85E-05	0.000230633	Dnajc3	DnaJ heat shock protein family (Hsp40) member C3	100037258
1423545_a_at	1.119447775	9.920494636	12.34096046	9.16E-09	8.15E-07	Zfp207	zinc finger protein 207	22680
1427188_at	1.119867912	6.990805325	11.23508322	2.95E-08	1.85E-06	Arih1	ariadne RBR E3 ubiquitin protein ligase 1	23806
1452448_at	1.121690865	5.734443204	8.277260984	1.13E-06	2.64E-05	Aqr	aquarius	11834
1453769_at	1.122611629	7.146151089	12.35298951	9.05E-09	8.07E-07	Ckap2l	cytoskeleton associated protein 2-like	70466
1453470_a_at	1.122637993	7.114453415	12.53164529	7.56E-09	7.11E-07	Gna13	guanine nucleotide binding protein, alpha 13	14674
1450913_at	1.12515706	7.494912003	13.12592258	4.21E-09	4.70E-07	B4galt6	UDP-Gal:betaGlcNAc beta 1,4-galactosyltransferase, polypeptide 6	56386
1450876_at	1.125266111	2.894829481	12.80829922	5.74E-09	5.79E-07	Cfh	complement component factor h	12628
1455183_at	1.125349648	7.093686021	12.42460237	8.42E-09	7.67E-07	Stk38l	serine/threonine kinase 38 like	232533
1450378_at	1.125569434	7.313401432	9.609975071	1.97E-07	7.21E-06	Tapbp	TAP binding protein	21356
1418718_at	1.126823675	9.304974948	14.51274372	1.17E-09	1.95E-07	Cxcl16	chemokine (C-X-C motif) ligand 16	66102
1447100_s_at	1.128469269	5.270422497	9.045170613	4.04E-07	1.21E-05	5730508B09Rik	RIKEN cDNA 5730508B09 gene	70617
1416462_at	1.1292475	9.584837503	9.857551281	1.45E-07	5.84E-06	Caprin1	cell cycle associated protein 1	53872
1429684_at	1.130796762	6.07710664	9.418903262	2.50E-07	8.60E-06	Ttpal	tocopherol (alpha) transfer protein-like	76080

1415807_s_at	1.131465872	9.440581366	10.78195186	4.89E-08	2.71E-06	Srsf2	serine/arginine-rich splicing factor 2	20382
1452833_at	1.131626604	9.436184592	14.04644348	1.78E-09	2.56E-07	Rapgef2	Rap guanine nucleotide exchange factor (GEF) 2	76089
1425573_a_at	1.13351272	7.513039043	14.89067534	8.43E-10	1.58E-07	Asap1	ArfGAP with SH3 domain, ankyrin repeat and PH domain1	13196
1416296_at	1.134815361	9.353545574	12.75818169	6.03E-09	6.00E-07	Il2rg	interleukin 2 receptor, gamma chain	16186
1449317_at	1.134887499	7.516817867	14.65845689	1.03E-09	1.81E-07	Cflar	CASP8 and FADD-like apoptosis regulator	12633
1418352_at	1.135464236	4.863436454	10.99016662	3.87E-08	2.27E-06	Hsd17b2	hydroxysteroid (17-beta) dehydrogenase 2	15486
1422978_at	1.135524677	6.577279179	9.317713774	2.85E-07	9.44E-06	Cybb	cytochrome b-245, beta polypeptide	13058
1433492_at	1.136671291	7.030489764	13.06160945	4.48E-09	4.92E-07	Epb41l2	erythrocyte membrane protein band 4.1 like 2	13822
1448663_s_at	1.137220667	7.21234636	12.63357261	6.82E-09	6.58E-07	Mvd	mevalonate (diphospho) decarboxylase	192156
1425444_a_at	1.13805686	7.40796361	10.55739669	6.33E-08	3.25E-06	Tgfbr2	transforming growth factor, beta receptor II	21813
1418946_at	1.138189191	6.440090029	13.89624954	2.04E-09	2.80E-07	St3gal1	ST3 beta-galactoside alpha-2,3-sialyltransferase 1	20442
1415812_at	1.140760491	9.850311348	13.97809462	1.89E-09	2.67E-07	Gsn	gelsolin	227753
1449615_s_at	1.140888041	7.284966647	12.95925971	4.95E-09	5.23E-07	Hdlbp	high density lipoprotein (HDL) binding protein	110611

1423058_at	1.145437155	9.999243662	8.32487315	1.06E-06	2.52E-05	Capza2	capping protein (actin filament) muscle Z-line, alpha 2	12343
1432304_a_at	1.146502971	6.187105912	9.862122419	1.44E-07	5.82E-06	9030624J02Rik	RIKEN cDNA 9030624J02 gene	71517
1438040_a_at	1.149914519	10.28605187	10.14141077	1.03E-07	4.66E-06	Hsp90b1	heat shock protein 90, beta (Grp94), member 1	22027
1451619_at	1.153246862	6.911384764	12.93819032	5.05E-09	5.31E-07	Golph3l	golgi phosphoprotein 3-like	229593
1437463_x_at	1.156109377	10.06393884	10.73170264	5.18E-08	2.81E-06	Tgfbi	transforming growth factor, beta induced	21810
1455204_at	1.156150881	7.290717288	14.56711165	1.12E-09	1.90E-07	Pitpnc1	phosphatidylinositol transfer protein, cytoplasmic 1	71795
1417801_a_at	1.156166166	8.021695663	12.09770382	1.18E-08	9.74E-07	Ppfibp2	PTPRF interacting protein, binding protein 2 (liprin beta 2)	19024
1422776_at	1.156169453	6.89362293	13.17181873	4.03E-09	4.55E-07	Serpinb8	serine (or cysteine) peptidase inhibitor, clade B, member 8	20725
1448130_at	1.157500885	8.687154587	15.04444495	7.38E-10	1.46E-07	Fdft1	farnesyl diphosphate farnesyl transferase 1	14137
1416576_at	1.157884173	6.482410981	12.81865594	5.68E-09	5.77E-07	Socs3	suppressor of cytokine signaling 3	12702
1449591_at	1.160210769	8.218445056	13.37919906	3.31E-09	3.93E-07	Casp4	caspase 4, apoptosis-related cysteine peptidase	12363
1415988_at	1.160486954	6.819000473	10.35876122	7.97E-08	3.83E-06	Hdlbp	high density lipoprotein (HDL) binding protein	110611

1426599_a_at	1.161121526	9.322750362	12.36326785	8.95E-09	8.05E-07	Slc2a1	solute carrier family 2 (facilitated glucose transporter), member 1	20525
1451843_a_at	1.16148506	6.891902894	10.79690373	4.81E-08	2.67E-06	Ggta1	glycoprotein galactosyltransferase alpha 1, 3	14594
1424580_at	1.162459806	6.991592511	8.958908273	4.52E-07	1.32E-05	Slc35a3	solute carrier family 35 (UDP-N-acetylglucosamine (UDP-GlcNAc) transporter), member 3	229782
1450846_at	1.162804384	9.111612145	10.02293108	1.19E-07	5.08E-06	Bzw1	basic leucine zipper and W2 domains 1	66882
1418829_a_at	1.164471254	7.908727529	13.488877	2.98E-09	3.67E-07	Eno2	enolase 2, gamma neuronal	13807
1433582_at	1.166485912	8.927586936	13.21485633	3.87E-09	4.39E-07	1190002N15Rik	RIKEN cDNA 1190002N15 gene	68861
1451161_a_at	1.172133378	7.878962462	9.717517073	1.72E-07	6.56E-06	Adgre1	adhesion G protein-coupled receptor E1	13733
1452427_s_at	1.173030049	6.948598657	9.197553801	3.32E-07	1.05E-05	Hacd3	3-hydroxyacyl-CoA dehydratase 3	57874
1449209_a_at	1.181372411	7.802781694	12.88837848	5.31E-09	5.49E-07	Rdh11	retinol dehydrogenase 11	17252
1456028_x_at	1.183212984	8.883919306	11.66145603	1.86E-08	1.36E-06	Marcks	myristoylated alanine rich protein kinase C substrate	17118
1448865_at	1.184003649	7.752451429	12.49770858	7.82E-09	7.24E-07	Hsd17b7	hydroxysteroid (17-beta) dehydrogenase 7	15490
1438322_x_at	1.184076331	8.9810953	13.02371757	4.65E-09	5.02E-07	Fdft1	farnesyl diphosphate farnesyl transferase 1	14137
1420011_s_at	1.184226825	8.417074738	13.54472619	2.83E-09	3.57E-07	Xbp1	X-box binding protein 1	22433

1454677_at	1.186674083	9.155723539	14.26200292	1.46E-09	2.27E-07	Timp2	tissue inhibitor of metalloproteinase 2	21858
1419735_at	1.188173767	7.407825571	15.83657297	3.81E-10	9.27E-08	Csn3	casein kappa	12994
1450385_at	1.190171533	7.649880783	11.24228828	2.92E-08	1.84E-06	Kpna3	karyopherin (importin) alpha 3	16648
1454663_at	1.190457085	8.59814797	12.71220738	6.31E-09	6.19E-07	Eif5	eukaryotic translation initiation factor 5	217869
1422845_at	1.194628417	9.420889554	6.978453153	7.61E-06	0.000113883	Canx	calnexin	12330
1423804_a_at	1.200037252	8.449338202	7.427179068	3.85E-06	6.69E-05	Idi1	isopentenyl-diphosphate delta isomerase	319554
1418353_at	1.201489851	6.472062362	9.671452757	1.83E-07	6.85E-06	Cd5	CD5 antigen	12507
1422018_at	1.20163454	5.888088017	11.19181377	3.09E-08	1.93E-06	Hivep2	human immunodeficiency virus type I enhancer binding protein 2	15273
1435878_at	1.203742227	6.32176792	12.14366045	1.12E-08	9.43E-07	Stk38l	serine/threonine kinase 38 like	232533
1451457_at	1.205206905	8.778769258	12.51283235	7.70E-09	7.17E-07	Sc5d	sterol-C5-desaturase	235293
1456312_x_at	1.20575032	10.41345623	14.13603844	1.64E-09	2.44E-07	Gsn	gelsolin	227753
1423017_a_at	1.206986755	6.924377552	10.49267402	6.82E-08	3.42E-06	Il1rn	interleukin 1 receptor antagonist	16181
1423557_at	1.207685101	7.918627691	12.98785115	4.81E-09	5.11E-07	Ifngr2	interferon gamma receptor 2	15980
1436023_at	1.209050935	8.467570199	14.5140657	1.17E-09	1.95E-07	Bclaf1	BCL2-associated transcription factor 1	72567
1449578_at	1.209685373	6.848996387	9.024522157	4.15E-07	1.23E-05	Supt16	suppressor of Ty 16	114741
1421268_at	1.21045568	6.931347586	9.734402437	1.69E-07	6.49E-06	Ugcg	UDP-glucose ceramide glucosyltransferase	22234
1427736_a_at	1.213538498	7.08135232	12.47026864	8.04E-09	7.38E-07	Ccrl2	chemokine (C-C motif) receptor-like 2	54199

1450104_at	1.21417726	6.925966505	9.638921751	1.90E-07	7.04E-06	Adam10	a disintegrin and metallopeptidase domain 10	11487
1427329_a_at	1.215964939	6.634833932	13.95567977	1.93E-09	2.70E-07	Ighm	immunoglobulin heavy constant mu	16019
1451927_a_at	1.2167763	5.815304399	6.617295524	1.34E-05	0.000177791	Mapk14	mitogen-activated protein kinase 14	26416
1421839_at	1.218534015	6.907881722	9.053196262	4.00E-07	1.20E-05	Abca1	ATP-binding cassette, sub-family A (ABC1), member 1	11303
1430971_a_at	1.220035711	4.421884022	11.77889384	1.64E-08	1.23E-06	Aqr	aquarius	11834
1452638_s_at	1.226899902	5.82176128	9.576015555	2.06E-07	7.45E-06	Dnm1l	dynamin 1-like	74006
1435226_at	1.228561198	8.440835134	14.77661028	9.30E-10	1.70E-07	Rnf19b	ring finger protein 19B	75234
1425099_a_at	1.229048361	7.491679109	16.08215144	3.12E-10	8.09E-08	Arntl	aryl hydrocarbon receptor nuclear translocator-like	11865
1422054_a_at	1.230352682	5.641584065	12.54431329	7.46E-09	7.05E-07	Skil	SKI-like	20482
1451264_at	1.230490534	5.952248097	11.62278419	1.94E-08	1.40E-06	Frmd6	FERM domain containing 6	319710
1423418_at	1.231847268	10.18570482	14.25726439	1.47E-09	2.27E-07	Fdps	farnesyl diphosphate synthetase	110196
1439902_at	1.235742724	9.953089715	9.037839717	4.08E-07	1.22E-05	C5ar1	complement component 5a receptor 1	12273
1452161_at	1.236275171	7.918162085	10.73122328	5.18E-08	2.81E-06	Tiparp	TCDD-inducible poly(ADP-ribose) polymerase	99929
1438036_x_at	1.236579207	4.212737952	7.816197569	2.18E-06	4.31E-05	Rmdn2	regulator of microtubule dynamics 2	381110
1437244_at	1.243178836	7.496876126	9.91255971	1.36E-07	5.56E-06	Gas2l3	growth arrest-specific 2 like 3	237436

1452217_at	1.244026355	10.27470862	15.83745367	3.81E-10	9.27E-08	Ahnak	AHNAK nucleoprotein (desmoyokin)	66395
1429660_s_at	1.245565867	5.574456679	6.937122846	8.11E-06	0.000119912	Smc2	structural maintenance of chromosomes 2	14211
1456250_x_at	1.248514657	10.75637007	11.48606799	2.24E-08	1.54E-06	Tgfbi	transforming growth factor, beta induced	21810
1420852_a_at	1.253261988	7.558405534	13.14913119	4.12E-09	4.63E-07	B3gnt2	UDP-GlcNAc:betaGal beta-1,3-N-acetylglucosaminyltransferase 2	53625
1433491_at	1.254230347	7.279548476	11.27423963	2.82E-08	1.80E-06	Epb41l2	erythrocyte membrane protein band 4.1 like 2	13822
1456081_a_at	1.255450976	7.069482611	14.70926508	9.86E-10	1.76E-07	Aacs	acetoacetyl-CoA synthetase	78894
1448364_at	1.25941563	7.464926767	9.619132475	1.95E-07	7.15E-06	Ccng2	cyclin G2	12452
1427746_x_at	1.261122042	9.450568389	14.45047701	1.24E-09	2.04E-07	H2-K1	histocompatibility 2, K1, K region	14972
1421102_a_at	1.263004756	6.232932277	8.381000989	9.83E-07	2.36E-05	Vamp3	vesicle-associated membrane protein 3	22319
1435828_at	1.26393894	5.899281987	6.391051747	1.93E-05	0.000238559	Maf	avian musculoaponeurotic fibrosarcoma oncogene homolog	17132
1440837_at	1.267313448	5.942479204	7.28317408	4.78E-06	7.94E-05	H2-Ob	histocompatibility 2, O region beta locus	15002
1438035_at	1.268088711	4.017606117	5.829847481	4.93E-05	0.000505568	Rmdn2	regulator of microtubule dynamics 2	381110
1425929_a_at	1.268864326	6.795247506	10.93006273	4.14E-08	2.36E-06	Rnf14	ring finger protein 14	56736
1417752_at	1.270343178	7.42295903	10.26150669	8.94E-08	4.17E-06	Coro1c	coronin, actin binding protein 1C	23790

1425066_a_at	1.27345046	8.243326275	15.58843457	4.67E-10	1.06E-07	Mtmr14	myotubularin related protein 14	97287
1437171_x_at	1.273697396	10.20047085	13.95900108	1.93E-09	2.70E-07	Gsn	gelsolin	227753
1416389_a_at	1.275752344	9.202234123	15.18728351	6.54E-10	1.34E-07	Rcbtb2	regulator of chromosome condensation (RCC1) and BTB (POZ) domain containing protein 2	105670
1451956_a_at	1.276990137	7.831183765	12.07998258	1.20E-08	9.83E-07	Sigmar1	sigma non-opioid intracellular receptor 1	18391
1435342_at	1.281455145	9.250915485	16.3030494	2.62E-10	7.24E-08	Kcnk6	potassium inwardly-rectifying channel, subfamily K, member 6	52150
1421392_a_at	1.282521149	9.623623562	14.62365434	1.06E-09	1.84E-07	Birc3	baculoviral IAP repeat-containing 3	11796
1422863_s_at	1.284518336	7.475335653	7.818199172	2.17E-06	4.30E-05	Pdlim5	PDZ and LIM domain 5	56376
1424484_at	1.284735106	8.674349211	9.856433339	1.45E-07	5.84E-06	Mob1a	MOB kinase activator 1A	232157
1427483_at	1.28949673	7.686652354	10.51156103	6.67E-08	3.37E-06	Slc25a24	solute carrier family 25 (mitochondrial carrier, phosphate carrier), member 24	229731
1418133_at	1.289566846	6.709561882	12.68997007	6.45E-09	6.27E-07	Bcl3	B cell leukemia/lymphoma 3	12051
1424486_a_at	1.291035116	6.552555782	13.64951407	2.56E-09	3.34E-07	Txnrd1	thioredoxin reductase 1	50493
1426600_at	1.29324102	8.59275183	14.94838708	8.02E-10	1.55E-07	Slc2a1	solute carrier family 2 (facilitated glucose transporter), member 1	20525
1426721_s_at	1.294444403	8.882329131	16.21005615	2.82E-10	7.65E-08	Tiparp	TCDD-inducible poly(ADP-ribose) polymerase	99929

1448291_at	1.295020818	4.355942442	12.52626234	7.60E-09	7.12E-07	Mmp9	matrix metallopeptidase 9	17395
1426370_at	1.297325477	8.289261565	12.11839085	1.15E-08	9.61E-07	Far1	fatty acyl CoA reductase 1	67420
1450729_at	1.298517222	9.299266169	16.99327434	1.53E-10	4.95E-08	Hs2st1	heparan sulfate 2-O-sulfotransferase 1	23908
1426259_at	1.298968737	7.053706655	10.32640269	8.28E-08	3.95E-06	Pank3	pantothenate kinase 3	211347
1450744_at	1.299974075	8.336569605	14.25840557	1.47E-09	2.27E-07	Ell2	elongation factor RNA polymerase II 2	192657
1418711_at	1.303825443	7.202896692	14.39072859	1.31E-09	2.11E-07	Pdgfa	platelet derived growth factor, alpha	18590
1430127_a_at	1.30404209	6.40232815	12.69047378	6.45E-09	6.27E-07	Ccnd2	cyclin D2	12444
1436530_at	1.305652882	10.19992406	17.01572231	1.50E-10	4.90E-08	Wfdc17	WAP four-disulfide core domain 17	100034251
1448239_at	1.306364272	8.665219847	12.12659056	1.14E-08	9.55E-07	Hmox1	heme oxygenase 1	15368
1431197_at	1.306445191	6.896411707	12.81078969	5.73E-09	5.79E-07	Atl2	atlastin GTPase 2	56298
1422474_at	1.312597046	6.535328664	14.44715573	1.24E-09	2.04E-07	Pde4b	phosphodiesterase 4B, cAMP specific	18578
1436991_x_at	1.313351875	10.01111207	16.41622934	2.39E-10	6.86E-08	Gsn	gelsolin	227753
1448127_at	1.319659227	9.075827002	9.94782435	1.30E-07	5.40E-06	Rrm1	ribonucleotide reductase M1	20133
1421895_at	1.335146022	8.370995712	9.533569743	2.17E-07	7.73E-06	Eif2s3x	eukaryotic translation initiation factor 2, subunit 3, structural gene X-linked	26905
1417509_at	1.33571069	6.855690884	11.34965272	2.60E-08	1.70E-06	Rnf19a	ring finger protein 19A	30945
1418972_at	1.34394439	9.164509077	14.94064569	8.07E-10	1.55E-07	Bcl10	B cell leukemia/lymphoma 10	12042
1416326_at	1.345341717	11.31683159	16.40087288	2.42E-10	6.86E-08	Crip1	cysteine-rich protein 1 (intestinal)	12925
1428663_at	1.349370163	6.064774619	15.41771351	5.38E-10	1.16E-07	Sgms2	sphingomyelin synthase 2	74442

1438975_x_at	1.352545045	9.185978711	15.79314621	3.95E-10	9.36E-08	Zdhhc14	zinc finger, DHHC domain containing 14	224454
1415784_at	1.353712139	7.599884379	11.54421257	2.11E-08	1.48E-06	Vps35	VPS35 retromer complex component	65114
1452483_a_at	1.353836153	9.221693235	14.51360181	1.17E-09	1.95E-07	Cd44	CD44 antigen	12505
1418018_at	1.356673122	8.024860388	13.95640832	1.93E-09	2.70E-07	Cpd	carboxypeptidase D	12874
1437614_x_at	1.357204409	9.1291502	15.37191815	5.59E-10	1.19E-07	Zdhhc14	zinc finger, DHHC domain containing 14	224454
1417303_at	1.358872431	6.974470844	17.78431857	8.43E-11	3.30E-08	Mvd	mevalonate (diphospho) decarboxylase	192156
1421821_at	1.359136112	9.550685341	16.78504434	1.79E-10	5.53E-08	Ldlr	low density lipoprotein receptor	16835
1416122_at	1.365980317	6.784470429	9.623464764	1.94E-07	7.13E-06	Ccnd2	cyclin D2	12444
1457248_x_at	1.366166679	8.357924778	15.49611595	5.04E-10	1.10E-07	Hsd17b7	hydroxysteroid (17-beta) dehydrogenase 7	15490
1420816_at	1.367652252	6.605245266	6.297178635	2.26E-05	0.000269211	Ywhag	tyrosine 3-monooxygenase/tryptophan 5-monooxygenase activation protein, gamma polypeptide	22628
1431096_at	1.368754053	4.123802072	9.868344029	1.43E-07	5.80E-06	Ints8	integrator complex subunit 8	72656
1448123_s_at	1.368978023	10.79538878	15.48816266	5.08E-10	1.11E-07	Tgfbi	transforming growth factor, beta induced	21810
1417424_at	1.369937222	7.421264666	8.000596645	1.67E-06	3.54E-05	Ier3ip1	immediate early response 3 interacting protein 1	66191
1427229_at	1.375408085	8.716869411	8.496237833	8.39E-07	2.10E-05	Hmgcr	3-hydroxy-3-methylglutaryl-Coenzyme A reductase	15357

1431843_a_at	1.375835547	7.293909962	13.43144697	3.15E-09	3.84E-07	Nfkbie	nuclear factor of kappa light polypeptide gene enhancer in B cells inhibitor, epsilon	18037
1438619_x_at	1.385462003	9.316544738	17.09575674	1.41E-10	4.71E-08	Zdhhc14	zinc finger, DHHC domain containing 14	224454
1450105_at	1.38628312	7.389797997	9.587031288	2.03E-07	7.37E-06	Adam10	a disintegrin and metallopeptidase domain 10	11487
1448731_at	1.386654864	6.938763861	13.88757212	2.06E-09	2.80E-07	Il10ra	interleukin 10 receptor, alpha	16154
1425227_a_at	1.386804989	6.968535136	18.26982597	5.93E-11	2.70E-08	Atp6v0a1	ATPase, H+ transporting, lysosomal V0 subunit A1	11975
1451122_at	1.391258613	8.863999818	18.61885611	4.63E-11	2.30E-08	Idi1	isopentenyl-diphosphate delta isomerase	319554
1441811_x_at	1.392790101	7.770497725	12.98763264	4.82E-09	5.11E-07	Tmem176a	transmembrane protein 176A	66058
1455033_at	1.394185201	8.790948244	17.34317437	1.17E-10	4.12E-08	Fam102b	family with sequence similarity 102, member B	329739
1431768_a_at	1.395655828	5.537556651	7.651387296	2.76E-06	5.17E-05	Prmt3	protein arginine N-methyltransferase 3	71974
1415824_at	1.397653877	8.863313409	10.03693542	1.17E-07	5.03E-06	Scd2	stearoyl-Coenzyme A desaturase 2	20250
1422861_s_at	1.401409065	7.230283144	14.90692992	8.31E-10	1.58E-07	Pdlim5	PDZ and LIM domain 5	56376
1428587_at	1.401485635	8.378256158	15.15694974	6.71E-10	1.37E-07	Tmem41b	transmembrane protein 41B	233724
1423134_at	1.403257948	9.061691759	17.34951739	1.16E-10	4.12E-08	Rilpl2	Rab interacting lysosomal protein-like 2	80291

1449262_s_at	1.406042616	6.832757517	7.29295115	4.71E-06	7.85E-05	Lin7c	lin-7 homolog C (C. elegans)	22343
1426184_a_at	1.40764858	6.412721349	11.11511267	3.36E-08	2.05E-06	Pdcd6ip	programmed cell death 6 interacting protein	18571
1434339_at	1.407726068	9.045021876	12.32092186	9.35E-09	8.30E-07	Fnbp1l	formin binding protein 1-like	214459
AFFX-TransRecMur/X57349_M_at	1.413055355	4.3974011	14.09332939	1.71E-09	2.49E-07	Tfrc	transferrin receptor	22042
1415973_at	1.413250096	9.272129466	18.16595455	6.39E-11	2.77E-08	Marcks	myristoylated alanine rich protein kinase C substrate	17118
1417123_at	1.417280368	7.774032515	7.915328074	1.89E-06	3.86E-05	Vav3	vav 3 oncogene	57257
1419212_at	1.417910609	7.900613347	18.17385118	6.35E-11	2.77E-08	Icosl	icos ligand	50723
1455956_x_at	1.418598391	6.319836161	15.14902475	6.75E-10	1.37E-07	Ccnd2	cyclin D2	12444
1449773_s_at	1.420544115	6.180343508	14.65755715	1.03E-09	1.81E-07	Gadd45b	growth arrest and DNA-damage-inducible 45 beta	17873
1428025_s_at	1.421183985	5.84226038	14.25331908	1.48E-09	2.27E-07	Pitpnc1	phosphatidylinositol transfer protein, cytoplasmic 1	71795
1416157_at	1.425190557	5.441183542	13.38990743	3.27E-09	3.90E-07	Vcl	vinculin	22330
1434191_at	1.431179705	5.917144081	11.66890213	1.84E-08	1.36E-06	Agmo	alkylglycerol monooxygenase	319660
1449382_at	1.435330865	5.856209143	16.17686033	2.89E-10	7.70E-08	Slc6a12	solute carrier family 6 (neurotransmitter transporter, betaine/GABA), member 12	14411
1430671_a_at	1.437242246	6.443010929	8.720268678	6.20E-07	1.67E-05	Brox	BRO1 domain and CAAX motif containing	71678

1419135_at	1.443158638	7.612734926	12.15049188	1.11E-08	9.42E-07	Ltb	lymphotoxin B	16994
1417611_at	1.444634466	8.443410773	17.41291656	1.11E-10	4.01E-08	Tmem37	transmembrane protein 37	170706
1420886_a_at	1.446288704	10.41200471	16.50892043	2.22E-10	6.64E-08	Xbp1	X-box binding protein 1	22433
1423413_at	1.451320947	7.530859399	14.19628946	1.55E-09	2.38E-07	Ndrg1	N-myc downstream regulated gene 1	17988
1416653_at	1.455696113	8.615288244	14.99147375	7.73E-10	1.51E-07	Stxbp3	syntaxin binding protein 3	20912
1456700_x_at	1.456487503	9.546002492	16.16241703	2.93E-10	7.70E-08	Marcks	myristoylated alanine rich protein kinase C substrate	17118
1420899_at	1.458784105	7.85552844	10.61948945	5.89E-08	3.08E-06	Rab18	RAB18, member RAS oncogene family	19330
1448793_a_at	1.460075104	7.613483447	16.40671095	2.41E-10	6.86E-08	Sdc4	syndecan 4	20971
1442347_at	1.462035943	5.133223312	13.85683633	2.12E-09	2.87E-07	Lrp8	low density lipoprotein receptor-related protein 8, apolipoprotein e receptor	16975
1415893_at	1.46635946	6.869235725	11.38263473	2.51E-08	1.66E-06	Sgpl1	sphingosine phosphate lyase 1	20397
1460521_a_at	1.467116907	5.515291782	14.43864651	1.25E-09	2.04E-07	Nabp1	nucleic acid binding protein 1	109019
1449399_a_at	1.470757615	6.580151277	16.45269063	2.32E-10	6.80E-08	Il1b	interleukin 1 beta	16176
1430604_a_at	1.473855014	8.696437184	10.73929649	5.13E-08	2.80E-06	Dab2	disabled 2, mitogen-responsive phosphoprotein	13132
1450786_x_at	1.47723133	7.419461065	15.78105586	3.99E-10	9.36E-08	Pdlim5	PDZ and LIM domain 5	56376
1423909_at	1.480013294	8.587956819	18.48165039	5.10E-11	2.47E-08	Tmem176a	transmembrane protein 176A	66058
1460469_at	1.480787028	7.593690001	15.24283233	6.24E-10	1.30E-07	Tnfrsf9	tumor necrosis factor receptor superfamily, member 9	21942
1420924_at	1.483233451	6.87724152	14.0490552	1.78E-09	2.56E-07	Timp2	tissue inhibitor of metalloproteinase 2	21858

1450007_at	1.485759722	7.798810375	10.81641708	4.70E-08	2.63E-06	Chp1	calcineurin-like EF hand protein 1	56398
1460650_at	1.489162777	5.757841428	11.17882928	3.14E-08	1.95E-06	Atp6v0a1	ATPase, H+ transporting, lysosomal V0 subunit A1	11975
1435626_a_at	1.491002695	7.931416443	13.42491551	3.17E-09	3.85E-07	Herpud1	homocysteine-inducible, endoplasmic reticulum stress-inducible, ubiquitin-like domain member 1	64209
1417111_at	1.492769213	8.696130043	18.06733262	6.86E-11	2.87E-08	Man1a	mannosidase 1, alpha	17155
1428444_at	1.494757148	7.270688237	11.7799046	1.64E-08	1.23E-06	Asb2	ankyrin repeat and SOCS box-containing 2	65256
1432478_a_at	1.49487361	8.002142637	17.20092388	1.30E-10	4.42E-08	Rnf19b	ring finger protein 19B	75234
1422013_at	1.49831222	6.389820015	11.48393374	2.25E-08	1.54E-06	Clec4a2	C-type lectin domain family 4, member a2	26888
1432262_at	1.501296111	6.807266672	15.91012635	3.59E-10	9.09E-08	Fam63a	family with sequence similarity 63, member A	75007
1422537_a_at	1.506988895	10.36747275	18.34163937	5.63E-11	2.63E-08	Id2	inhibitor of DNA binding 2	15902
1416010_a_at	1.509200908	10.10030707	17.62979371	9.45E-11	3.57E-08	Ehd1	EH-domain containing 1	13660
1441709_at	1.515557112	5.05354351	15.60155686	4.62E-10	1.05E-07	Slc11a2	solute carrier family 11 (proton-coupled divalent metal ion transporters), member 2	18174
1416011_x_at	1.52239184	10.14509686	17.45035188	1.08E-10	3.93E-08	Ehd1	EH-domain containing 1	13660
1452315_at	1.523773872	6.910464224	15.8302138	3.83E-10	9.27E-08	Kif11	kinesin family member 11	16551
1449187_at	1.529674555	7.460197696	18.11179171	6.64E-11	2.80E-08	Pdgfa	platelet derived growth factor, alpha	18590
1431385_a_at	1.531200431	5.301014262	14.90001137	8.36E-10	1.58E-07	Mbtps1	membrane-bound transcription factor peptidase, site 1	56453

1450392_at	1.53461606	5.754495591	8.977331615	4.42E-07	1.30E-05	Abca1	ATP-binding cassette, sub-family A (ABC1), member 1	11303
1426849_at	1.534968509	6.616998552	14.35159293	1.35E-09	2.15E-07	Sec24b	Sec24 related gene family, member B (S. cerevisiae)	99683
1419030_at	1.535209287	8.50749572	13.88826554	2.06E-09	2.80E-07	Ero1l	ERO1-like (S. cerevisiae)	50527
1424942_a_at	1.536568615	7.117549651	15.29620555	5.96E-10	1.25E-07	Myc	myelocytomatosis oncogene	17869
1448898_at	1.537889873	11.48371598	12.51563458	7.68E-09	7.17E-07	Ccl9	chemokine (C-C motif) ligand 9	20308
1448303_at	1.540735897	7.074186897	6.891117408	8.72E-06	0.000126537	Gpnmb	glycoprotein (transmembrane) nmb	93695
1441907_s_at	1.541872022	5.7235132	16.06610884	3.16E-10	8.15E-08	Cd93	CD93 antigen	17064
1424996_at	1.543112583	8.164891631	15.96280916	3.44E-10	8.81E-08	Cflar	CASP8 and FADD-like apoptosis regulator	12633
1431030_a_at	1.543657799	4.583925166	10.64370758	5.73E-08	3.03E-06	Rnf14	ring finger protein 14	56736
1421857_at	1.544619803	8.187301057	11.27338726	2.83E-08	1.80E-06	Adam17	a disintegrin and metallopeptidase domain 17	11491
1419647_a_at	1.545325768	9.97650556	19.6543013	2.27E-11	1.42E-08	Ier3	immediate early response 3	15937
1427689_a_at	1.550871952	9.161424561	17.04449181	1.47E-10	4.83E-08	Tnip1	TNFAIP3 interacting protein 1	57783
1445522_at	1.555074498	6.538601556	15.789754	3.96E-10	9.36E-08	Atp8b4	ATPase, class I, type 8B, member 4	241633
1436555_at	1.556807211	6.748745722	14.35249439	1.35E-09	2.15E-07	Slc7a2	solute carrier family 7 (cationic amino acid transporter, y+ system), member 2	11988

1454880_s_at	1.557635927	6.418812863	8.657650705	6.75E-07	1.79E-05	Bmf	BCL2 modifying factor	171543
1449504_at	1.55827632	6.284413497	13.78091836	2.27E-09	3.01E-07	Kpna1	karyopherin (importin) alpha 1	16646
1415972_at	1.564254629	8.687135032	15.5788386	4.71E-10	1.06E-07	Marcks	myristoylated alanine rich protein kinase C substrate	17118
1450629_at	1.564569487	7.974998932	19.43220019	2.64E-11	1.55E-08	Lima1	LIM domain and actin binding 1	65970
1424067_at	1.566460219	9.226298281	15.07570785	7.19E-10	1.43E-07	Icam1	intercellular adhesion molecule 1	15894
1420088_at	1.567255047	11.28313153	19.24917526	2.99E-11	1.66E-08	Nfkbia	nuclear factor of kappa light polypeptide gene enhancer in B cells inhibitor, alpha	18035
1424826_s_at	1.572294155	8.519830306	14.44942777	1.24E-09	2.04E-07	Mtss1	metastasis suppressor 1	211401
1434745_at	1.575354354	6.163619258	13.34052701	3.43E-09	4.02E-07	Ccnd2	cyclin D2	12444
1427351_s_at	1.577600298	7.619632928	13.41131371	3.21E-09	3.88E-07	Ighm	immunoglobulin heavy constant mu	16019
1415871_at	1.582209209	10.04377613	12.91088595	5.19E-09	5.43E-07	Tgfbi	transforming growth factor, beta induced	21810
1449168_a_at	1.586108887	6.973977408	14.74288751	9.58E-10	1.72E-07	Akap2	A kinase (PRKA) anchor protein 2	11641
1430533_a_at	1.59477727	9.301833142	12.26647371	9.88E-09	8.62E-07	Ctnnb1	catenin (cadherin associated protein), beta 1	12387
1438157_s_at	1.59527849	10.63179892	20.54716988	1.27E-11	1.00E-08	Nfkbia	nuclear factor of kappa light polypeptide gene enhancer in B cells inhibitor, alpha	18035
1437513_a_at	1.596799776	8.594623687	13.45637441	3.07E-09	3.76E-07	Serinc1	serine incorporator 1	56442

1426441_at	1.599068617	5.991436415	19.69208217	2.22E-11	1.41E-08	Slc11a2	solute carrier family 11 (proton-coupled divalent metal ion transporters), member 2	18174
1450413_at	1.60315827	7.480015603	18.9732664	3.61E-11	1.92E-08	Pdgfb	platelet derived growth factor, B polypeptide	18591
1425603_at	1.606590753	6.692548993	20.46096313	1.34E-11	1.02E-08	Tmem176a	transmembrane protein 176A	66058
1430332_a_at	1.626903723	9.244159977	10.49426588	6.81E-08	3.42E-06	Gusb	glucuronidase, beta	110006
1452430_s_at	1.631371248	8.815412891	9.494421085	2.28E-07	8.01E-06	Srsf1	serine/arginine-rich splicing factor 1	110809
1447392_s_at	1.631741341	5.42585238	14.64595833	1.04E-09	1.82E-07	Cpd	carboxypeptidase D	12874
1421413_a_at	1.632227695	6.251459745	20.35221314	1.44E-11	1.06E-08	Pdlim5	PDZ and LIM domain 5	56376
1416881_at	1.633454444	9.032132044	16.16332138	2.92E-10	7.70E-08	Mcl1	myeloid cell leukemia sequence 1	17210
1433443_a_at	1.641796761	10.17432387	17.58209901	9.79E-11	3.62E-08	Hmgcs1	3-hydroxy-3-methylglutaryl-Coenzyme A synthase 1	208715
1430692_a_at	1.642814329	7.115170335	13.91973932	2.00E-09	2.77E-07	Sel1l	sel-1 suppressor of lin-12-like (C. elegans)	20338
1448185_at	1.646354455	8.82848337	19.23889945	3.01E-11	1.66E-08	Herpud1	homocysteine-inducible, endoplasmic reticulum stress-inducible, ubiquitin-like domain member 1	64209
1425686_at	1.647490439	8.261801835	17.77493509	8.49E-11	3.30E-08	Cflar	CASP8 and FADD-like apoptosis regulator	12633

1416390_at	1.648228657	6.961082749	14.99493705	7.70E-10	1.51E-07	Rcbtb2	regulator of chromosome condensation (RCC1) and BTB (POZ) domain containing protein 2	105670
1431686_a_at	1.649124472	5.110104396	12.86317725	5.44E-09	5.57E-07	Gmfb	glia maturation factor, beta	63985
1448175_at	1.650597794	9.539905306	19.429495	2.65E-11	1.55E-08	Ehd1	EH-domain containing 1	13660
1418004_a_at	1.650661702	9.900508459	19.8939668	1.94E-11	1.31E-08	Tmem176b	transmembrane protein 176B	65963
1452078_a_at	1.651746662	8.167165496	16.97411701	1.55E-10	4.99E-08	Slc11a2	solute carrier family 11 (proton-coupled divalent metal ion transporters), member 2	18174
1428750_at	1.652685661	7.21076959	11.97169348	1.34E-08	1.07E-06	Cdc42ep2	CDC42 effector protein (Rho GTPase binding) 2	104252
1456080_a_at	1.653053484	9.226265572	9.264044453	3.05E-07	9.92E-06	Serinc3	serine incorporator 3	26943
1448348_at	1.654097606	8.368600493	16.91374281	1.62E-10	5.13E-08	Caprin1	cell cycle associated protein 1	53872
1415993_at	1.654529616	9.940826959	18.61289692	4.65E-11	2.30E-08	Sqle	squalene epoxidase	20775
1428735_at	1.658250565	4.714060996	19.42552283	2.65E-11	1.55E-08	Cd69	CD69 antigen	12515
1418288_at	1.662269073	6.972681288	11.29667277	2.75E-08	1.76E-06	Lpin1	lipin 1	14245
1435877_at	1.663071883	5.274811446	12.40148602	8.61E-09	7.80E-07	Stk38l	serine/threonine kinase 38 like	232533
1434225_at	1.667464481	9.347603016	21.09244419	8.97E-12	8.25E-09	Swap70	SWA-70 protein	20947
1418219_at	1.669122577	7.042618517	15.29964186	5.95E-10	1.25E-07	Il15	interleukin 15	16168
1416012_at	1.673045247	7.154144454	19.59976176	2.36E-11	1.46E-08	Ehd1	EH-domain containing 1	13660
1417069_a_at	1.675088182	5.891372716	10.097902	1.09E-07	4.80E-06	Gmfb	glia maturation factor, beta	63985
1416593_at	1.676284068	8.61278565	11.27840876	2.81E-08	1.80E-06	Glrx	glutaredoxin	93692
1448183_a_at	1.685672197	6.823941363	10.69261301	5.42E-08	2.91E-06	Hif1a	hypoxia inducible factor 1, alpha subunit	15251

1417871_at	1.692399288	7.536839316	16.4994445	2.24E-10	6.64E-08	Hsd17b7	hydroxysteroid (17-beta) dehydrogenase 7	15490
1425863_a_at	1.694320779	6.429346334	15.23211594	6.29E-10	1.31E-07	Ptpro	protein tyrosine phosphatase, receptor type, O	19277
1426913_at	1.700518043	6.199398807	13.10967377	4.28E-09	4.74E-07	Lss	lanosterol synthase	16987
1425654_a_at	1.700983086	6.816769777	17.99350503	7.24E-11	2.95E-08	Lima1	LIM domain and actin binding 1	65970
1434980_at	1.703178103	8.013353806	20.78478182	1.09E-11	8.77E-09	Pik3r5	phosphoinositide-3-kinase, regulatory subunit 5, p101	320207
1433444_at	1.711383532	9.949519967	13.27827622	3.64E-09	4.22E-07	Hmgcs1	3-hydroxy-3-methylglutaryl-Coenzyme A synthase 1	208715
1419589_at	1.712240785	8.117402635	21.19209389	8.42E-12	8.02E-09	Cd93	CD93 antigen	17064
1417110_at	1.717411107	7.827130632	22.50606202	3.80E-12	4.63E-09	Man1a	mannosidase 1, alpha	17155
1417263_at	1.725033372	6.909444308	16.16458266	2.92E-10	7.70E-08	Ptgs2	prostaglandin-endoperoxide synthase 2	19225
1425714_a_at	1.727274118	7.219261287	16.15790589	2.94E-10	7.70E-08	Nfam1	Nfat activating molecule with ITAM motif 1	74039
1421922_at	1.728303342	7.119673124	20.41207456	1.38E-11	1.04E-08	Sh3bp5	SH3-domain binding protein 5 (BTK-associated)	24056
1421198_at	1.731525266	6.023264044	19.92878059	1.89E-11	1.30E-08	Itgav	integrin alpha V	16410
1451206_s_at	1.735066849	8.361288503	12.37814993	8.82E-09	7.96E-07	Cytip	cytohesin 1 interacting protein	227929
1451584_at	1.738903924	6.419029685	20.93125862	9.92E-12	8.71E-09	Havcr2	hepatitis A virus cellular receptor 2	171285

1449731_s_at	1.748461895	9.216305432	19.50034405	2.52E-11	1.54E-08	Nfkbia	nuclear factor of kappa light polypeptide gene enhancer in B cells inhibitor, alpha	18035
1418025_at	1.757805488	7.394237492	22.23195342	4.47E-12	5.04E-09	Bhlhe40	basic helix-loop-helix family, member e40	20893
1420610_at	1.76947507	6.270498193	14.6662929	1.02E-09	1.81E-07	Prkacb	protein kinase, cAMP dependent, catalytic, beta	18749
1427348_at	1.772267628	5.861949999	12.00738416	1.29E-08	1.04E-06	Zc3h12a	zinc finger CCCH type containing 12A	230738
1452214_at	1.77271737	9.298135875	21.44046504	7.22E-12	7.27E-09	Skil	SKI-like	20482
1417185_at	1.773166352	9.836986997	16.32140938	2.58E-10	7.20E-08	Ly6a	lymphocyte antigen 6 complex, locus A	110454
1448021_at	1.773558386	5.675797608	15.7866897	3.97E-10	9.36E-08	Fam46c	family with sequence similarity 46, member C	74645
1415823_at	1.781724936	9.520277216	10.73730072	5.15E-08	2.81E-06	Scd2	stearoyl-Coenzyme A desaturase 2	20250
1419607_at	1.790000415	9.521040839	18.58022797	4.76E-11	2.33E-08	Tnf	tumor necrosis factor	21926
1433445_x_at	1.79199912	10.05253145	17.65315541	9.29E-11	3.55E-08	Hmgcs1	3-hydroxy-3-methylglutaryl-Coenzyme A synthase 1	208715
AFFX-TransRecMur/X57349_5_at	1.813320454	4.26664734	12.81451315	5.70E-09	5.78E-07	Tfrc	transferrin receptor	22042
1418019_at	1.81402066	8.666260269	15.07863232	7.17E-10	1.43E-07	Cpd	carboxypeptidase D	12874
1421845_at	1.819330127	6.9691919	14.70672145	9.88E-10	1.76E-07	Golph3	golgi phosphoprotein 3	66629
1431691_a_at	1.832225794	7.263773261	15.51734077	4.95E-10	1.10E-07	Rab31	RAB31, member RAS oncogene family	106572

1421073_a_at	1.837479145	4.467316841	11.66242151	1.86E-08	1.36E-06	Ptger4	prostaglandin E receptor 4 (subtype EP4)	19219
1444402_at	1.841295428	6.699961381	22.442988	3.94E-12	4.68E-09	Zc3h12c	zinc finger CCCH type containing 12C	244871
1447849_s_at	1.850874993	7.195951491	13.22151086	3.84E-09	4.39E-07	Maf	avian musculoaponeurotic fibrosarcoma oncogene homolog	17132
1450383_at	1.86085162	7.692341262	23.34999184	2.33E-12	3.51E-09	Ldlr	low density lipoprotein receptor	16835
1424754_at	1.861490236	8.778775092	21.49699629	6.97E-12	7.27E-09	Ms4a7	membrane-spanning 4-domains, subfamily A, member 7	109225
1419721_at	1.898275915	7.25167948	18.14910825	6.47E-11	2.77E-08	Hcar2	hydroxycarboxylic acid receptor 2	80885
1458299_s_at	1.902455828	8.404498996	21.17176098	8.53E-12	8.02E-09	Nfkbie	nuclear factor of kappa light polypeptide gene enhancer in B cells inhibitor, epsilon	18037
1421923_at	1.909367439	8.10341102	18.33655599	5.65E-11	2.63E-08	Sh3bp5	SH3-domain binding protein 5 (BTK-associated)	24056
1435697_a_at	1.918240588	8.478875209	15.7618854	4.05E-10	9.41E-08	Cytip	cytohesin 1 interacting protein	227929
1448306_at	1.922966726	9.385405601	19.81549199	2.04E-11	1.35E-08	Nfkbia	nuclear factor of kappa light polypeptide gene enhancer in B cells inhibitor, alpha	18035
1416630_at	1.940220525	6.831192125	14.61513831	1.07E-09	1.84E-07	Id3	inhibitor of DNA binding 3	15903

1437865_at	1.949841858	6.21972733	17.34327764	1.17E-10	4.12E-08	Spata13	spermatogenesis associated 13	219140
1422966_a_at	1.954009742	7.269530121	23.28949875	2.41E-12	3.51E-09	Tfrc	transferrin receptor	22042
1456212_x_at	1.963366673	6.800605629	18.22692971	6.12E-11	2.74E-08	Socs3	suppressor of cytokine signaling 3	12702
1418020_s_at	1.983949528	8.127975396	14.10439636	1.69E-09	2.47E-07	Cpd	carboxypeptidase D	12874
1426519_at	1.985466836	6.558984571	20.83691798	1.05E-11	8.71E-09	P4ha1	procollagen-proline, 2-oxoglutarate 4-dioxygenase (proline 4-hydroxylase), alpha 1 polypeptide	18451
1456060_at	1.988538622	8.767396572	12.31430758	9.41E-09	8.34E-07	Maf	avian musculoaponeurotic fibrosarcoma oncogene homolog	17132
1429954_at	1.990216003	7.270747499	16.95242449	1.57E-10	5.04E-08	Clec4a3	C-type lectin domain family 4, member a3	73149
AFFX-TransRecMur/X57349_3_at	1.993412319	6.157028152	22.52476621	3.76E-12	4.63E-09	Tfrc	transferrin receptor	22042
1418509_at	2.001502592	6.807789536	18.67292761	4.46E-11	2.26E-08	Cbr2	carbonyl reductase 2	12409
1423311_s_at	2.00965193	5.713574049	23.54022933	2.09E-12	3.33E-09	Tpbg	trophoblast glycoprotein	21983
1453550_a_at	2.043604672	6.757622365	14.26310081	1.46E-09	2.27E-07	Far1	fatty acyl CoA reductase 1	67420
1450646_at	2.060109766	8.158467335	25.80160202	6.18E-13	1.74E-09	Cyp51	cytochrome P450, family 51	13121
1423078_a_at	2.062884592	9.737611378	24.2799431	1.39E-12	2.50E-09	Msmo1	methylsterol monoxygenase 1	66234
1415971_at	2.069453603	9.597610982	25.21506395	8.39E-13	2.06E-09	Marcks	myristoylated alanine rich protein kinase C substrate	17118

1416654_at	2.073049329	8.639152528	20.46943773	1.33E-11	1.02E-08	Slc31a2	solute carrier family 31, member 2	20530
1455870_at	2.085003322	6.947440341	16.81845019	1.75E-10	5.43E-08	Akap2	A kinase (PRKA) anchor protein 2	11641
1424208_at	2.090332869	7.705104914	22.8403965	3.13E-12	4.41E-09	Ptger4	prostaglandin E receptor 4 (subtype EP4)	19219
1419132_at	2.165024236	10.23204023	24.46247251	1.26E-12	2.36E-09	Tlr2	toll-like receptor 2	24088
1422862_at	2.17465319	5.037330477	12.21621301	1.04E-08	8.94E-07	Pdlim5	PDZ and LIM domain 5	56376
1423310_at	2.190724437	5.079903519	24.6923958	1.11E-12	2.27E-09	Tpbg	trophoblast glycoprotein	21983
1418480_at	2.190961295	4.642837836	25.60317789	6.85E-13	1.82E-09	Ppbp	pro-platelet basic protein	57349
1423543_at	2.191023866	9.065896144	24.08112127	1.55E-12	2.69E-09	Swap70	SWA-70 protein	20947
1432344_a_at	2.201269856	7.118939452	11.50300443	2.20E-08	1.53E-06	Aplp2	amyloid beta (A4) precursor-like protein 2	11804
1419532_at	2.209159155	8.293540199	19.12629141	3.25E-11	1.75E-08	Il1r2	interleukin 1 receptor, type II	16178
1460251_at	2.210984908	6.850521862	21.47843189	7.05E-12	7.27E-09	Fas	Fas (TNF receptor superfamily member 6)	14102
1452160_at	2.212196165	6.636306993	16.72714616	1.87E-10	5.75E-08	Tiparp	TCDD-inducible poly(ADP-ribose) polymerase	99929
1422533_at	2.215380017	6.755059656	18.7652187	4.18E-11	2.17E-08	Cyp51	cytochrome P450, family 51	13121
1436538_at	2.216862937	7.03539337	20.823366	1.06E-11	8.71E-09	Ankrd37	ankyrin repeat domain 37	654824
1434302_at	2.24437191	7.371797832	23.50026168	2.14E-12	3.33E-09	Raph1	Ras association (RalGDS/AF-6) and pleckstrin homology domains 1	77300
1453721_a_at	2.244904098	8.023865146	27.949421	2.12E-13	1.06E-09	Slc31a2	solute carrier family 31, member 2	20530

1430984_at	2.261984574	6.366910718	20.08339954	1.71E-11	1.21E-08	Azin1	antizyme inhibitor 1	54375
1423602_at	2.297293267	6.238864093	14.50911291	1.18E-09	1.95E-07	Traf1	TNF receptor-associated factor 1	22029
1456046_at	2.363655597	8.718742952	31.0282879	5.24E-14	7.88E-10	Cd93	CD93 antigen	17064
1422190_at	2.370670769	9.336466676	15.817097	3.87E-10	9.28E-08	C5ar1	complement component 5a receptor 1	12273
1433699_at	2.376276905	6.916660881	27.28393635	2.93E-13	1.32E-09	Tnfaip3	tumor necrosis factor, alpha-induced protein 3	21929
1455899_x_at	2.380996358	7.600295764	29.6931539	9.45E-14	8.00E-10	Socs3	suppressor of cytokine signaling 3	12702
1429239_a_at	2.381163369	7.299923607	21.36612247	7.56E-12	7.41E-09	Stard4	StAR-related lipid transfer (START) domain containing 4	170459
1454656_at	2.381582119	7.069381587	24.95497552	9.64E-13	2.14E-09	Spata13	spermatogenesis associated 13	219140
1434520_at	2.400574281	5.942498326	26.28125352	4.83E-13	1.56E-09	Sc5d	sterol-C5-desaturase	235293
1448728_a_at	2.402067285	8.051186622	25.15568427	8.66E-13	2.06E-09	Nfkbiz	nuclear factor of kappa light polypeptide gene enhancer in B cells inhibitor, zeta	80859
1422967_a_at	2.427431484	5.56525759	21.43249248	7.26E-12	7.27E-09	Tfrc	transferrin receptor	22042
1458802_at	2.431223579	6.284410073	17.21437026	1.29E-10	4.41E-08	Hivep3	human immunodeficiency virus type I enhancer binding protein 3	16656
1416111_at	2.493327329	6.926137839	26.52986894	4.26E-13	1.48E-09	Cd83	CD83 antigen	12522
1433446_at	2.501948675	8.80222315	18.21976093	6.15E-11	2.74E-08	Hmgcs1	3-hydroxy-3-methylglutaryl-Coenzyme A synthase 1	208715

1417483_at	2.605932118	9.202823621	29.43160342	1.06E-13	8.00E-10	Nfkbiz	nuclear factor of kappa light polypeptide gene enhancer in B cells inhibitor, zeta	80859
1428034_a_at	2.777497892	5.712199247	24.89375699	9.96E-13	2.14E-09	Tnfrsf9	tumor necrosis factor receptor superfamily, member 9	21942
1417256_at	2.973249753	5.645352694	29.54533477	1.01E-13	8.00E-10	Mmp13	matrix metallopeptidase 13	17386
1437025_at	4.043393244	6.86014192	35.56135383	8.40E-15	3.79E-10	Cd28	CD28 antigen	12487
1417597_at	5.464468898	7.275457054	28.81834225	1.41E-13	8.22E-10	Cd28	CD28 antigen	12487

Down-regulated Probes or Genes

PROBEID	logFC	AveExpr	t	P.Value	adj.P.Val	SYMBOL	GENENAME	ENTREZID
1450297_at	-2.582753486	5.083688067	-32.19266049	3.20E-14	7.22E-10	Il6	interleukin 6	16193
1418776_at	-3.006902193	5.803166188	-28.74727281	1.46E-13	8.22E-10	Gbp8	guanylate-binding protein 8	76074
1437325_x_at	-2.706900575	7.439258568	-26.62783462	4.06E-13	1.48E-09	Aldh18a1	aldehyde dehydrogenase 18 family, member A1	56454
1423100_at	-2.568803732	8.870468671	-26.7994927	3.72E-13	1.48E-09	Fos	FBJ osteosarcoma oncogene	14281
1429216_at	-2.09221922	5.107414696	-25.95648937	5.70E-13	1.72E-09	Paqr3	progestin and adipoQ receptor family member III	231474
1457687_at	-1.817990888	7.339563446	-23.99428922	1.62E-12	2.71E-09	Bcl2	B cell leukemia/lymphoma 2	12043
1440226_at	-1.682712855	5.368734227	-22.77880365	3.24E-12	4.43E-09	Zfp760	zinc finger protein 760	240034
1452157_at	-1.901479456	9.221315839	-22.5189574	3.77E-12	4.63E-09	Eprs	glutamyl-prolyl-tRNA synthetase	107508
1456225_x_at	-2.086048995	8.532042459	-22.36604285	4.13E-12	4.77E-09	Trib3	tribbles pseudokinase 3	228775

1426243_at	-1.595753564	9.531375631	-21.56983915	6.67E-12	7.27E-09	Cth	cystathionase (cystathionine gamma-lyase)	107869
1420411_a_at	-1.797969505	7.106898243	-21.01683782	9.40E-12	8.48E-09	Pi4k2b	phosphatidylinositol 4-kinase type 2 beta	67073
1426065_a_at	-2.172354025	8.15940621	-20.85168681	1.04E-11	8.71E-09	Trib3	tribbles pseudokinase 3	228775
1426015_s_at	-1.678059946	6.298816301	-20.85725615	1.04E-11	8.71E-09	Asph	aspartate-beta-hydroxylase	65973
1415836_at	-1.650539759	6.630990707	-20.13846173	1.65E-11	1.18E-08	Aldh18a1	aldehyde dehydrogenase 18 family, member A1	56454
1435432_at	-1.724354921	6.761294316	-19.92755248	1.90E-11	1.30E-08	Agap1	ArfGAP with GTPase domain, ankyrin repeat and PH domain 1	347722
1419254_at	-2.274099136	8.950477555	-19.72813231	2.16E-11	1.39E-08	Mthfd2	methylenetetrahydrofolate dehydrogenase (NAD+ dependent), methenyltetrahydrofolate cyclohydrolase	17768
1425615_a_at	-1.903285026	8.320112202	-19.3963687	2.71E-11	1.56E-08	Pck2	phosphoenolpyruvate carboxykinase 2 (mitochondrial)	74551
1419247_at	-1.809883187	8.303031166	-19.37184359	2.75E-11	1.57E-08	Rgs2	regulator of G-protein signaling 2	19735
1434709_at	-1.458487385	7.070425115	-19.17087337	3.15E-11	1.71E-08	Nrcam	neuronal cell adhesion molecule	319504
1416690_at	-1.422120336	8.472305353	-18.85625995	3.92E-11	2.06E-08	Gtpbp2	GTP binding protein 2	56055
1434129_s_at	-1.727452583	6.625945383	-18.67748245	4.44E-11	2.26E-08	Lhfpl2	lipoma HMGIC fusion partner-like 2	218454

1440344_at	-1.875531639	5.034197305	-18.37824524	5.49E-11	2.61E-08	Snord89	small nucleolar RNA, C/D box 89	100217461
1419248_at	-1.863365793	7.664301958	-18.38782866	5.45E-11	2.61E-08	Rgs2	regulator of G-protein signaling 2	19735
1456150_at	-1.475152225	6.8491407	-18.31909345	5.72E-11	2.63E-08	Kdm7a	lysine (K)-specific demethylase 7A	338523
1455007_s_at	-1.716756856	5.609606979	-18.15529265	6.44E-11	2.77E-08	Gpt2	glutamic pyruvate transaminase (alanine aminotransferase) 2	108682
1455084_x_at	-1.630135846	9.95450067	-18.14139534	6.50E-11	2.77E-08	Shmt2	serine hydroxymethyltransferase 2 (mitochondrial)	108037
1438130_at	-1.906186068	4.639323221	-17.99085987	7.25E-11	2.95E-08	Taf15	TATA-box binding protein associated factor 15	70439
1428083_at	-1.752057415	8.817055142	-17.98953578	7.26E-11	2.95E-08	Neat1	nuclear paraspeckle assembly transcript 1 (non-protein coding)	66961
1420499_at	-1.629710694	7.876290216	-17.90148807	7.74E-11	3.12E-08	Gch1	GTP cyclohydrolase 1	14528
1433769_at	-1.401887424	6.739392049	-17.80495001	8.31E-11	3.29E-08	Als2cl	ALS2 C-terminal like	235633
1434384_at	-1.597864979	6.017921525	-17.73803609	8.72E-11	3.36E-08	Nrip1	nuclear receptor interacting protein 1	268903
1448890_at	-1.903372505	6.553298477	-17.61116969	9.58E-11	3.57E-08	Klf2	Kruppel-like factor 2 (lung)	16598
1436303_at	-1.327376995	6.504664714	-17.61705596	9.54E-11	3.57E-08	Afdn	afadin, adherens junction formation factor	17356
1421732_at	-1.425036296	7.244933696	-17.51527913	1.03E-10	3.77E-08	Glrp1	glutamine repeat protein 1	14659
1423429_at	-1.759206469	6.2445101	-17.29938077	1.21E-10	4.23E-08	Rhox5	reproductive homeobox 5	18617
1416101_a_at	-1.655861435	9.293163939	-17.2250133	1.28E-10	4.40E-08	Hist1h1c	histone cluster 1, H1c	50708

1417516_at	-1.694850011	8.1368029	-17.1336369	1.37E-10	4.62E-08	Ddit3	DNA-damage inducible transcript 3	13198
1436195_at	-1.37889023	6.201515094	-17.08846601	1.42E-10	4.71E-08	Lrrc75a	leucine rich repeat containing 75A	192976
1433460_at	-1.378682122	7.51772062	-16.90979268	1.63E-10	5.13E-08	Ttc7b	tetratricopeptide repeat domain 7B	104718
1436210_at	-1.580541461	5.978787438	-16.68274637	1.94E-10	5.91E-08	Gk5	glycerol kinase 5 (putative)	235533
1426530_a_at	-1.370645722	7.751502675	-16.65520563	1.98E-10	6.00E-08	Klhl5	kelch-like 5	71778
1434241_at	-1.424519804	8.168083655	-16.54456378	2.16E-10	6.50E-08	Tbc1d31	TBC1 domain family, member 31	210544
1452158_at	-1.458581574	9.230175473	-16.48193721	2.27E-10	6.69E-08	Eprs	glutamyl-prolyl-tRNA synthetase	107508
1426713_s_at	-1.480498353	9.39499102	-16.40994292	2.40E-10	6.86E-08	Eprs	glutamyl-prolyl-tRNA synthetase	107508
1423784_at	-1.209362173	9.984453404	-16.36239972	2.49E-10	7.03E-08	Gars	glycyl-tRNA synthetase	353172
1436421_s_at	-1.301609148	8.400393676	-16.28935834	2.64E-10	7.27E-08	Arpc5l	actin related protein 2/3 complex, subunit 5-like	74192
1417177_at	-1.333335526	7.748057903	-16.18509039	2.87E-10	7.70E-08	Galk1	galactokinase 1	14635
1452679_at	-1.46248806	5.469831234	-16.09511393	3.09E-10	8.05E-08	Tubb2b	tubulin, beta 2B class IIB	73710
1415834_at	-1.609068226	8.969092899	-15.93087778	3.53E-10	8.99E-08	Dusp6	dual specificity phosphatase 6	67603
1456439_x_at	-1.550965128	6.362801391	-15.89082922	3.64E-10	9.13E-08	Mical1	microtubule associated monooxygenase, calponin and LIM domain containing 1	171580
1416378_at	-1.336070266	7.212729962	-15.89177162	3.64E-10	9.13E-08	Pnkp	polynucleotide kinase 3'-phosphatase	59047
1423626_at	-1.687221922	6.616474407	-15.86872657	3.71E-10	9.24E-08	Dst	dystonin	13518
1423534_at	-1.374285758	8.092269685	-15.85972593	3.74E-10	9.26E-08	Pdcd2	programmed cell death 2	18567

1428797_at	-1.356661523	6.629920186	-15.85278608	3.76E-10	9.26E-08	Setd6	SET domain containing 6	66083
1426462_at	-1.222827738	5.795225514	-15.82553721	3.84E-10	9.27E-08	Gphn	gephyrin	268566
1455246_at	-1.45947785	7.265927971	-15.76616775	4.03E-10	9.41E-08	Smarcc1	SWI/SNF related, matrix associated, actin dependent regulator of chromatin, subfamily c, member 1	20588
1442886_at	-1.423956135	5.333187407	-15.63514909	4.49E-10	1.03E-07	Tra2a	transformer 2 alpha homolog (Drosophila)	101214
1424413_at	-1.172417081	7.242282249	-15.60417811	4.61E-10	1.05E-07	Ogfrl1	opioid growth factor receptor-like 1	70155
1427342_at	-1.309076963	6.741473172	-15.54200225	4.85E-10	1.08E-07	Fastkd1	FAST kinase domains 1	320720
1448558_a_at	-1.189841787	10.07396456	-15.55770615	4.79E-10	1.08E-07	Pla2g4a	phospholipase A2, group IVA (cytosolic, calcium-dependent)	18783
1437122_at	-1.515633406	6.371585746	-15.50433243	5.01E-10	1.10E-07	Bcl2	B cell leukemia/lymphoma 2	12043
1417976_at	-1.350852247	6.704322524	-15.51059596	4.98E-10	1.10E-07	Ada	adenosine deaminase	11486
1434907_at	-1.150959606	7.150421799	-15.46976496	5.16E-10	1.12E-07	Tbpl1	TATA box binding protein-like 1	237336
1452210_at	-1.231069126	7.489960997	-15.40749049	5.43E-10	1.17E-07	Dna2	DNA replication helicase/nuclease 2	327762
1442058_s_at	-1.175846247	7.263030827	-15.37877611	5.56E-10	1.19E-07	Psmc3ip	proteasome (prosome, macropain) 26S subunit, ATPase 3, interacting protein	19183
1451512_s_at	-1.562257812	6.880772129	-15.30993216	5.89E-10	1.25E-07	Hibch	3-hydroxyisobutyryl-Coenzyme A hydrolase	227095

1452690_at	-1.29223214	7.161532609	-15.2232024	6.34E-10	1.31E-07	Khsrp	KH-type splicing regulatory protein	16549
1448274_at	-1.185724463	10.02246781	-15.20945324	6.42E-10	1.32E-07	C1qbp	complement component 1, q subcomponent binding protein	12261
1429642_at	-1.716803094	5.1258492	-15.01882263	7.55E-10	1.49E-07	Zfand4	zinc finger, AN1-type domain 4	67492
1423701_at	-1.290219961	7.441045634	-14.96724099	7.89E-10	1.53E-07	Coasy	Coenzyme A synthase	71743
1421276_a_at	-1.366892915	5.627407932	-14.93744173	8.09E-10	1.55E-07	Dst	dystonin	13518
1420502_at	-1.206808073	8.296583532	-14.94989402	8.01E-10	1.55E-07	Sat1	spermidine/spermine N1-acetyl transferase 1	20229
1447830_s_at	-1.820551429	7.05423755	-14.89439212	8.40E-10	1.58E-07	Rgs2	regulator of G-protein signaling 2	19735
1424986_s_at	-1.652148422	6.209237809	-14.87929197	8.51E-10	1.59E-07	Fbxw7	F-box and WD-40 domain protein 7	50754
1450943_at	-1.515281182	6.241029223	-14.81974536	8.96E-10	1.66E-07	Magohb	mago homolog B, exon junction complex core component	66441
1433570_s_at	-1.133780722	5.598785232	-14.82315942	8.93E-10	1.66E-07	Naa35	N(alpha)-acetyltransferase 35, NatC auxiliary subunit	78689
1415694_at	-1.165861308	8.218586964	-14.8084966	9.05E-10	1.67E-07	Wars	tryptophanyl-tRNA synthetase	22375
1419253_at	-1.486818361	8.591729921	-14.77070618	9.35E-10	1.70E-07	Mthfd2	methylenetetrahydrofolate dehydrogenase (NAD+ dependent), methenyltetrahydrofolate cyclohydrolase	17768

1428897_at	-1.26573588	7.092295783	-14.75058362	9.51E-10	1.72E-07	Tstd3	thiosulfate sulfurtransferase (rhodanese)-like domain containing 3	77032
1424396_a_at	-1.166736512	7.641614678	-14.74887069	9.53E-10	1.72E-07	Asrgl1	asparaginase like 1	66514
1416616_s_at	-1.236633477	8.183371813	-14.71623276	9.80E-10	1.75E-07	Clpp	caseinolytic mitochondrial matrix peptidase proteolytic subunit	53895
1434695_at	-1.171485761	7.127084092	-14.63982203	1.05E-09	1.82E-07	Dtl	denticleless E3 ubiquitin protein ligase	76843
1439272_at	-1.528003833	6.266127561	-14.63444299	1.05E-09	1.83E-07	Lcorl	ligand dependent nuclear receptor corepressor-like	209707
1427564_at	-1.635873812	5.404512378	-14.60145447	1.08E-09	1.86E-07	Diaph2	diaphanous related formin 2	54004
1449841_at	-1.256284614	7.08881246	-14.57166778	1.11E-09	1.90E-07	Kif3a	kinesin family member 3A	16568
1449020_at	-1.134495004	7.812636829	-14.54777236	1.14E-09	1.93E-07	Plscr3	phospholipid scramblase 3	70310
1438480_a_at	-1.228098158	8.514854477	-14.52089757	1.16E-09	1.95E-07	Thyn1	thymocyte nuclear protein 1	77862
1415936_at	-1.271692439	8.018130448	-14.39574823	1.30E-09	2.11E-07	Bcar3	breast cancer anti-estrogen resistance 3	29815
1433640_at	-1.35262067	5.7866673	-14.34477415	1.36E-09	2.15E-07	Fubp1	far upstream element (FUSE) binding protein 1	51886
1424431_at	-1.089116814	8.838966165	-14.35042643	1.35E-09	2.15E-07	Csgalnact2	chondroitin sulfate N-acetylgalactosaminyltransfer ase 2	78752
1418396_at	-1.208238625	7.639453827	-14.33672346	1.37E-09	2.16E-07	Gpsm3	G-protein signalling modulator 3 (AGS3-like, C. elegans)	106512

1434604_at	-1.288626792	8.071882617	-14.30780543	1.41E-09	2.21E-07	Eif5b	eukaryotic translation initiation factor 5B	226982
1416926_at	-1.094373064	7.00825259	-14.30001968	1.42E-09	2.22E-07	Trp53inp1	transformation related protein 53 inducible nuclear protein 1	60599
1417395_at	-1.262766561	7.377414354	-14.24842003	1.48E-09	2.27E-07	Klf4	Kruppel-like factor 4 (gut)	16600
1451012_a_at	-1.423756921	9.47980657	-14.18683634	1.57E-09	2.39E-07	Ybx3	Y box protein 3	56449
1434554_at	-1.395719869	5.743680195	-14.18221149	1.57E-09	2.39E-07	Trim37	tripartite motif-containing 37	68729
1452351_at	-1.530702716	5.987272271	-14.16583842	1.60E-09	2.42E-07	Parp4	poly (ADP-ribose) polymerase family, member 4	328417
1423157_at	-1.424429021	6.09171673	-14.15951585	1.61E-09	2.42E-07	Gnpnat1	glucosamine-phosphate N-acetyltransferase 1	54342
1428449_at	-1.224768724	5.604841889	-14.15748452	1.61E-09	2.42E-07	Gtf3c2	general transcription factor IIIC, polypeptide 2, beta	71752
1448403_at	-1.209946499	9.509518674	-14.13058507	1.65E-09	2.44E-07	Lars	leucyl-tRNA synthetase	107045
1424877_a_at	-1.122981926	7.788441439	-14.14040727	1.63E-09	2.44E-07	Alad	aminolevulinate, delta-, dehydratase	17025
1434401_at	-1.045346255	6.895795838	-14.1295536	1.65E-09	2.44E-07	Zcchc2	zinc finger, CCHC domain containing 2	227449
1455686_at	-1.107629641	5.917819663	-14.10362858	1.69E-09	2.47E-07	Lcorl	ligand dependent nuclear receptor corepressor-like	209707
1435372_a_at	-1.148843541	9.71763856	-14.07277882	1.74E-09	2.52E-07	Pa2g4	proliferation-associated 2G4	18813
1424532_at	-1.057832883	7.692959813	-14.06250958	1.75E-09	2.53E-07	Ylpm1	YLP motif containing 1	56531
1422439_a_at	-1.192688215	8.282104638	-14.03712805	1.79E-09	2.57E-07	Cdk4	cyclin-dependent kinase 4	12567
1416489_at	-1.325507218	7.143486339	-14.00279804	1.85E-09	2.63E-07	Pi4k2b	phosphatidylinositol 4-kinase type 2 beta	67073

1418015_at	-1.266957277	7.749091156	-14.00612848	1.85E-09	2.63E-07	Pum2	pumilio RNA-binding family member 2	80913
1419286_s_at	-1.16635126	5.587214293	-14.00489862	1.85E-09	2.63E-07	Ift81	intraflagellar transport 81	12589
1419572_a_at	-1.120149566	6.152941963	-13.97912325	1.89E-09	2.67E-07	Abcd4	ATP-binding cassette, sub-family D (ALD), member 4	19300
1449519_at	-1.439535315	7.864540578	-13.9407702	1.96E-09	2.73E-07	Gadd45a	growth arrest and DNA-damage-inducible 45 alpha	13197
1448325_at	-1.759600105	7.830117219	-13.90190457	2.03E-09	2.80E-07	Ppp1r15a	protein phosphatase 1, regulatory (inhibitor) subunit 15A	17872
1438094_x_at	-1.189872539	8.63423647	-13.89877113	2.04E-09	2.80E-07	Ola1	Obg-like ATPase 1	67059
1435433_at	-1.155319119	6.053945718	-13.90518731	2.02E-09	2.80E-07	Agap1	ArfGAP with GTPase domain, ankyrin repeat and PH domain 1	347722
1417667_a_at	-1.079458073	7.575388823	-13.8268908	2.18E-09	2.95E-07	Pter	phosphotriesterase related	19212
1424615_at	-1.318575721	7.877397034	-13.81462624	2.20E-09	2.96E-07	Pgap2	post-GPI attachment to proteins 2	233575
1422653_at	-1.195577558	6.506878641	-13.81026502	2.21E-09	2.97E-07	Cep70	centrosomal protein 70	68121
1455696_a_at	-1.299700045	6.251082514	-13.78237891	2.27E-09	3.01E-07	Prpf4b	pre-mRNA processing factor 4B	19134
1450916_at	-1.190904586	6.982526735	-13.79043854	2.25E-09	3.01E-07	Stau2	staufen (RNA binding protein) homolog 2 (Drosophila)	29819
1438161_s_at	-1.226868691	7.50383544	-13.76846661	2.30E-09	3.04E-07	Rfc4	replication factor C (activator 1) 4	106344
1428796_at	-1.355492074	6.753999766	-13.73521329	2.37E-09	3.12E-07	Bbx	bobby sox homolog (Drosophila)	70508

1438192_s_at	-1.12925667	7.422859992	-13.72694728	2.39E-09	3.14E-07	Baz2a	bromodomain adjacent to zinc finger domain, 2A	116848
1439017_x_at	-1.087412373	9.684589707	-13.70985842	2.42E-09	3.18E-07	Adipor1	adiponectin receptor 1	72674
1449626_s_at	-1.285830818	6.83443166	-13.65076062	2.56E-09	3.34E-07	Acbd4	acyl-Coenzyme A binding domain containing 4	67131
1428909_at	-1.4247599	7.013643214	-13.63741101	2.59E-09	3.37E-07	A130040M12Rik	RIKEN cDNA A130040M12 gene	319269
1417695_a_at	-1.062409659	9.272468095	-13.60230174	2.68E-09	3.45E-07	Soat1	sterol O-acyltransferase 1	20652
1424211_at	-1.180514864	6.965241464	-13.58407617	2.73E-09	3.49E-07	Slc25a33	solute carrier family 25, member 33	70556
1417134_at	-1.104445508	7.096365292	-13.58453427	2.72E-09	3.49E-07	Srpk2	serine/arginine-rich protein specific kinase 2	20817
1436420_a_at	-1.16336197	7.366190509	-13.5743633	2.75E-09	3.51E-07	Ipo4	importin 4	75751
1450897_at	-1.135725623	7.497631688	-13.56860187	2.77E-09	3.52E-07	Arhgap5	Rho GTPase activating protein 5	11855
1448748_at	-1.098401873	10.67388322	-13.56335756	2.78E-09	3.53E-07	Plek	pleckstrin	56193
1460692_at	-1.024922646	8.49539335	-13.55912685	2.79E-09	3.53E-07	Ehmt2	euchromatic histone lysine N-methyltransferase 2	110147
1415792_at	-1.047129072	7.816782202	-13.54331465	2.83E-09	3.57E-07	Rbck1	RanBP-type and C3HC4-type zinc finger containing 1	24105
1436572_at	-1.064610586	5.405761557	-13.51666878	2.90E-09	3.63E-07	Cep95	centrosomal protein 95	320162
1452830_s_at	-1.432855275	6.728214348	-13.50888033	2.92E-09	3.64E-07	Cad	carbamoyl-phosphate synthetase 2, aspartate transcarbamylase, and dihydroorotase	69719
1451053_a_at	-1.099718132	5.66369305	-13.50831657	2.93E-09	3.64E-07	Mdm1	transformed mouse 3T3 cell double minute 1	17245

1424144_at	-1.01367898	8.304134903	-13.50526543	2.93E-09	3.64E-07	Cdt1	chromatin licensing and DNA replication factor 1	67177
1416759_at	-1.728972566	5.755540693	-13.47485838	3.02E-09	3.70E-07	Mical1	microtubule associated monooxygenase, calponin and LIM domain containing 1	171580
1424306_at	-1.352618055	6.415921585	-13.42147292	3.18E-09	3.85E-07	Elovl4	elongation of very long chain fatty acids (FEN1/Elo2, SUR4/Elo3, yeast)-like 4	83603
1417586_at	-1.325654668	6.882613976	-13.40102493	3.24E-09	3.89E-07	Timeless	timeless circadian clock 1	21853
1425577_at	-1.210694331	5.61035765	-13.40595406	3.22E-09	3.89E-07	Zmym5	zinc finger, MYM-type 5	219105
1422508_at	-1.004846435	9.898396436	-13.3991146	3.24E-09	3.89E-07	Atp6v1a	ATPase, H+ transporting, lysosomal V1 subunit A	11964
1448691_at	-1.040678627	7.630735711	-13.39462064	3.26E-09	3.90E-07	Ubqln4	ubiquilin 4	94232
1416915_at	-1.187587697	7.721320793	-13.36763862	3.34E-09	3.96E-07	Msh6	mutS homolog 6	17688
1460711_at	-1.147668857	7.391394337	-13.36677259	3.34E-09	3.96E-07	Dnajc21	DnaJ heat shock protein family (Hsp40) member C21	78244
1434353_at	-1.057295162	6.399379143	-13.3632295	3.36E-09	3.96E-07	Sfmbt2	Scm-like with four mbt domains 2	353282
1452599_s_at	-1.050762583	8.017775144	-13.34993629	3.40E-09	4.00E-07	AI413582	expressed sequence AI413582	106672
1453111_a_at	-1.082513658	9.164221607	-13.33478138	3.45E-09	4.03E-07	Slc25a39	solute carrier family 25, member 39	68066
1452620_at	-1.565167	5.712743701	-13.27398646	3.65E-09	4.23E-07	Pck2	phosphoenolpyruvate carboxykinase 2 (mitochondrial)	74551

1426998_at	-1.24861826	7.200390843	-13.26866748	3.67E-09	4.23E-07	Zfand3	zinc finger, AN1-type domain 3	21769
1436767_at	-1.18265576	6.158673855	-13.26826061	3.67E-09	4.23E-07	Luc7l2	LUC7-like 2 (S. cerevisiae)	192196
1417038_at	-1.018222382	7.640580514	-13.24832445	3.74E-09	4.30E-07	9-Sep	septin 9	53860
1438659_x_at	-1.224682025	7.333784861	-13.21738995	3.86E-09	4.39E-07	Chchd6	coiled-coil-helix-coiled-coil-helix domain containing 6	66098
1449008_at	-1.164230204	6.929560249	-13.20286211	3.91E-09	4.43E-07	Tulp3	tubby-like protein 3	22158
1460643_at	-1.098901641	6.380645093	-13.1583257	4.08E-09	4.60E-07	Ell	elongation factor RNA polymerase II	13716
1437072_at	-1.321521237	7.182689998	-13.13692657	4.17E-09	4.67E-07	Arhgap25	Rho GTPase activating protein 25	232201
1424101_at	-1.089084636	7.813048354	-13.13624793	4.17E-09	4.67E-07	Hnrnpl	heterogeneous nuclear ribonucleoprotein L	15388
1424629_at	-1.299242661	6.94640871	-13.11854561	4.24E-09	4.72E-07	Brca1	breast cancer 1, early onset	12189
1426735_at	-1.033134526	7.582367487	-13.09385169	4.34E-09	4.80E-07	Iars2	isoleucine-tRNA synthetase 2, mitochondrial	381314
1418530_at	-1.076086882	9.070563846	-13.07263037	4.43E-09	4.89E-07	Nup160	nucleoporin 160	59015
1451525_at	-1.01564867	7.179460573	-13.06948069	4.45E-09	4.89E-07	Arhgap12	Rho GTPase activating protein 12	75415
1426323_x_at	-1.299143493	7.791931767	-13.05682217	4.50E-09	4.92E-07	Siva1	SIVA1, apoptosis-inducing factor	30954
1422704_at	-1.053155165	8.928576378	-13.05360546	4.52E-09	4.92E-07	Gk	glycerol kinase	14933
1417351_a_at	-1.020253979	9.36047968	-13.0579749	4.50E-09	4.92E-07	Snrpa1	small nuclear ribonucleoprotein polypeptide A'	68981

1449217_at	-1.10391021	7.758722084	-13.04900119	4.54E-09	4.93E-07	Casp8ap2	caspase 8 associated protein 2	26885
1451382_at	-1.411976465	7.180865409	-13.02956386	4.62E-09	5.01E-07	Chac1	ChaC, cation transport regulator 1	69065
1447936_at	-1.336274577	8.069609257	-13.02277352	4.65E-09	5.02E-07	2410006H16Rik	RIKEN cDNA 2410006H16 gene	69221
1451308_at	-1.117040328	6.484207206	-13.01941413	4.67E-09	5.03E-07	Elovl4	elongation of very long chain fatty acids (FEN1/Elo2, SUR4/Elo3, yeast)-like 4	83603
1456614_at	-1.179360194	6.778580117	-13.01007964	4.71E-09	5.05E-07	Sdhaf3	succinate dehydrogenase complex assembly factor 3	71238
1434855_at	-1.317411926	8.075605609	-12.99178587	4.80E-09	5.11E-07	Amdhd2	amidohydrolase domain containing 2	245847
1426992_at	-1.022732054	7.875805114	-12.99246981	4.79E-09	5.11E-07	Xpr1	xenotropic and polytropic retrovirus receptor 1	19775
1438143_s_at	-1.017181931	6.625761226	-12.97221186	4.89E-09	5.18E-07	Atxn2	ataxin 2	20239
1452014_a_at	-1.134218993	8.338992019	-12.90744066	5.21E-09	5.44E-07	Igf1	insulin-like growth factor 1	16000
1451974_at	-1.185525417	8.2653174	-12.88594762	5.32E-09	5.49E-07	Osbpl2	oxysterol binding protein-like 2	228983
1455608_at	-1.179549901	5.615913864	-12.88643303	5.32E-09	5.49E-07	Sclt1	sodium channel and clathrin linker 1	67161
1419833_s_at	-1.120668553	7.13767569	-12.87918795	5.35E-09	5.51E-07	Arap3	ArfGAP with RhoGAP domain, ankyrin repeat and PH domain 3	106952
1427163_at	-1.085428231	7.469278495	-12.82301661	5.66E-09	5.76E-07	Ubr2	ubiquitin protein ligase E3 component n-recognin 2	224826

1451716_at	-1.201288158	8.339222166	-12.80002865	5.79E-09	5.81E-07	Mafb	v-maf musculoaponeurotic fibrosarcoma oncogene family, protein B (avian)	16658
1424048_a_at	-1.007489558	10.17070026	-12.78420263	5.88E-09	5.89E-07	Cyb5r1	cytochrome b5 reductase 1	72017
1452908_at	-1.050846173	6.942962349	-12.7608703	6.01E-09	6.00E-07	Dip2a	disco interacting protein 2 homolog A	64451
1417323_at	-1.259397721	7.35771857	-12.7294506	6.20E-09	6.15E-07	Psrc1	proline/serine-rich coiled-coil 1	56742
1428153_at	-1.105222137	9.079228252	-12.72305422	6.24E-09	6.16E-07	Mrps10	mitochondrial ribosomal protein S10	64657
1448721_at	-1.063344266	8.210087825	-12.64132749	6.77E-09	6.55E-07	D1Ertd622e	DNA segment, Chr 1, ERATO Doi 622, expressed	52392
1424323_at	-1.188119178	6.910150371	-12.62859209	6.86E-09	6.58E-07	Noc2l	NOC2 like nucleolar associated transcriptional repressor	57741
1425262_at	-1.02085158	8.129060943	-12.61363284	6.96E-09	6.67E-07	Cebpg	CCAAT/enhancer binding protein (C/EBP), gamma	12611
1426705_s_at	-1.059904644	8.256757353	-12.56659475	7.30E-09	6.96E-07	Iars	isoleucine-tRNA synthetase	105148
1437119_at	-1.295082916	7.000628456	-12.56080494	7.34E-09	6.98E-07	Ern1	endoplasmic reticulum (ER) to nucleus signalling 1	78943
1415712_at	-1.232661225	6.348112295	-12.54503747	7.45E-09	7.05E-07	Zranb1	zinc finger, RAN-binding domain containing 1	360216
1439153_at	-1.034163315	6.949666373	-12.54654418	7.44E-09	7.05E-07	Rnf144b	ring finger protein 144B	218215

1416814_at	-1.321484754	6.749676765	-12.51540104	7.68E-09	7.17E-07	Tia1	cytotoxic granule-associated RNA binding protein 1	21841
1460403_at	-1.297244299	6.736915683	-12.50724965	7.74E-09	7.19E-07	Psip1	PC4 and SFRS1 interacting protein 1	101739
1429758_at	-1.080952127	7.830029652	-12.49270734	7.86E-09	7.26E-07	1700017B05Rik	RIKEN cDNA 1700017B05 gene	74211
1418991_at	-1.363736721	7.280907628	-12.48584392	7.91E-09	7.30E-07	Bak1	BCL2-antagonist/killer 1	12018
1428635_at	-1.041997719	8.009052058	-12.48125626	7.95E-09	7.32E-07	Comtd1	catechol-O-methyltransferase domain containing 1	69156
1424792_at	-1.244750898	6.251627651	-12.4450421	8.24E-09	7.53E-07	Rpp40	ribonuclease P 40 subunit	208366
1428231_at	-1.157244365	7.901438761	-12.41694159	8.48E-09	7.71E-07	Cpsf6	cleavage and polyadenylation specific factor 6	432508
1417630_at	-1.050844232	8.052710426	-12.38763034	8.74E-09	7.90E-07	Mknk1	MAP kinase-interacting serine/threonine kinase 1	17346
1438385_s_at	-1.563321517	5.878829244	-12.31062614	9.45E-09	8.35E-07	Gpt2	glutamic pyruvate transaminase (alanine aminotransferase) 2	108682
1444058_at	-1.088381633	4.340134771	-12.30381458	9.51E-09	8.40E-07	Dzip3	DAZ interacting protein 3, zinc finger	224170
1452715_at	-1.070449346	5.70140497	-12.27164799	9.83E-09	8.61E-07	Haus5	HAUS augmin-like complex, subunit 5	71909
1437832_x_at	-1.045450342	7.920456436	-12.27166417	9.83E-09	8.61E-07	Wars	tryptophanyl-tRNA synthetase	22375
1428588_a_at	-1.039850865	8.179987561	-12.25327873	1.00E-08	8.71E-07	Mrpl41	mitochondrial ribosomal protein L41	107733
1419275_at	-1.088926633	7.979038985	-12.23776765	1.02E-08	8.81E-07	Dazap1	DAZ associated protein 1	70248

1450998_at	-1.066849773	5.59390452	-12.2351701	1.02E-08	8.82E-07	Zfp110	zinc finger protein 110	65020
1428673_at	-1.259403776	7.72573545	-12.18285743	1.08E-08	9.20E-07	Sympk	symplekin	68188
1424560_at	-1.106169958	8.333843387	-12.16983987	1.09E-08	9.28E-07	Pstpip1	proline-serine-threonine phosphatase-interacting protein 1	19200
1428798_s_at	-1.574771151	5.966493817	-12.14497446	1.12E-08	9.43E-07	Setd6	SET domain containing 6	66083
1451311_a_at	-1.059138521	9.498660916	-12.1325721	1.13E-08	9.50E-07	Adipor1	adiponectin receptor 1	72674
1433970_at	-1.196977723	8.502797105	-12.07992712	1.20E-08	9.83E-07	Bola3	bolA-like 3 (E. coli)	78653
1423364_a_at	-1.194093454	6.144958164	-12.0672386	1.21E-08	9.95E-07	Aktip	thymoma viral proto-oncogene 1 interacting protein	14339
1417061_at	-1.150860977	8.362443142	-12.04242346	1.24E-08	1.01E-06	Slc40a1	solute carrier family 40 (iron-regulated transporter), member 1	53945
1428949_at	-1.022174774	9.247259401	-12.03978744	1.25E-08	1.01E-06	Xpot	exportin, tRNA (nuclear export receptor for tRNAs)	73192
1456748_a_at	-1.091206421	6.774659079	-11.98272854	1.32E-08	1.06E-06	Nipsnap1	nipsnap homolog 1 (C. elegans)	18082
1452353_at	-1.058791692	5.890809122	-11.99298886	1.31E-08	1.06E-06	Gpr155	G protein-coupled receptor 155	68526
1430776_s_at	-1.147231754	6.084217923	-11.94893104	1.37E-08	1.09E-06	Ankrd24	ankyrin repeat domain 24	70615
1417010_at	-1.081040463	9.009990509	-11.94628154	1.38E-08	1.09E-06	Zbtb18	zinc finger and BTB domain containing 18	30928
1454701_at	-1.554714173	5.832817291	-11.92270644	1.41E-08	1.11E-06	4930503L19Rik	RIKEN cDNA 4930503L19 gene	269033
1453122_at	-1.028293421	7.735355409	-11.92662425	1.40E-08	1.11E-06	Fam135a	family with sequence similarity 135, member A	68187

1428709_a_at	-1.043617717	9.664636171	-11.87430254	1.48E-08	1.16E-06	Mrpl24	mitochondrial ribosomal protein L24	67707
1453045_at	-1.018144303	7.266352393	-11.83179651	1.55E-08	1.19E-06	Cep83	centrosomal protein 83	77048
1428347_at	-1.366828412	7.086162934	-11.7906266	1.62E-08	1.23E-06	Cyfip2	cytoplasmic FMR1 interacting protein 2	76884
1437327_x_at	-1.143422684	8.159331255	-11.77854284	1.64E-08	1.23E-06	Enoph1	enolase-phosphatase 1	67870
1448971_at	-1.069080833	6.238523484	-11.7663234	1.66E-08	1.24E-06	Coprs	coordinator of PRMT5, differentiation stimulator	66423
1455790_at	-1.223595578	5.337435076	-11.7584209	1.68E-08	1.25E-06	E2f2	E2F transcription factor 2	242705
1435822_at	-1.004995124	8.298920741	-11.74703361	1.70E-08	1.26E-06	D830012I24Rik	RIKEN cDNA D830012I24 gene	320070
1432494_a_at	-1.041656994	7.2325657	-11.72878658	1.73E-08	1.28E-06	Ift43	intraflagellar transport 43	76411
1449078_at	-1.642026167	6.386391025	-11.70346194	1.78E-08	1.32E-06	St3gal6	ST3 beta-galactoside alpha-2,3-sialyltransferase 6	54613
1434278_at	-1.522270876	5.711945471	-11.66789	1.85E-08	1.36E-06	Mtm1	X-linked myotubular myopathy gene 1	17772
1450350_a_at	-1.017144603	7.529870832	-11.66178557	1.86E-08	1.36E-06	Jdp2	Jun dimerization protein 2	81703
1448774_at	-1.290514015	7.791882281	-11.65418427	1.87E-08	1.37E-06	Stoml2	stomatin (Epb7.2)-like 2	66592
1448402_at	-1.137641135	9.584578266	-11.64187825	1.90E-08	1.38E-06	Tln1	talin 1	21894
1460360_at	-1.042182924	7.428044004	-11.62077912	1.94E-08	1.40E-06	Asrgl1	asparaginase like 1	66514
1429382_at	-1.024824877	6.883503075	-11.61919401	1.94E-08	1.40E-06	Tomm40l	translocase of outer mitochondrial membrane 40 homolog-like (yeast)	641376
1436892_at	-1.245739465	5.746821483	-11.61411896	1.95E-08	1.41E-06	Spred2	sprouty-related, EVH1 domain containing 2	114716
1434813_x_at	-1.168720795	8.592322338	-11.58982014	2.01E-08	1.43E-06	Wars	tryptophanyl-tRNA synthetase	22375

1438052_at	-1.085484627	5.288191328	-11.58954162	2.01E-08	1.43E-06	A130071D04Rik	RIKEN cDNA A130071D04 gene	320791
1435855_x_at	-1.201593906	7.896457951	-11.57799852	2.03E-08	1.44E-06	Aldh18a1	aldehyde dehydrogenase 18 family, member A1	56454
1452020_a_at	-1.141300593	7.782264757	-11.55060203	2.09E-08	1.47E-06	Siva1	SIVA1, apoptosis-inducing factor	30954
1425927_a_at	-1.166942364	7.956310137	-11.53197763	2.13E-08	1.49E-06	Atf5	activating transcription factor 5	107503
1448907_at	-1.133256287	6.252887838	-11.52685243	2.15E-08	1.50E-06	Thop1	thimet oligopeptidase 1	50492
1451330_a_at	-1.100643574	7.98275008	-11.52546495	2.15E-08	1.50E-06	Inpp5b	inositol polyphosphate-5-phosphatase B	16330
1441045_at	-1.329007145	5.29221493	-11.50583201	2.20E-08	1.52E-06	Ddx43	DEAD (Asp-Glu-Ala-Asp) box polypeptide 43	100048658
1437164_x_at	-1.012189612	9.577485744	-11.50715721	2.19E-08	1.52E-06	Atp5o	ATP synthase, H+ transporting, mitochondrial F1 complex, O subunit	28080
1424643_at	-1.217673557	6.791042604	-11.49427784	2.22E-08	1.54E-06	Tcof1	treacle ribosome biogenesis factor 1	21453
1451083_s_at	-1.142868106	9.287581167	-11.49267309	2.23E-08	1.54E-06	Aars	alanyl-tRNA synthetase	234734
1448472_at	-1.279633128	8.16945236	-11.47179664	2.28E-08	1.55E-06	Vars	valyl-tRNA synthetase	22321
1448189_a_at	-1.092276892	7.671344134	-11.47195453	2.28E-08	1.55E-06	Flii	flightless I actin binding protein	14248
1426893_at	-1.202695852	6.215717662	-11.44908165	2.33E-08	1.58E-06	Fam102a	family with sequence similarity 102, member A	98952
1428474_at	-1.011830057	4.868086153	-11.45033345	2.33E-08	1.58E-06	Ppp3cb	protein phosphatase 3, catalytic subunit, beta isoform	19056

1420743_a_at	-1.096006345	5.120134933	-11.4344544	2.37E-08	1.60E-06	Ppp3cc	protein phosphatase 3, catalytic subunit, gamma isoform	19057
1423685_at	-1.021685689	8.899651075	-11.42737166	2.39E-08	1.61E-06	Aars	alanyl-tRNA synthetase	234734
1428570_at	-1.299812434	6.780538202	-11.41650755	2.42E-08	1.62E-06	Ccnc	cyclin C	51813
1416691_at	-1.080153386	7.569769184	-11.38965564	2.49E-08	1.66E-06	Gtpbp2	GTP binding protein 2	56055
1449446_at	-1.103202246	7.286381885	-11.36901881	2.55E-08	1.68E-06	Ccdc59	coiled-coil domain containing 59	52713
1443837_x_at	-1.018519991	4.666267768	-11.36868002	2.55E-08	1.68E-06	Bcl2	B cell leukemia/lymphoma 2	12043
1444177_at	-1.341961801	6.940932996	-11.34424956	2.62E-08	1.70E-06	E330020D12Rik	Riken cDNA E330020D12 gene	626058
1452061_s_at	-1.191639244	6.898107055	-11.3563495	2.58E-08	1.70E-06	Strbp	spermatid perinuclear RNA binding protein	20744
1454848_at	-1.108849294	6.741746496	-11.34757606	2.61E-08	1.70E-06	Ppp1r12c	protein phosphatase 1, regulatory (inhibitor) subunit 12C	232807
1417601_at	-1.68125983	9.209645535	-11.33585391	2.64E-08	1.72E-06	Rgs1	regulator of G-protein signaling 1	50778
1453016_at	-1.413348924	6.199320216	-11.33121644	2.65E-08	1.72E-06	Hspb11	heat shock protein family B (small), member 11	72938
1448546_at	-1.130656225	7.333461353	-11.32393979	2.67E-08	1.73E-06	Rassf3	Ras association (RalGDS/AF-6) domain family member 3	192678
1436994_a_at	-1.672216732	8.876141336	-11.31955777	2.69E-08	1.74E-06	Hist1h1c	histone cluster 1, H1c	50708
1437660_at	-1.516255726	5.907592239	-11.31039015	2.71E-08	1.75E-06	Nktr	natural killer tumor recognition sequence	18087

1416812_at	-1.034284813	7.671769701	-11.29676572	2.75E-08	1.76E-06	Tia1	cytotoxic granule-associated RNA binding protein 1	21841
1449633_s_at	-1.041086421	6.879940199	-11.25153344	2.89E-08	1.83E-06	Nt5c3b	5'-nucleotidase, cytosolic IIIB	68106
1448944_at	-1.023050579	8.87088218	-11.25140541	2.90E-08	1.83E-06	Nrp1	neuropilin 1	18186
1417410_s_at	-1.048714539	7.471219907	-11.2319059	2.96E-08	1.86E-06	Prkci	protein kinase C, iota	18759
1435800_a_at	-1.100574962	10.66969109	-11.22183121	2.99E-08	1.87E-06	Ybx3	Y box protein 3	56449
1418774_a_at	-1.020870226	8.709704542	-11.18061914	3.13E-08	1.95E-06	Atp7a	ATPase, Cu++ transporting, alpha polypeptide	11977
1448899_s_at	-1.094579507	7.609102623	-11.15685109	3.21E-08	1.98E-06	Rad51ap1	RAD51 associated protein 1	19362
1434294_at	-1.067699545	6.51206082	-11.16129319	3.20E-08	1.98E-06	Fam199x	family with sequence similarity 199, X-linked	245622
1439189_at	-1.105404322	8.408847615	-11.12604683	3.32E-08	2.03E-06	Fnip2	folliculin interacting protein 2	329679
1438767_at	-1.02502529	7.62702088	-11.10579628	3.40E-08	2.06E-06	Osm	oncostatin M	18413
1439107_a_at	-1.082310126	5.973504346	-11.08781541	3.47E-08	2.10E-06	Kmt2e	lysine (K)-specific methyltransferase 2E	69188
1448923_at	-1.079296697	7.437213529	-11.07483015	3.52E-08	2.11E-06	Prkra	protein kinase, interferon inducible double stranded RNA dependent activator	23992
1428769_at	-1.020760713	6.34741419	-11.04970302	3.62E-08	2.15E-06	Tatdn3	TatD DNase domain containing 3	68972
1435575_at	-1.076867688	6.98385759	-11.04392164	3.64E-08	2.16E-06	Kntc1	kinetochore associated 1	208628

1434060_at	-1.156751363	6.841892318	-11.03852487	3.66E-08	2.17E-06	Herc1	HECT and RLD domain containing E3 ubiquitin protein ligase family member 1	235439
1434403_at	-1.263121288	7.910849182	-10.98010036	3.91E-08	2.28E-06	Spred2	sprouty-related, EVH1 domain containing 2	114716
1431873_a_at	-1.058054559	4.707900512	-10.98074253	3.91E-08	2.28E-06	Tube1	epsilon-tubulin 1	71924
1460348_at	-1.033303308	6.845483684	-10.97457745	3.94E-08	2.28E-06	Mad2l2	MAD2 mitotic arrest deficient-like 2	71890
1417353_x_at	-1.022388671	9.444757639	-10.97920205	3.91E-08	2.28E-06	Snrpa1	small nuclear ribonucleoprotein polypeptide A'	68981
1418326_at	-1.198631822	8.1344067	-10.96407107	3.98E-08	2.30E-06	Slc7a5	solute carrier family 7 (cationic amino acid transporter, y+ system), member 5	20539
1455712_at	-1.011670639	4.52251908	-10.96275716	3.99E-08	2.30E-06	Hist3h2a	histone cluster 3, H2a	319162
1423877_at	-1.037939124	7.401297901	-10.93606976	4.11E-08	2.35E-06	Chaf1b	chromatin assembly factor 1, subunit B (p60)	110749
1448543_at	-1.002818695	8.065531502	-10.92830411	4.15E-08	2.36E-06	Slmo2	slowmo homolog 2 (Drosophila)	66390
1417431_a_at	-1.293900064	6.721719009	-10.92421691	4.16E-08	2.37E-06	Sphk2	sphingosine kinase 2	56632
1451108_at	-1.185601023	7.045416185	-10.91311178	4.22E-08	2.39E-06	Rnf185	ring finger protein 185	193670
1417938_at	-1.033213576	8.229731828	-10.91297384	4.22E-08	2.39E-06	Rad51ap1	RAD51 associated protein 1	19362
1435492_at	-1.277819083	5.709332749	-10.87328737	4.41E-08	2.48E-06	Socs6	suppressor of cytokine signaling 6	54607
1436759_x_at	-1.255586924	3.947271616	-10.86021216	4.48E-08	2.51E-06	Cnn3	calponin 3, acidic	71994
1452331_s_at	-1.09747701	5.173116123	-10.86105241	4.47E-08	2.51E-06	Qser1	glutamine and serine rich 1	99003

1460677_at	-1.039411746	7.726215139	-10.80907745	4.74E-08	2.65E-06	Spats2	spermatogenesis associated, serine-rich 2	72572
1423581_at	-1.032871738	5.168109207	-10.7987897	4.80E-08	2.67E-06	Nmt2	N-myristoyltransferase 2	18108
1416934_at	-1.248260159	6.658419991	-10.76256851	5.00E-08	2.76E-06	Mtm1	X-linked myotubular myopathy gene 1	17772
1451992_at	-1.062340727	8.446737172	-10.74972543	5.07E-08	2.78E-06	Grk2	G protein-coupled receptor kinase 2	110355
1417331_a_at	-1.068612715	7.063204922	-10.7352939	5.16E-08	2.81E-06	Arl6	ADP-ribosylation factor-like 6	56297
1436186_at	-1.006336721	7.428038001	-10.73536473	5.16E-08	2.81E-06	E2f8	E2F transcription factor 8	108961
1452394_at	-1.129460197	8.239181328	-10.68748226	5.45E-08	2.92E-06	Cars	cysteinyl-tRNA synthetase	27267
1416648_at	-1.077542902	8.863271179	-10.68669258	5.45E-08	2.92E-06	Dync1h1	dynein cytoplasmic 1 heavy chain 1	13424
1436766_at	-1.386646309	7.125460049	-10.67086611	5.55E-08	2.96E-06	Luc7l2	LUC7-like 2 (S. cerevisiae)	192196
1427055_at	-1.193632451	6.505645883	-10.66798866	5.57E-08	2.97E-06	Them4	thioesterase superfamily member 4	75778
1451044_at	-1.073553825	6.508425511	-10.65842649	5.63E-08	2.99E-06	Gemin2	gem (nuclear organelle) associated protein 2	66603
1456109_a_at	-1.033448436	8.120630406	-10.648535	5.70E-08	3.02E-06	Mrps15	mitochondrial ribosomal protein S15	66407
1437333_x_at	-1.063240701	6.018472163	-10.62154375	5.88E-08	3.08E-06	Aldh18a1	aldehyde dehydrogenase 18 family, member A1	56454
1460741_x_at	-1.034908525	6.302989996	-10.56801613	6.25E-08	3.24E-06	D17Wsu92e	DNA segment, Chr 17, Wayne State University 92, expressed	224647
1452359_at	-1.068684506	5.531904875	-10.55718195	6.33E-08	3.25E-06	Rell1	RELT-like 1	100532
1455951_at	-1.085818405	8.987985724	-10.54160566	6.44E-08	3.29E-06	Mars	methionine-tRNA synthetase	216443

1460675_at	-1.109136625	9.400521474	-10.49765697	6.78E-08	3.41E-06	Igsf8	immunoglobulin superfamily, member 8	140559
1450769_s_at	-1.2699389	5.325289505	-10.47402806	6.97E-08	3.48E-06	Stard5	StAR-related lipid transfer (START) domain containing 5	170460
1430357_at	-1.05466209	6.635193183	-10.47593042	6.95E-08	3.48E-06	H3f3b	H3 histone, family 3B	15081
1436025_at	-1.03063455	6.443000111	-10.43850338	7.26E-08	3.60E-06	Ccdc88a	coiled coil domain containing 88A	108686
1423941_at	-1.102478659	6.874437163	-10.43117691	7.33E-08	3.62E-06	Camk2g	calcium/calmodulin-dependent protein kinase II gamma	12325
1418377_a_at	-1.091936761	7.039631039	-10.40609939	7.54E-08	3.70E-06	Siva1	SIVA1, apoptosis-inducing factor	30954
1424412_at	-1.051295742	8.241410188	-10.31785978	8.37E-08	3.98E-06	Ogfrl1	opioid growth factor receptor-like 1	70155
1416714_at	-1.015720324	8.785729204	-10.31464816	8.40E-08	3.99E-06	Irf8	interferon regulatory factor 8	15900
1450018_s_at	-1.042830342	6.413670088	-10.30047456	8.54E-08	4.05E-06	Slc25a30	solute carrier family 25, member 30	67554
1428859_at	-1.101512794	6.251318926	-10.29566686	8.59E-08	4.06E-06	Paox	polyamine oxidase (exo-N4-amino)	212503
1424143_a_at	-1.027453411	9.925232668	-10.29428	8.60E-08	4.06E-06	Cdt1	chromatin licensing and DNA replication factor 1	67177
1418181_at	-1.001999369	5.93701709	-10.26760134	8.88E-08	4.16E-06	Ptp4a3	protein tyrosine phosphatase 4a3	19245
1437216_at	-1.118788999	6.551931456	-10.24783075	9.09E-08	4.23E-06	Ccdc88a	coiled coil domain containing 88A	108686
1433893_s_at	-1.027121318	7.800451108	-10.24621973	9.10E-08	4.24E-06	Spag5	sperm associated antigen 5	54141

1417657_s_at	-1.00401989	8.324119041	-10.2376106	9.20E-08	4.26E-06	Dnajc2	DnaJ heat shock protein family (Hsp40) member C2	22791
1436183_at	-1.268253577	8.75947155	-10.22750936	9.31E-08	4.30E-06	Zc3hav1	zinc finger CCCH type, antiviral 1	78781
1429351_at	-1.123394087	6.003288474	-10.21846708	9.41E-08	4.34E-06	Klhl24	kelch-like 24	75785
1452606_at	-1.440885587	5.565312014	-10.15870283	1.01E-07	4.59E-06	Mnd1	meiotic nuclear divisions 1	76915
1437627_at	-1.165254272	5.174841891	-10.13778964	1.04E-07	4.66E-06	Mex3d	mex3 RNA binding family member D	237400
1416587_a_at	-1.058704961	6.83933486	-10.10798988	1.07E-07	4.77E-06	Xrcc1	X-ray repair complementing defective repair in Chinese hamster cells 1	22594
1448187_at	-1.027543568	7.554585883	-10.11103537	1.07E-07	4.77E-06	Pold1	polymerase (DNA directed), delta 1, catalytic subunit	18971
1427033_at	-1.110119008	6.310274515	-10.09282424	1.09E-07	4.81E-06	Dnmbp	dynamin binding protein	71972
1416206_at	-1.23582873	7.117314805	-10.0857104	1.10E-07	4.84E-06	Sipa1	signal-induced proliferation associated gene 1	20469
1437236_a_at	-1.040970958	5.985180626	-10.06345454	1.13E-07	4.95E-06	Zfp110	zinc finger protein 110	65020
1455218_at	-1.190597749	6.076658871	-10.03937899	1.17E-07	5.02E-06	Ccp110	centriolar coiled coil protein 110	101565
1418157_at	-1.151739042	5.339992635	-10.03533682	1.17E-07	5.03E-06	Nr2f1	nuclear receptor subfamily 2, group F, member 1	13865
1455746_at	-1.282923577	5.409106512	-9.974327933	1.26E-07	5.28E-06	Kif13a	kinesin family member 13A	16553
1434543_a_at	-1.028317453	9.81548686	-9.935765413	1.32E-07	5.46E-06	Bola2	bolA-like 2 (E. coli)	66162
1424616_s_at	-1.249238195	7.207257242	-9.924114944	1.34E-07	5.51E-06	Pgap2	post-GPI attachment to proteins 2	233575

1460370_at	-1.080614971	6.774211965	-9.905491893	1.37E-07	5.59E-06	Top1mt	DNA topoisomerase 1, mitochondrial	72960
1442003_at	-1.128239721	6.807902098	-9.881362696	1.41E-07	5.72E-06	Diaph2	diaphanous related formin 2	54004
1435348_at	-1.132873756	5.921402083	-9.848771355	1.47E-07	5.88E-06	Kdm5c	lysine (K)-specific demethylase 5C	20591
1450935_at	-1.087965849	6.085819959	-9.844136364	1.48E-07	5.90E-06	Ercc5	excision repair cross-complementing rodent repair deficiency, complementation group 5	22592
1419866_s_at	-1.212515888	6.025756107	-9.835044725	1.49E-07	5.94E-06	Atxn2	ataxin 2	20239
1444350_at	-1.027911864	5.113127433	-9.822629274	1.52E-07	6.01E-06	Slfn10-ps	schlafen 10, pseudogene	237887
1452438_s_at	-1.128904299	6.049456045	-9.778839526	1.60E-07	6.24E-06	Taf4	TATA-box binding protein associated factor 4	228980
1442744_at	-1.207447983	6.045141846	-9.756810061	1.64E-07	6.39E-06	Rbm39	RNA binding motif protein 39	170791
1418332_a_at	-1.168768694	6.455835618	-9.755381808	1.65E-07	6.39E-06	Agtpbp1	ATP/GTP binding protein 1	67269
1435866_s_at	-1.509171565	6.755876367	-9.663425173	1.84E-07	6.90E-06	Hist3h2a	histone cluster 3, H2a	319162
1427347_s_at	-1.079623516	5.717498425	-9.637362849	1.90E-07	7.04E-06	Tubb2a	tubulin, beta 2A class IIA	22151
1429692_s_at	-1.22180291	6.104952703	-9.601806591	1.99E-07	7.26E-06	Gch1	GTP cyclohydrolase 1	14528
1427416_x_at	-1.008297199	6.416008133	-9.580852376	2.04E-07	7.41E-06	Dusp7	dual specificity phosphatase 7	235584
1417656_at	-1.020882295	6.579841408	-9.570115219	2.07E-07	7.50E-06	Mybl2	myeloblastosis oncogene-like 2	17865
1420981_a_at	-1.066165992	8.608132556	-9.563507381	2.09E-07	7.54E-06	Lmo4	LIM domain only 4	16911
1456712_at	-1.289550098	4.796641174	-9.551277675	2.12E-07	7.62E-06	Lcorl	ligand dependent nuclear receptor corepressor-like	209707

1424577_at	-1.006567941	7.7276904	-9.542048428	2.14E-07	7.69E-06	Msto1	misato 1, mitochondrial distribution and morphology regulator	229524
1451313_a_at	-1.589323305	6.282837052	-9.535583514	2.16E-07	7.73E-06	Lgalsl	lectin, galactoside binding-like	216551
1422521_at	-1.028689149	7.322571346	-9.483382232	2.31E-07	8.07E-06	Dctn1	dynactin 1	13191
1428656_at	-1.119324533	6.732175339	-9.346403954	2.74E-07	9.19E-06	Drosha	drosha, ribonuclease type III	14000
1441139_at	-1.073093295	5.253599372	-9.330176048	2.80E-07	9.34E-06	Gm10336	predicted gene 10336	328186
1459840_s_at	-1.046960066	6.043007751	-9.319380268	2.84E-07	9.43E-06	Ccdc28b	coiled coil domain containing 28B	66264
1416892_s_at	-1.227427715	7.475501921	-9.303018418	2.90E-07	9.57E-06	Fam107b	family with sequence similarity 107, member B	66540
1428228_at	-1.061218165	7.033580773	-9.296685208	2.92E-07	9.62E-06	Pgm3	phosphoglucomutase 3	109785
1450687_at	-1.109372515	6.031854094	-9.289575262	2.95E-07	9.67E-06	Igf2bp3	insulin-like growth factor 2 mRNA binding protein 3	140488
1425814_a_at	-1.434719608	4.895335201	-9.243312745	3.13E-07	1.01E-05	Calcrl	calcitonin receptor-like	54598
1434408_at	-1.072936951	6.626294744	-9.210761545	3.26E-07	1.04E-05	Atxn3	ataxin 3	110616
1416081_at	-1.009131882	5.279154317	-9.206282934	3.28E-07	1.04E-05	Smad1	SMAD family member 1	17125
1452111_at	-1.016293624	8.515033173	-9.11408652	3.70E-07	1.14E-05	Mrps35	mitochondrial ribosomal protein S35	232536
1436986_at	-1.231390493	5.095943696	-9.084895865	3.84E-07	1.17E-05	Sntb2	syntrophin, basic 2	20650
1417394_at	-1.172378865	7.252679844	-8.899615602	4.89E-07	1.40E-05	Klf4	Kruppel-like factor 4 (gut)	16600
1422440_at	-1.031884397	8.677639215	-8.865650366	5.11E-07	1.45E-05	Cdk4	cyclin-dependent kinase 4	12567
1426411_a_at	-1.044735013	5.534753204	-8.856390331	5.18E-07	1.46E-05	Strbp	spermatid perinuclear RNA binding protein	20744
1424239_at	-1.018757971	5.447154528	-8.853402726	5.20E-07	1.46E-05	Fam65a	family with sequence similarity 65, member A	75687

1439433_a_at	-1.03035548	5.95722005	-8.735961299	6.07E-07	1.65E-05	Slc35a2	solute carrier family 35 (UDP-galactose transporter), member A2	22232
1439998_at	-1.005275243	5.174819622	-8.589605339	7.39E-07	1.91E-05	Jmjd1c	jumonji domain containing 1C	108829
1427102_at	-1.05095025	9.42399862	-8.588521382	7.41E-07	1.92E-05	Slfn4	schlafen 4	20558
1437878_s_at	-1.099191287	6.984116878	-8.580897193	7.48E-07	1.93E-05	Ttc14	tetratricopeptide repeat domain 14	67120
1455195_at	-1.032567504	5.182431868	-8.549308503	7.81E-07	1.99E-05	Rps24	ribosomal protein S24	20088
1434175_s_at	-1.042433656	7.597633599	-8.533932397	7.97E-07	2.02E-05	Tecpr1	tectonin beta-propeller repeat containing 1	70381
1450896_at	-1.149539301	6.077362769	-8.49434974	8.41E-07	2.10E-05	Arhgap5	Rho GTPase activating protein 5	11855
1434471_at	-1.035261757	7.155164902	-8.400190959	9.57E-07	2.31E-05	BC003331	cDNA sequence BC003331	226499
1421205_at	-1.040953796	6.85910356	-8.204628251	1.25E-06	2.85E-05	Atm	ataxia telangiectasia mutated	11920
1452179_at	-1.377336818	5.35685929	-8.160293752	1.33E-06	2.99E-05	Jade1	jade family PHD finger 1	269424
1458667_at	-1.465458173	4.080267781	-8.105838985	1.44E-06	3.17E-05	Ninl	ninein-like	78177
1428915_at	-1.207289284	5.480927454	-8.070717265	1.51E-06	3.29E-05	Sirt5	sirtuin 5	68346
1427049_s_at	-1.056054617	6.231324152	-7.859114774	2.05E-06	4.12E-05	Smo	smoothened, frizzled class receptor	319757
1428267_at	-1.168570105	5.930189042	-7.829060361	2.14E-06	4.25E-05	Dhx40	DEAH (Asp-Glu-Ala-His) box polypeptide 40	67487
1436735_at	-1.173817349	5.69922052	-7.826859131	2.14E-06	4.26E-05	Nsun3	NOL1/NOP2/Sun domain family member 3	106338
1438238_at	-1.006686353	6.63530186	-7.582490751	3.06E-06	5.61E-05	2010315B03Rik	RIKEN cDNA 2010315B03 gene	630836

1438719_at	-1.13847636	5.659032619	-7.176601219	5.61E-06	9.03E-05	Map3k2	mitogen-activated protein kinase kinase kinase 2	26405
1456577_x_at	-1.142044435	5.691562534	-7.086779463	6.44E-06	0.000100228	Pitrm1	pitrilysin metallepetidase 1	69617
1435052_at	-1.12403905	6.105494137	-6.926829688	8.25E-06	0.000121489	Ccdc181	coiled-coil domain containing 181	74895
1437372_at	-1.025792522	4.398931137	-6.886955572	8.77E-06	0.000127239	Cpsf6	cleavage and polyadenylation specific factor 6	432508
1419810_x_at	-1.011499085	6.500610595	-6.789945196	1.02E-05	0.000143249	Arhgap9	Rho GTPase activating protein 9	216445
1427151_at	-1.029756828	4.744348014	-6.422656541	1.84E-05	0.000229433	Qser1	glutamine and serine rich 1	99003
1425495_at	-1.029965628	6.213751426	-6.130393807	2.97E-05	0.00033732	Zfp62	zinc finger protein 62	22720
1451511_at	-1.311242948	5.15193952	-5.976846314	3.84E-05	0.000414229	Hibch	3-hydroxyisobutyryl-Coenzyme A hydrolase	227095
1422139_at	-1.124561958	7.457014947	-5.94916927	4.03E-05	0.000429615	Plau	plasminogen activator, urokinase	18792
1436202_at	-1.117860171	7.583399706	-5.822350927	4.99E-05	0.000509856	Malat1	metastasis associated lung adenocarcinoma transcript 1 (non-coding RNA)	72289
1427797_s_at	-1.095550072	6.039229332	-5.64314315	6.79E-05	0.000654221	Ctse	cathepsin E	13034
1449840_at	-1.047766289	6.183114334	-5.301676617	0.000123724	0.00107371	Sntb2	syntrophin, basic 2	20650
1450407_a_at	-1.046792192	8.435088852	-3.961067087	0.001500653	0.008189936	Anp32a	acidic (leucine-rich) nuclear phosphoprotein 32 family, member A	11737
