# Supplementary material for: Integrative Transcriptomic Profiling Identifies TNF and IL1B as Candidate Key Early-Response Genes in Macrophages Infected with Smooth Brucella Using a Comprehensive Bioinformatic Approach
Source: Biology (Basel). 2025 May 21;14(5):579. doi: 10.3390/biology14050579 (PMC12109160; doi:10.3390/biology14050579)
Supplement: Supplementary file 1 [file biology-14-00579-s001.zip › Table S5.pdf]

Table S5 GO terms enriched by 45 common differentially expressed genes

pathway ID	pathway description	observed gene count	false discovery rate	matching proteins in your network (labels)
GO:0009987	cellular process	40	4.50E-115	Atp7a,Bcl3,Birc3,Ccng2,Ccrl2,Cd69,Cd83,Cdc42ep2,Cflar,Dusp6,Ehd1,Ell2,Fas,Gadd45b,Hcar2,Herpud1,Icam1,Icosl,Ier3,Il10ra,Il1b,Klf2,Nfe2l2,Nfkbia,Nfkbiz,Pdgfb,Ptger4,Ptgs2,Rilpl2,Rnf19b,Skil,Slc31a2,Socs3,Tlr2,Tnf,Tnfaip3,Tnip1,Traf1,Zc3h12a,Zc3h12c
GO:0050789	regulation of biological process	38	3.30E-108	Atp7a,Bcl3,Birc3,Ccng2,Ccrl2,Cd83,Cdc42ep2,Cflar,Dusp6,Ehd1,Ell2,Fam46c,Fas,Gadd45b,Hcar2,Herpud1,Icam1,Icosl,Ier3,Il10ra,Il1b,Klf2,Nfe2l2,Nfkbia,Nfkbiz,Pdgfb,Ptger4,Ptgs2,Rnf19b,Skil,Slc31a2,Socs3,Tlr2,Tnf,Tnfaip3,Tnip1,Traf1,Zc3h12a
GO:0050794	regulation of cellular process	37	3.95E-105	Atp7a,Bcl3,Birc3,Ccng2,Ccrl2,Cd83,Cdc42ep2,Cflar,Dusp6,Ehd1,Ell2,Fam46c,Fas,Gadd45b,Hcar2,Herpud1,Icam1,Icosl,Ier3,Il10ra,Il1b,Klf2,Nfe2l2,Nfkbia,Nfkbiz,Pdgfb,Ptger4,Ptgs2,Rnf19b,Skil,Socs3,Tlr2,Tnf,Tnfaip3,Tnip1,Traf1,Zc3h12a
GO:0050896	response to stimulus	36	8.62E-102	Atp7a,Bcl3,Birc3,Ccrl2,Cd69,Cd83,Cdc42ep2,Cflar,Dusp6,Ehd1,Fas,Gadd45b,Hcar2,Herpud1,Icam1,Icosl,Ier3,Il10ra,Il1b,Klf2,Nfe2l2,Nfkbia,Nfkbie,Nfkbiz,Pdgfb,Ptger4,Ptgs2,Rnf19b,Skil,Socs3,Tlr2,Tnf,Tnfaip3,Tnip1,Traf1,Zc3h12a
GO:0048518	positive regulation of biological process	34	1.27E-90	Atp7a,Bcl3,Birc3,Cd83,Cdc42ep2,Cflar,Dusp6,Ehd1,Ell2,Fam46c,Fas,Gadd45b,Hcar2,Herpud1,Icam1,Icosl,Ier3,Il1b,Klf2,Nfe2l2,Nfkbia,Nfkbiz,Pdgfb,Ptger4,Ptgs2,Rnf19b,Skil,Socs3,Tlr2,Tnf,Tnfaip3,Tnip1,Traf1,Zc3h12a
GO:0048522	positive regulation of cellular process	31	1.64E-85	Atp7a,Bcl3,Birc3,Cd83,Cdc42ep2,Cflar,Dusp6,Ehd1,Ell2,Fas,Gadd45b,Hcar2,Herpud1,Icam1,Icosl,Il1b,Klf2,Nfe2l2,Nfkbia,Nfkbiz,Pdgfb,Ptger4,Ptgs2,Rnf19b,Skil,Socs3,Tlr2,Tnf,Tnfaip3,Tnip1,Zc3h12a

GO:0080090	regulation of primary metabolic process	31	1.44E-83	Atp7a,Bcl3,Birc3,Cflar,Dusp6,Ell2,Fam46c,Fas,Gadd45b,Hcar2,Herpud1,Icam1,Icosl,Ier3,Il1b,Klf2,Nfe2l2,Nfkbia,Nfkbiz,Pdgfb,Ptger4,Ptgs2,Rnf19b,Skil,Socs3,Tlr2,Tnf,Tnfaip3,Tnip1,Traf1,Zc3h12a
GO:0051171	regulation of nitrogen compound metabolic process	30	1.94E-82	Atp7a,Bcl3,Birc3,Cflar,Dusp6,Ell2,Fam46c,Fas,Gadd45b,Herpud1,Icam1,Icosl,Ier3,Il1b,Klf2,Nfe2l2,Nfkbia,Nfkbiz,Pdgfb,Ptger4,Ptgs2,Rnf19b,Skil,Socs3,Tlr2,Tnf,Tnfaip3,Tnip1,Traf1,Zc3h12a
GO:0060255	regulation of macromolecule metabolic process	30	1.94E-81	Atp7a,Bcl3,Birc3,Cflar,Dusp6,Ell2,Fam46c,Fas,Gadd45b,Herpud1,Icam1,Icosl,Ier3,Il1b,Klf2,Nfe2l2,Nfkbia,Nfkbiz,Pdgfb,Ptger4,Ptgs2,Rnf19b,Skil,Socs3,Tlr2,Tnf,Tnfaip3,Tnip1,Traf1,Zc3h12a
GO:0051716	cellular response to stimulus	32	7.87E-79	Atp7a,Bcl3,Birc3,Ccrl2,Cd69,Cd83,Cdc42ep2,Cflar,Ehd1,Fas,Hcar2,Herpud1,Icam1,Icosl,Ier3,Il10ra,Il1b,Klf2,Nfe2l2,Nfkbia,Nfkbiz,Pdgfb,Ptger4,Ptgs2,Skil,Socs3,Tlr2,Tnf,Tnfaip3,Tnip1,Traf1,Zc3h12a
GO:0042221	response to chemical	28	1.84E-71	Atp7a,Birc3,Ccrl2,Cd69,Cd83,Cdc42ep2,Cflar,Dusp6,Ehd1,Fas,Herpud1,Icam1,Il10ra,Il1b,Klf2,Nfe2l2,Nfkbia,Nfkbie,Pdgfb,Ptger4,Ptgs2,Skil,Socs3,Tlr2,Tnf,Tnfaip3,Tnip1,Zc3h12a
GO:0048519	negative regulation of biological process	27	1.48E-70	Atp7a,Bcl3,Birc3,Cd83,Cflar,Dusp6,Fam46c,Fas,Gadd45b,Hcar2,Herpud1,Icam1,Ier3,Il1b,Klf2,Nfe2l2,Nfkbia,Pdgfb,Ptger4,Ptgs2,Skil,Socs3,Tlr2,Tnf,Tnfaip3,Tnip1,Zc3h12a
GO:0048583	regulation of response to stimulus	26	3.18E-70	Atp7a,Bcl3,Birc3,Cdc42ep2,Cflar,Dusp6,Fas,Gadd45b,Herpud1,Icam1,Ier3,Il1b,Nfe2l2,Nfkbia,Nfkbiz,Pdgfb,Ptger4,Ptgs2,Skil,Socs3,Tlr2,Tnf,Tnfaip3,Tnip1,Traf1,Zc3h12a
GO:0010033	response to organic substance	26	2.16E-68	Atp7a,Birc3,Ccrl2,Cd83,Cdc42ep2,Cflar,Dusp6,Ehd1,Fas,Herpud1,Icam1,Il10ra,Il1b,Klf2,Nfe2l2,Nfkbia,Pdgfb,Ptger4,Ptgs2,Skil,Socs3,Tlr2,Tnf,Tnfaip3,Tnip1,Zc3h12a
GO:0006950	response to stress	25	3.03E-67	Atp7a,Bcl3,Ccrl2,Cd83,Cdc42ep2,Cflar,Dusp6,Fas,Gadd45b,Herpud1,Icam1,Ier3,Il1b,Klf2,Nfe2l2,Nfkbiz,Pdgfb,Ptger4,Ptgs2,Rnf19b,Tlr2,Tnf,Tnfaip3,Tnip1,Zc3h12a

GO:0007154	cell communication	25	3.03E-67	Atp7a,Bcl3,Ccrl2,Cd83,Cdc42ep2,Fas,Hcar2,Herpud1,Icosl,Ier3,Il10ra,Il1b,Nfe2l2,Nfkbia,Nfkbiz,Pdgfb,Ptger4,Ptgs2,Skil,Socs3,Tlr2,Tnf,Tnip1,Traf1,Zc3h12a
GO:0009893	positive regulation of metabolic process	25	3.03E-67	Atp7a,Bcl3,Birc3,Cflar,Ell2,Fam46c,Fas,Gadd45b,Herpud1,Icam1,Icosl,Ier3,Il1b,Klf2,Nfe2l2,Nfkbia,Pdgfb,Ptger4,Ptgs2,Rnf19b,Tlr2,Tnf,Tnfaip3,Tnip1,Zc3h12a
GO:0048523	negative regulation of cellular process	25	3.03E-67	Atp7a,Bcl3,Birc3,Cflar,Dusp6,Fam46c,Fas,Gadd45b,Herpud1,Icam1,Ier3,Il1b,Klf2,Nfe2l2,Nfkbia,Pdgfb,Ptger4,Ptgs2,Skil,Socs3,Tlr2,Tnf,Tnfaip3,Tnip1,Zc3h12a
GO:0070887	cellular response to chemical stimulus	25	2.29E-66	Atp7a,Birc3,Ccrl2,Cd69,Cdc42ep2,Cflar,Ehd1,Fas,Herpud1,Icam1,Il10ra,Il1b,Klf2,Nfe2l2,Nfkbia,Pdgfb,Ptger4,Ptgs2,Skil,Socs3,Tlr2,Tnf,Tnfaip3,Tnip1,Zc3h12a
GO:0010646	regulation of cell communication	25	1.58E-65	Bcl3,Birc3,Cdc42ep2,Cflar,Dusp6,Fas,Gadd45b,Hcar2,Herpud1,Icam1,Ier3,Il1b,Nfe2l2,Nfkbia,Pdgfb,Ptger4,Ptgs2,Skil,Socs3,Tlr2,Tnf,Tnfaip3,Tnip1,Traf1,Zc3h12a
GO:0010604	positive regulation of macromolecule metabolic process	24	2.29E-64	Bcl3,Birc3,Cflar,Ell2,Fam46c,Fas,Gadd45b,Herpud1,Icam1,Icosl,Ier3,Il1b,Klf2,Nfe2l2,Nfkbia,Pdgfb,Ptger4,Ptgs2,Rnf19b,Tlr2,Tnf,Tnfaip3,Tnip1,Zc3h12a
GO:0071310	cellular response to organic substance	24	2.29E-64	Atp7a,Birc3,Ccrl2,Cdc42ep2,Cflar,Ehd1,Fas,Herpud1,Icam1,Il10ra,Il1b,Klf2,Nfe2l2,Nfkbia,Pdgfb,Ptger4,Ptgs2,Skil,Socs3,Tlr2,Tnf,Tnfaip3,Tnip1,Zc3h12a
GO:0009966	regulation of signal transduction	24	1.82E-63	Bcl3,Birc3,Cdc42ep2,Cflar,Dusp6,Fas,Gadd45b,Herpud1,Icam1,Ier3,Il1b,Nfe2l2,Nfkbia,Pdgfb,Ptger4,Ptgs2,Skil,Socs3,Tlr2,Tnf,Tnfaip3,Tnip1,Traf1,Zc3h12a
GO:0071704	organic substance metabolic process	24	1.82E-63	Atp7a,Bcl3,Birc3,Cflar,Dusp6,Ell2,Fas,Herpud1,Icosl,Il1b,Klf2,Nfe2l2,Nfkbiz,Pdgfb,Ptger4,Ptgs2,Rnf19b,Socs3,Tlr2,Tnf,Tnfaip3,Tnip1,Zc3h12a,Zc3h12c
GO:0007165	signal transduction	24	1.18E-62	Atp7a,Bcl3,Ccrl2,Cd83,Cdc42ep2,Fas,Hcar2,Herpud1,Icosl,Ier3,Il10ra,Il1b,Nfe2l2,Nfkbia,Nfkbiz,Pdgfb,Ptger4,Skil,Socs3,Tlr2,Tnf,Tnip1,Traf1,Zc3h12a

GO:0065008	regulation of biological quality	23	1.76E-61	Atp7a,Cdc42ep2,Ehd1,Fam46c,Fas,Hcar2,Herpud1,Icam1,Ier3,Il10ra,Il1b,Klf2,Nfe2l2,Nfkbia,Pdgfb,Ptger4,Ptgs2,Skil,Slc31a2,Tlr2,Tnf,Tnfaip3,Zc3h12a
GO:0044238	primary metabolic process	23	1.43E-60	Atp7a,Bcl3,Birc3,Cflar,Dusp6,Ell2,Herpud1,Icosl,Il1b,Klf2,Nfe2l2,Nfkbiz,Pdgfb,Ptger4,Ptgs2,Rnf19b,Socs3,Tlr2,Tnf,Tnfaip3,Tnip1,Zc3h12a,Zc3h12c
GO:0051173	positive regulation of nitrogen compound metabolic process	23	9.31E-60	Bcl3,Birc3,Cflar,Ell2,Fas,Gadd45b,Herpud1,Icam1,Icosl,Ier3,Il1b,Klf2,Nfe2l2,Nfkbia,Pdgfb,Ptger4,Ptgs2,Rnf19b,Tlr2,Tnf,Tnfaip3,Tnip1,Zc3h12a
GO:0048856	anatomical structure development	23	5.05E-59	Atp7a,Bcl3,Cflar,Dusp6,Ehd1,Fas,Gadd45b,Icam1,Icosl,Il1b,Klf2,Nfe2l2,Nfkbia,Pdgfb,Ptger4,Ptgs2,Rilpl2,Skil,Socs3,Tlr2,Tnf,Tnfaip3,Zc3h12a
GO:0044237	cellular metabolic process	22	1.39E-58	Atp7a,Bcl3,Birc3,Dusp6,Ell2,Herpud1,Icosl,Il1b,Klf2,Nfe2l2,Nfkbiz,Pdgfb,Ptger4,Ptgs2,Rnf19b,Socs3,Tlr2,Tnf,Tnfaip3,Tnip1,Zc3h12a,Zc3h12c
GO:0007275	multicellular organism development	22	7.28E-57	Atp7a,Bcl3,Cflar,Dusp6,Ehd1,Fas,Gadd45b,Icam1,Icosl,Il1b,Klf2,Nfe2l2,Nfkbia,Pdgfb,Ptger4,Ptgs2,Skil,Socs3,Tlr2,Tnf,Tnfaip3,Zc3h12a
GO:0010468	regulation of gene expression	22	3.82E-56	Atp7a,Bcl3,Birc3,Cflar,Ell2,Fam46c,Fas,Icam1,Il1b,Klf2,Nfe2l2,Nfkbia,Nfkbiz,Pdgfb,Ptger4,Skil,Tlr2,Tnf,Tnfaip3,Tnip1,Traf1,Zc3h12a
GO:0002376	immune system process	21	1.12E-55	Atp7a,Bcl3,Cd83,Cdc42ep2,Fas,Hcar2,Icam1,Icosl,Il1b,Klf2,Nfkbia,Nfkbiz,Pdgfb,Ptger4,Rnf19b,Skil,Tlr2,Tnf,Tnfaip3,Tnip1,Zc3h12a
GO:0050793	regulation of developmental process	21	1.12E-55	Cd83,Cdc42ep2,Cflar,Dusp6,Ehd1,Fas,Icam1,Il1b,Klf2,Nfe2l2,Nfkbia,Nfkbiz,Pdgfb,Ptger4,Ptgs2,Skil,Socs3,Tlr2,Tnf,Tnfaip3,Zc3h12a
GO:0051247	positive regulation of protein metabolic process	21	1.12E-55	Bcl3,Birc3,Cflar,Fas,Gadd45b,Herpud1,Icam1,Icosl,Ier3,Il1b,Klf2,Nfkbia,Pdgfb,Ptger4,Ptgs2,Rnf19b,Tlr2,Tnf,Tnfaip3,Tnip1,Zc3h12a
GO:0051246	regulation of protein metabolic process	23	1.21E-55	Bcl3,Birc3,Cflar,Dusp6,Fas,Gadd45b,Herpud1,Icam1,Icosl,Ier3,Il1b,Klf2,Nfkbia,Pdgfb,Ptger4,Ptgs2,Rnf19b,Socs3,Tlr2,Tnf,Tnfaip3,Tnip1,Zc3h12a

GO:0051239	regulation of multicellular organismal process	22	6.47E-55	Bcl3,Cd83,Cflar,Dusp6,Ehd1,Fas,Hcar2,Icam1,Icosl,Il1b,Klf2,Nfe2l2,Nfkbia,Nfkbiz,Pdgfb,Ptger4,Ptgs2,Skil,Tlr2,Tnf,Tnfaip3,Zc3h12a
GO:0042981	regulation of apoptotic process	20	8.09E-53	Atp7a,Bcl3,Birc3,Cflar,Dusp6,Fas,Gadd45b,Hcar2,Herpud1,Icam1,Ier3,Il1b,Nfe2l2,Ptgs2,Skil,Socs3,Tnf,Tnfaip3,Traf1,Zc3h12a
GO:0048731	system development	20	8.09E-53	Atp7a,Bcl3,Cflar,Ehd1,Fas,Icam1,Icosl,Il1b,Klf2,Nfe2l2,Nfkbia,Pdgfb,Ptger4,Ptgs2,Skil,Socs3,Tlr2,Tnf,Tnfaip3,Zc3h12a
GO:0065009	regulation of molecular function	20	8.09E-53	Atp7a,Bcl3,Birc3,Cdc42ep2,Cflar,Dusp6,Fas,Gadd45b,Herpud1,Icam1,Il1b,Nfkbia,Pdgfb,Ptgs2,Socs3,Tlr2,Tnf,Tnfaip3,Traf1,Zc3h12a
GO:0009892	negative regulation of metabolic process	20	5.67E-52	Bcl3,Birc3,Cflar,Dusp6,Fam46c,Gadd45b,Hcar2,Herpud1,Ier3,Il1b,Klf2,Pdgfb,Ptger4,Ptgs2,Skil,Socs3,Tnf,Tnfaip3,Tnip1,Zc3h12a
GO:0044260	cellular macromolecule metabolic process	20	3.33E-51	Atp7a,Bcl3,Birc3,Dusp6,Ell2,Herpud1,Icosl,Il1b,Klf2,Nfe2l2,Nfkbiz,Pdgfb,Ptger4,Rnf19b,Socs3,Tlr2,Tnf,Tnfaip3,Tnip1,Zc3h12a
GO:1902531	regulation of intracellular signal transduction	20	3.33E-51	Bcl3,Birc3,Cflar,Dusp6,Fas,Gadd45b,Herpud1,Icam1,Il1b,Nfe2l2,Pdgfb,Ptger4,Ptgs2,Skil,Socs3,Tlr2,Tnf,Tnfaip3,Tnip1,Zc3h12a
GO:0043170	macromolecule metabolic process	23	1.01E-50	Atp7a,Bcl3,Birc3,Cflar,Dusp6,Ell2,Fas,Herpud1,Icosl,Il1b,Klf2,Nfe2l2,Nfkbiz,Pdgfb,Ptger4,Rnf19b,Socs3,Tlr2,Tnf,Tnfaip3,Tnip1,Zc3h12a,Zc3h12c
GO:0010556	regulation of macromolecule biosynthetic process	19	2.26E-48	Bcl3,Cflar,Ell2,Icam1,Icosl,Il1b,Klf2,Nfe2l2,Nfkbia,Nfkbiz,Pdgfb,Ptger4,Skil,Tlr2,Tnf,Tnfaip3,Tnip1,Traf1,Zc3h12a
GO:0006807	nitrogen compound metabolic process	22	2.49E-48	Atp7a,Bcl3,Birc3,Cflar,Dusp6,Ell2,Herpud1,Icosl,Il1b,Klf2,Nfe2l2,Nfkbiz,Pdgfb,Ptger4,Rnf19b,Socs3,Tlr2,Tnf,Tnfaip3,Tnip1,Zc3h12a,Zc3h12c
GO:0080134	regulation of response to stress	19	1.04E-47	Atp7a,Birc3,Fas,Gadd45b,Herpud1,Ier3,Il1b,Nfe2l2,Nfkbia,Pdgfb,Ptger4,Ptgs2,Skil,Socs3,Tlr2,Tnf,Tnfaip3,Tnip1,Zc3h12a

GO:0048585	negative regulation of response to stimulus	18	3.90E-47	Bcl3,Cflar,Dusp6,Herpud1,Icam1,Ier3,Il1b,Nfe2l2,Nfkbia,Pdgfb,Ptger4,Ptgs2,Skil,Socs3,Tnf,Tnfaip3,Tnip1,Zc3h12a
GO:0051172	negative regulation of nitrogen compound metabolic process	18	2.68E-46	Bcl3,Birc3,Cflar,Dusp6,Fam46c,Gadd45b,Herpud1,Ier3,Il1b,Klf2,Pdgfb,Ptgs2,Skil,Socs3,Tnf,Tnfaip3,Tnip1,Zc3h12a
GO:2000026	regulation of multicellular organismal development	18	2.68E-46	Cd83,Cflar,Dusp6,Ehd1,Fas,Il1b,Klf2,Nfe2l2,Nfkbia,Nfkbiz,Pdgfb,Ptger4,Ptgs2,Skil,Tlr2,Tnf,Tnfaip3,Zc3h12a
GO:0006355	regulation of transcription, DNA-templated	17	2.72E-44	Bcl3,Cflar,Ell2,Icam1,Il1b,Klf2,Nfe2l2,Nfkbia,Nfkbiz,Pdgfb,Skil,Tlr2,Tnf,Tnfaip3,Tnip1,Traf1,Zc3h12a
GO:1901700	response to oxygen-containing compound	17	2.72E-44	Atp7a,Cflar,Fas,Icam1,Il10ra,Il1b,Klf2,Nfe2l2,Nfkbia,Pdgfb,Ptger4,Ptgs2,Tlr2,Tnf,Tnfaip3,Tnip1,Zc3h12a
GO:0051240	positive regulation of multicellular organismal process	18	2.78E-43	Bcl3,Cd83,Cflar,Ehd1,Hcar2,Icam1,Icosl,Il1b,Nfe2l2,Nfkbiz,Pdgfb,Ptger4,Ptgs2,Skil,Tlr2,Tnf,Tnfaip3,Zc3h12a
GO:0048584	positive regulation of response to stimulus	18	2.22E-42	Atp7a,Cflar,Fas,Gadd45b,Herpud1,Icam1,Il1b,Nfe2l2,Nfkbia,Nfkbiz,Pdgfb,Ptger4,Ptgs2,Skil,Tlr2,Tnf,Tnip1,Zc3h12a
GO:0045595	regulation of cell differentiation	17	3.61E-42	Cd83,Cflar,Dusp6,Ehd1,Fas,Il1b,Nfe2l2,Nfkbia,Nfkbiz,Pdgfb,Ptger4,Ptgs2,Skil,Socs3,Tlr2,Tnf,Zc3h12a
GO:0010605	negative regulation of macromolecule metabolic process	18	1.42E-41	Bcl3,Birc3,Cflar,Dusp6,Fam46c,Gadd45b,Herpud1,Il1b,Klf2,Pdgfb,Ptger4,Ptgs2,Skil,Socs3,Tnf,Tnfaip3,Tnip1,Zc3h12a

GO:0009968	negative regulation of signal transduction	16	1.61E-41	Bcl3,Cflar,Dusp6,Herpud1,Icam1,Ier3,Il1b,Nfe2l2,Nfkbia,Ptgs2,Skil,Socs3,Tnf,Tnfaip3,Tnip1,Zc3h12a
GO:0051179	localization	16	1.61E-41	Atp7a,Bcl3,Ehd1,Herpud1,Icam1,Ier3,Il1b,Nfkbia,Nfkbie,Pdgfb,Rilpl2,Rnf19b,Slc31a2,Tlr2,Tnf,Tnfaip3
GO:0016043	cellular component organization	16	9.92E-41	Atp7a,Bcl3,Birc3,Cdc42ep2,Cflar,Ehd1,Fas,Icam1,Ier3,Klf2,Pdgfb,Rilpl2,Skil,Tlr2,Tnf,Zc3h12a
GO:0006952	defense response	16	2.02E-39	Bcl3,Ccrl2,Cd83,Cdc42ep2,Fas,Icam1,Il1b,Nfe2l2,Nfkbiz,Ptgs2,Rnf19b,Tlr2,Tnf,Tnfaip3,Tnip1,Zc3h12a
GO:0002682	regulation of immune system process	16	7.20E-39	Birc3,Cd83,Fas,Hcar2,Icam1,Icosl,Il1b,Nfe2l2,Nfkbia,Nfkbiz,Ptger4,Tlr2,Tnf,Tnfaip3,Tnip1,Zc3h12a
GO:0051094	positive regulation of developmental process	15	9.91E-39	Cd83,Cflar,Ehd1,Il1b,Nfe2l2,Nfkbiz,Pdgfb,Ptger4,Ptgs2,Skil,Socs3,Tlr2,Tnf,Tnfaip3,Zc3h12a
GO:0051128	regulation of cellular component organization	15	9.91E-39	Atp7a,Cdc42ep2,Cflar,Ehd1,Fas,Icam1,Ier3,Il10ra,Il1b,Nfe2l2,Pdgfb,Ptger4,Skil,Tlr2,Tnf
GO:1901701	cellular response to oxygen-containing compound	15	9.91E-39	Atp7a,Cflar,Icam1,Il1b,Klf2,Nfe2l2,Nfkbia,Pdgfb,Ptger4,Ptgs2,Tlr2,Tnf,Tnfaip3,Tnip1,Zc3h12a
GO:0009605	response to external stimulus	16	2.22E-38	Bcl3,Ccrl2,Fas,Ier3,Il10ra,Il1b,Nfe2l2,Nfkbia,Pdgfb,Ptger4,Ptgs2,Tlr2,Tnf,Tnfaip3,Tnip1,Zc3h12a
GO:0051049	regulation of transport	15	5.57E-38	Atp7a,Ehd1,Hcar2,Icam1,Ier3,Il1b,Nfe2l2,Nfkbia,Pdgfb,Ptger4,Ptgs2,Slc31a2,Tlr2,Tnf,Zc3h12a
GO:0051704	multi-organism process	15	5.57E-38	Bcl3,Fas,Icam1,Ier3,Il10ra,Il1b,Nfkbia,Ptger4,Ptgs2,Skil,Tlr2,Tnf,Tnfaip3,Tnip1,Zc3h12a

GO:0042325	regulation of phosphorylation	18	1.36E-37	Atp7a,Birc3,Cflar,Dusp6,Fas,Gadd45b,Icam1,Ier3,Il1b,Pdgfb,Ptger4,Ptgs2,Socs3,Tlr2,Tnf,Tnfaip3,Tnip1,Zc3h12a
GO:0051252	regulation of RNA metabolic process	18	3.68E-37	Bcl3,Cflar,Ell2,Fam46c,Icam1,Il1b,Klf2,Nfe2l2,Nfkbia,Nfkbiz,Pdgfb,Skil,Tlr2,Tnf,Tnfaip3,Tnip1,Traf1,Zc3h12a
GO:0044092	negative regulation of molecular function	14	5.75E-36	Atp7a,Birc3,Cflar,Dusp6,Gadd45b,Herpud1,Il1b,Nfkbia,Pdgfb,Ptgs2,Socs3,Tnf,Tnfaip3,Zc3h12a
GO:0050790	regulation of catalytic activity	15	3.13E-35	Atp7a,Birc3,Cdc42ep2,Cflar,Dusp6,Fas,Gadd45b,Herpud1,Icam1,Il1b,Pdgfb,Ptgs2,Socs3,Tnf,Tnfaip3
GO:0019538	protein metabolic process	16	4.94E-35	Atp7a,Birc3,Cflar,Dusp6,Herpud1,Il1b,Nfe2l2,Pdgfb,Ptger4,Rnf19b,Socs3,Tlr2,Tnf,Tnfaip3,Tnip1,Zc3h12a
GO:0048513	animal organ development	15	8.16E-35	Atp7a,Bcl3,Cflar,Fas,Icam1,Klf2,Nfkbia,Pdgfb,Ptger4,Ptgs2,Skil,Socs3,Tlr2,Tnf,Tnfaip3
GO:0006357	regulation of transcription by RNA polymerase II	13	3.39E-33	Bcl3,Ell2,Il1b,Klf2,Nfe2l2,Nfkbia,Nfkbiz,Pdgfb,Skil,Tlr2,Tnf,Tnip1,Zc3h12a
GO:0006464	cellular protein modification process	13	3.39E-33	Atp7a,Birc3,Dusp6,Il1b,Nfe2l2,Pdgfb,Ptger4,Rnf19b,Socs3,Tlr2,Tnf,Tnfaip3,Zc3h12a
GO:0006955	immune response	13	3.39E-33	Atp7a,Bcl3,Cd83,Cdc42ep2,Fas,Icam1,Icosl,Il1b,Ptger4,Rnf19b,Tlr2,Tnf,Tnfaip3
GO:0009653	anatomical structure morphogenesis	13	3.39E-33	Atp7a,Bcl3,Cflar,Klf2,Pdgfb,Ptger4,Ptgs2,Rilpl2,Skil,Socs3,Tlr2,Tnf,Zc3h12a
GO:0010557	positive regulation of macromolecule biosynthetic process	13	3.39E-33	Bcl3,Ell2,Icosl,Il1b,Klf2,Nfe2l2,Nfkbia,Pdgfb,Ptger4,Tlr2,Tnf,Tnip1,Zc3h12a
GO:0051234	establishment of localization	13	3.39E-33	Atp7a,Bcl3,Ehd1,Herpud1,Ier3,Nfkbia,Nfkbie,Rilpl2,Rnf19b,Slc31a2,Tlr2,Tnf,Tnfaip3

GO:0051707	response to other organism	13	3.39E-33	Bcl3,Fas,Ier3,Il10ra,Il1b,Nfkbia,Ptger4,Ptgs2,Tlr2,Tnf,Tnfaip3,Tnip1,Zc3h12a
GO:2000377	regulation of reactive oxygen species metabolic process	13	3.39E-33	Birc3,Cflar,Icam1,Ier3,Il1b,Klf2,Nfe2l2,Pdgfb,Ptger4,Ptgs2,Tlr2,Tnf,Zc3h12a
GO:0007166	cell surface receptor signaling pathway	13	1.64E-32	Ccrl2,Fas,Hcar2,Il10ra,Il1b,Nfkbia,Nfkbiz,Pdgfb,Skil,Socs3,Tlr2,Tnf,Zc3h12a
GO:0051241	negative regulation of multicellular organismal process	13	1.64E-32	Bcl3,Cd83,Il1b,Klf2,Nfe2l2,Nfkbia,Pdgfb,Ptger4,Ptgs2,Tlr2,Tnf,Tnfaip3,Zc3h12a
GO:0044267	cellular protein metabolic process	14	3.17E-32	Atp7a,Birc3,Dusp6,Herpud1,Il1b,Nfe2l2,Pdgfb,Ptger4,Rnf19b,Socs3,Tlr2,Tnf,Tnfaip3,Zc3h12a
GO:0045597	positive regulation of cell differentiation	13	2.36E-31	Cd83,Cflar,Ehd1,Il1b,Nfe2l2,Nfkbiz,Ptger4,Ptgs2,Skil,Socs3,Tlr2,Tnf,Zc3h12a
GO:0010628	positive regulation of gene expression	13	7.39E-31	Bcl3,Ell2,Fam46c,Il1b,Klf2,Nfe2l2,Nfkbia,Pdgfb,Ptger4,Tlr2,Tnf,Tnip1,Zc3h12a
GO:0071345	cellular response to cytokine stimulus	15	1.27E-30	Birc3,Ccrl2,Cdc42ep2,Fas,Icam1,Il10ra,Il1b,Klf2,Nfe2l2,Nfkbia,Pdgfb,Socs3,Tlr2,Tnf,Zc3h12a
GO:0002684	positive regulation of immune system process	12	1.69E-30	Cd83,Hcar2,Icam1,Icosl,Il1b,Nfkbia,Nfkbiz,Ptger4,Tlr2,Tnf,Tnip1,Zc3h12a
GO:0006810	transport	12	8.20E-30	Atp7a,Bcl3,Ehd1,Herpud1,Ier3,Nfkbia,Nfkbie,Rilpl2,Rnf19b,Slc31a2,Tlr2,Tnf
GO:2001233	regulation of apoptotic signaling pathway	12	8.20E-30	Cflar,Fas,Herpud1,Icam1,Ier3,Il1b,Nfe2l2,Ptgs2,Skil,Tnf,Tnfaip3,Traf1
GO:0006915	apoptotic process	13	2.91E-29	Atp7a,Bcl3,Birc3,Cflar,Fas,Gadd45b,Hcar2,Ier3,Il1b,Tnf,Tnfaip3,Traf1,Zc3h12a

GO:0009617	response to bacterium	12	3.19E-29	Bcl3,Fas,Il10ra,Il1b,Nfkbia,Ptger4,Ptgs2,Tlr2,Tnf,Tnfaip3,Tnip1,Zc3h12a
GO:0044093	positive regulation of molecular function	12	3.19E-29	Atp7a,Cdc42ep2,Cflar,Fas,Gadd45b,Herpud1,Icam1,Il1b,Pdgfb,Tlr2,Tnf,Traf1
GO:0006954	inflammatory response	12	1.05E-28	Ccrl2,Fas,Icam1,Il1b,Nfe2l2,Nfkbiz,Ptgs2,Tlr2,Tnf,Tnfaip3,Tnip1,Zc3h12a
GO:0051248	negative regulation of protein metabolic process	13	4.82E-28	Bcl3,Birc3,Cflar,Dusp6,Gadd45b,Herpud1,Il1b,Ptgs2,Socs3,Tnf,Tnfaip3,Tnip1,Zc3h12a
GO:0014070	response to organic cyclic compound	11	8.71E-28	Cd83,Cflar,Dusp6,Fas,Il1b,Klf2,Nfkbia,Pdgfb,Ptgs2,Tnf,Zc3h12a
GO:0043086	negative regulation of catalytic activity	11	8.71E-28	Atp7a,Birc3,Cflar,Dusp6,Gadd45b,Herpud1,Il1b,Ptgs2,Socs3,Tnf,Tnfaip3
GO:0045944	positive regulation of transcription by RNA polymerase II	11	8.71E-28	Bcl3,Ell2,Il1b,Klf2,Nfe2l2,Nfkbia,Pdgfb,Tlr2,Tnf,Tnip1,Zc3h12a
GO:0050727	regulation of inflammatory response	11	8.71E-28	Ier3,Il1b,Nfkbia,Ptger4,Ptgs2,Socs3,Tlr2,Tnf,Tnfaip3,Tnip1,Zc3h12a
GO:0051093	negative regulation of developmental process	11	8.71E-28	Cflar,Il1b,Klf2,Nfe2l2,Nfkbia,Pdgfb,Ptger4,Skil,Tlr2,Tnf,Zc3h12a
GO:0010647	positive regulation of cell communication	13	2.66E-27	Cflar,Fas,Gadd45b,Hcar2,Icam1,Il1b,Pdgfb,Ptger4,Ptgs2,Skil,Tlr2,Tnf,Zc3h12a
GO:0009967	positive regulation of signal transduction	11	3.78E-27	Cflar,Fas,Gadd45b,Icam1,Il1b,Pdgfb,Ptger4,Skil,Tlr2,Tnf,Zc3h12a

GO:0043408	regulation of MAPK cascade	11	3.78E-27	Cflar,Dusp6,Fas,Gadd45b,Icam1,Il1b,Pdgfb,Tlr2,Tnf,Tnip1,Zc3h12a
GO:0048468	cell development	11	3.78E-27	Atp7a,Cflar,Ehd1,Fas,Icam1,Il1b,Klf2,Pdgfb,Rilpl2,Skil,Tlr2
GO:0051050	positive regulation of transport	11	3.78E-27	Ehd1,Hcar2,Il1b,Nfe2l2,Nfkbia,Pdgfb,Ptger4,Ptgs2,Tlr2,Tnf,Zc3h12a
GO:0051130	positive regulation of cellular component organization	11	3.78E-27	Atp7a,Cdc42ep2,Cflar,Ehd1,Fas,Icam1,Il1b,Nfe2l2,Pdgfb,Skil,Tnf
GO:0001817	regulation of cytokine production	11	1.34E-26	Bcl3,Cd83,Icosl,Il1b,Klf2,Ptger4,Ptgs2,Tlr2,Tnf,Tnfaip3,Zc3h12a
GO:0001934	positive regulation of protein phosphorylation	11	1.34E-26	Cflar,Fas,Gadd45b,Icam1,Il1b,Pdgfb,Ptger4,Ptgs2,Tlr2,Tnf,Zc3h12a
GO:0009719	response to endogenous stimulus	11	1.34E-26	Atp7a,Cflar,Ehd1,Fas,Klf2,Nfe2l2,Ptger4,Ptgs2,Skil,Tlr2,Tnf
GO:0010243	response to organonitrogen compound	11	4.19E-26	Atp7a,Cflar,Herpud1,Il1b,Klf2,Nfe2l2,Nfkbia,Ptgs2,Tlr2,Tnf,Tnfaip3
GO:0042127	regulation of cell proliferation	12	7.28E-26	Atp7a,Cflar,Fas,Icosl,Il1b,Nfkbia,Pdgfb,Ptger4,Ptgs2,Tlr2,Tnf,Tnfaip3
GO:0042493	response to drug	14	8.87E-26	Cd69,Cflar,Dusp6,Il1b,Klf2,Nfe2l2,Nfkbia,Nfkbie,Pdgfb,Ptgs2,Tlr2,Tnf,Tnfaip3, Zc3h12a
GO:0009894	regulation of catabolic process	10	3.99E-25	Fam46c,Hcar2,Herpud1,Ier3,Il1b,Rnf19b,Tlr2,Tnf,Tnfaip3,Zc3h12a
GO:0071495	cellular response to endogenous stimulus	10	3.99E-25	Atp7a,Cflar,Ehd1,Klf2,Nfe2l2,Ptger4,Ptgs2,Skil,Tlr2,Tnf

GO:1902533	positive regulation of intracellular signal transduction	10	3.99E-25	Cflar,Gadd45b,Icam1,Il1b,Pdgfb,Ptger4,Skil,Tlr2,Tnf,Zc3h12a
GO:0035556	intracellular signal transduction	10	1.69E-24	Bcl3,Cdc42ep2,Ier3,Il1b,Nfkbia,Pdgfb,Ptger4,Socs3,Tlr2,Tnf
GO:0044085	cellular component biogenesis	10	1.69E-24	Atp7a,Birc3,Cflar,Ehd1,Fas,Pdgfb,Rilpl2,Skil,Tlr2,Zc3h12a
GO:0007399	nervous system development	10	5.79E-24	Atp7a,Cflar,Ehd1,Fas,Il1b,Nfe2l2,Pdgfb,Skil,Tlr2,Zc3h12a
GO:0009890	negative regulation of biosynthetic process	10	5.79E-24	Bcl3,Cflar,Ier3,Il1b,Klf2,Pdgfb,Ptger4,Skil,Tnf,Zc3h12a
GO:0080135	regulation of cellular response to stress	10	5.79E-24	Fas,Gadd45b,Herpud1,Ier3,Il1b,Nfe2l2,Ptgs2,Skil,Tnf,Zc3h12a
GO:1901576	organic substance biosynthetic process	10	5.79E-24	Atp7a,Bcl3,Ell2,Il1b,Klf2,Nfe2l2,Nfkbiz,Ptgs2,Tnf,Tnip1
GO:1902532	negative regulation of intracellular signal transduction	10	5.79E-24	Bcl3,Dusp6,Herpud1,Il1b,Nfe2l2,Ptgs2,Socs3,Tnfaip3,Tnip1,Zc3h12a
GO:0045936	negative regulation of phosphate metabolic process	11	1.15E-23	Birc3,Dusp6,Gadd45b,Ier3,Il1b,Pdgfb,Socs3,Tnf,Tnfaip3,Tnip1,Zc3h12a
GO:0002520	immune system development	9	1.94E-22	Atp7a,Bcl3,Fas,Icosl,Klf2,Ptger4,Tlr2,Tnf,Tnfaip3

GO:0006139	nucleobase-containing compound metabolic process	9	1.94E-22	Atp7a,Bcl3,Ell2,Icosl,Klf2,Nfe2l2,Nfkbiz,Zc3h12a,Zc3h12c
GO:0009636	response to toxic substance	9	1.94E-22	Atp7a,Fas,Klf2,Nfe2l2,Pdgfb,Ptgs2,Tnf,Tnfaip3,Zc3h12a
GO:0009888	tissue development	9	1.94E-22	Atp7a,Cflar,Icam1,Klf2,Pdgfb,Ptgs2,Rilpl2,Skil,Socs3
GO:0030335	positive regulation of cell migration	9	1.94E-22	Atp7a,Icam1,Il1b,Nfe2l2,Pdgfb,Ptger4,Ptgs2,Tlr2,Zc3h12a
GO:0044249	cellular biosynthetic process	9	1.94E-22	Atp7a,Bcl3,Ell2,Il1b,Klf2,Nfe2l2,Nfkbiz,Ptgs2,Tnip1
GO:0045184	establishment of protein localization	9	1.94E-22	Bcl3,Ehd1,Herpud1,Nfkbia,Rilpl2,Rnf19b,Tlr2,Tnf,Tnfaip3
GO:0051051	negative regulation of transport	9	1.94E-22	Atp7a,Icam1,Ier3,Il1b,Ptger4,Ptgs2,Tlr2,Tnf,Zc3h12a
GO:0051726	regulation of cell cycle	9	1.94E-22	Ccng2,Gadd45b,Ier3,Il1b,Pdgfb,Ptgs2,Skil,Tnf,Tnfaip3
GO:0071702	organic substance transport	9	1.94E-22	Bcl3,Ehd1,Herpud1,Nfkbia,Nfkbie,Rilpl2,Rnf19b,Tlr2,Tnf
GO:2001234	negative regulation of apoptotic signaling pathway	9	1.94E-22	Cflar,Herpud1,Icam1,Ier3,Il1b,Nfe2l2,Ptgs2,Tnf,Tnfaip3
GO:0001505	regulation of neurotransmitter levels	9	6.93E-22	Atp7a,Icam1,Il1b,Klf2,Ptger4,Ptgs2,Tlr2,Tnf,Zc3h12a
GO:0009059	macromolecule biosynthetic process	9	6.93E-22	Atp7a,Bcl3,Ell2,Il1b,Klf2,Nfe2l2,Nfkbiz,Tnf,Tnip1

GO:0043085	positive regulation of catalytic activity	9	6.93E-22	Atp7a,Cdc42ep2,Cflar,Fas,Gadd45b,Icam1,Il1b,Pdgfb,Tnf
GO:0048699	generation of neurons	9	6.93E-22	Atp7a,Cflar,Ehd1,Fas,Il1b,Nfe2l2,Pdgfb,Skil,Tlr2
GO:0051090	regulation of DNA-binding transcription factor activity	9	6.93E-22	Cflar,Icam1,Il1b,Nfkbia,Tlr2,Tnf,Tnfaip3,Traf1,Zc3h12a
GO:2000379	positive regulation of reactive oxygen species metabolic process	9	2.15E-21	Icam1,Il1b,Klf2,Nfe2l2,Pdgfb,Ptgs2,Tlr2,Tnf,Zc3h12a
GO:0043065	positive regulation of apoptotic process	9	5.95E-21	Dusp6,Fas,Gadd45b,Hcar2,Il1b,Ptgs2,Skil,Tnf,Zc3h12a
GO:0045934	negative regulation of nucleobase-containing compound metabolic process	9	5.95E-21	Bcl3,Fam46c,Ier3,Il1b,Klf2,Pdgfb,Skil,Tnf,Zc3h12a
GO:0009628	response to abiotic stimulus	9	1.47E-20	Bcl3,Cflar,Fas,Il1b,Nfe2l2,Nfkbia,Ptger4,Ptgs2,Tnf
GO:0008284	positive regulation of cell proliferation	9	7.08E-20	Atp7a,Cflar,Icosl,Il1b,Pdgfb,Ptger4,Ptgs2,Tnf,Tnfaip3
GO:0050776	regulation of immune response	9	7.08E-20	Birc3,Il1b,Nfkbia,Nfkbiz,Tlr2,Tnf,Tnfaip3,Tnip1,Zc3h12a
GO:0015031	protein transport	8	8.02E-20	Bcl3,Ehd1,Herpud1,Nfkbia,Rilpl2,Rnf19b,Tlr2,Tnf
GO:0019221	cytokine-mediated signaling pathway	8	8.02E-20	Ccrl2,Fas,Il10ra,Il1b,Nfkbia,Pdgfb,Socs3,Tnf

GO:0050865	regulation of cell activation	8	8.02E-20	Cd83,Fas,Icosl,Il1b,Nfkbiz,Pdgfb,Tnfaip3,Zc3h12a
GO:0051336	regulation of hydrolase activity	8	8.02E-20	Birc3,Cdc42ep2,Cflar,Fas,Herpud1,Icam1,Ptgs2,Tnf
GO:0090304	nucleic acid metabolic process	8	8.02E-20	Bcl3,Ell2,Icosl,Klf2,Nfe2l2,Nfkbiz,Zc3h12a,Zc3h12c
GO:1903706	regulation of hemopoiesis	8	8.02E-20	Cd83,Fas,Il1b,Nfe2l2,Nfkbia,Nfkbiz,Tnf,Zc3h12a
GO:0003008	system process	10	1.30E-19	Cflar,Fas,Icam1,Ier3,Il1b,Pdgfb,Ptger4,Ptgs2,Tlr2,Tnf
GO:0071705	nitrogen compound transport	9	2.53E-19	Bcl3,Ehd1,Herpud1,Nfkbia,Nfkbie,Rilpl2,Rnf19b,Tlr2,Tnf
GO:0010035	response to inorganic substance	8	2.79E-19	Atp7a,Cflar,Fas,Klf2,Nfe2l2,Ptgs2,Tnfaip3,Zc3h12a
GO:0010629	negative regulation of gene expression	8	2.79E-19	Bcl3,Birc3,Il1b,Klf2,Pdgfb,Skil,Tnf,Zc3h12a
GO:0043410	positive regulation of MAPK cascade	8	2.79E-19	Cflar,Gadd45b,Icam1,Il1b,Pdgfb,Tlr2,Tnf,Zc3h12a
GO:0044057	regulation of system process	8	2.79E-19	Hcar2,Icam1,Il1b,Pdgfb,Ptger4,Ptgs2,Tnf,Zc3h12a
GO:0051253	negative regulation of RNA metabolic process	8	2.79E-19	Bcl3,Fam46c,Il1b,Klf2,Pdgfb,Skil,Tnf,Zc3h12a
GO:0071396	cellular response to lipid	8	2.79E-19	Cflar,Il1b,Nfkbia,Ptger4,Tnf,Tnfaip3,Tnip1,Zc3h12a
GO:1901342	regulation of vasculature development	8	2.79E-19	Cflar,Il1b,Klf2,Nfe2l2,Pdgfb,Ptger4,Ptgs2,Zc3h12a

GO:0001819	positive regulation of cytokine production	8	7.99E-19	Bcl3,Cd83,Icosl,Il1b,Ptger4,Ptgs2,Tlr2,Tnf
GO:0006996	organelle organization	8	7.99E-19	Atp7a,Cdc42ep2,Cflar,Ehd1,Ier3,Pdgfb,Rilpl2,Tnf
GO:0009896	positive regulation of catabolic process	8	7.99E-19	Herpud1,Ier3,Il1b,Rnf19b,Tlr2,Tnf,Tnfaip3,Zc3h12a
GO:0010558	negative regulation of macromolecule biosynthetic process	8	7.99E-19	Bcl3,Il1b,Klf2,Pdgfb,Ptger4,Skil,Tnf,Zc3h12a
GO:0030097	hemopoiesis	8	7.99E-19	Atp7a,Bcl3,Fas,Klf2,Ptger4,Tlr2,Tnf,Tnfaip3
GO:0071407	cellular response to organic cyclic compound	8	7.99E-19	Cflar,Il1b,Klf2,Nfkbia,Pdgfb,Ptgs2,Tnf,Zc3h12a
GO:0035690	cellular response to drug	10	9.99E-19	Cd69,Cflar,Il1b,Klf2,Nfe2l2,Pdgfb,Ptgs2,Tnf,Tnfaip3,Zc3h12a
GO:0001101	response to acid chemical	8	2.01E-18	Atp7a,Klf2,Pdgfb,Ptger4,Ptgs2,Tlr2,Tnf,Zc3h12a
GO:0045862	positive regulation of proteolysis	8	2.01E-18	Cflar,Fas,Herpud1,Il1b,Rnf19b,Tnf,Tnip1,Zc3h12a
GO:0060429	epithelium development	8	2.01E-18	Atp7a,Icam1,Klf2,Pdgfb,Ptgs2,Rilpl2,Skil,Socs3
GO:0001818	negative regulation of cytokine production	8	4.61E-18	Bcl3,Cd83,Klf2,Ptger4,Tlr2,Tnf,Tnfaip3,Zc3h12a
GO:0045596	negative regulation of cell differentiation	8	4.61E-18	Cflar,Il1b,Nfe2l2,Nfkbia,Pdgfb,Skil,Tnf,Zc3h12a

GO:0097237	cellular response to toxic substance	8	4.61E-18	Atp7a,Klf2,Nfe2l2,Pdgfb,Ptgs2,Tnf,Tnfaip3,Zc3h12a
GO:0043066	negative regulation of apoptotic process	15	5.33E-18	Atp7a,Bcl3,Birc3,Cflar,Fas,Herpud1,Icam1,Ier3,Il1b,Nfe2l2,Ptgs2,Socs3,Tnf,Tnfaip3,Zc3h12a
GO:0002521	leukocyte differentiation	7	3.24E-17	Atp7a,Bcl3,Fas,Ptger4,Tlr2,Tnf,Tnfaip3
GO:0044087	regulation of cellular component biogenesis	7	3.24E-17	Atp7a,Cdc42ep2,Fas,Icam1,Ptger4,Tlr2,Tnf
GO:0050778	positive regulation of immune response	7	3.24E-17	Il1b,Nfkbia,Nfkbiz,Tlr2,Tnf,Tnip1,Zc3h12a
GO:0071219	cellular response to molecule of bacterial origin	7	3.24E-17	Il1b,Nfkbia,Tlr2,Tnf,Tnfaip3,Tnip1,Zc3h12a
GO:0071417	cellular response to organonitrogen compound	7	3.24E-17	Atp7a,Cflar,Klf2,Nfe2l2,Ptgs2,Tlr2,Tnf
GO:0120035	regulation of plasma membrane bounded cell projection organization	7	3.24E-17	Atp7a,Cdc42ep2,Cflar,Ehd1,Icam1,Nfe2l2,Skil
GO:0042592	homeostatic process	12	5.69E-17	Atp7a,Ehd1,Fas,Hcar2,Herpud1,Icam1,Il1b,Klf2,Nfe2l2,Skil,Slc31a2,Tnfaip3
GO:2001236	regulation of extrinsic apoptotic signaling pathway	8	6.17E-17	Cflar,Fas,Icam1,Il1b,Skil,Tnf,Tnfaip3,Traf1

GO:0001933	negative regulation of protein phosphorylation	7	1.02E-16	Dusp6,Gadd45b,Il1b,Socs3,Tnfaip3,Tnip1,Zc3h12a
GO:0045428	regulation of nitric oxide biosynthetic process	7	1.02E-16	Icam1,Il1b,Klf2,Ptgs2,Tlr2,Tnf,Zc3h12a
GO:0045785	positive regulation of cell adhesion	7	1.02E-16	Cd83,Icam1,Icosl,Il1b,Nfkbiz,Ptger4,Tnf
GO:0051249	regulation of lymphocyte activation	7	1.02E-16	Cd83,Fas,Icosl,Il1b,Nfkbiz,Tnfaip3,Zc3h12a
GO:0051960	regulation of nervous system development	7	1.02E-16	Cflar,Ehd1,Il1b,Nfe2l2,Skil,Tlr2,Tnf
GO:0070372	regulation of ERK1 and ERK2 cascade	7	1.02E-16	Cflar,Dusp6,Icam1,Il1b,Pdgfb,Tlr2,Tnip1
GO:0006796	phosphate-containing compound metabolic process	7	2.70E-16	Atp7a,Dusp6,Il1b,Pdgfb,Ptger4,Tlr2,Tnf
GO:0006979	response to oxidative stress	7	2.70E-16	Atp7a,Cflar,Klf2,Nfe2l2,Ptgs2,Tnfaip3,Zc3h12a
GO:0051222	positive regulation of protein transport	7	2.70E-16	Hcar2,Il1b,Ptger4,Ptgs2,Tlr2,Tnf,Zc3h12a
GO:0062012	regulation of small molecule metabolic process	7	2.70E-16	Atp7a,Ier3,Il1b,Pdgfb,Ptger4,Ptgs2,Tnf

GO:1903426	regulation of reactive oxygen species biosynthetic process	8	2.70E-16	Cflar,Icam1,Il1b,Klf2,Ptgs2,Tlr2,Tnf,Zc3h12a
GO:2000113	negative regulation of cellular macromolecule biosynthetic process	7	2.70E-16	Bcl3,Il1b,Klf2,Pdgfb,Skil,Tnf,Zc3h12a
GO:0035295	tube development	7	6.29E-16	Atp7a,Klf2,Pdgfb,Ptger4,Ptgs2,Tnf,Zc3h12a
GO:0043269	regulation of ion transport	7	6.29E-16	Atp7a,Icam1,Il1b,Pdgfb,Ptgs2,Slc31a2,Tnf
GO:0045859	regulation of protein kinase activity	7	6.29E-16	Dusp6,Gadd45b,Il1b,Pdgfb,Socs3,Tnf,Tnfaip3
GO:0048646	anatomical structure formation involved in morphogenesis	7	6.29E-16	Atp7a,Bcl3,Cflar,Ptgs2,Skil,Tlr2,Zc3h12a
GO:0060284	regulation of cell development	7	6.29E-16	Cflar,Ehd1,Il1b,Nfe2l2,Skil,Tlr2,Tnf
GO:0046649	lymphocyte activation	7	2.64E-15	Atp7a,Bcl3,Fas,Icam1,Icosl,Ptger4,Tnfaip3
GO:0001568	blood vessel development	6	1.25E-14	Atp7a,Pdgfb,Ptger4,Ptgs2,Socs3,Zc3h12a
GO:0001944	vasculature development	6	1.25E-14	Atp7a,Pdgfb,Ptger4,Ptgs2,Socs3,Zc3h12a
GO:0022414	reproductive process	6	1.25E-14	Icam1,Il1b,Pdgfb,Ptgs2,Skil,Socs3
GO:0042326	negative regulation of phosphorylation	9	1.25E-14	Birc3,Dusp6,Gadd45b,Ier3,Il1b,Socs3,Tnfaip3,Tnip1,Zc3h12a

GO:0045429	positive regulation of nitric oxide biosynthetic process	6	1.25E-14	Icam1,Il1b,Klf2,Ptgs2,Tlr2,Tnf
GO:0045732	positive regulation of protein catabolic process	6	1.25E-14	Herpud1,Ier3,Il1b,Rnf19b,Tnf,Tnfaip3
GO:0048878	chemical homeostasis	6	1.25E-14	Atp7a,Ehd1,Herpud1,Icam1,Il1b,Slc31a2
GO:0050708	regulation of protein secretion	6	1.25E-14	Hcar2,Il1b,Ptger4,Tlr2,Tnf,Zc3h12a
GO:0070647	protein modification by small protein conjugation or removal	6	1.25E-14	Birc3,Nfe2l2,Rnf19b,Socs3,Tnfaip3,Zc3h12a
GO:0072359	circulatory system development	6	1.25E-14	Atp7a,Pdgfb,Ptger4,Ptgs2,Socs3,Zc3h12a
GO:0097190	apoptotic signaling pathway	6	1.25E-14	Atp7a,Bcl3,Fas,Ier3,Il1b,Tnf
GO:1901362	organic cyclic compound biosynthetic process	6	1.25E-14	Atp7a,Bcl3,Ell2,Klf2,Nfe2l2,Nfkbiz
GO:1903039	positive regulation of leukocyte cell-cell adhesion	6	1.25E-14	Cd83,Icam1,Icosl,Il1b,Nfkbiz,Tnf
GO:1904018	positive regulation of vasculature development	6	1.25E-14	Cflar,Il1b,Nfe2l2,Pdgfb,Ptgs2,Zc3h12a

GO:0002697	regulation of immune effector process	6	3.47E-14	Il1b,Nfkbiz,Tlr2,Tnf,Tnfaip3,Zc3h12a
GO:0009725	response to hormone	6	3.47E-14	Cflar,Fas,Nfe2l2,Ptger4,Ptgs2,Tnf
GO:0010467	gene expression	6	3.47E-14	Bcl3,Ell2,Fas,Klf2,Nfe2l2,Nfkbiz
GO:0045088	regulation of innate immune response	6	3.47E-14	Birc3,Nfkbia,Tlr2,Tnf,Tnfaip3,Tnip1
GO:0050769	positive regulation of neurogenesis	6	3.47E-14	Cflar,Ehd1,Il1b,Nfe2l2,Skil,Tlr2
GO:0051092	positive regulation of NF-kappaB transcription factor activity	6	3.47E-14	Cflar,Icam1,Il1b,Tlr2,Tnf,Traf1
GO:0071496	cellular response to external stimulus	6	3.47E-14	Fas,Il1b,Nfe2l2,Ptger4,Ptgs2,Zc3h12a
GO:0071900	regulation of protein serine/threonine kinase activity	6	3.47E-14	Dusp6,Gadd45b,Il1b,Pdgfb,Tnf,Tnfaip3
GO:1902105	regulation of leukocyte differentiation	6	3.47E-14	Cd83,Fas,Il1b,Nfkbiz,Tnf,Zc3h12a
GO:1903037	regulation of leukocyte cell-cell adhesion	7	3.47E-14	Cd83,Icam1,Icosl,Il1b,Nfkbiz,Tnf,Zc3h12a
GO:0045321	leukocyte activation	8	4.21E-14	Atp7a,Bcl3,Fas,Icam1,Icosl,Ptger4,Tlr2,Tnfaip3

GO:0002683	negative regulation of immune system process	7	5.14E-14	Fas,Nfe2l2,Nfkbia,Ptger4,Tnf,Tnfaip3,Zc3h12a
GO:0071356	cellular response to tumor necrosis factor	7	5.14E-14	Birc3,Fas,Klf2,Nfe2l2,Nfkbia,Tnf,Zc3h12a
GO:0006886	intracellular protein transport	6	8.15E-14	Bcl3,Ehd1,Herpud1,Nfkbia,Rilpl2,Tnf
GO:0009895	negative regulation of catabolic process	6	8.15E-14	Fam46c,Hcar2,Ier3,Il1b,Tnf,Tnfaip3
GO:0019438	aromatic compound biosynthetic process	6	8.15E-14	Atp7a,Bcl3,Ell2,Klf2,Nfe2l2,Nfkbiz
GO:0070848	response to growth factor	6	8.15E-14	Atp7a,Cflar,Dusp6,Ehd1,Pdgfb,Skil
GO:0050729	positive regulation of inflammatory response	7	1.09E-13	Il1b,Nfkbia,Ptger4,Ptgs2,Tlr2,Tnf,Tnip1
GO:0043254	regulation of protein complex assembly	6	1.74E-13	Cdc42ep2,Fas,Icam1,Ptger4,Tlr2,Tnf
GO:0045892	negative regulation of transcription, DNA-templated	6	1.74E-13	Bcl3,Il1b,Klf2,Pdgfb,Skil,Tnf
GO:0071229	cellular response to acid chemical	6	1.74E-13	Atp7a,Klf2,Pdgfb,Ptger4,Tnf,Zc3h12a
GO:1901652	response to peptide	6	1.74E-13	Cflar,Klf2,Nfe2l2,Nfkbia,Ptgs2,Tnfaip3
GO:0019725	cellular homeostasis	6	3.43E-13	Atp7a,Herpud1,Icam1,Il1b,Nfe2l2,Slc31a2

GO:0090066	regulation of anatomical structure size	6	3.43E-13	Atp7a,Cdc42ep2,Icam1,Pdgfb,Ptgs2,Tlr2
GO:0043933	protein-containing complex subunit organization	6	6.30E-13	Atp7a,Birc3,Ehd1,Fas,Skil,Zc3h12a
GO:0034097	response to cytokine	17	7.42E-13	Birc3,Ccrl2,Cdc42ep2,Fas,Icam1,Il10ra,Il1b,Klf2,Nfe2l2,Nfkbia,Pdgfb,Ptgs2,Skil,Socs3,Tlr2,Tnf,Zc3h12a
GO:0000003	reproduction	6	1.81E-12	Icam1,Il1b,Pdgfb,Ptgs2,Skil,Socs3
GO:0010638	positive regulation of organelle organization	6	1.81E-12	Cdc42ep2,Fas,Icam1,Il1b,Pdgfb,Tnf
GO:0043122	regulation of I-kappaB kinase/NF-kappaB signaling	6	2.88E-12	Cflar,Il1b,Tnf,Tnfaip3,Tnip1,Zc3h12a
GO:0071222	cellular response to lipopolysaccharide	6	2.88E-12	Il1b,Nfkbia,Tnf,Tnfaip3,Tnip1,Zc3h12a
GO:0000122	negative regulation of transcription by RNA polymerase II	5	4.30E-12	Il1b,Klf2,Pdgfb,Skil,Tnf
GO:0006351	transcription, DNA-templated	5	4.30E-12	Bcl3,Ell2,Klf2,Nfe2l2,Nfkbiz
GO:0006508	proteolysis	5	4.30E-12	Cflar,Herpud1,Nfe2l2,Tnfaip3,Zc3h12a
GO:0010959	regulation of metal ion transport	5	4.30E-12	Atp7a,Icam1,Pdgfb,Ptgs2,Slc31a2
GO:0043271	negative regulation of ion transport	5	4.30E-12	Atp7a,Icam1,Il1b,Ptgs2,Tnf

GO:0044281	small molecule metabolic process	5	4.30E-12	Atp7a,Il1b,Ptgs2,Tlr2,Tnf
GO:0045765	regulation of angiogenesis	5	4.30E-12	Il1b,Klf2,Nfe2l2,Ptgs2,Zc3h12a
GO:0048666	neuron development	5	4.30E-12	Atp7a,Ehd1,Fas,Pdgfb,Skil
GO:0048872	homeostasis of number of cells	5	4.30E-12	Fas,Hcar2,Klf2,Skil,Tnfaip3
GO:0050728	negative regulation of inflammatory response	5	4.30E-12	Ier3,Ptger4,Socs3,Tnfaip3,Zc3h12a
GO:0051259	protein complex oligomerization	5	4.30E-12	Birc3,Ehd1,Fas,Skil,Zc3h12a
GO:0051345	positive regulation of hydrolase activity	5	4.30E-12	Cdc42ep2,Cflar,Fas,Icam1,Tnf
GO:0051346	negative regulation of hydrolase activity	5	4.30E-12	Birc3,Cflar,Herpud1,Ptgs2,Tnf
GO:1903320	regulation of protein modification by small protein conjugation or removal	5	4.30E-12	Birc3,Herpud1,Tnfaip3,Tnip1,Zc3h12a
GO:0008285	negative regulation of cell proliferation	5	1.07E-11	Il1b,Ptgs2,Tlr2,Tnf,Tnfaip3
GO:0009612	response to mechanical stimulus	5	1.07E-11	Il1b,Nfkbia,Ptger4,Ptgs2,Tnf
GO:0016567	protein ubiquitination	5	1.07E-11	Birc3,Nfe2l2,Rnf19b,Socs3,Tnfaip3

GO:0019216	regulation of lipid metabolic process	5	1.07E-11	Hcar2,Il1b,Pdgfb,Ptgs2,Tnf
GO:0032989	cellular component morphogenesis	5	1.07E-11	Atp7a,Cflar,Klf2,Rilpl2,Tlr2
GO:0043405	regulation of MAP kinase activity	5	1.07E-11	Dusp6,Gadd45b,Il1b,Pdgfb,Tnf
GO:0043900	regulation of multi-organism process	5	1.07E-11	Il1b,Tlr2,Tnf,Tnfaip3,Zc3h12a
GO:0044089	positive regulation of cellular component biogenesis	5	1.07E-11	Atp7a,Cdc42ep2,Fas,Icam1,Tnf
GO:0045619	regulation of lymphocyte differentiation	5	1.07E-11	Cd83,Fas,Il1b,Nfkbiz,Zc3h12a
GO:0045664	regulation of neuron differentiation	5	1.07E-11	Cflar,Ehd1,Il1b,Nfe2l2,Skil
GO:0045786	negative regulation of cell cycle	5	1.07E-11	Ier3,Ptgs2,Skil,Tnf,Tnfaip3
GO:0050863	regulation of T cell activation	5	1.07E-11	Cd83,Icosl,Il1b,Nfkbiz,Zc3h12a
GO:0055082	cellular chemical homeostasis	5	1.07E-11	Atp7a,Herpud1,Icam1,Il1b,Slc31a2
GO:0043281	regulation of cysteine-type endopeptidase activity involved in apoptotic process	6	1.71E-11	Birc3,Cflar,Fas,Herpud1,Ptgs2,Tnf

GO:0072358	cardiovascular system development	6	1.71E-11	Atp7a,Pdgfb,Ptger4,Ptgs2,Socs3,Zc3h12a
GO:0006469	negative regulation of protein kinase activity	5	2.27E-11	Dusp6,Gadd45b,Il1b,Socs3,Tnfaip3
GO:0032872	regulation of stress-activated MAPK cascade	5	2.27E-11	Fas,Gadd45b,Il1b,Tnf,Zc3h12a
GO:0040011	locomotion	5	2.27E-11	Ccrl2,Icam1,Il1b,Pdgfb,Tnf
GO:0050707	regulation of cytokine secretion	5	2.27E-11	Il1b,Ptger4,Tlr2,Tnf,Zc3h12a
GO:0050877	nervous system process	5	2.27E-11	Icam1,Il1b,Ptgs2,Tlr2,Tnf
GO:2001237	negative regulation of extrinsic apoptotic signaling pathway	5	2.27E-11	Cflar,Icam1,Il1b,Tnf,Tnfaip3
GO:0050678	regulation of epithelial cell proliferation	5	4.44E-11	Atp7a,Cflar,Pdgfb,Tnf,Tnfaip3
GO:0071236	cellular response to antibiotic	5	4.44E-11	Klf2,Nfe2l2,Pdgfb,Tnfaip3,Zc3h12a
GO:1901222	regulation of NIK/NF-kappaB signaling	6	8.92E-11	Bcl3,Birc3,Il1b,Tlr2,Tnf,Zc3h12a

GO:0002822	regulation of adaptive immune response based on somatic recombination of immune ...	5	1.38E-10	Il1b,Nfkbiz,Tnf,Tnfaip3,Zc3h12a
GO:0070374	positive regulation of ERK1 and ERK2 cascade	5	1.38E-10	Cflar,Icam1,Il1b,Pdgfb,Tlr2
GO:0002757	immune response-activating signal transduction	6	1.42E-10	Nfkbia,Nfkbiz,Tlr2,Tnf,Tnip1,Zc3h12a
GO:0002237	response to molecule of bacterial origin	11	2.13E-10	Fas,Il10ra,Il1b,Nfkbia,Ptger4,Ptgs2,Tlr2,Tnf,Tnfaip3,Tnip1,Zc3h12a
GO:0040008	regulation of growth	5	3.43E-10	Dusp6,Ptger4,Socs3,Tlr2,Tnf
GO:0023051	regulation of signaling	25	4.79E-10	Bcl3,Birc3,Cdc42ep2,Cflar,Dusp6,Fas,Gadd45b,Hcar2,Herpud1,Icam1,Ier3,Il1b,Nfe2l2,Nfkbia,Pdgfb,Ptger4,Ptgs2,Skil,Socs3,Tlr2,Tnf,Tnfaip3,Tnip1,Traf1,Zc3h12a
GO:0050714	positive regulation of protein secretion	5	7.41E-10	Hcar2,Il1b,Ptger4,Tlr2,Tnf
GO:0032270	positive regulation of cellular protein metabolic process	18	8.91E-10	Bcl3,Birc3,Cflar,Fas,Gadd45b,Herpud1,Icam1,Il1b,Nfkbia,Pdgfb,Ptger4,Ptgs2,Rnf19b,Tlr2,Tnf,Tnfaip3,Tnip1,Zc3h12a
GO:0006468	protein phosphorylation	5	1.04E-09	Il1b,Pdgfb,Ptger4,Tlr2,Tnf

GO:0051129	negative regulation of cellular component organization	5	1.04E-09	Cflar,Ier3,Ptger4,Tlr2,Tnf
GO:0001776	leukocyte homeostasis	4	1.41E-09	Fas,Hcar2,Skil,Tnfaip3
GO:0002286	T cell activation involved in immune response	4	1.41E-09	Atp7a,Bcl3,Icam1,Ptger4
GO:0002460	adaptive immune response based on somatic recombination of immune receptors ...	4	1.41E-09	Bcl3,Fas,Icam1,Icosl
GO:0002699	positive regulation of immune effector process	4	1.41E-09	Il1b,Nfkbiz,Tlr2,Tnf
GO:0002758	innate immune response-activating signal transduction	4	1.41E-09	Nfkbia,Tlr2,Tnf,Tnip1
GO:0006974	cellular response to DNA damage stimulus	4	1.41E-09	Bcl3,Ier3,Tnf,Zc3h12a
GO:0007346	regulation of mitotic cell cycle	4	1.41E-09	Ier3,Il1b,Pdgfb,Tnf
GO:0008015	blood circulation	4	1.41E-09	Icam1,Ier3,Pdgfb,Ptgs2

GO:0010469	regulation of signaling receptor activity	4	1.41E-09	Cdc42ep2,Il1b,Pdgfb,Tnf
GO:0032720	negative regulation of tumor necrosis factor production	4	1.41E-09	Bcl3,Ptger4,Tnfaip3,Zc3h12a
GO:0044265	cellular macromolecule catabolic process	4	1.41E-09	Herpud1,Nfe2l2,Tnfaip3,Zc3h12a
GO:0045834	positive regulation of lipid metabolic process	4	1.41E-09	Il1b,Pdgfb,Ptgs2,Tnf
GO:0046883	regulation of hormone secretion	4	1.41E-09	Hcar2,Il1b,Ptger4,Tnf
GO:0046890	regulation of lipid biosynthetic process	4	1.41E-09	Il1b,Pdgfb,Ptgs2,Tnf
GO:0048608	reproductive structure development	4	1.41E-09	Icam1,Pdgfb,Ptgs2,Socs3
GO:0050709	negative regulation of protein secretion	4	1.41E-09	Il1b,Ptger4,Tnf,Zc3h12a
GO:0050870	positive regulation of T cell activation	4	1.41E-09	Cd83,Icosl,Il1b,Nfkbiz
GO:0051384	response to glucocorticoid	4	1.41E-09	Cflar,Fas,Ptgs2,Tnf
GO:0070542	response to fatty acid	4	1.41E-09	Ptger4,Ptgs2,Tlr2,Zc3h12a

GO:0071241	cellular response to inorganic substance	4	1.41E-09	Atp7a,Cflar,Fas,Ptgs2
GO:0071453	cellular response to oxygen levels	4	1.41E-09	Cflar,Fas,Nfe2l2,Ptgs2
GO:1902041	regulation of extrinsic apoptotic signaling pathway via death domain receptors	4	1.41E-09	Cflar,Icam1,Skil,Tnfaip3
GO:0032496	response to lipopolysaccharide	10	2.44E-09	Fas,Il10ra,Il1b,Nfkbia,Ptger4,Ptgs2,Tnf,Tnfaip3,Tnip1,Zc3h12a
GO:0031347	regulation of defense response	12	2.77E-09	Birc3,Ier3,Il1b,Nfkbia,Ptger4,Ptgs2,Socs3,Tlr2,Tnf,Tnfaip3,Tnip1,Zc3h12a
GO:0001701	in utero embryonic development	4	2.96E-09	Klf2,Pdgfb,Skil,Socs3
GO:0002687	positive regulation of leukocyte migration	4	2.96E-09	Icam1,Il1b,Ptger4,Tlr2
GO:0002700	regulation of production of molecular mediator of immune response	4	2.96E-09	Il1b,Tlr2,Tnf,Tnfaip3
GO:0007159	leukocyte cell-cell adhesion	4	2.96E-09	Icam1,Il1b,Tnf,Tnip1
GO:0010608	posttranscriptional regulation of gene expression	4	2.96E-09	Bcl3,Fam46c,Tnf,Zc3h12a

GO:0010976	positive regulation of neuron projection development	4	2.96E-09	Cflar,Ehd1,Nfe2l2,Skil
GO:0032101	regulation of response to external stimulus	13	2.96E-09	Ier3,Il1b,Nfe2l2,Nfkbia,Pdgfb,Ptger4,Ptgs2,Socs3,Tlr2,Tnf,Tnfaip3,Tnip1,Zc3h12a
GO:0032652	regulation of interleukin-1 production	4	2.96E-09	Ptger4,Tlr2,Tnfaip3,Zc3h12a
GO:0043406	positive regulation of MAP kinase activity	4	2.96E-09	Gadd45b,Il1b,Pdgfb,Tnf
GO:0043523	regulation of neuron apoptotic process	4	2.96E-09	Atp7a,Fas,Il1b,Tnf
GO:0043901	negative regulation of multi-organism process	4	2.96E-09	Tlr2,Tnf,Tnfaip3,Zc3h12a
GO:0045580	regulation of T cell differentiation	4	2.96E-09	Cd83,Il1b,Nfkbiz,Zc3h12a
GO:0045766	positive regulation of angiogenesis	4	2.96E-09	Il1b,Nfe2l2,Ptgs2,Zc3h12a
GO:0048660	regulation of smooth muscle cell proliferation	4	2.96E-09	Pdgfb,Ptgs2,Tnf,Tnfaip3
GO:0050679	positive regulation of epithelial cell proliferation	4	2.96E-09	Atp7a,Cflar,Pdgfb,Tnfaip3

GO:0050730	regulation of peptidyl-tyrosine phosphorylation	4	2.96E-09	Icam1,Pdgfb,Ptger4,Socs3
GO:0050866	negative regulation of cell activation	4	2.96E-09	Fas,Pdgfb,Tnfaip3,Zc3h12a
GO:0060627	regulation of vesicle-mediated transport	4	2.96E-09	Ehd1,Il1b,Tlr2,Tnf
GO:0061458	reproductive system development	4	2.96E-09	Icam1,Pdgfb,Ptgs2,Socs3
GO:1902107	positive regulation of leukocyte differentiation	4	2.96E-09	Cd83,Il1b,Nfkbiz,Tnf
GO:2000117	negative regulation of cysteine-type endopeptidase activity	4	2.96E-09	Birc3,Cflar,Herpud1,Ptgs2
GO:0031323	regulation of cellular metabolic process	30	4.47E-09	Atp7a,Bcl3,Birc3,Cflar,Dusp6,Ell2,Fam46c,Fas,Gadd45b,Herpud1,Icam1,Icosl,Ier3,Il1b,Klf2,Nfe2l2,Nfkbia,Nfkbiz,Pdgfb,Ptger4,Ptgs2,Rnf19b,Skil,Socs3,Tlr2,Tnf,Tnfaip3,Tnip1,Traf1,Zc3h12a
GO:0009790	embryo development	5	4.69E-09	Klf2,Pdgfb,Skil,Socs3,Tnf
GO:0002831	regulation of response to biotic stimulus	4	5.47E-09	Il1b,Tlr2,Tnfaip3,Zc3h12a
GO:0007610	behavior	4	5.47E-09	Atp7a,Il1b,Ptgs2,Tlr2

GO:0010595	positive regulation of endothelial cell migration	4	5.47E-09	Nfe2l2,Pdgfb,Ptgs2,Zc3h12a
GO:0043436	oxoacid metabolic process	4	5.47E-09	Atp7a,Il1b,Ptgs2,Tlr2
GO:0045995	regulation of embryonic development	4	5.47E-09	Dusp6,Nfe2l2,Pdgfb,Tnfaip3
GO:0050900	leukocyte migration	4	5.47E-09	Icam1,Il1b,Pdgfb,Tnf
GO:0003006	developmental process involved in reproduction	5	5.70E-09	Icam1,Il1b,Pdgfb,Ptgs2,Socs3
GO:0002449	lymphocyte mediated immunity	5	8.76E-09	Bcl3,Fas,Icam1,Icosl,Rnf19b
GO:0062013	positive regulation of small molecule metabolic process	5	8.76E-09	Il1b,Pdgfb,Ptger4,Ptgs2,Tnf
GO:0072594	establishment of protein localization to organelle	4	9.43E-09	Bcl3,Nfkbia,Tnf,Tnfaip3
GO:1903034	regulation of response to wounding	4	9.43E-09	Atp7a,Nfe2l2,Pdgfb,Ptger4
GO:2000378	negative regulation of reactive oxygen species metabolic process	4	9.43E-09	Birc3,Cflar,Ptger4,Zc3h12a

GO:0050867	positive regulation of cell activation	5	1.28E-08	Cd83,Icosl,Il1b,Nfkbiz,Pdgfb
GO:0006875	cellular metal ion homeostasis	4	1.55E-08	Atp7a,Herpud1,Il1b,Slc31a2
GO:0045833	negative regulation of lipid metabolic process	4	1.55E-08	Hcar2,Il1b,Pdgfb,Tnf
GO:0034612	response to tumor necrosis factor	8	1.74E-08	Birc3,Fas,Klf2,Nfe2l2,Nfkbia,Ptgs2,Tnf,Zc3h12a
GO:0071363	cellular response to growth factor stimulus	5	1.83E-08	Atp7a,Cflar,Ehd1,Pdgfb,Skil
GO:0048514	blood vessel morphogenesis	4	2.39E-08	Pdgfb,Ptger4,Ptgs2,Zc3h12a
GO:1904036	negative regulation of epithelial cell apoptotic process	4	2.39E-08	Cflar,Icam1,Nfe2l2,Tnfaip3
GO:1905954	positive regulation of lipid localization	4	2.39E-08	Ehd1,Il1b,Nfkbia,Zc3h12a
GO:0022603	regulation of anatomical structure morphogenesis	14	3.15E-08	Cdc42ep2,Cflar,Dusp6,Ehd1,Icam1,Il1b,Klf2,Nfe2l2,Ptger4,Ptgs2,Skil,Tnf,Tnfaip3,Zc3h12a
GO:0010564	regulation of cell cycle process	4	3.53E-08	Ier3,Il1b,Pdgfb,Tnf
GO:0110053	regulation of actin filament organization	4	3.53E-08	Cdc42ep2,Icam1,Ptger4,Tlr2

GO:2001242	regulation of intrinsic apoptotic signaling pathway	4	3.53E-08	Herpud1,Nfe2l2,Ptgs2,Skil
GO:0031325	positive regulation of cellular metabolic process	22	4.89E-08	Bcl3,Birc3,Cflar,Ell2,Fas,Gadd45b,Herpud1,Icam1,Icosl,Il1b,Klf2,Nfe2l2,Nfkbia,Pdgfb,Ptger4,Ptgs2,Rnf19b,Tlr2,Tnf,Tnfaip3,Tnip1,Zc3h12a
GO:0032268	regulation of cellular protein metabolic process	20	5.25E-08	Bcl3,Birc3,Cflar,Dusp6,Fas,Gadd45b,Herpud1,Icam1,Il1b,Nfkbia,Pdgfb,Ptger4,Ptgs2,Rnf19b,Socs3,Tlr2,Tnf,Tnfaip3,Tnip1,Zc3h12a
GO:0060341	regulation of cellular localization	6	5.68E-08	Ehd1,Ier3,Ptger4,Ptgs2,Tnf,Zc3h12a
GO:0031399	regulation of protein modification process	17	7.92E-08	Birc3,Cflar,Dusp6,Fas,Gadd45b,Herpud1,Icam1,Il1b,Pdgfb,Ptger4,Ptgs2,Socs3,Tlr2,Tnf,Tnfaip3,Tnip1,Zc3h12a
GO:0120036	plasma membrane bounded cell projection organization	4	1.27E-07	Atp7a,Ehd1,Fas,Rilpl2
GO:0001932	regulation of protein phosphorylation	15	1.64E-07	Cflar,Dusp6,Fas,Gadd45b,Icam1,Il1b,Pdgfb,Ptger4,Ptgs2,Socs3,Tlr2,Tnf,Tnfaip3,Tnip1,Zc3h12a
GO:0023052	signaling	24	1.65E-07	Atp7a,Bcl3,Ccrl2,Cd83,Cdc42ep2,Fas,Hcar2,Herpud1,Icosl,Ier3,Il10ra,Il1b,Nfe2l2,Nfkbia,Nfkbiz,Pdgfb,Ptger4,Skil,Socs3,Tlr2,Tnf,Tnip1,Traf1,Zc3h12a
GO:0031324	negative regulation of cellular metabolic process	19	1.88E-07	Bcl3,Birc3,Cflar,Dusp6,Fam46c,Gadd45b,Herpud1,Ier3,Il1b,Klf2,Pdgfb,Ptger4,Ptgs2,Skil,Socs3,Tnf,Tnfaip3,Tnip1,Zc3h12a
GO:0090287	regulation of cellular response to growth factor stimulus	4	2.10E-07	Cflar,Dusp6,Pdgfb,Skil

GO:0033993	response to lipid	12	2.85E-07	Cflar,Fas,Il10ra,Il1b,Nfkbia,Ptger4,Ptgs2,Tlr2,Tnf,Tnfaip3,Tnip1,Zc3h12a
GO:0002285	lymphocyte activation involved in immune response	6	3.74E-07	Atp7a,Bcl3,Icam1,Icosl,Ptger4,Tnfaip3
GO:0002440	production of molecular mediator of immune response	3	3.74E-07	Fas,Icosl,Tlr2
GO:0002695	negative regulation of leukocyte activation	3	3.74E-07	Fas,Tnfaip3,Zc3h12a
GO:0002698	negative regulation of immune effector process	3	3.74E-07	Tnf,Tnfaip3,Zc3h12a
GO:0006109	regulation of carbohydrate metabolic process	3	3.74E-07	Ier3,Pdgfb,Ptger4
GO:0006417	regulation of translation	3	3.74E-07	Bcl3,Tnf,Zc3h12a
GO:0006511	ubiquitin-dependent protein catabolic process	3	3.74E-07	Herpud1,Nfe2l2,Tnfaip3
GO:0006606	protein import into nucleus	3	3.74E-07	Bcl3,Nfkbia,Tnf
GO:0007249	I-kappaB kinase/NF-kappaB signaling	3	3.74E-07	Bcl3,Nfkbia,Tlr2

GO:0008217	regulation of blood pressure	3	3.74E-07	Ier3,Pdgfb,Ptgs2
GO:0010827	regulation of glucose transmembrane transport	3	3.74E-07	Il1b,Nfe2l2,Tnf
GO:0010883	regulation of lipid storage	3	3.74E-07	Ehd1,Nfkbia,Zc3h12a
GO:0010952	positive regulation of peptidase activity	3	3.74E-07	Cflar,Fas,Tnf
GO:0030198	extracellular matrix organization	3	3.74E-07	Atp7a,Bcl3,Tnf
GO:0031330	negative regulation of cellular catabolic process	3	3.74E-07	Fam46c,Ier3,Tnfaip3
GO:0032088	negative regulation of NF-kappaB transcription factor activity	3	3.74E-07	Nfkbia,Tnfaip3,Zc3h12a
GO:0032755	positive regulation of interleukin-6 production	3	3.74E-07	Il1b,Tlr2,Tnf
GO:0033209	tumor necrosis factor-mediated signaling pathway	3	3.74E-07	Fas,Nfkbia,Tnf
GO:0043409	negative regulation of MAPK cascade	3	3.74E-07	Dusp6,Il1b,Tnip1

GO:0043903	regulation of symbiosis, encompassing mutualism through parasitism	3	3.74E-07	Tlr2,Tnf,Zc3h12a
GO:0046425	regulation of JAK-STAT cascade	3	3.74E-07	Bcl3,Ptger4,Socs3
GO:0046822	regulation of nucleocytoplasmic transport	3	3.74E-07	Ier3,Ptgs2,Zc3h12a
GO:0048661	positive regulation of smooth muscle cell proliferation	3	3.74E-07	Pdgfb,Ptgs2,Tnf
GO:0050710	negative regulation of cytokine secretion	3	3.74E-07	Ptger4,Tnf,Zc3h12a
GO:0050764	regulation of phagocytosis	3	3.74E-07	Il1b,Tlr2,Tnf
GO:0050777	negative regulation of immune response	3	3.74E-07	Tnf,Tnfaip3,Zc3h12a
GO:0050806	positive regulation of synaptic transmission	3	3.74E-07	Ptger4,Ptgs2,Tnf
GO:0050878	regulation of body fluid levels	3	3.74E-07	Nfe2l2,Pdgfb,Ptger4
GO:0050994	regulation of lipid catabolic process	3	3.74E-07	Hcar2,Il1b,Tnf

GO:0051147	regulation of muscle cell differentiation	3	3.74E-07	Cflar,Ehd1,Pdgfb
GO:0051193	regulation of cofactor metabolic process	3	3.74E-07	Ier3,Nfe2l2,Ptger4
GO:0051235	maintenance of location	3	3.74E-07	Il1b,Nfkbia,Tnf
GO:0070925	organelle assembly	3	3.74E-07	Cflar,Ehd1,Rilpl2
GO:0090276	regulation of peptide hormone secretion	3	3.74E-07	Il1b,Ptger4,Tnf
GO:0097191	extrinsic apoptotic signaling pathway	3	3.74E-07	Fas,Il1b,Tnf
GO:1903829	positive regulation of cellular protein localization	3	3.74E-07	Ptgs2,Tnf,Zc3h12a
GO:1904752	regulation of vascular associated smooth muscle cell migration	3	3.74E-07	Atp7a,Nfe2l2,Pdgfb
GO:2000146	negative regulation of cell motility	3	3.74E-07	Nfe2l2,Pdgfb,Ptger4
GO:2001243	negative regulation of intrinsic apoptotic signaling pathway	3	3.74E-07	Herpud1,Nfe2l2,Ptgs2
GO:0032103	positive regulation of response to external stimulus	8	3.91E-07	Il1b,Nfkbia,Pdgfb,Ptger4,Ptgs2,Tlr2,Tnf,Tnip1

GO:0050715	positive regulation of cytokine secretion	4	5.29E-07	Il1b,Ptger4,Tlr2,Tnf
GO:0050804	modulation of chemical synaptic transmission	4	5.29E-07	Il1b,Ptger4,Ptgs2,Tnf
GO:0002718	regulation of cytokine production involved in immune response	3	6.56E-07	Il1b,Tlr2,Tnf
GO:0002824	positive regulation of adaptive immune response based on somatic recombination ...	3	6.56E-07	Il1b,Nfkbiz,Tnf
GO:0006811	ion transport	3	6.56E-07	Atp7a,Nfkbie,Slc31a2
GO:0007049	cell cycle	3	6.56E-07	Ccng2,Ier3,Skil
GO:0007600	sensory perception	3	6.56E-07	Icam1,Ptgs2,Tnf
GO:0009267	cellular response to starvation	3	6.56E-07	Fas,Nfe2l2,Zc3h12a
GO:0009611	response to wounding	3	6.56E-07	Cflar,Pdgfb,Tnfaip3
GO:0022604	regulation of cell morphogenesis	3	6.56E-07	Cdc42ep2,Icam1,Skil
GO:0042093	T-helper cell differentiation	3	6.56E-07	Atp7a,Bcl3,Ptger4

GO:0043370	regulation of CD4-positive, alpha-beta T cell differentiation	3	6.56E-07	Cd83,Nfkbiz,Zc3h12a
GO:0045637	regulation of myeloid cell differentiation	3	6.56E-07	Fas,Nfkbia,Tnf
GO:0045840	positive regulation of mitotic nuclear division	3	6.56E-07	Il1b,Pdgfb,Tnf
GO:0046330	positive regulation of JNK cascade	3	6.56E-07	Gadd45b,Il1b,Tnf
GO:0046889	positive regulation of lipid biosynthetic process	3	6.56E-07	Il1b,Ptgs2,Tnf
GO:0050704	regulation of interleukin-1 secretion	3	6.56E-07	Ptger4,Tlr2,Zc3h12a
GO:0050731	positive regulation of peptidyl-tyrosine phosphorylation	3	6.56E-07	Icam1,Pdgfb,Ptger4
GO:0051098	regulation of binding	3	6.56E-07	Bcl3,Herpud1,Pdgfb
GO:0051961	negative regulation of nervous system development	3	6.56E-07	Il1b,Tlr2,Tnf
GO:0071248	cellular response to metal ion	3	6.56E-07	Atp7a,Fas,Ptgs2
GO:0071498	cellular response to fluid shear stress	3	6.56E-07	Klf2,Nfe2l2,Ptgs2

GO:0097327	response to antineoplastic agent	3	6.56E-07	Cflar,Pdgfb,Zc3h12a
GO:0098754	detoxification	3	6.56E-07	Atp7a,Nfe2l2,Ptgs2
GO:1900745	positive regulation of p38MAPK cascade	3	6.56E-07	Gadd45b,Il1b,Zc3h12a
GO:1901653	cellular response to peptide	3	6.56E-07	Cflar,Klf2,Nfe2l2
GO:1903322	positive regulation of protein modification by small protein conjugation or ...	3	6.56E-07	Birc3,Tnip1,Zc3h12a
GO:1903364	positive regulation of cellular protein catabolic process	3	6.56E-07	Herpud1,Rnf19b,Tnfaip3
GO:1903707	negative regulation of hemopoiesis	3	6.56E-07	Nfe2l2,Nfkbia,Zc3h12a
GO:2000027	regulation of animal organ morphogenesis	3	6.56E-07	Cflar,Tnf,Tnfaip3
GO:0032677	regulation of interleukin-8 production	5	7.25E-07	Bcl3,Il1b,Ptger4,Tlr2,Tnf
GO:0042108	positive regulation of cytokine biosynthetic process	5	7.83E-07	Bcl3,Icosl,Il1b,Tlr2,Tnf

GO:0031401	positive regulation of protein modification process	13	9.68E-07	Birc3,Cflar,Fas,Gadd45b,Icam1,Il1b,Pdgfb,Ptger4,Ptgs2,Tlr2,Tnf,Tnip1,Zc3h12a
GO:0044403	symbiont process	4	9.68E-07	Icam1,Tlr2,Tnip1,Zc3h12a
GO:0000902	cell morphogenesis	3	1.07E-06	Atp7a,Klf2,Rilpl2
GO:0003014	renal system process	3	1.07E-06	Fas,Pdgfb,Ptger4
GO:0007186	G-protein coupled receptor signaling pathway	3	1.07E-06	Ccrl2,Hcar2,Ptger4
GO:0009743	response to carbohydrate	3	1.07E-06	Icam1,Il1b,Ptgs2
GO:0032675	regulation of interleukin-6 production	6	1.07E-06	Il1b,Klf2,Tlr2,Tnf,Tnfaip3,Zc3h12a
GO:0051924	regulation of calcium ion transport	3	1.07E-06	Icam1,Pdgfb,Ptgs2
GO:0061061	muscle structure development	3	1.07E-06	Cflar,Pdgfb,Skil
GO:1902042	negative regulation of extrinsic apoptotic signaling pathway via death domain ...	3	1.07E-06	Cflar,Icam1,Tnfaip3
GO:1904467	regulation of tumor necrosis factor secretion	3	1.07E-06	Ptger4,Tlr2,Zc3h12a

GO:2000352	negative regulation of endothelial cell apoptotic process	3	1.07E-06	Icam1,Nfe2l2,Tnfaip3
GO:0001890	placenta development	3	1.67E-06	Pdgfb,Ptgs2,Socs3
GO:0002009	morphogenesis of an epithelium	3	1.67E-06	Atp7a,Pdgfb,Socs3
GO:0002703	regulation of leukocyte mediated immunity	3	1.67E-06	Il1b,Tlr2,Tnf
GO:0010506	regulation of autophagy	3	1.67E-06	Tlr2,Tnfaip3,Zc3h12a
GO:0043536	positive regulation of blood vessel endothelial cell migration	3	1.67E-06	Nfe2l2,Pdgfb,Ptgs2
GO:0048732	gland development	3	1.67E-06	Nfkbia,Pdgfb,Tnf
GO:0051260	protein homooligomerization	3	1.67E-06	Ehd1,Fas,Skil
GO:0051353	positive regulation of oxidoreductase activity	3	1.67E-06	Atp7a,Il1b,Tnf
GO:0070266	necroptotic process	3	1.67E-06	Birc3,Fas,Tnf
GO:2001023	regulation of response to drug	3	1.67E-06	Nfe2l2,Ptger4,Tnf

GO:2001239	regulation of extrinsic apoptotic signaling pathway in absence of ligand	3	1.67E-06	Fas,Il1b,Tnf
GO:1903522	regulation of blood circulation	4	1.87E-06	Icam1,Pdgfb,Ptgs2,Zc3h12a
GO:0032269	negative regulation of cellular protein metabolic process	12	2.12E-06	Birc3,Cflar,Dusp6,Gadd45b,Herpud1,Il1b,Ptgs2,Socs3,Tnf,Tnfaip3,Tnip1,Zc3h12a
GO:0022407	regulation of cell-cell adhesion	8	2.28E-06	Cd83,Icam1,Icosl,Il1b,Nfkbiz,Tnf,Tnfaip3,Zc3h12a
GO:0030031	cell projection assembly	3	2.42E-06	Ehd1,Pdgfb,Rilpl2
GO:0044257	cellular protein catabolic process	3	2.42E-06	Herpud1,Nfe2l2,Tnfaip3
GO:0120032	regulation of plasma membrane bounded cell projection assembly	3	2.42E-06	Atp7a,Cdc42ep2,Icam1
GO:0030154	cell differentiation	21	2.59E-06	Atp7a,Bcl3,Cflar,Dusp6,Ehd1,Fas,Gadd45b,Icam1,Il1b,Klf2,Nfe2l2,Pdgfb,Ptger4,Ptgs2,Rilpl2,Skil,Socs3,Tlr2,Tnf,Tnfaip3,Zc3h12a
GO:0006959	humoral immune response	3	3.42E-06	Bcl3,Cd83,Tnf
GO:0008630	intrinsic apoptotic signaling pathway in response to DNA damage	3	3.42E-06	Bcl3,Ier3,Tnf

GO:0043123	positive regulation of I-kappaB kinase/NF-kappaB signaling	3	3.42E-06	Cflar,Il1b,Tnf
GO:0043525	positive regulation of neuron apoptotic process	3	3.42E-06	Fas,Il1b,Tnf
GO:0045907	positive regulation of vasoconstriction	3	3.42E-06	Icam1,Pdgfb,Ptgs2
GO:0031326	regulation of cellular biosynthetic process	21	3.89E-06	Bcl3,Cflar,Ell2,Icam1,Icosl,Ier3,Il1b,Klf2,Nfe2l2,Nfkbia,Nfkbiz,Pdgfb,Ptger4,Ptgs2,Skil,Tlr2,Tnf,Tnfaip3,Tnip1,Traf1,Zc3h12a
GO:0032733	positive regulation of interleukin-10 production	4	4.13E-06	Bcl3,Cd83,Ptger4,Tlr2
GO:0002252	immune effector process	8	4.40E-06	Atp7a,Bcl3,Fas,Icam1,Icosl,Ptger4,Rnf19b,Tnfaip3
GO:0002429	immune response-activating cell surface receptor signaling pathway	3	4.63E-06	Nfkbiz,Tlr2,Zc3h12a
GO:0010656	negative regulation of muscle cell apoptotic process	3	4.63E-06	Cflar,Nfe2l2,Zc3h12a
GO:0031328	positive regulation of cellular biosynthetic process	15	4.63E-06	Bcl3,Ell2,Icam1,Icosl,Il1b,Klf2,Nfe2l2,Nfkbia,Pdgfb,Ptger4,Ptgs2,Tlr2,Tnf,Tnip1,Zc3h12a

GO:0045087	innate immune response	3	4.63E-06	Cdc42ep2,Rnf19b,Tlr2
GO:0045582	positive regulation of T cell differentiation	3	4.63E-06	Cd83,Il1b,Nfkbiz
GO:0072593	reactive oxygen species metabolic process	3	4.63E-06	Atp7a,Pdgfb,Tlr2
GO:0030162	regulation of proteolysis	10	4.73E-06	Birc3,Cflar,Fas,Herpud1,Il1b,Ptgs2,Rnf19b,Tnf,Tnip1,Zc3h12a
GO:0001775	cell activation	9	5.01E-06	Atp7a,Bcl3,Fas,Icam1,Icosl,Ptger4,Tlr2,Tnf,Tnfaip3
GO:0031622	positive regulation of fever generation	3	6.06E-06	Il1b,Ptgs2,Tnf
GO:0042742	defense response to bacterium	3	6.06E-06	Bcl3,Tlr2,Tnf
GO:0061041	regulation of wound healing	3	6.06E-06	Nfe2l2,Pdgfb,Ptger4
GO:1901343	negative regulation of vasculature development	3	6.06E-06	Klf2,Pdgfb,Ptger4
GO:0033554	cellular response to stress	13	6.70E-06	Atp7a,Bcl3,Cflar,Fas,Herpud1,Ier3,Klf2,Nfe2l2,Ptger4,Ptgs2,Tnf,Tnfaip3,Zc3h12a
GO:0045932	negative regulation of muscle contraction	3	7.78E-06	Ptger4,Ptgs2,Zc3h12a
GO:0071260	cellular response to mechanical stimulus	3	7.78E-06	Il1b,Ptger4,Ptgs2

GO:0032757	positive regulation of interleukin-8 production	4	8.54E-06	Il1b,Ptger4,Tlr2,Tnf
GO:0019219	regulation of nucleobase-containing compound metabolic process	20	9.13E-06	Atp7a,Bcl3,Cflar,Ell2,Fam46c,Icam1,Ier3,Il1b,Klf2,Nfe2l2,Nfkbia,Nfkbiz,Pdgfb,Skil,Tlr2,Tnf,Tnfaip3,Tnip1,Traf1,Zc3h12a
GO:0032715	negative regulation of interleukin-6 production	4	9.28E-06	Klf2,Tnf,Tnfaip3,Zc3h12a
GO:0000060	protein import into nucleus, translocation	3	9.80E-06	Bcl3,Nfkbia,Tnf
GO:0006935	chemotaxis	3	9.80E-06	Ccrl2,Il1b,Pdgfb
GO:0030155	regulation of cell adhesion	9	1.31E-05	Cd83,Icam1,Icosl,Il1b,Nfkbiz,Ptger4,Tnf,Tnfaip3,Zc3h12a
GO:1901224	positive regulation of NIK/NF-kappaB signaling	3	1.49E-05	Il1b,Tlr2,Tnf
GO:0031329	regulation of cellular catabolic process	9	1.61E-05	Fam46c,Herpud1,Ier3,Il1b,Rnf19b,Tlr2,Tnf,Tnfaip3,Zc3h12a
GO:0031331	positive regulation of cellular catabolic process	7	1.65E-05	Herpud1,Il1b,Rnf19b,Tlr2,Tnf,Tnfaip3,Zc3h12a
GO:0034599	cellular response to oxidative stress	6	1.71E-05	Atp7a,Cflar,Klf2,Nfe2l2,Tnfaip3,Zc3h12a

GO:0044003	modification by symbiont of host morphology or physiology	3	1.79E-05	Tlr2,Tnip1,Zc3h12a
GO:0051781	positive regulation of cell division	3	1.79E-05	Il1b,Pdgfb,Tnf
GO:0034614	cellular response to reactive oxygen species	5	2.30E-05	Atp7a,Cflar,Klf2,Nfe2l2,Tnfaip3
GO:0051674	localization of cell	4	2.37E-05	Icam1,Il1b,Pdgfb,Tnf
GO:0043434	response to peptide hormone	3	2.52E-05	Cflar,Nfe2l2,Ptgs2
GO:0071456	cellular response to hypoxia	3	2.95E-05	Cflar,Nfe2l2,Ptgs2
GO:0071901	negative regulation of protein serine/threonine kinase activity	3	2.95E-05	Dusp6,Il1b,Tnfaip3
GO:1901655	cellular response to ketone	3	2.95E-05	Cflar,Klf2,Ptger4
GO:0032680	regulation of tumor necrosis factor production	5	3.14E-05	Bcl3,Ptger4,Tlr2,Tnfaip3,Zc3h12a
GO:0023056	positive regulation of signaling	13	3.83E-05	Cflar,Fas,Gadd45b,Hcar2,Icam1,Il1b,Pdgfb,Ptger4,Ptgs2,Skil,Tlr2,Tnf,Zc3h12a
GO:0070301	cellular response to hydrogen peroxide	3	3.94E-05	Klf2,Nfe2l2,Tnfaip3

GO:0042110	T cell activation	6	5.13E-05	Atp7a,Bcl3,Fas,Icam1,Icosl,Ptger4
GO:0019724	B cell mediated immunity	3	5.14E-05	Bcl3,Fas,Icosl
GO:0034114	regulation of heterotypic cell-cell adhesion	3	5.14E-05	Il1b,Tnf,Tnfaip3
GO:1901137	carbohydrate derivative biosynthetic process	3	5.81E-05	Atp7a,Il1b,Tnip1
GO:0032692	negative regulation of interleukin-1 production	3	6.55E-05	Ptger4,Tnfaip3,Zc3h12a
GO:0048568	embryonic organ development	3	6.55E-05	Pdgfb,Socs3,Tnf
GO:0032102	negative regulation of response to external stimulus	6	7.33E-05	Ier3,Pdgfb,Ptger4,Socs3,Tnfaip3,Zc3h12a
GO:1903036	positive regulation of response to wounding	3	7.33E-05	Atp7a,Nfe2l2,Ptger4
GO:0031400	negative regulation of protein modification process	8	7.68E-05	Dusp6,Gadd45b,Il1b,Socs3,Tnf,Tnfaip3,Tnip1,Zc3h12a
GO:0000079	regulation of cyclin-dependent protein serine/threonine kinase activity	2	8.12E-05	Pdgfb,Tnfaip3

GO:0001892	embryonic placenta development	2	8.12E-05	Pdgfb,Socs3
GO:0002526	acute inflammatory response	2	8.12E-05	Icam1,Il1b
GO:0002637	regulation of immunoglobulin production	2	8.12E-05	Tnf,Tnfaip3
GO:0002676	regulation of chronic inflammatory response	2	8.12E-05	Tnf,Tnfaip3
GO:0002708	positive regulation of lymphocyte mediated immunity	2	8.12E-05	Il1b,Tnf
GO:0003158	endothelium development	2	8.12E-05	Icam1,Pdgfb
GO:0006690	icosanoid metabolic process	2	8.12E-05	Ptgs2,Tlr2
GO:0006812	cation transport	2	8.12E-05	Atp7a,Nfkbie
GO:0006874	cellular calcium ion homeostasis	2	8.12E-05	Herpud1,Il1b
GO:0006878	cellular copper ion homeostasis	2	8.12E-05	Atp7a,Slc31a2
GO:0007167	enzyme linked receptor protein signaling pathway	2	8.12E-05	Pdgfb,Skil
GO:0007254	JNK cascade	2	8.12E-05	Ptger4,Tnf

GO:0007435	salivary gland morphogenesis	2	8.12E-05	Pdgfb,Tnf
GO:0007568	aging	2	8.12E-05	Nfe2l2,Ptgs2
GO:0007612	learning	2	8.12E-05	Ptgs2,Tlr2
GO:0008360	regulation of cell shape	2	8.12E-05	Cdc42ep2,Icam1
GO:0008544	epidermis development	2	8.12E-05	Atp7a,Ptgs2
GO:0009411	response to UV	2	8.12E-05	Bcl3,Ptgs2
GO:0010470	regulation of gastrulation	2	8.12E-05	Dusp6,Tnfaip3
GO:0010821	regulation of mitochondrion organization	2	8.12E-05	Fas,Ier3
GO:0010829	negative regulation of glucose transmembrane transport	2	8.12E-05	Il1b,Tnf
GO:0015893	drug transport	2	8.12E-05	Nfkbie,Tlr2
GO:0016358	dendrite development	2	8.12E-05	Atp7a,Fas
GO:0016579	protein deubiquitination	2	8.12E-05	Tnfaip3,Zc3h12a
GO:0019233	sensory perception of pain	2	8.12E-05	Ptgs2,Tnf
GO:0032495	response to muramyl dipeptide	2	8.12E-05	Nfkbia,Tnfaip3

GO:0032729	positive regulation of interferon-gamma production	3	8.12E-05	Bcl3,Il1b,Tnf
GO:0032940	secretion by cell	2	8.12E-05	Rnf19b,Tlr2
GO:0033198	response to ATP	2	8.12E-05	Il1b,Ptgs2
GO:0042063	gliogenesis	2	8.12E-05	Pdgfb,Tlr2
GO:0042102	positive regulation of T cell proliferation	2	8.12E-05	Icosl,Il1b
GO:0042633	hair cycle	2	8.12E-05	Atp7a,Ptgs2
GO:0042692	muscle cell differentiation	2	8.12E-05	Cflar,Pdgfb
GO:0043010	camera-type eye development	2	8.12E-05	Pdgfb,Skil
GO:0043280	positive regulation of cysteine-type endopeptidase activity involved in ...	2	8.12E-05	Fas,Tnf
GO:0043372	positive regulation of CD4-positive, alpha-beta T cell differentiation	2	8.12E-05	Cd83,Nfkbiz
GO:0043488	regulation of mRNA stability	2	8.12E-05	Fam46c,Zc3h12a
GO:0043491	protein kinase B signaling	2	8.12E-05	Il1b,Tnf

GO:0044068	modulation by symbiont of host cellular process	2	8.12E-05	Tnip1,Zc3h12a
GO:0045071	negative regulation of viral genome replication	2	8.12E-05	Tnf,Zc3h12a
GO:0045622	regulation of T-helper cell differentiation	2	8.12E-05	Nfkbiz,Zc3h12a
GO:0046627	negative regulation of insulin receptor signaling pathway	2	8.12E-05	Il1b,Socs3
GO:0048469	cell maturation	2	8.12E-05	Klf2,Pdgfb
GO:0050688	regulation of defense response to virus	2	8.12E-05	Il1b,Zc3h12a
GO:0050706	regulation of interleukin-1 beta secretion	2	8.12E-05	Tlr2,Zc3h12a
GO:0050803	regulation of synapse structure or activity	2	8.12E-05	Il10ra,Tlr2
GO:0050807	regulation of synapse organization	2	8.12E-05	Il10ra,Tlr2
GO:0050830	defense response to Gram-positive bacterium	2	8.12E-05	Tlr2,Tnf

GO:0050852	T cell receptor signaling pathway	2	8.12E-05	Nfkbiz,Zc3h12a
GO:0051044	positive regulation of membrane protein ectodomain proteolysis	2	8.12E-05	Il1b,Tnf
GO:0051195	negative regulation of cofactor metabolic process	2	8.12E-05	Ier3,Ptger4
GO:0051291	protein heterooligomerizatio n	2	8.12E-05	Birc3,Skil
GO:0051955	regulation of amino acid transport	2	8.12E-05	Il1b,Tnf
GO:0060271	cilium assembly	2	8.12E-05	Ehd1,Rilpl2
GO:0061024	membrane organization	2	8.12E-05	Ier3,Rilpl2
GO:0061138	morphogenesis of a branching epithelium	2	8.12E-05	Pdgfb,Socs3
GO:0070163	regulation of adiponectin secretion	2	8.12E-05	Hcar2,Il1b
GO:0071347	cellular response to interleukin-1	2	8.12E-05	Klf2,Zc3h12a
GO:0071466	cellular response to xenobiotic stimulus	2	8.12E-05	Cflar,Pdgfb

GO:0071499	cellular response to laminar fluid shear stress	2	8.12E-05	Klf2,Nfe2l2
GO:0072659	protein localization to plasma membrane	2	8.12E-05	Rilpl2,Tnf
GO:0090303	positive regulation of wound healing	2	8.12E-05	Nfe2l2,Ptger4
GO:0097527	necroptotic signaling pathway	2	8.12E-05	Fas,Tnf
GO:0097529	myeloid leukocyte migration	2	8.12E-05	Il1b,Pdgfb
GO:0098869	cellular oxidant detoxification	2	8.12E-05	Atp7a,Ptgs2
GO:0120034	positive regulation of plasma membrane bounded cell projection assembly	2	8.12E-05	Atp7a,Cdc42ep2
GO:1900127	positive regulation of hyaluronan biosynthetic process	2	8.12E-05	Pdgfb,Ptger4
GO:1904019	epithelial cell apoptotic process	2	8.12E-05	Fas,Tnf
GO:1904707	positive regulation of vascular smooth muscle cell proliferation	2	8.12E-05	Pdgfb,Tnf

GO:1904754	positive regulation of vascular associated smooth muscle cell migration	2	8.12E-05	Atp7a,Pdgfb
GO:1904996	positive regulation of leukocyte adhesion to vascular endothelial cell	2	8.12E-05	Icam1,Tnf
GO:2000108	positive regulation of leukocyte apoptotic process	2	8.12E-05	Fas,Hcar2
GO:2001235	positive regulation of apoptotic signaling pathway	2	8.12E-05	Fas,Skil
GO:0014910	regulation of smooth muscle cell migration	4	8.81E-05	Atp7a,Nfe2l2,Pdgfb,Ptger4
GO:0001525	angiogenesis	2	0.00011	Ptgs2,Zc3h12a
GO:0001562	response to protozoan	2	0.00011	Bcl3,Ier3
GO:0001936	regulation of endothelial cell proliferation	2	0.00011	Pdgfb,Tnf
GO:0002377	immunoglobulin production	2	0.00011	Fas,Icosl
GO:0002437	inflammatory response to antigenic stimulus	2	0.00011	Icam1,Tnf

GO:0002693	positive regulation of cellular extravasation	2	0.00011	Icam1,Ptger4
GO:0002720	positive regulation of cytokine production involved in immune response	2	0.00011	Il1b,Tlr2
GO:0002755	MyD88-dependent toll-like receptor signaling pathway	2	0.00011	Tlr2,Tnip1
GO:0002790	peptide secretion	2	0.00011	Rnf19b,Tlr2
GO:0006366	transcription by RNA polymerase II	2	0.00011	Ell2,Nfe2l2
GO:0007005	mitochondrion organization	2	0.00011	Atp7a,Ier3
GO:0007519	skeletal muscle tissue development	2	0.00011	Cflar,Skil
GO:0009101	glycoprotein biosynthetic process	2	0.00011	Atp7a,Tnip1
GO:0010226	response to lithium ion	2	0.00011	Fas,Ptgs2
GO:0010575	positive regulation of vascular endothelial growth factor production	2	0.00011	Il1b,Ptgs2
GO:0030968	endoplasmic reticulum unfolded protein response	2	0.00011	Herpud1,Nfe2l2

GO:0040036	regulation of fibroblast growth factor receptor signaling pathway	2	0.00011	Dusp6,Pdgfb
GO:0042307	positive regulation of protein import into nucleus	2	0.00011	Ptgs2,Zc3h12a
GO:0043270	positive regulation of ion transport	2	0.00011	Il1b,Pdgfb
GO:0043393	regulation of protein binding	2	0.00011	Herpud1,Pdgfb
GO:0043407	negative regulation of MAP kinase activity	2	0.00011	Dusp6,Il1b
GO:0044130	negative regulation of growth of symbiont in host	2	0.00011	Tlr2,Tnf
GO:0045414	regulation of interleukin-8 biosynthetic process	2	0.00011	Bcl3,Tnf
GO:0045600	positive regulation of fat cell differentiation	2	0.00011	Ptgs2,Zc3h12a
GO:0045687	positive regulation of glial cell differentiation	2	0.00011	Il1b,Tlr2

GO:0045986	negative regulation of smooth muscle contraction	2	0.00011	Ptger4,Ptgs2
GO:0046700	heterocycle catabolic process	2	0.00011	Nfe2l2,Zc3h12a
GO:0050766	positive regulation of phagocytosis	2	0.00011	Il1b,Tnf
GO:0050796	regulation of insulin secretion	2	0.00011	Il1b,Tnf
GO:0050921	positive regulation of chemotaxis	2	0.00011	Il1b,Pdgfb
GO:0051052	regulation of DNA metabolic process	2	0.00011	Ier3,Pdgfb
GO:0051148	negative regulation of muscle cell differentiation	2	0.00011	Cflar,Pdgfb
GO:0051588	regulation of neurotransmitter transport	2	0.00011	Ptger4,Tnf
GO:0051606	detection of stimulus	2	0.00011	Tlr2,Tnf
GO:0060688	regulation of morphogenesis of a branching structure	2	0.00011	Il1b,Tnf
GO:0071887	leukocyte apoptotic process	2	0.00011	Fas,Hcar2

GO:0090305	nucleic acid phosphodiester bond hydrolysis	2	0.00011	Zc3h12a,Zc3h12c
GO:1901361	organic cyclic compound catabolic process	2	0.00011	Nfe2l2,Zc3h12a
GO:1902624	positive regulation of neutrophil migration	2	0.00011	Il1b,Ptger4
GO:1903845	negative regulation of cellular response to transforming growth factor beta ...	2	0.00011	Cflar,Skil
GO:1990776	response to angiotensin	2	0.00011	Nfe2l2,Ptgs2
GO:2000181	negative regulation of blood vessel morphogenesis	2	0.00011	Klf2,Ptger4
GO:2000316	regulation of T-helper 17 type immune response	2	0.00011	Nfkbiz,Zc3h12a
GO:2001024	negative regulation of response to drug	2	0.00011	Nfe2l2,Tnf
GO:0031663	lipopolysaccharide-mediated signaling pathway	3	0.00013	Il1b,Nfkbia,Tnf

GO:0032649	regulation of interferon-gamma production	4	0.00013	Bcl3,Il1b,Tnf,Zc3h12a
GO:0000187	activation of MAPK activity	2	0.00016	Il1b,Tnf
GO:0002573	myeloid leukocyte differentiation	2	0.00016	Tlr2,Tnf
GO:0007276	gamete generation	2	0.00016	Ptgs2,Skil
GO:0007431	salivary gland development	2	0.00016	Pdgfb,Tnf
GO:0009746	response to hexose	2	0.00016	Icam1,Ptgs2
GO:0010001	glial cell differentiation	2	0.00016	Pdgfb,Tlr2
GO:0010498	proteasomal protein catabolic process	2	0.00016	Herpud1,Nfe2l2
GO:0010508	positive regulation of autophagy	2	0.00016	Tlr2,Zc3h12a
GO:0010639	negative regulation of organelle organization	2	0.00016	Ier3,Tlr2
GO:0043547	positive regulation of GTPase activity	2	0.00016	Cdc42ep2,Icam1
GO:0046324	regulation of glucose import	2	0.00016	Nfe2l2,Tnf
GO:0046394	carboxylic acid biosynthetic process	2	0.00016	Il1b,Ptgs2
GO:0048536	spleen development	2	0.00016	Bcl3,Fas

GO:0048638	regulation of developmental growth	2	0.00016	Dusp6,Ptger4
GO:0050805	negative regulation of synaptic transmission	2	0.00016	Il1b,Ptgs2
GO:0051769	regulation of nitric-oxide synthase biosynthetic process	2	0.00016	Ptger4,Tlr2
GO:0051926	negative regulation of calcium ion transport	2	0.00016	Icam1,Ptgs2
GO:0051953	negative regulation of amine transport	2	0.00016	Il1b,Tnf
GO:0061515	myeloid cell development	2	0.00016	Klf2,Tlr2
GO:0070373	negative regulation of ERK1 and ERK2 cascade	2	0.00016	Dusp6,Tnip1
GO:0097435	supramolecular fiber organization	2	0.00016	Atp7a,Cflar
GO:0098801	regulation of renal system process	2	0.00016	Pdgfb,Ptger4
GO:1903427	negative regulation of reactive oxygen species biosynthetic process	2	0.00016	Cflar,Zc3h12a

GO:1990830	cellular response to leukemia inhibitory factor	2	0.00016	Icam1,Socs3
GO:2000347	positive regulation of hepatocyte proliferation	2	0.00016	Cflar,Tnfaip3
GO:0010884	positive regulation of lipid storage	2	0.00021	Ehd1,Zc3h12a
GO:0016064	immunoglobulin mediated immune response	2	0.00021	Bcl3,Icosl
GO:0018193	peptidyl-amino acid modification	2	0.00021	Atp7a,Pdgfb
GO:0032741	positive regulation of interleukin-18 production	2	0.00021	Tlr2,Tnf
GO:0032891	negative regulation of organic acid transport	2	0.00021	Il1b,Tnf
GO:0044703	multi-organism reproductive process	2	0.00021	Ptgs2,Skil
GO:0045667	regulation of osteoblast differentiation	2	0.00021	Ptger4,Tnf

GO:0050711	negative regulation of interleukin-1 secretion	2	0.00021	Ptger4,Zc3h12a
GO:0060541	respiratory system development	2	0.00021	Atp7a,Klf2
GO:0060664	epithelial cell proliferation involved in salivary gland morphogenesis	2	0.00021	Pdgfb,Tnf
GO:0071230	cellular response to amino acid stimulus	2	0.00021	Atp7a,Tnf
GO:1901739	regulation of myoblast fusion	2	0.00021	Cflar,Ehd1
GO:1903578	regulation of ATP metabolic process	2	0.00021	Atp7a,Ier3
GO:0032501	multicellular organismal process	24	0.00022	Atp7a,Bcl3,Cflar,Dusp6,Ehd1,Fas,Gadd45b,Icam1,Icosl,Ier3,Il1b,Klf2,Nfe2l2,Nfkbia,Pdgfb,Ptger4,Ptgs2,Rnf19b,Skil,Socs3,Tlr2,Tnf,Tnfaip3,Zc3h12a
GO:0009615	response to virus	3	0.00027	Bcl3,Tnf,Zc3h12a
GO:0002312	B cell activation involved in immune response	3	0.00028	Bcl3,Icosl,Tnfaip3
GO:0003073	regulation of systemic arterial blood pressure	2	0.00028	Ier3,Pdgfb

GO:0010927	cellular component assembly involved in morphogenesis	2	0.00028	Cflar,Tlr2
GO:0030730	sequestering of triglyceride	2	0.00028	Il1b,Tnf
GO:0045123	cellular extravasation	2	0.00028	Icam1,Tnf
GO:0046426	negative regulation of JAK-STAT cascade	2	0.00028	Bcl3,Socs3
GO:0048589	developmental growth	2	0.00028	Cflar,Klf2
GO:0050869	negative regulation of B cell activation	2	0.00028	Fas,Tnfaip3
GO:0071375	cellular response to peptide hormone stimulus	2	0.00028	Cflar,Nfe2l2
GO:1903670	regulation of sprouting angiogenesis	2	0.00028	Klf2,Ptgs2
GO:0007611	learning or memory	3	0.00032	Il1b,Ptgs2,Tlr2
GO:0030098	lymphocyte differentiation	5	0.00032	Atp7a,Bcl3,Fas,Ptger4,Tnfaip3
GO:0010042	response to manganese ion	2	0.00035	Atp7a,Ptgs2

GO:0010667	negative regulation of cardiac muscle cell apoptotic process	2	0.00035	Cflar,Nfe2l2
GO:0061448	connective tissue development	2	0.00035	Atp7a,Pdgfb
GO:0071398	cellular response to fatty acid	2	0.00035	Ptger4,Zc3h12a
GO:1903792	negative regulation of anion transport	2	0.00035	Il1b,Tnf
GO:0007417	central nervous system development	2	0.00043	Atp7a,Tlr2
GO:0046887	positive regulation of hormone secretion	2	0.00043	Hcar2,Ptger4
GO:0071346	cellular response to interferon-gamma	2	0.00043	Cdc42ep2,Tlr2
GO:0097306	cellular response to alcohol	2	0.00043	Klf2,Ptger4
GO:0097756	negative regulation of blood vessel diameter	2	0.00043	Pdgfb,Ptgs2
GO:0002250	adaptive immune response	5	0.00044	Bcl3,Fas,Icam1,Icosl,Rnf19b
GO:0032651	regulation of interleukin-1 beta production	3	0.00047	Tlr2,Tnfaip3,Zc3h12a
GO:0001816	cytokine production	4	0.0005	Il1b,Ptgs2,Rnf19b,Tlr2

GO:0021782	glial cell development	2	0.00052	Pdgfb,Tlr2
GO:0032663	regulation of interleukin-2 production	3	0.00052	Cd83,Il1b,Tnfaip3
GO:0097192	extrinsic apoptotic signaling pathway in absence of ligand	2	0.00052	Fas,Il1b
GO:1903140	regulation of establishment of endothelial barrier	2	0.00052	Il1b,Tnf
GO:0031346	positive regulation of cell projection organization	6	0.00055	Atp7a,Cdc42ep2,Cflar,Ehd1,Nfe2l2,Skil
GO:0001501	skeletal system development	2	0.00061	Atp7a,Ptger4
GO:0002315	marginal zone B cell differentiation	2	0.00061	Bcl3,Tnfaip3
GO:0006925	inflammatory cell apoptotic process	2	0.00061	Fas,Hcar2
GO:0060538	skeletal muscle organ development	2	0.00061	Cflar,Skil
GO:2001240	negative regulation of extrinsic apoptotic signaling pathway in absence of ...	2	0.00061	Il1b,Tnf

GO:0032722	positive regulation of chemokine production	3	0.00062	Il1b,Tlr2,Tnf
GO:0034121	regulation of toll-like receptor signaling pathway	3	0.00062	Birc3,Tlr2,Tnfaip3
GO:0014911	positive regulation of smooth muscle cell migration	3	0.00065	Atp7a,Pdgfb,Ptger4
GO:0030217	T cell differentiation	4	0.00066	Atp7a,Bcl3,Fas,Ptger4
GO:0032874	positive regulation of stress-activated MAPK cascade	4	0.0007	Gadd45b,Il1b,Tnf,Zc3h12a
GO:0034116	positive regulation of heterotypic cell-cell adhesion	2	0.00071	Il1b,Tnf
GO:0072126	positive regulation of glomerular mesangial cell proliferation	2	0.00071	Cflar,Pdgfb
GO:1901566	organonitrogen compound biosynthetic process	3	0.00071	Atp7a,Il1b,Tnip1
GO:2001020	regulation of response to DNA damage stimulus	2	0.00071	Ier3,Skil
GO:0002260	lymphocyte homeostasis	3	0.00073	Fas,Skil,Tnfaip3

GO:0033043	regulation of organelle organization	9	0.0008	Cdc42ep2,Fas,Icam1,Ier3,Il1b,Pdgfb,Ptger4,Tlr2,Tnf
GO:0043467	regulation of generation of precursor metabolites and energy	2	0.0011	Atp7a,Ier3
GO:0043507	positive regulation of JUN kinase activity	2	0.0011	Il1b,Tnf
GO:1904062	regulation of cation transmembrane transport	2	0.0011	Atp7a,Slc31a2
GO:0045930	negative regulation of mitotic cell cycle	2	0.0012	Ier3,Tnf
GO:0030855	epithelial cell differentiation	6	0.0013	Icam1,Klf2,Pdgfb,Ptgs2,Rilpl2,Skil
GO:0032691	negative regulation of interleukin-1 beta production	2	0.0014	Tnfaip3,Zc3h12a
GO:0007162	negative regulation of cell adhesion	2	0.0015	Tnfaip3,Zc3h12a
GO:0060546	negative regulation of necroptotic process	2	0.0015	Birc3,Cflar
GO:0042060	wound healing	2	0.0017	Cflar,Pdgfb

GO:0043124	negative regulation of I-kappaB kinase/NF-kappaB signaling	3	0.0017	Tnfaip3,Tnip1,Zc3h12a
GO:1904468	negative regulation of tumor necrosis factor secretion	2	0.0017	Ptger4,Zc3h12a
GO:0000904	cell morphogenesis involved in differentiation	2	0.0018	Atp7a,Rilpl2
GO:0007613	memory	2	0.0018	Il1b,Ptgs2
GO:0045080	positive regulation of chemokine biosynthetic process	2	0.0018	Il1b,Tnf
GO:0031669	cellular response to nutrient levels	4	0.0019	Fas,Nfe2l2,Ptgs2,Zc3h12a
GO:0008625	extrinsic apoptotic signaling pathway via death domain receptors	2	0.0022	Fas,Tnf
GO:0036003	positive regulation of transcription from RNA polymerase II promoter in response ...	2	0.0022	Klf2,Nfe2l2
GO:0045727	positive regulation of translation	2	0.0022	Bcl3,Tnf

GO:0000185	activation of MAPKKK activity	2	0.0026	Gadd45b,Tnf
GO:0031334	positive regulation of protein complex assembly	4	0.0026	Cdc42ep2,Fas,Icam1,Tnf
GO:1903003	positive regulation of protein deubiquitination	2	0.0032	Tnip1,Zc3h12a
GO:0032673	regulation of interleukin-4 production	2	0.0037	Cd83,Icosl
GO:0042133	neurotransmitter metabolic process	2	0.004	Atp7a,Tlr2
GO:2000121	regulation of removal of superoxide radicals	2	0.0042	Nfe2l2,Tnf
GO:0032743	positive regulation of interleukin-2 production	2	0.0045	Cd83,Il1b
GO:0034763	negative regulation of transmembrane transport	3	0.0048	Atp7a,Il1b,Tnf
GO:0034250	positive regulation of cellular amide metabolic process	3	0.005	Bcl3,Nfe2l2,Tnf
GO:0031667	response to nutrient levels	5	0.0051	Fas,Il1b,Nfe2l2,Ptgs2,Zc3h12a

GO:0030833	regulation of actin filament polymerization	3	0.006	Cdc42ep2,Icam1,Tlr2
GO:0032147	activation of protein kinase activity	4	0.0062	Gadd45b,Il1b,Pdgfb,Tnf
GO:0042509	regulation of tyrosine phosphorylation of STAT protein	2	0.0068	Ptger4,Socs3
GO:0050954	sensory perception of mechanical stimulus	2	0.0068	Icam1,Tnf
GO:0033365	protein localization to organelle	5	0.0077	Bcl3,Ehd1,Nfkbia,Tnf,Tnfaip3
GO:0071695	anatomical structure maturation	2	0.0078	Klf2,Pdgfb
GO:0060559	positive regulation of calcidiol 1-monooxygenase activity	2	0.0085	Il1b,Tnf
GO:0031099	regeneration	3	0.0087	Cflar,Fas,Nfkbia
GO:0034762	regulation of transmembrane transport	5	0.0091	Atp7a,Il1b,Nfe2l2,Slc31a2,Tnf
GO:0042149	cellular response to glucose starvation	2	0.0103	Nfe2l2,Zc3h12a
GO:0033036	macromolecule localization	10	0.0109	Bcl3,Ehd1,Herpud1,Il1b,Nfkbia,Rilpl2,Rnf19b,Tlr2,Tnf,Tnfaip3

GO:0034248	regulation of cellular amide metabolic process	4	0.0118	Bcl3,Nfe2l2,Tnf,Zc3h12a
GO:0032535	regulation of cellular component size	4	0.0119	Atp7a,Cdc42ep2,Icam1,Tlr2
GO:0042534	regulation of tumor necrosis factor biosynthetic process	2	0.0119	Bcl3,Tlr2
GO:0031327	negative regulation of cellular biosynthetic process	8	0.012	Bcl3,Ier3,Il1b,Klf2,Pdgfb,Skil,Tnf,Zc3h12a
GO:0022607	cellular component assembly	10	0.0123	Atp7a,Birc3,Cflar,Ehd1,Fas,Pdgfb,Rilpl2,Skil,Tlr2,Zc3h12a
GO:0035272	exocrine system development	2	0.0127	Pdgfb,Tnf
GO:0032386	regulation of intracellular transport	4	0.0133	Ehd1,Ier3,Ptgs2,Zc3h12a
GO:0034620	cellular response to unfolded protein	2	0.0136	Herpud1,Nfe2l2
GO:0031396	regulation of protein ubiquitination	3	0.0139	Birc3,Herpud1,Tnfaip3
GO:0034613	cellular protein localization	7	0.0149	Bcl3,Ehd1,Herpud1,Nfkbia,Rilpl2,Tnf,Tnfaip3
GO:0023014	signal transduction by protein phosphorylation	3	0.0157	Il1b,Ptger4,Tnf

GO:0032370	positive regulation of lipid transport	2	0.0163	Il1b,Nfkbia
GO:0032388	positive regulation of intracellular transport	3	0.017	Ehd1,Ptgs2,Zc3h12a
GO:0043588	skin development	2	0.0198	Atp7a,Ptgs2
GO:0030036	actin cytoskeleton organization	4	0.0203	Cdc42ep2,Cflar,Pdgfb,Tnf
GO:0002064	epithelial cell development	3	0.0206	Icam1,Pdgfb,Rilpl2
GO:0030193	regulation of blood coagulation	2	0.023	Nfe2l2,Pdgfb
GO:0030099	myeloid cell differentiation	3	0.024	Klf2,Tlr2,Tnf
GO:0030838	positive regulation of actin filament polymerization	2	0.0241	Cdc42ep2,Icam1
GO:0032436	positive regulation of proteasomal ubiquitin-dependent protein catabolic process	2	0.0295	Herpud1,Rnf19b
GO:0034766	negative regulation of ion transmembrane transport	2	0.0346	Atp7a,Tnf

GO:0033138	positive regulation of peptidyl-serine phosphorylation	2	0.0401	Ptgs2,Tnf
GO:0030595	leukocyte chemotaxis	2	0.0482	Il1b,Pdgfb
