# Supplementary material for: Integrative Transcriptomic Profiling Identifies TNF and IL1B as Candidate Key Early-Response Genes in Macrophages Infected with Smooth Brucella Using a Comprehensive Bioinformatic Approach
Source: Biology (Basel). 2025 May 21;14(5):579. doi: 10.3390/biology14050579 (PMC12109160; doi:10.3390/biology14050579)
Supplement: Supplementary file 1 [file biology-14-00579-s001.zip › Table S6.pdf]

Table S6 Reactome pathways enriched by 45 common differentially expressed genes

pathway ID	pathway description	observed gene count	false discovery rate	matching proteins in your network (labels)
MMU-162582	Signal Transduction	14	2.32E-37	Ccrl2,Cdc42ep2,Cflar,Dusp6,Fas,Hcar2,Nfkbia,Pde4b,Ptger4,Skil,Socs3,Tnf,Tnfaip3,Traf1
MMU-168256	Immune System	12	5.70E-32	Atp7a,Dusp6,Icam1,Icosl,Il10ra,Il1b,Nfkbia,Nfkbie,Rnf19b,Socs3,Tnf,Tnfaip3
MMU-1280215	Cytokine Signaling in Immune system	6	2.22E-15	Dusp6,Il10ra,Il1b,Nfkbia,Socs3,Tnf
MMU-1280218	Adaptive Immune System	6	2.22E-15	Icam1,Icosl,Nfkbia,Nfkbie,Rnf19b,Socs3
MMU-73887	Death Receptor Signalling	6	2.22E-15	Cflar,Fas,Nfkbia,Tnf,Tnfaip3,Traf1
MMU-168249	Innate Immune System	5	6.28E-13	Atp7a,Dusp6,Il1b,Nfkbia,Tnfaip3

MMU-449147	Signaling by Interleukins	5	6.28E-13	Dusp6,Il10ra,Il1b,Nfkbia,Socs3
MMU-372790	Signaling by GPCR	4	2.60E-10	Ccrl2,Hcar2,Pde4b,Ptger4
MMU-392499	Metabolism of proteins	4	2.60E-10	Nfkbia,Socs3,Tnfaip3,Tnip1
MMU-373076	Class A/1 (Rhodopsin-like receptors)	3	1.02E-07	Ccrl2,Hcar2,Ptger4
MMU-388396	GPCR downstream signalling	3	1.02E-07	Hcar2,Pde4b,Ptger4
MMU-500792	GPCR ligand binding	3	1.02E-07	Ccrl2,Hcar2,Ptger4
MMU-5357905	Regulation of TNFR1 signaling	3	1.02E-07	Tnf,Tnfaip3,Traf1
MMU-5357956	TNFR1-induced NFkappaB signaling pathway	3	1.02E-07	Tnf,Tnfaip3,Traf1

MMU-5688426	Deubiquitination	3	1.02E-07	Nfkbia,Tnfaip3,Tnip1
MMU-109581	Apoptosis	2	3.27E-05	Cflar,Fas
MMU-1168372	Downstream signaling events of B Cell Receptor (BCR)	2	3.27E-05	Nfkbia,Nfkbie
MMU-140534	Caspase activation via Death Receptors in the presence of ligand	2	3.27E-05	Cflar,Fas
MMU-166016	Toll Like Receptor 4 (TLR4) Cascade	2	3.27E-05	Dusp6,Nfkbia
MMU-168138	Toll Like Receptor 9 (TLR9) Cascade	2	3.27E-05	Dusp6,Nfkbia
MMU-168142	Toll Like Receptor 10 (TLR10) Cascade	2	3.27E-05	Dusp6,Nfkbia
MMU-168164	Toll Like Receptor 3 (TLR3) Cascade	2	3.27E-05	Dusp6,Nfkbia

MMU-168176	Toll Like Receptor 5 (TLR5) Cascade	2	3.27E-05	Dusp6,Nfkbia
MMU-168179	Toll Like Receptor TLR1:TLR2 Cascade	2	3.27E-05	Dusp6,Nfkbia
MMU-168181	Toll Like Receptor 7/8 (TLR7/8) Cascade	2	3.27E-05	Dusp6,Nfkbia
MMU-168188	Toll Like Receptor TLR6:TLR2 Cascade	2	3.27E-05	Dusp6,Nfkbia
MMU-168898	Toll-like Receptor Cascades	2	3.27E-05	Dusp6,Nfkbia
MMU-168928	DDX58/IFIH1-mediated induction of interferon-alpha/beta	2	3.27E-05	Nfkbia,Tnfaip3
MMU-418555	G alpha (s) signalling events	2	3.27E-05	Pde4b,Ptger4
MMU-418594	G alpha (i) signalling events	2	3.27E-05	Hcar2,Pde4b

MMU-446652	Interleukin-1 family signaling	2	3.27E-05	Il1b,Nfkbia
MMU-5213460	RIPK1-mediated regulated necrosis	2	3.27E-05	Cflar,Fas
MMU-5218859	Regulated Necrosis	2	3.27E-05	Cflar,Fas
MMU-5218900	CASP8 activity is inhibited	2	3.27E-05	Cflar,Fas
MMU-5357786	TNFR1-induced proapoptotic signaling	2	3.27E-05	Tnf,Tnfaip3
MMU-5607764	CLEC7A (Dectin-1) signaling	2	3.27E-05	Il1b,Nfkbia
MMU-5621481	C-type lectin receptors (CLRs)	2	3.27E-05	Il1b,Nfkbia
MMU-5683057	MAPK family signaling cascades	2	3.27E-05	Cdc42ep2,Dusp6

MMU-5689896	Ovarian tumor domain proteases	2	3.27E-05	Tnfaip3,Tnip1
MMU-73857	RNA Polymerase II Transcription	2	3.27E-05	Ell2,Skil
MMU-74160	Gene expression (Transcription)	2	3.27E-05	Ell2,Skil
MMU-937061	TRIF(TICAM1)-mediated TLR4 signaling	2	3.27E-05	Dusp6,Nfkbia
MMU-975138	TRAF6 mediated induction of NFkB and MAP kinases upon TLR7/8 or 9 activation	2	3.27E-05	Dusp6,Nfkbia
MMU-983168	Antigen processing: Ubiquitination & Proteasome degradation	2	3.27E-05	Rnf19b,Socs3
MMU-983705	Signaling by the B Cell Receptor (BCR)	2	3.27E-05	Nfkbia,Nfkbie
